# Supplementary material for: Conformational Analysis of 1,5-Diaryl-3-Oxo-1,4-Pentadiene Derivatives: A Nuclear Overhauser Effect Spectroscopy Investigation
Source: Int J Mol Sci. 2023 Nov 24;24(23):16707. doi: 10.3390/ijms242316707 (PMC10706324; doi:10.3390/ijms242316707)
Supplement: Supplementary file 1 [file ijms-24-16707-s001.zip › ijms-2699139-supplementary.pdf]

Supplementary materials

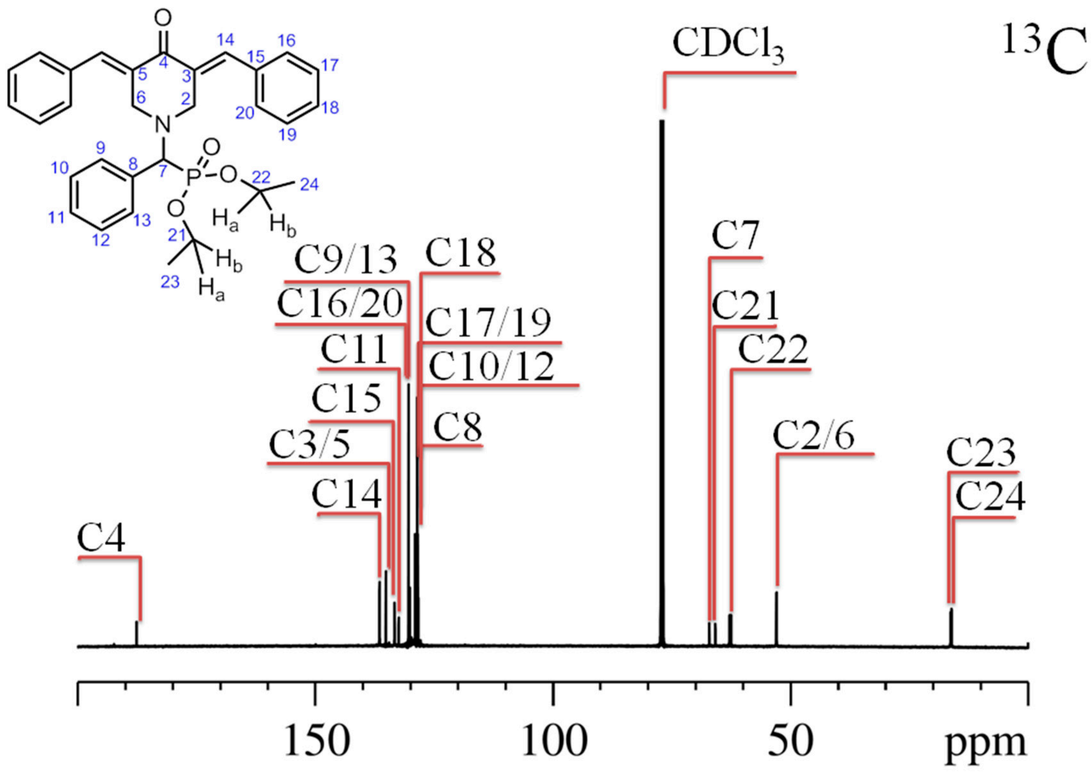

Figure S1. ^{13}C NMR spectrum of compound 1 in $CDCl_3$

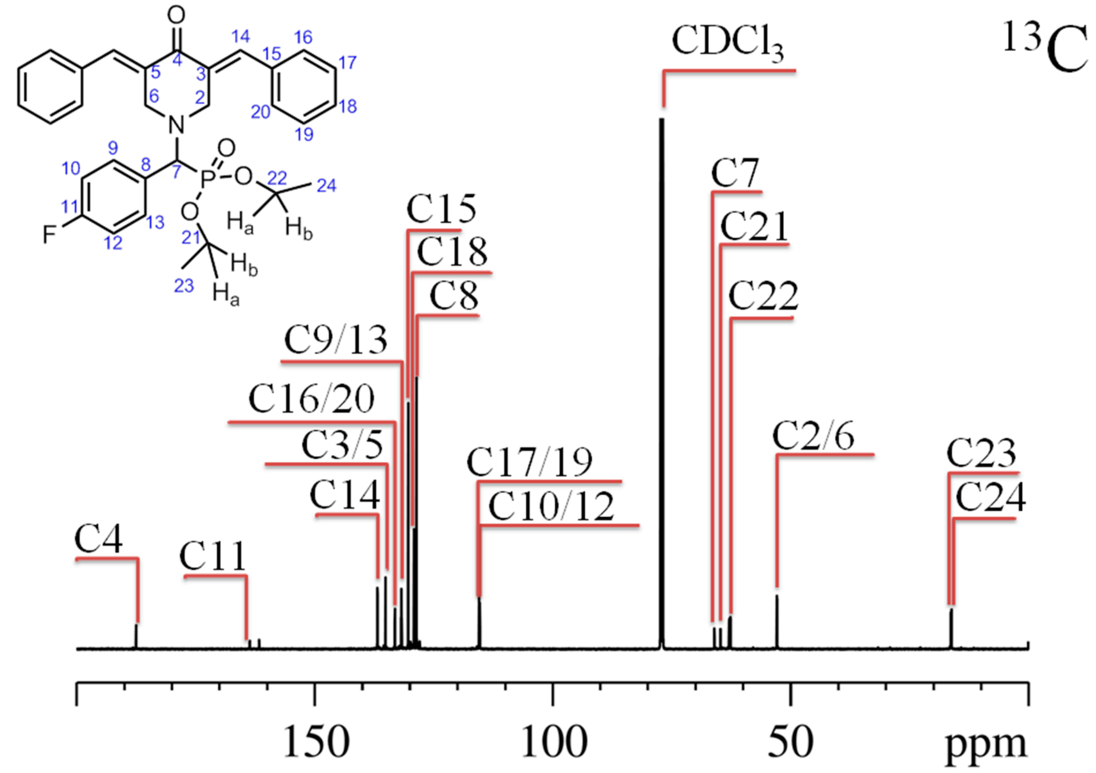

Figure S2. ^{13}C NMR spectrum of ***compound 2*** in $CDCl_3$

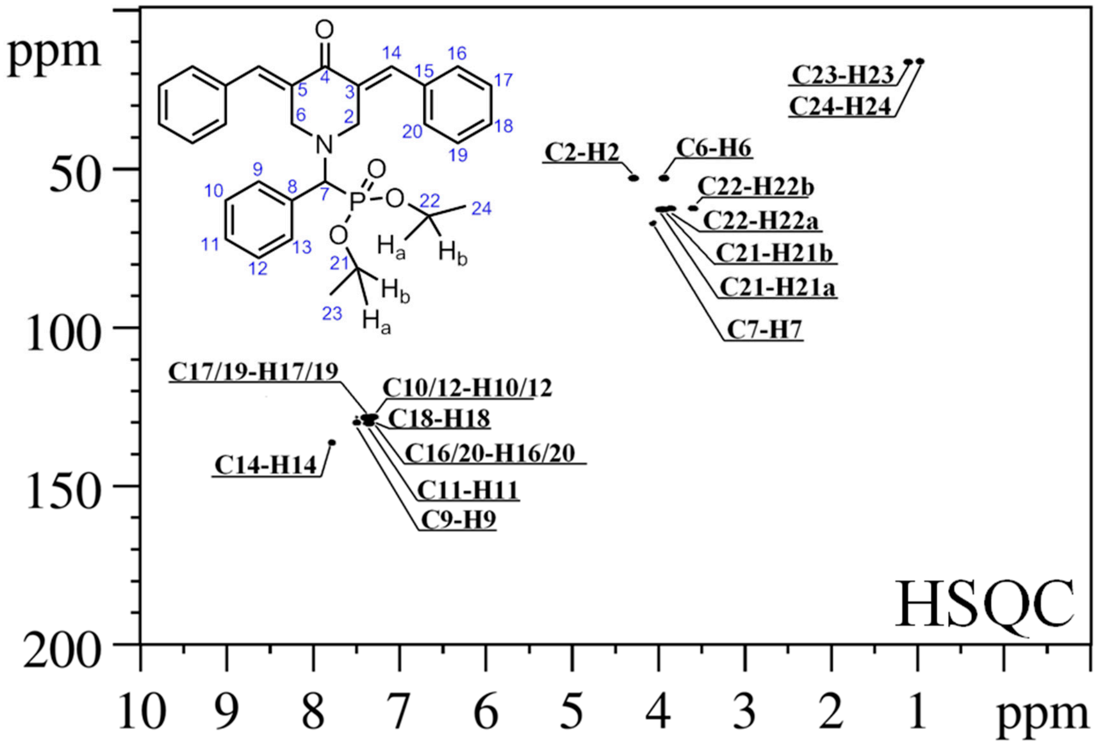

Figure S3. ^{1}H-^{13}C HSQC spectrum of ***compound 1*** in $CDCl_3$

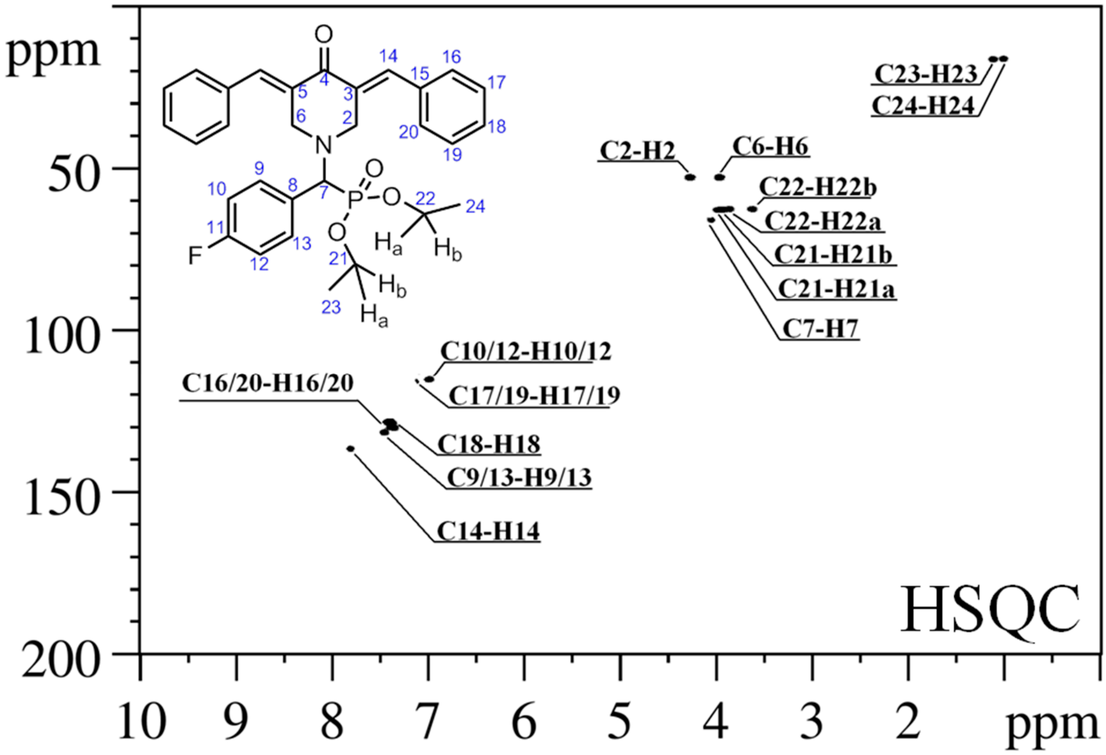

Figure S4. ^{1}H-^{13}C HSQC spectrum of ***compound 2*** in $CDCl_3$

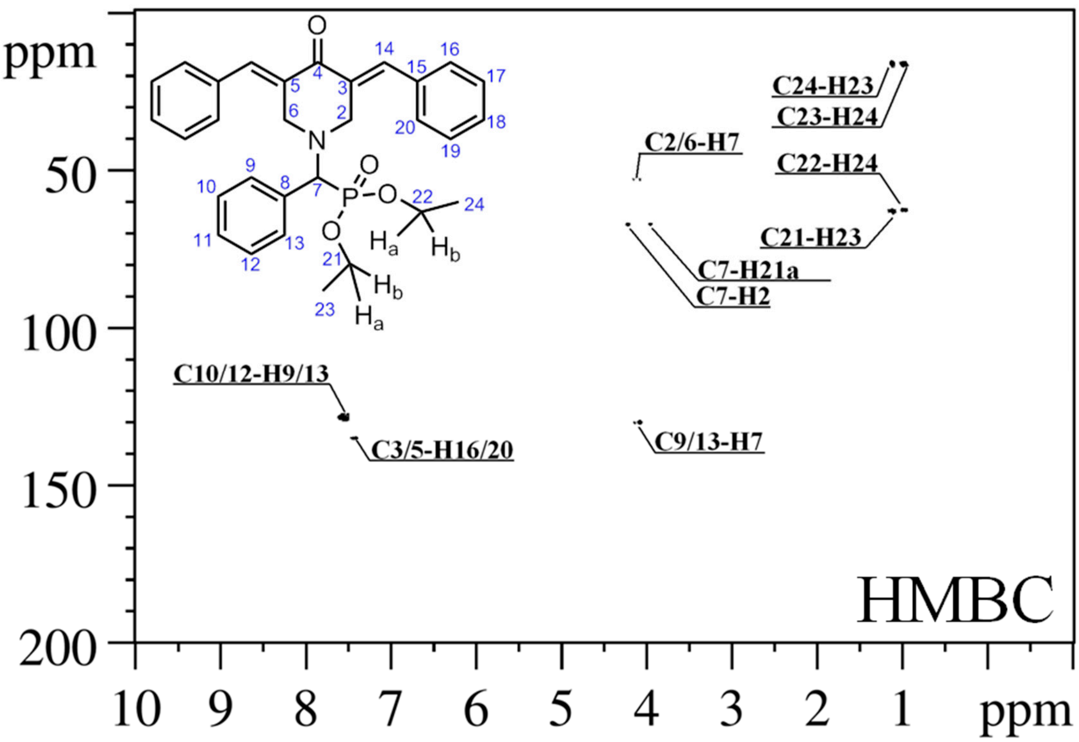

Figure S5. ^{1}H-^{13}C HMBC spectrum of ***compound 1*** in $CDCl_3$

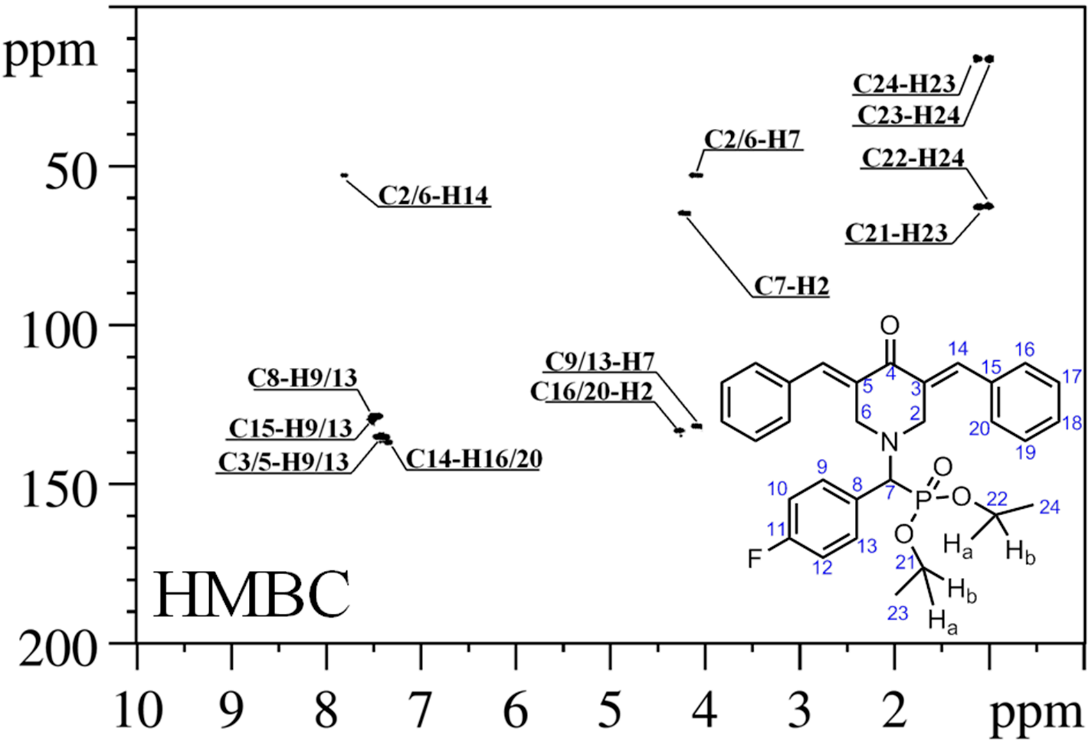

Figure S6. ^{1}H-^{13}C HMBC spectrum of ***compound 2*** in $CDCl_3$

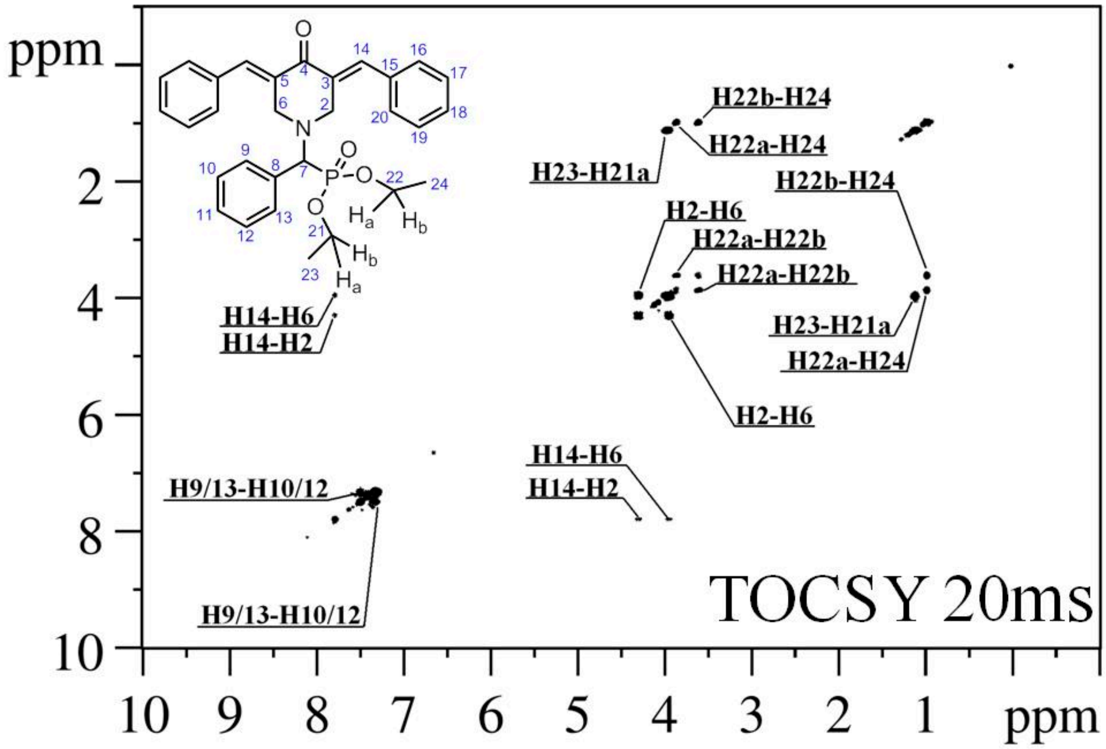

Figure S7. ^{1}H-^{1}H TOCSY spectrum of ***compound 1*** in $CDCl_3$
(mixing time 20 ms)

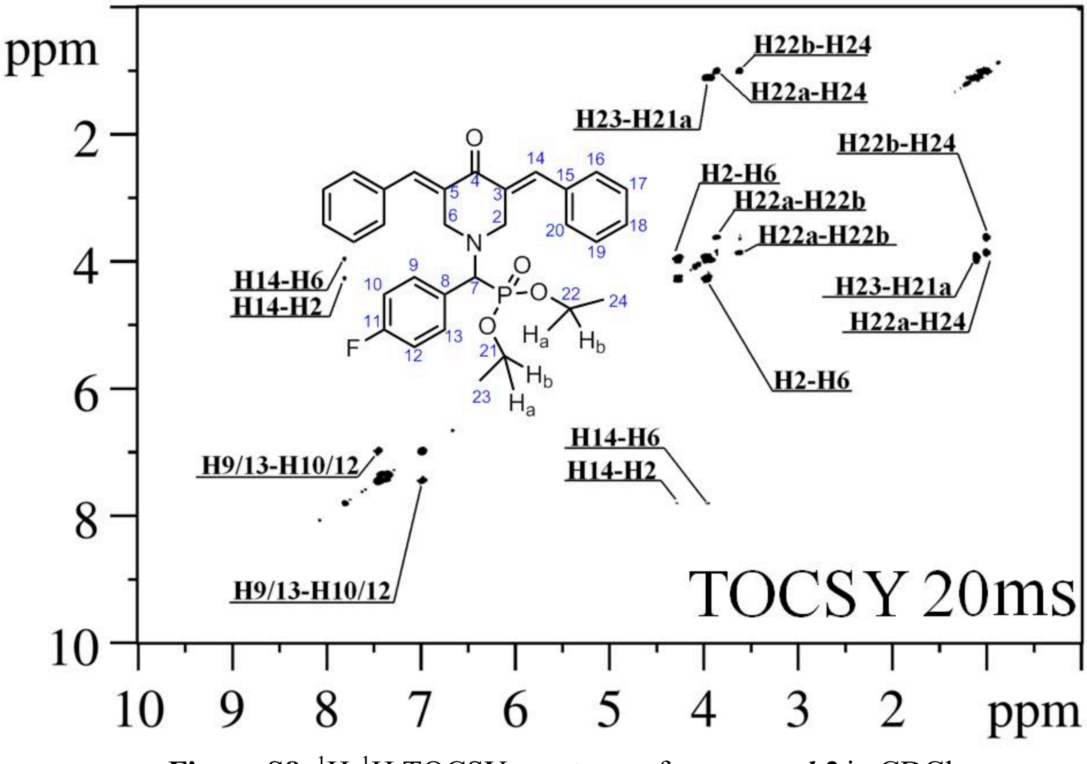

Figure S8. ^{1}H-^{1}H TOCSY spectrum of ***compound 2*** in $CDCl_3$
(mixing time 20 ms)

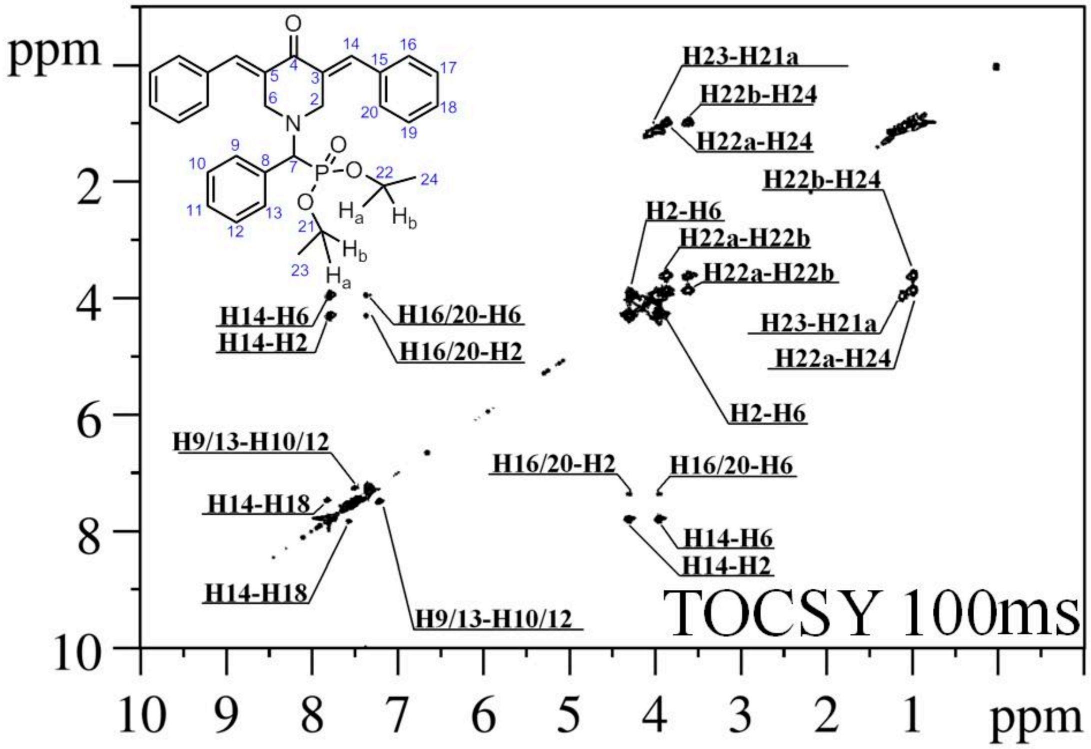

Figure S9. ${}^{1}H$-${}^{1}H$ TOCSY spectrum of ***compound 1*** in $CDCl_3$
(mixing time 100 ms)

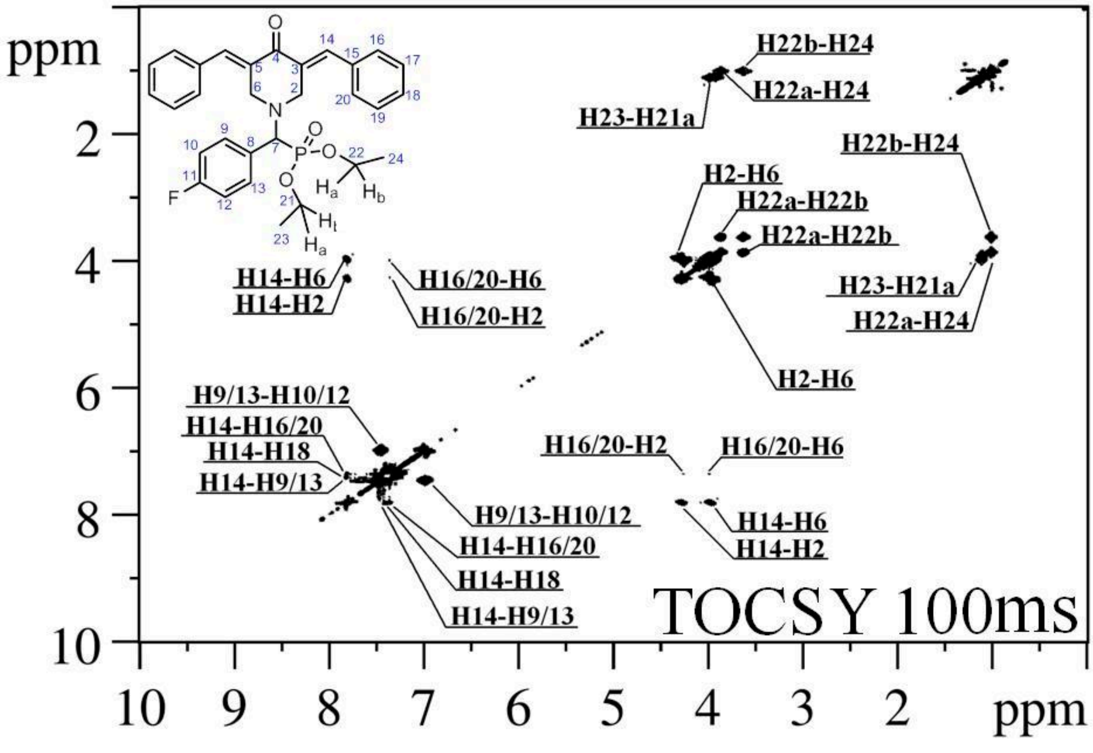

Figure S10. ${}^{1}H$-${}^{1}H$ TOCSY spectrum of ***compound 2*** in $CDCl_3$
(mixing time 100 ms)

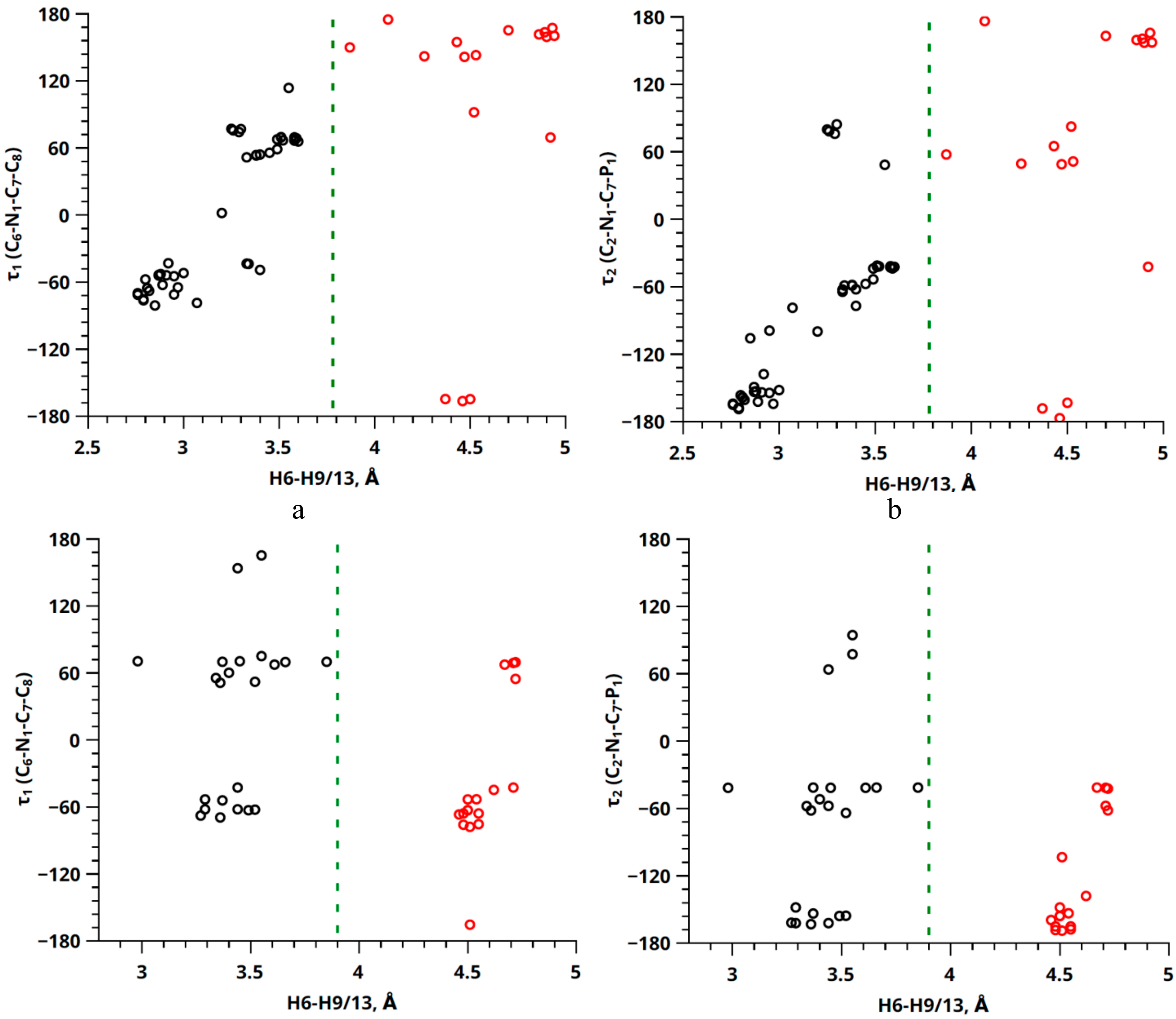

a b

c d

Figure S11. Angle values of different conformers A (black dots) and B (red dots) versus distances H6/H9-H13 for compound 1 (a,b) and compound 2 (c,d).

Table S1. ^{1}H and ^{13}C chemical shifts and observed cross-correlation peaks in 2D spectra of *compound 1* in $CDCl_3$

^{13}C	Δ ^{13}C	^{1}H	δ ^{1}H	HMBC	HSQC	TOCSY 20 ms	TOCSY 100 ms	NOESY
C2/6	62.55	H2	4.30	C2/6-H7	C2-H2	H2-H6	H2-H6	H2-H6<br>H2-H9/13<br>H2-H11
C3/5	136.49	-	-	C3/5-H16/20	-	-	-	-
C4	187.62	H6	3.96	-	C6-H6	-	-	H6-H9/13<br>H6-H11
C7	67.09	H7	4.09	C7-H21a<br>C7-H2	C7-H7	-	-	H7-H9/13<br>H7-H11
C8	128.33	-	-	-	-	-	-	-
C9/13	130.16	H9/13	7.50	C9/13-H7	C9/13-H9/13	H9/13-H10/12	H9/13-H10/12	H9/13-H10/12<br>H9/13-H23<br>H9/13-H24
C10/12	128.42	H10/12	7.29	C10/12-H9/13	C10/12-H10/12	-	-	-
C11	132.45	H11	7.34	-	C11-H11	-	-	-
C14	135.17	H14	7.79	-	C14-H14	H14-H2<br>H14-H6	H14-H2<br>H14-H6	H14-H2<br>H14-H6
C15	133.34	-	-	-	-	-	-	-
C16/20	130.36	H16/20	7.35	-	C16/20-H16/20	-	H16/20-H2<br>H16/20-H6	H16/20-H23
C17/19	128.56	H17/19	7.39	-	C17/19-H17/19	-	-	-
C18	129.11	H18	7.31	-	C18-H18	-	-	-
C21	65.82	H21a	3.97	C21-H23	C21-H21a	-	-	-
		H21b	3.94	-	C21-H21b	-	-	-
C22	62.84	H22a	3.87	C22-H24	C22-H22a	H22a-H22b<br>H22a-H24	H22a-H22b<br>H22a-H24	H22a-H22b<br>H22a-H24
		H22b	3.61	-	C22-H22b	H22b-H24	H22b-H24	-

C23	16.30	H23	1.12	C23-H24	C23-H23	H23-H21a	H23-H21a	H23-H21a
C24	16.12	H24	0.99	C24-H23	C24-H24	-	-	-

Table S2. ^{1}H and ^{13}C chemical shifts and observed cross-correlation peaks in 2D spectra of compound 2 in $CDCl_3$

^{13}C	$\delta\ ^{13}C$	^{1}H	$\delta\ ^{1}H$	HMBC	HSQC	TOCSY		NOESY
						20 ms	100 ms	
C2/6	52.83	H2	4.25	C2/6-H7<br>C2/6-H14	C2-H2	H2-H6<br>H2-H14	H2-H6<br>H2-H14	H2-H6<br>H2-H14<br>H2-H9/13<br>H2-H11<br>H2-H7
C3/5	135.08	-	-	C3/5-H9/13	-	-	-	-
C4	187.52	H6	3.93	-	C6-H6	H6-H14	H6-H14	H6-H14<br>H6-H9/13<br>H6-H11
C7	65.97	H7	4.05	C7-H2	C7-H7	-	-	H7-H9/13
C8	128.57	-	-	C8-H9/13	-	-	-	-
C9/13	131.74	H9/13	7.43	C9/13-H7	C9/13-H9/13	H9/13-H10/12	H9/13-H10/12	H9/13-H10/12<br>H9/13-H24
C10/12	115.26	H10/12	6.96	-	C10/12-H10/12	-	-	-
C11	163.61	-	-	-	-	-	-	-
C14	136.78	H14	7.78	C14-H16/20	C14-H14	-	H14-H16/20<br>H14-H18<br>H14-H9/13	H14-H16/20
C15	130.33	-	-	C15-H9/13	-	-	-	-
C16/20	133.11	H16/20	7.34	C16/20-H2	-	-	H16/20-H2<br>H16/20-H6	H16/20-H23
C17/19	115.43	H17/19	7.06	-	C17/19-H17/19	-	-	-
C18	129.11	H18	7.39	-	C18-H18	-	-	H18-H23
C21	62.85	H21a	3.96	C21-H23	C21-H21a	H21a-H23	H21a-H23	H21a-H23

		H21b	3.88	-	C21-H21b	-	-	-
C22	62.58	H22a	3.83	C22-H24	C22-H22a	H22a-<br>H22b<br>H22a-H24	H22a-H22b<br>H22a-H24	H22a-H22b<br>H22a-H24
		H22b	3.60	-	C22-H22b	H22b-H24	H22b-H24	H22b-H24
C23	16.29	H23	1.09	C23-H24	C23-H23	-	-	-
C24	16.15	H24	0.99	C24-H23	C24-H24	-	-	-

Table S3. Spatial structure of the conformers of *compound 1*, determined from quantum chemical calculations

A1	A2
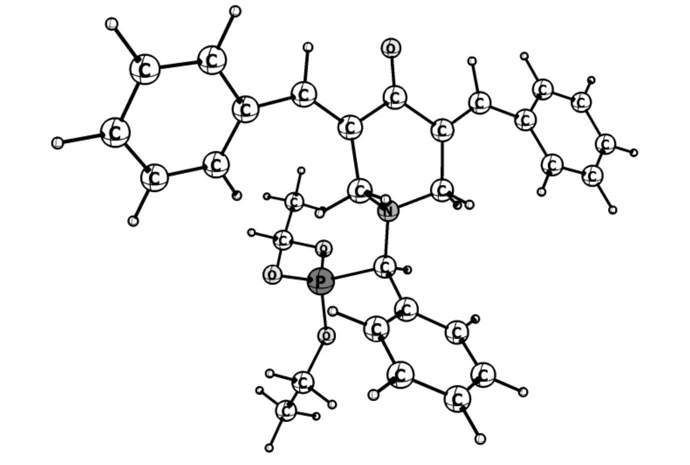	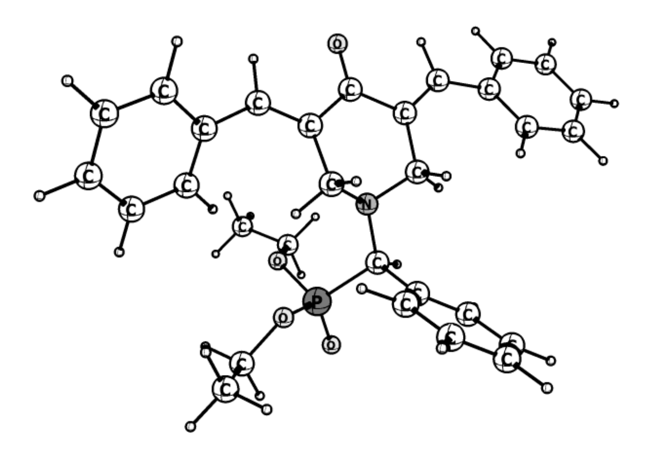
A3	A4
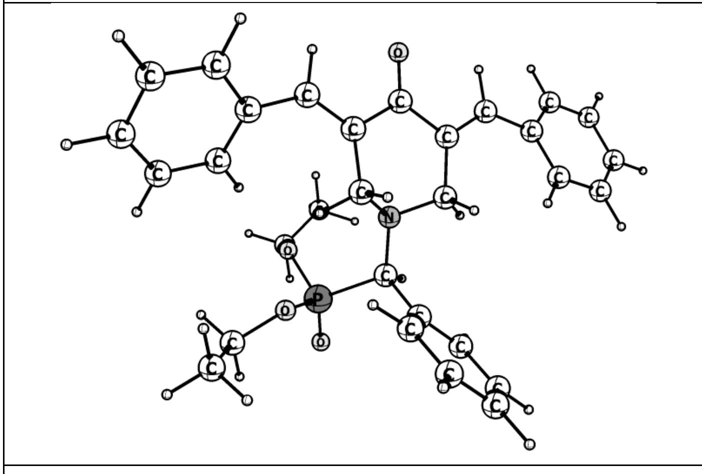	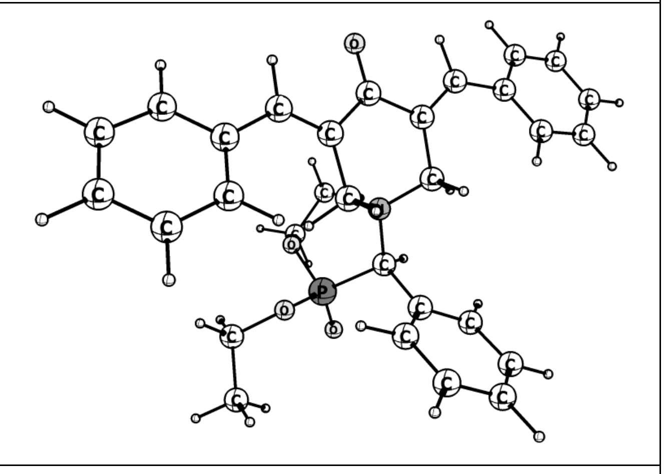
A5	A6

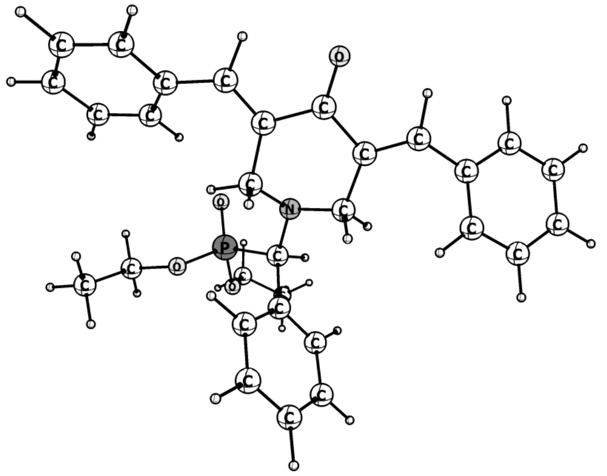	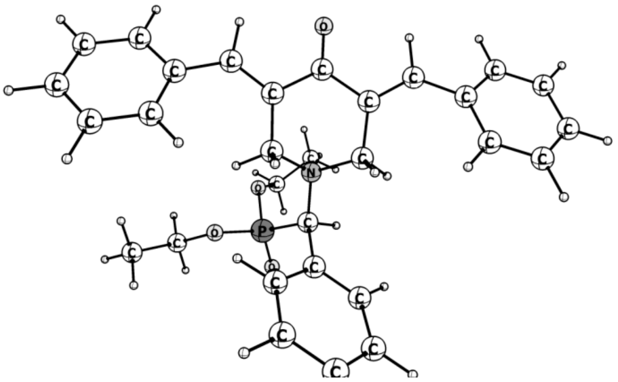
A7	A8
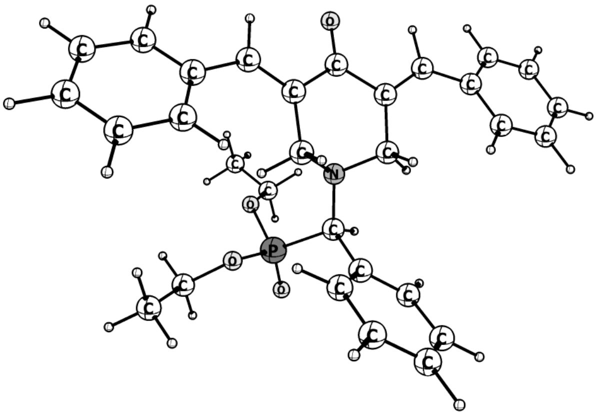	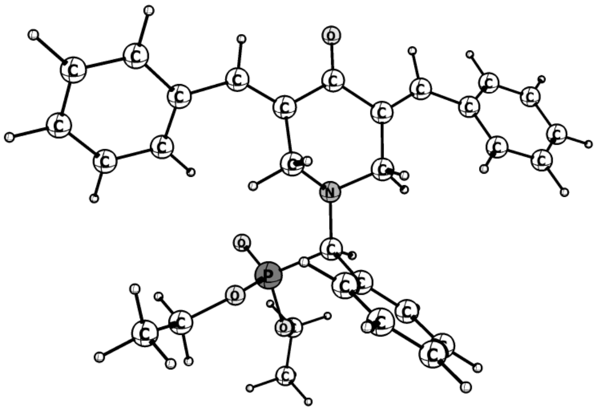
A9	A10
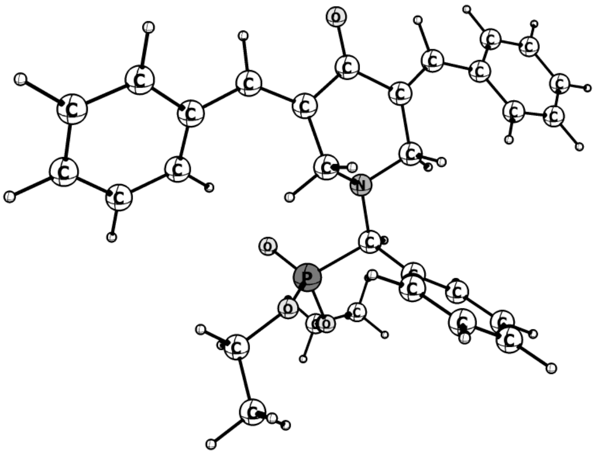	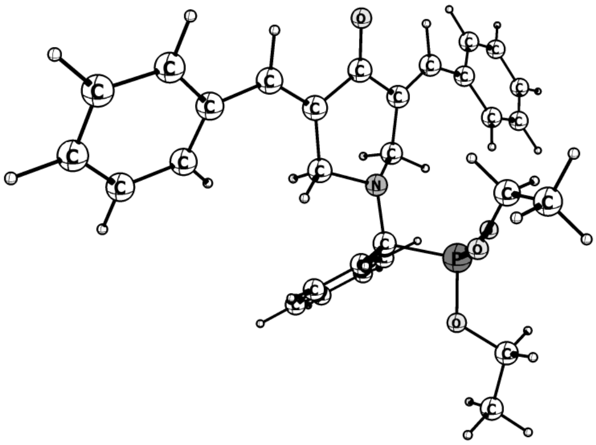
A11	A12

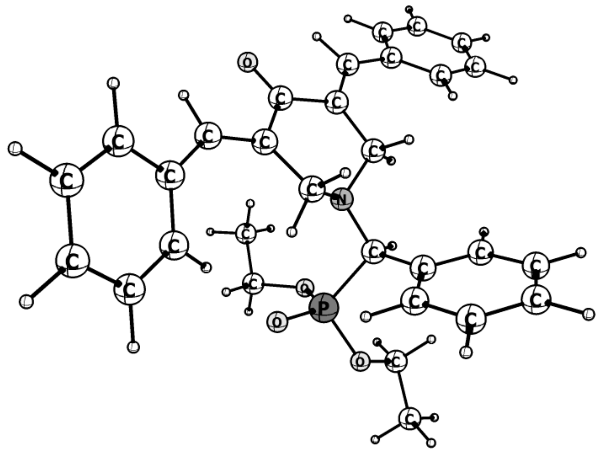

A13

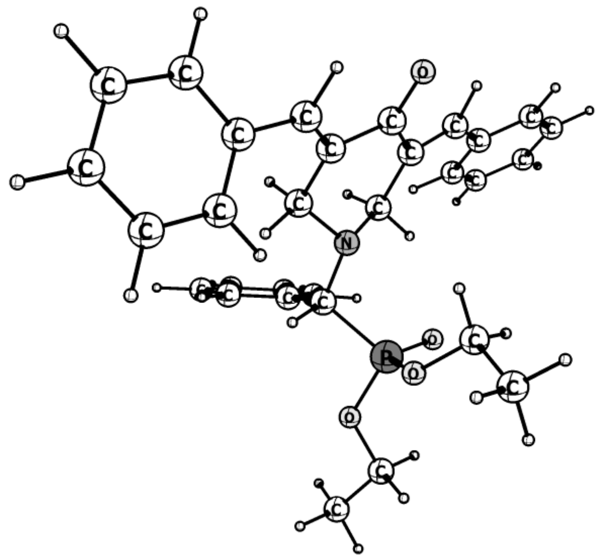

A14

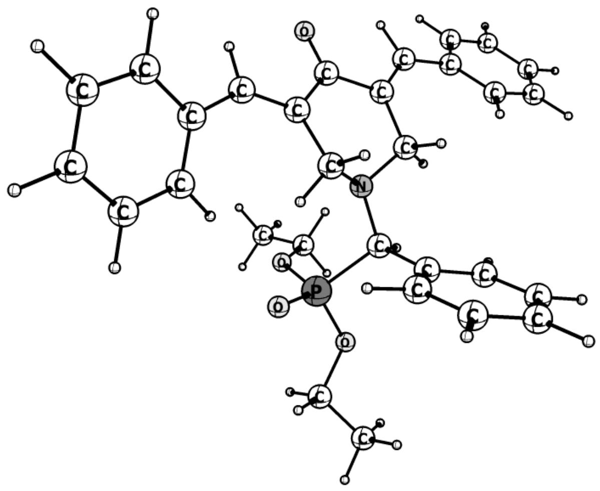

A15

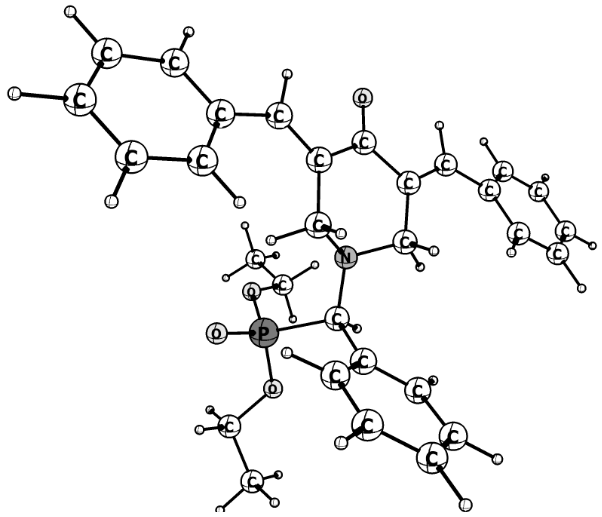

A16

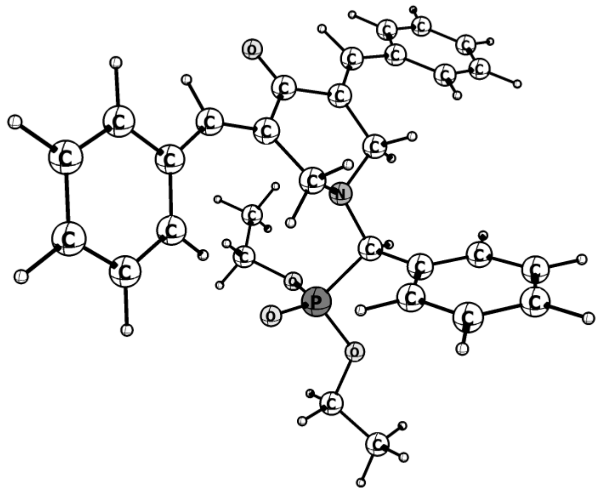

A17

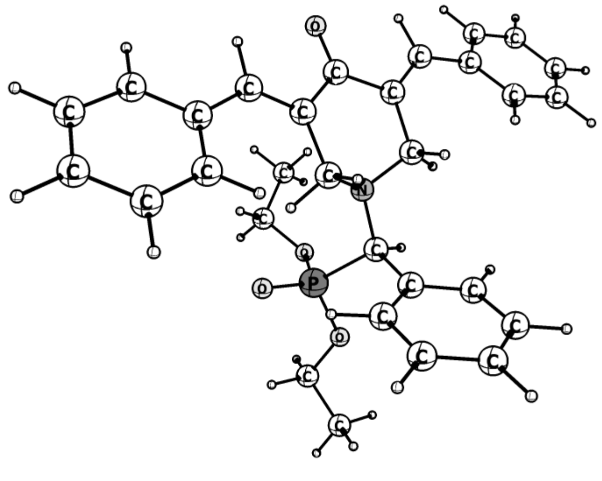

A18

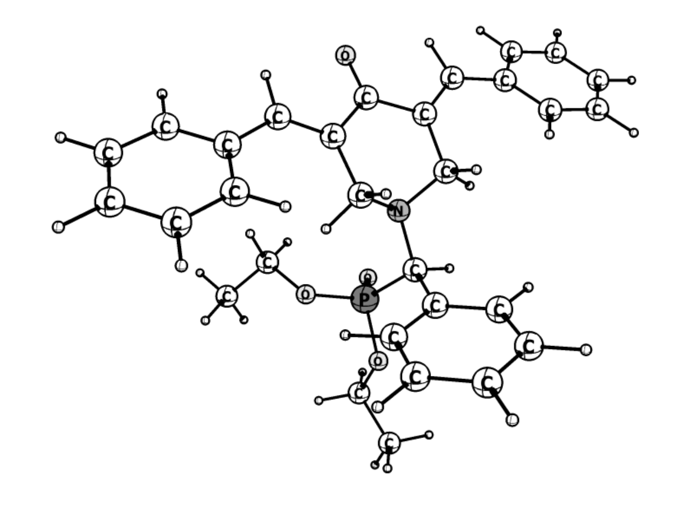

A19

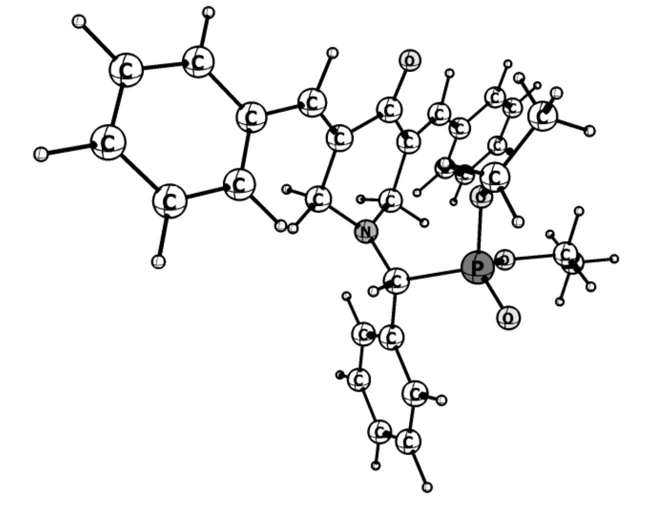

A20

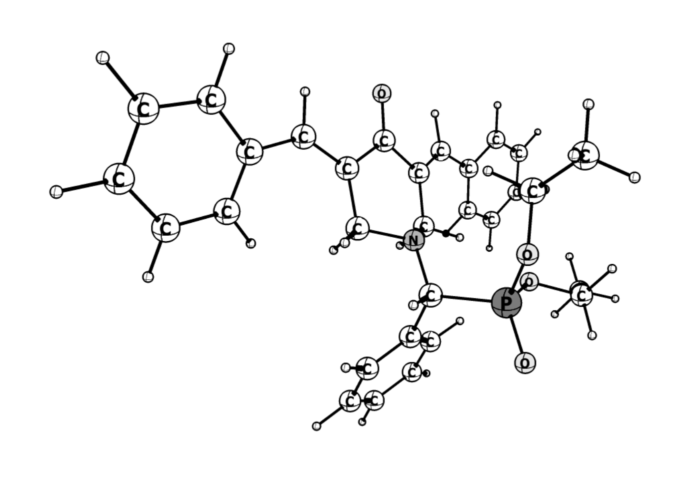

A21

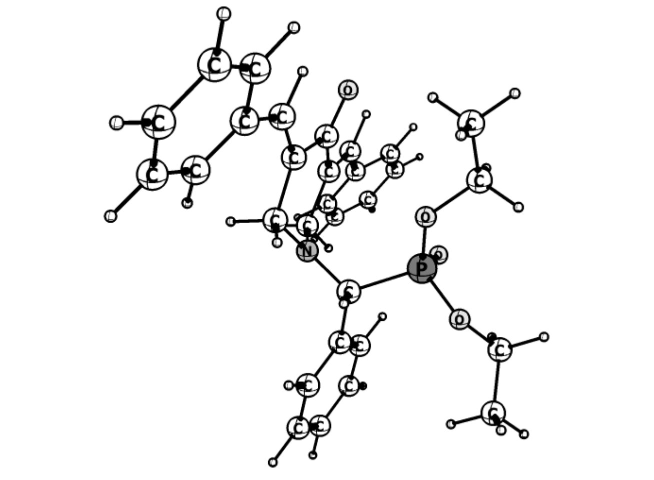

A22

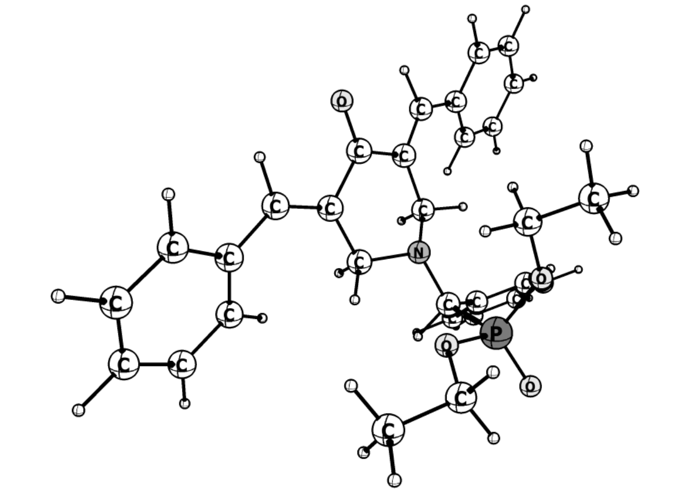

A23

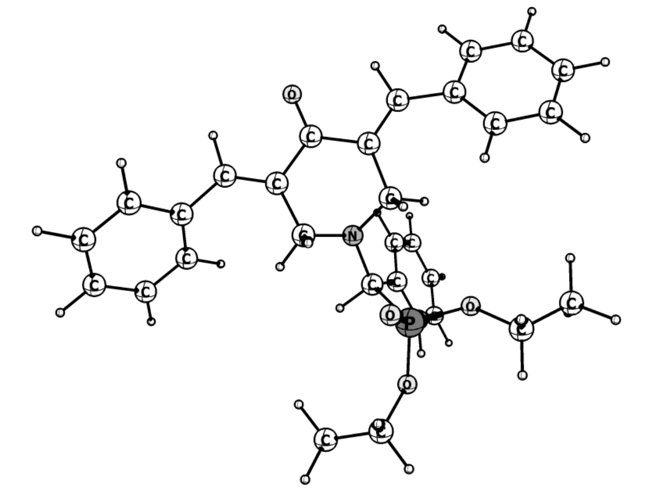

A24

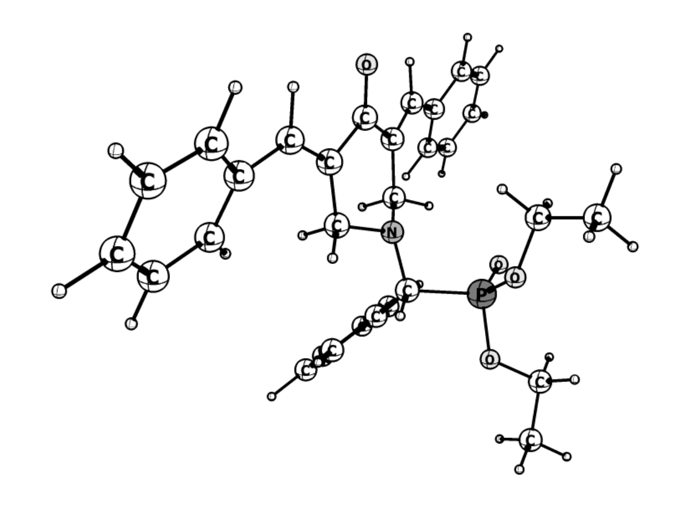
A25

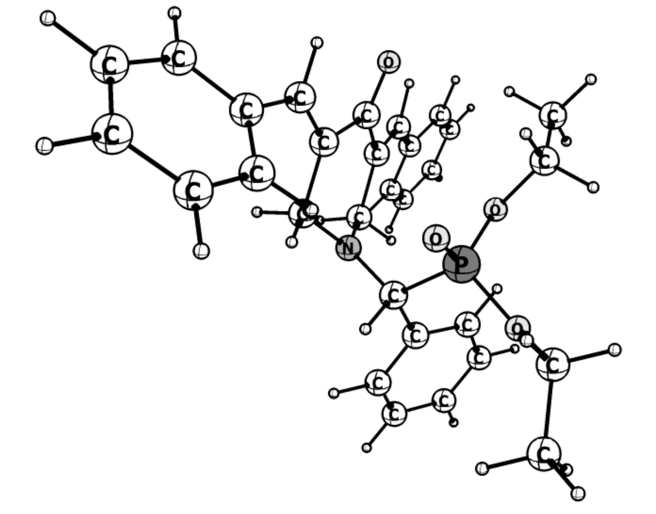
A26

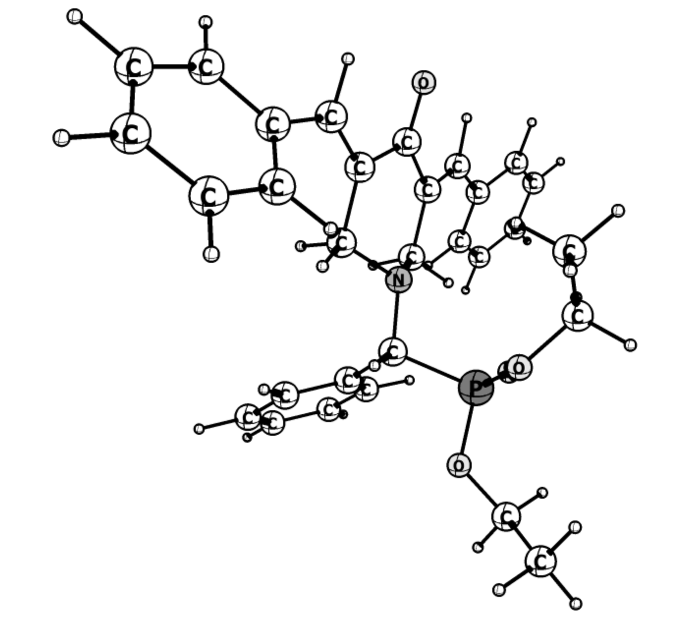
A27

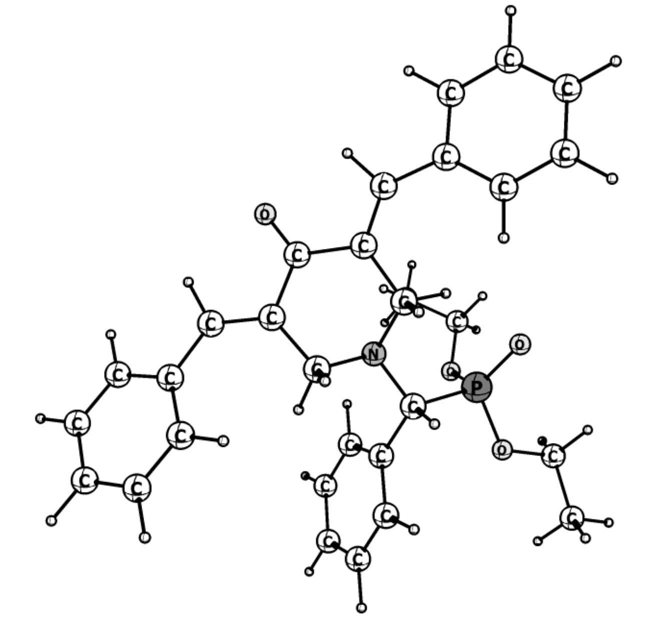
A28

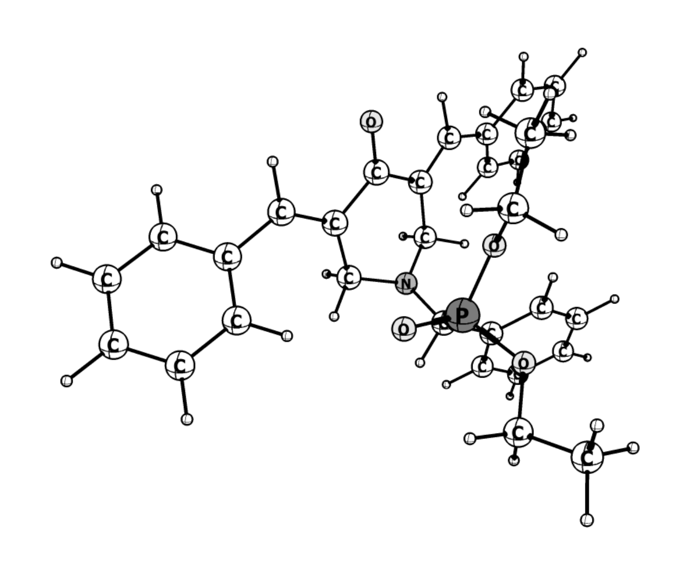
A29

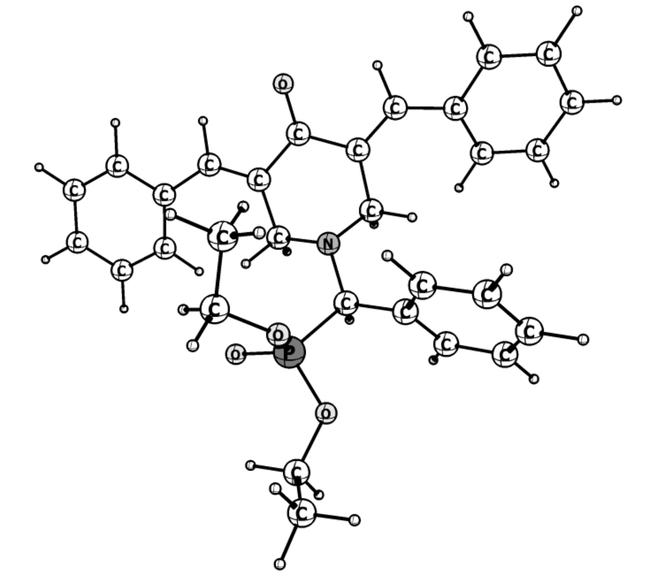
A30

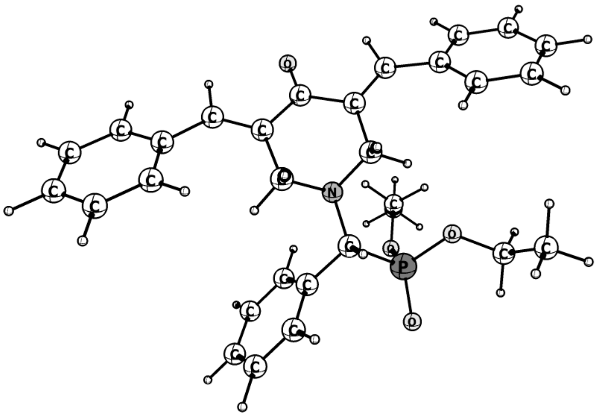	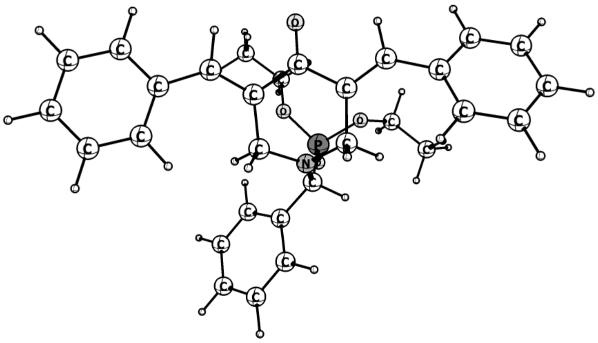
A31	A32
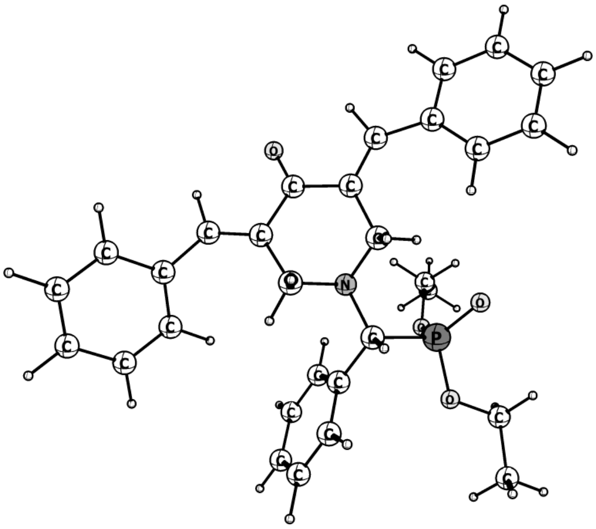	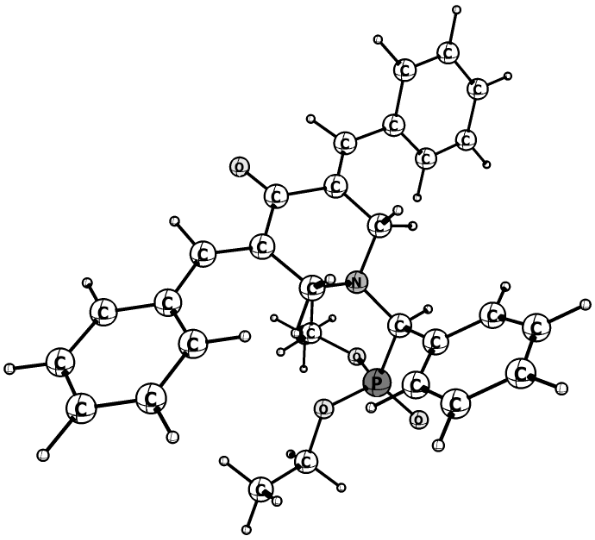
A33	A34
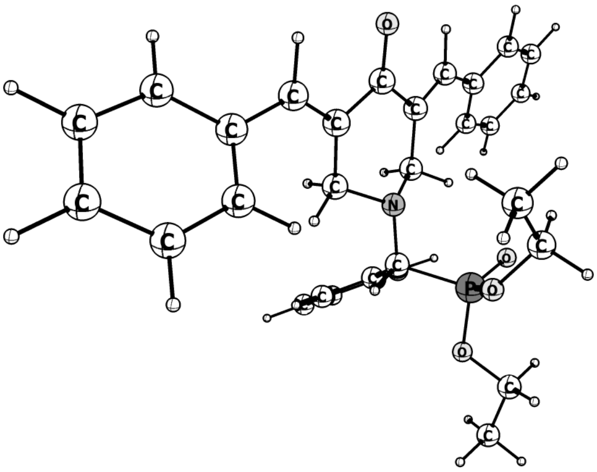	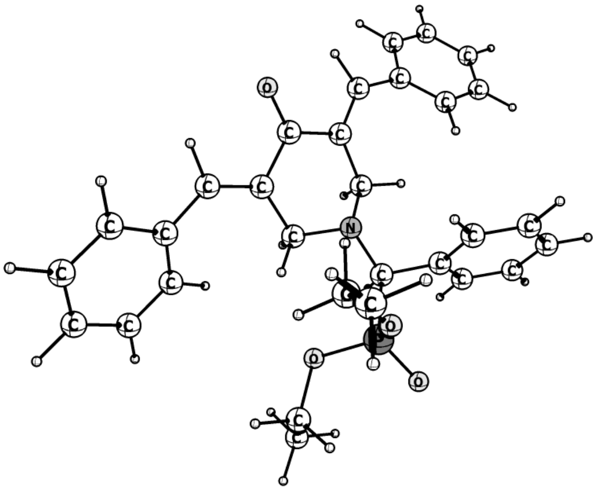
A35	A36

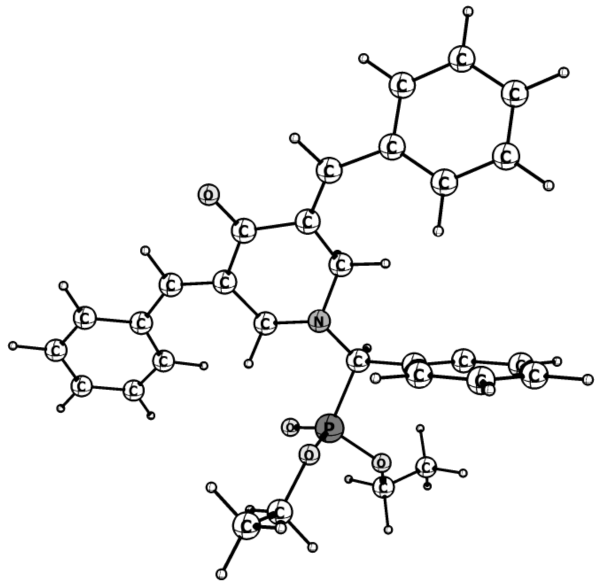	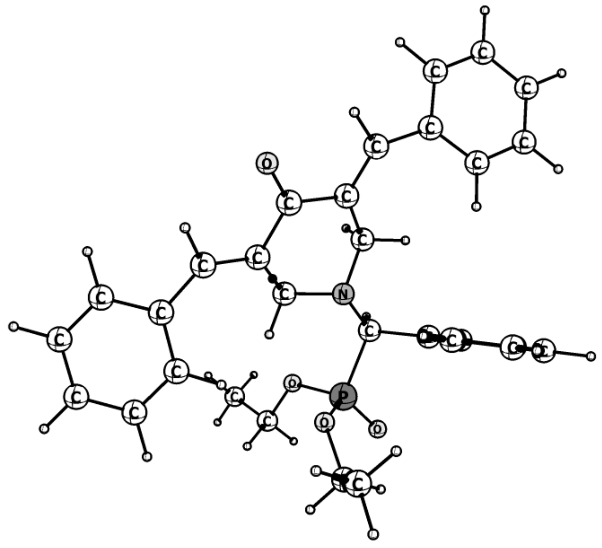
A37	A38
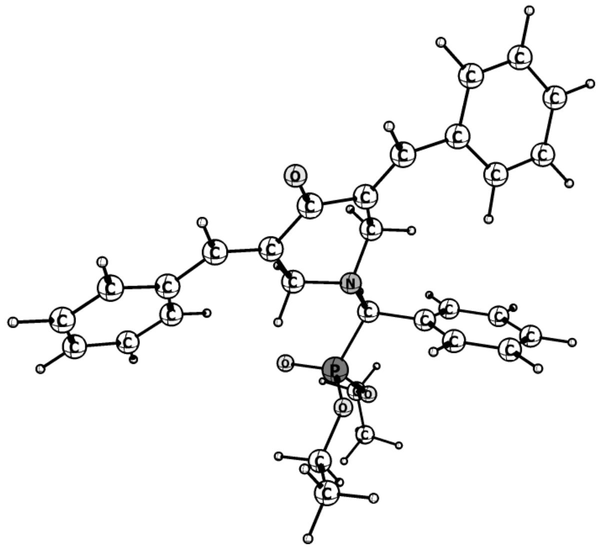	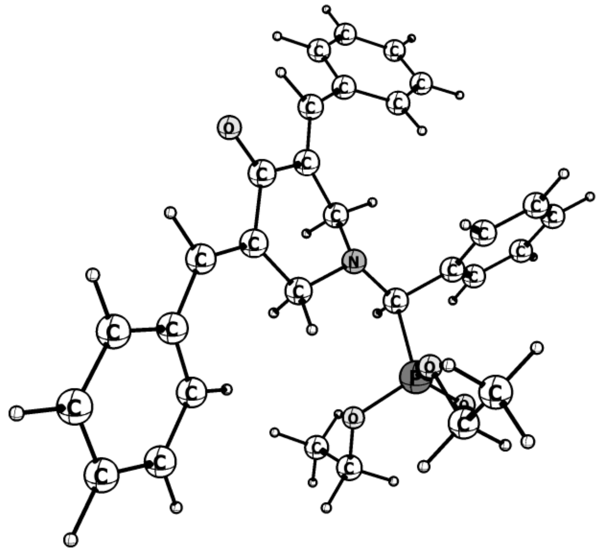
A39	A40
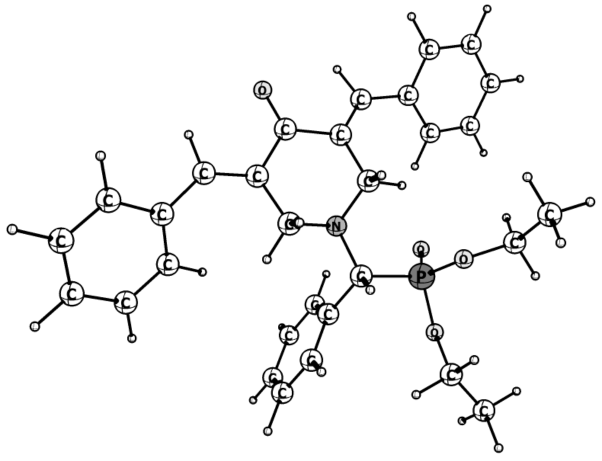	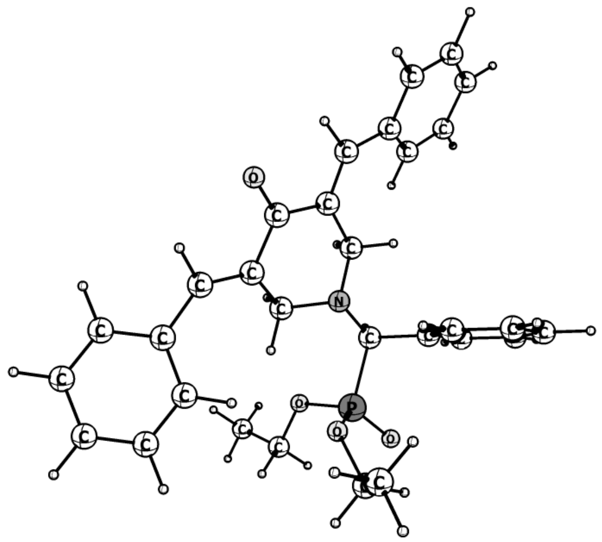
A41	A42

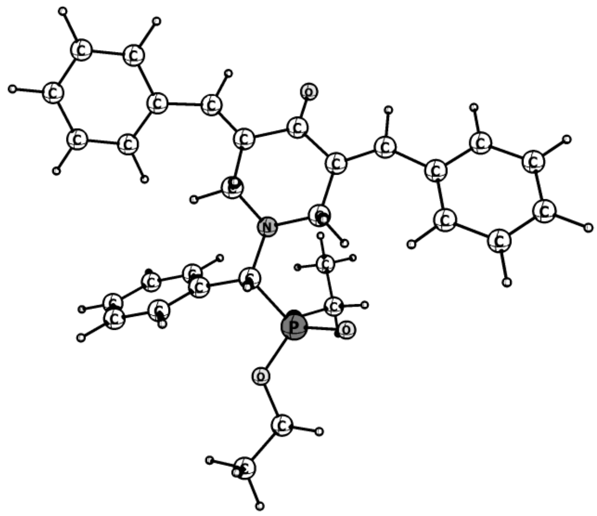	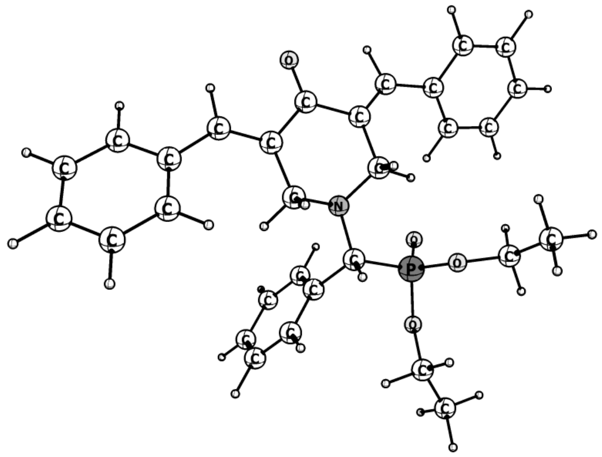
A43	A44
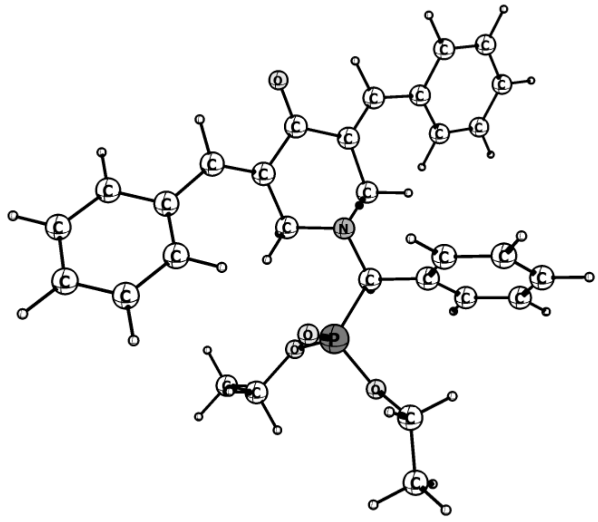	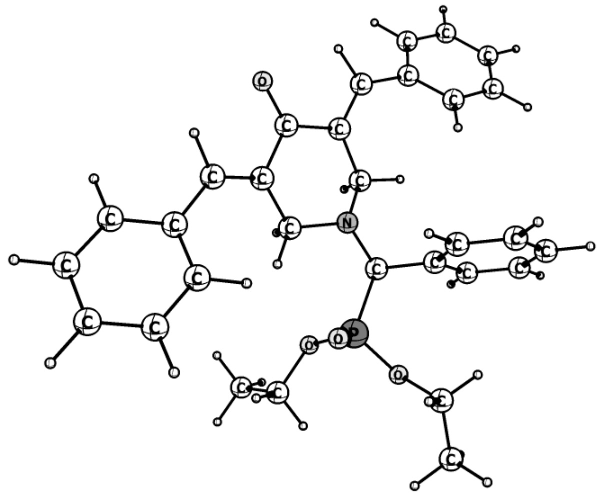
A45	B1
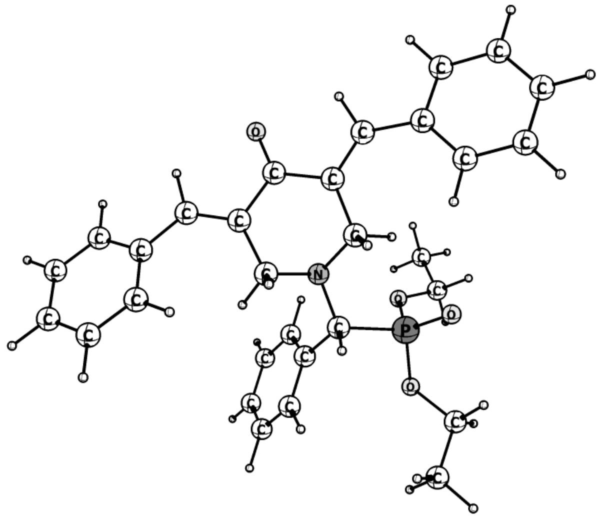	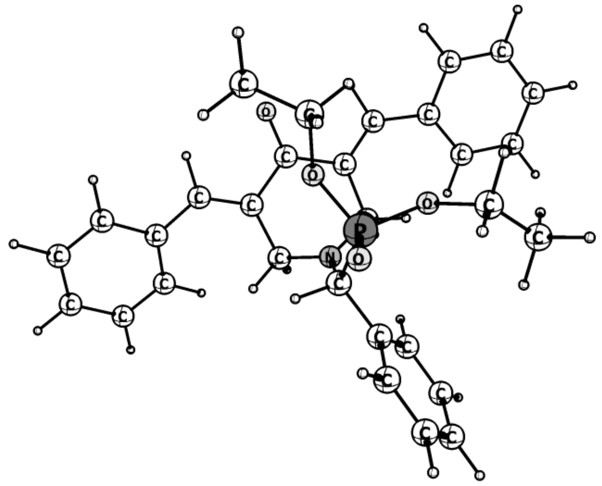
B2	B3

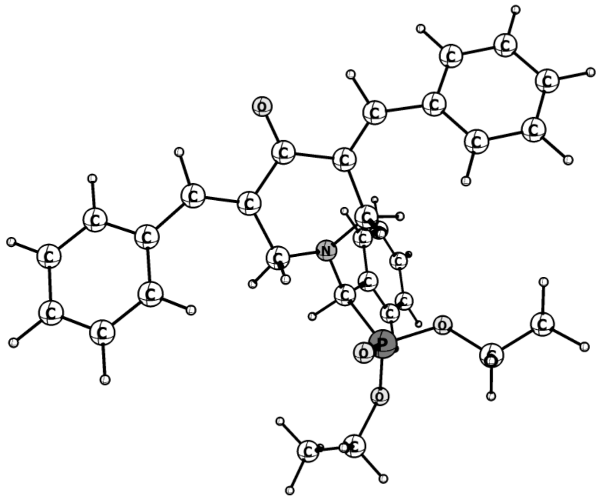	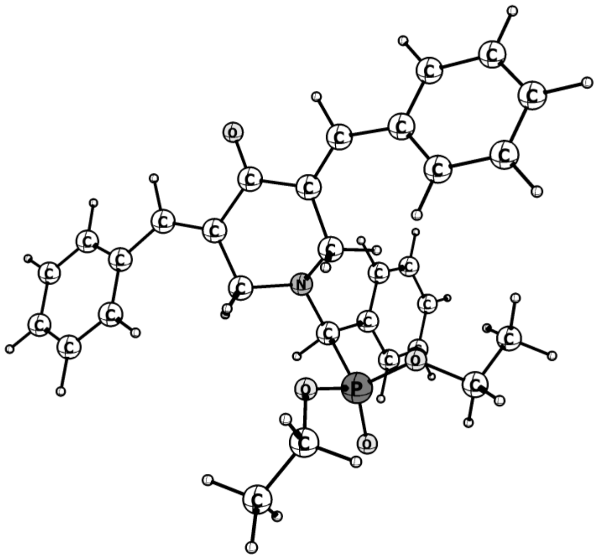
B4	B5
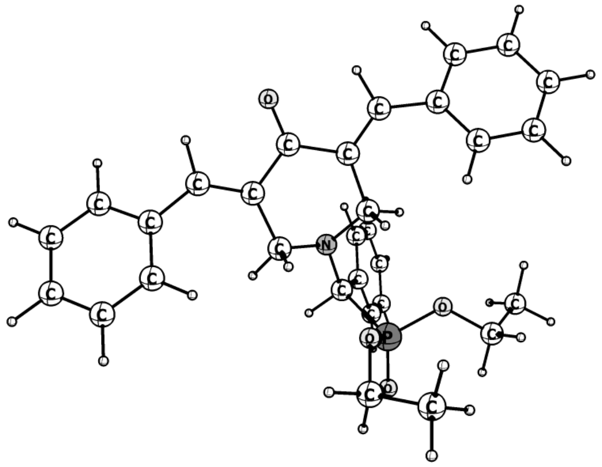	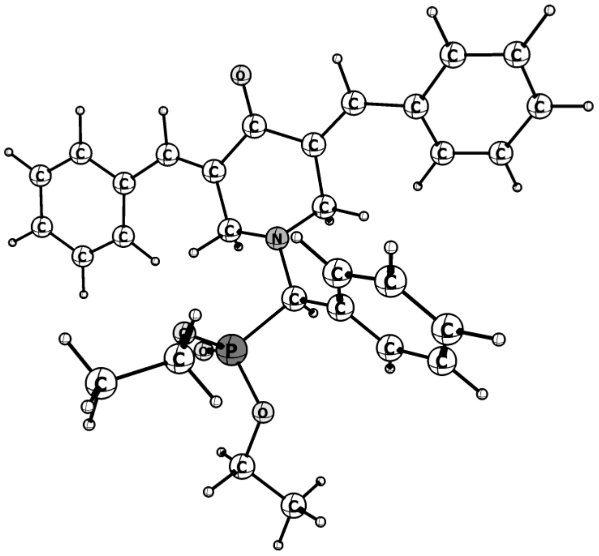
B6	B7
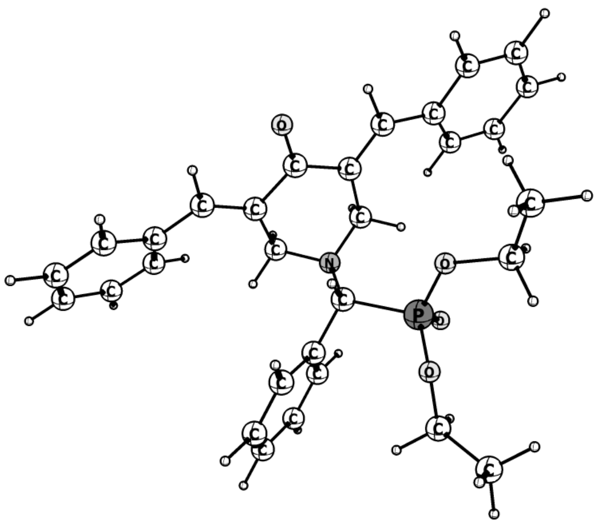	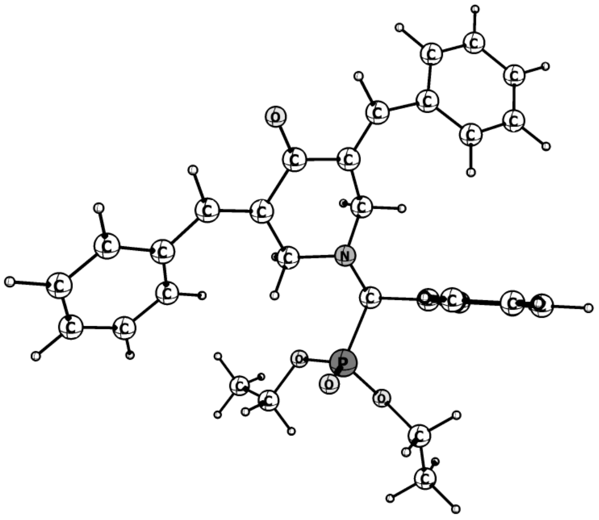
B8	B9

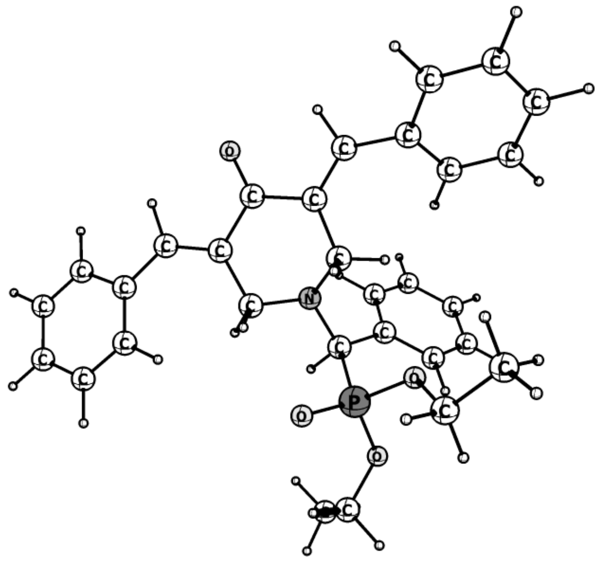	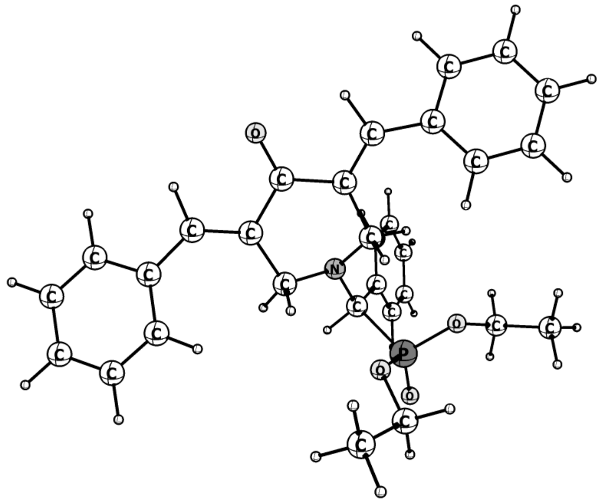
B10	B11
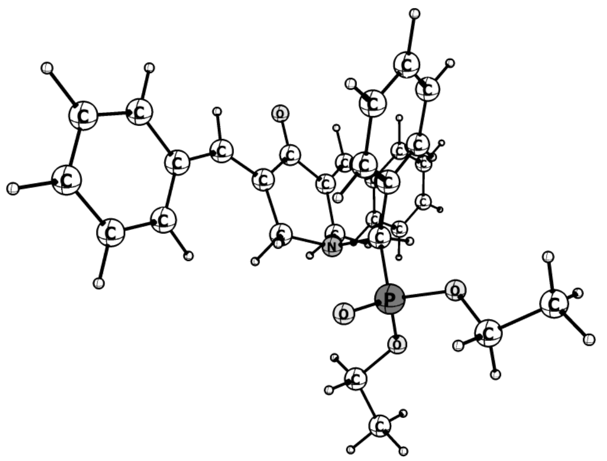	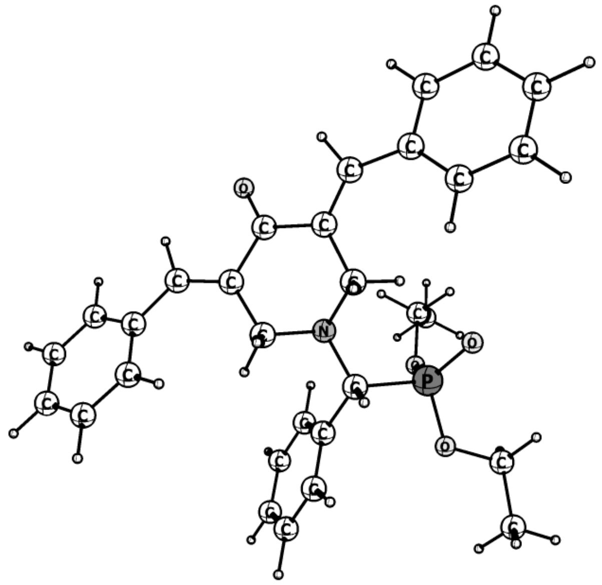
B12	B13
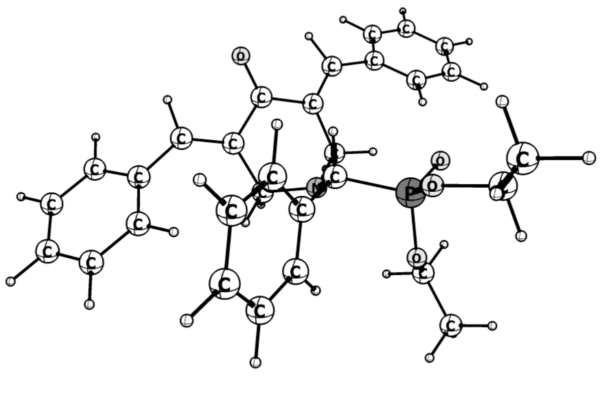	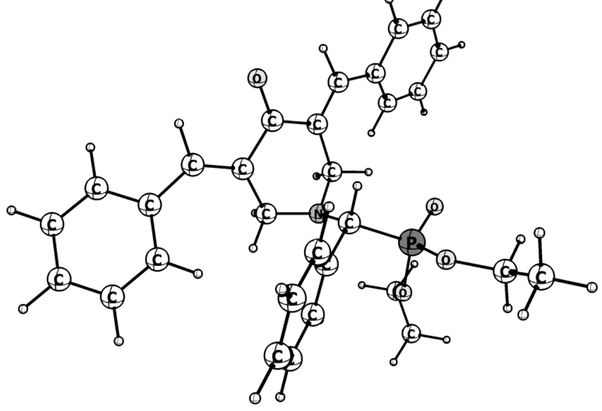
B14	B15

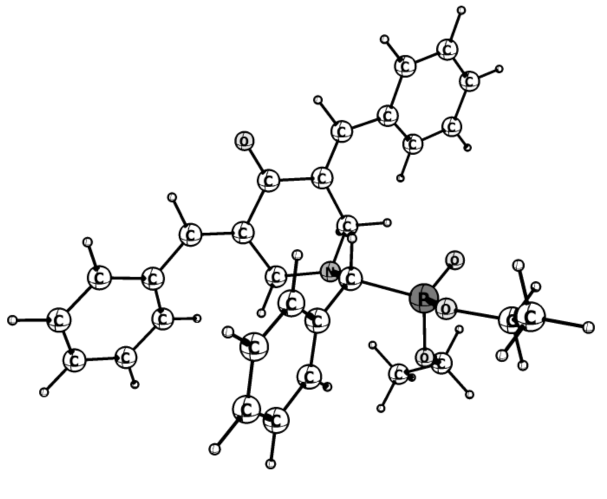	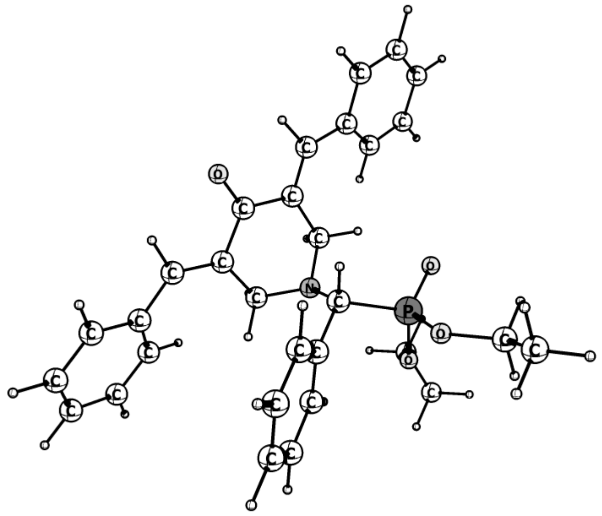
B16	B17
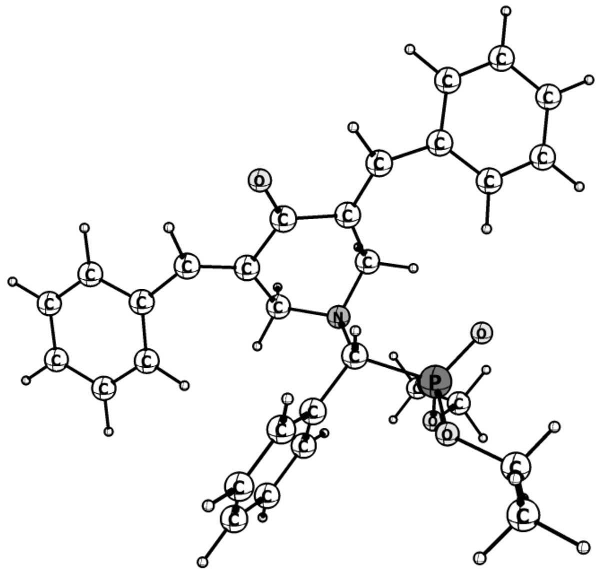	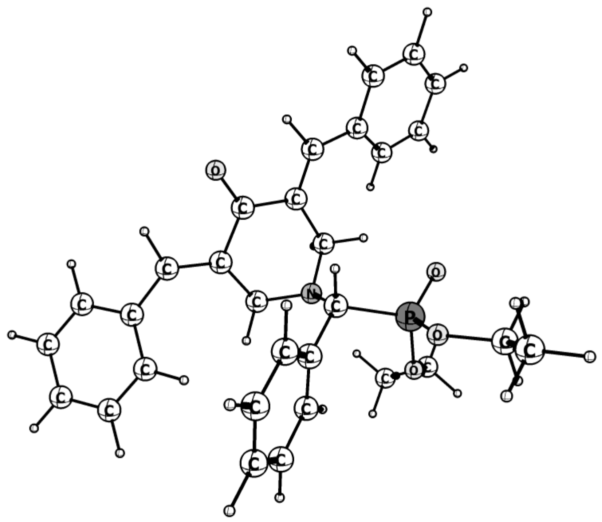

Table S4. Spatial structure of the conformers of *compound 2*, determined from quantum chemical calculations

A1	A2
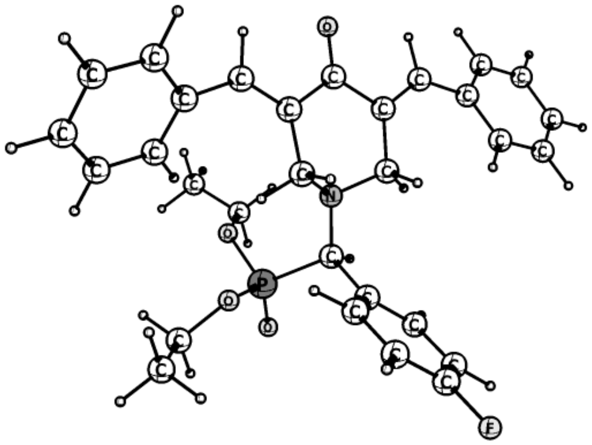	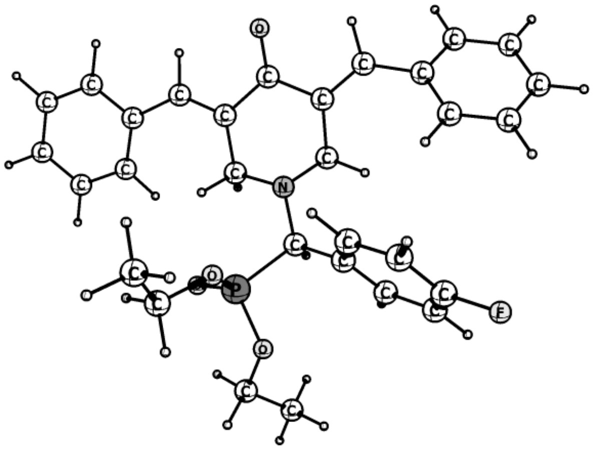
A3	A4

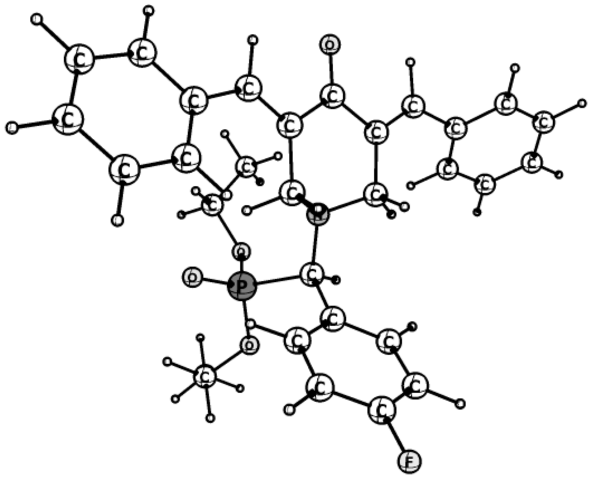	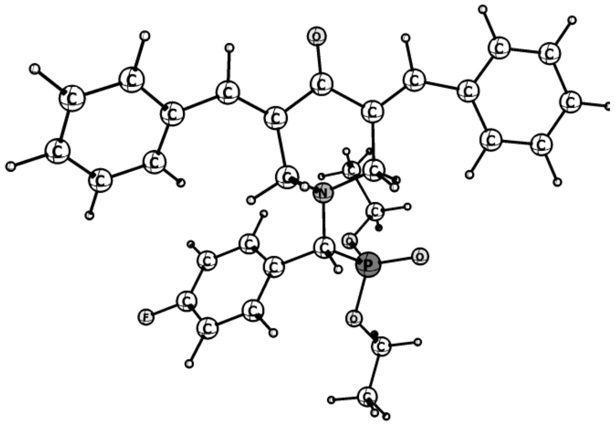
A5	A6
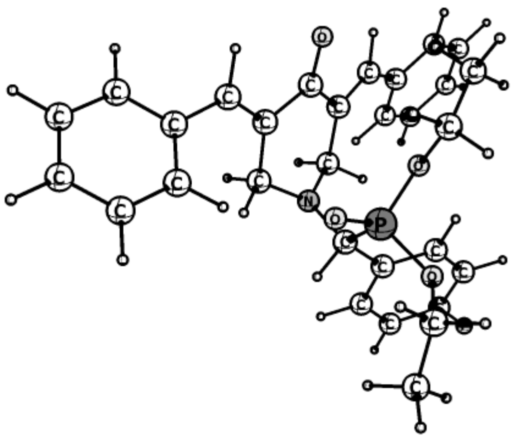	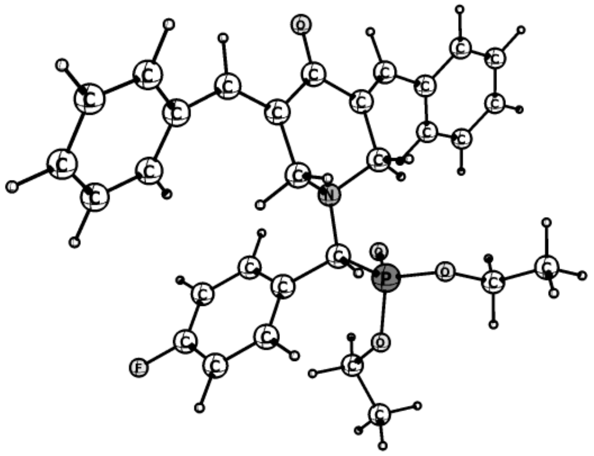
A7	A8
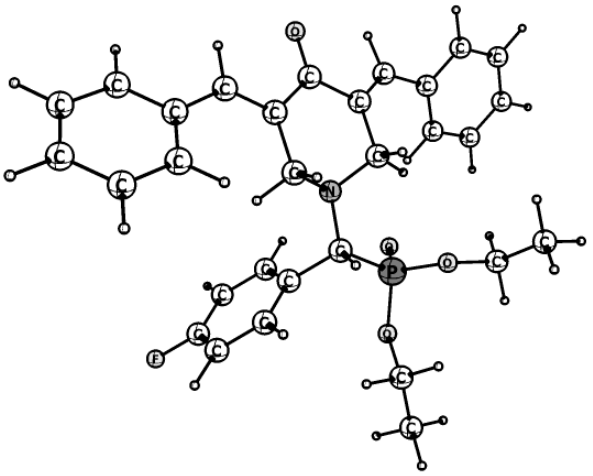	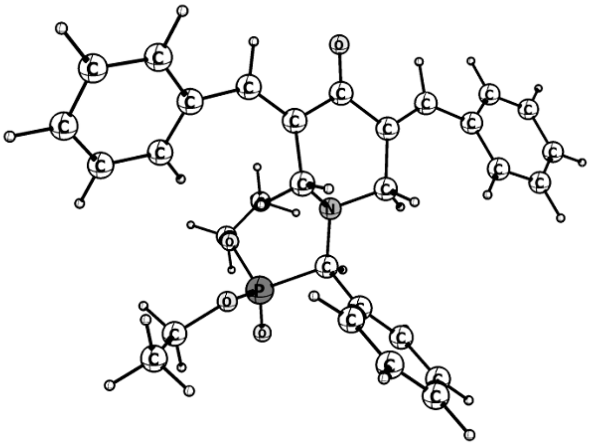
A9	A10

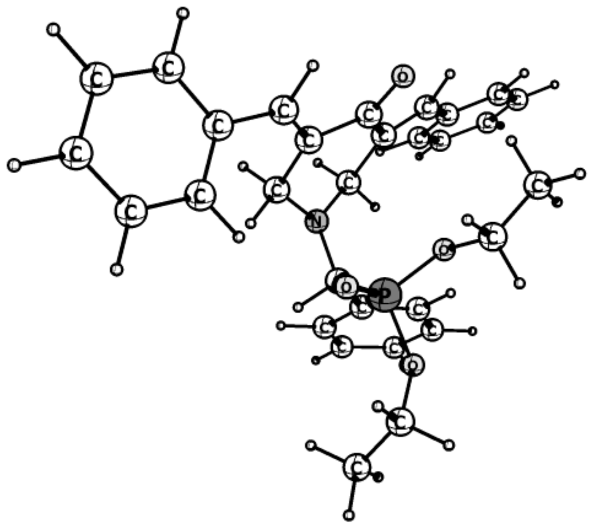

A11

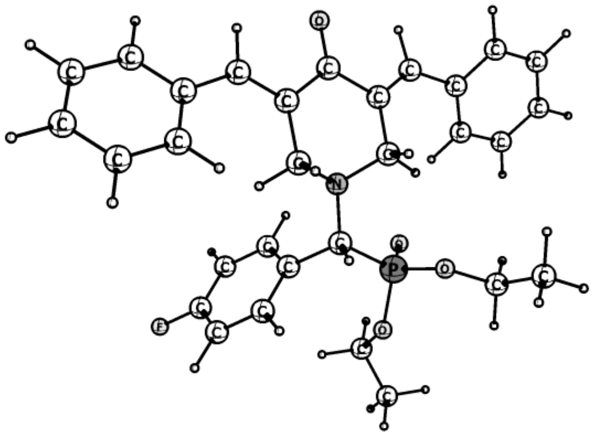

A12

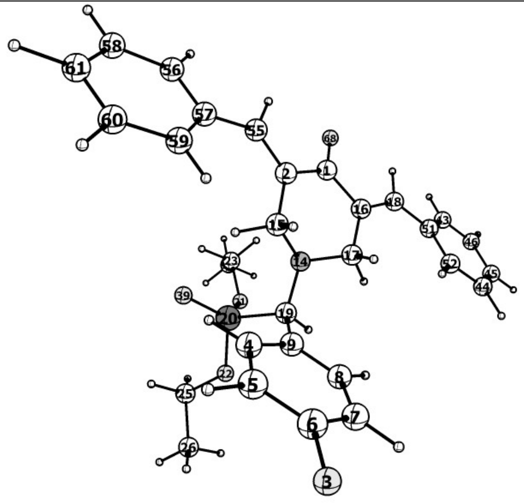

A13

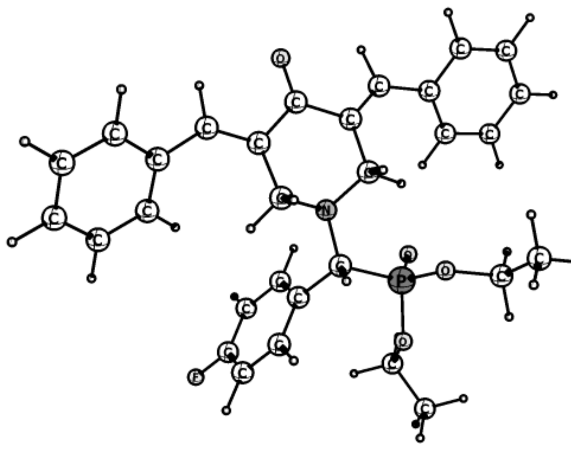

A14

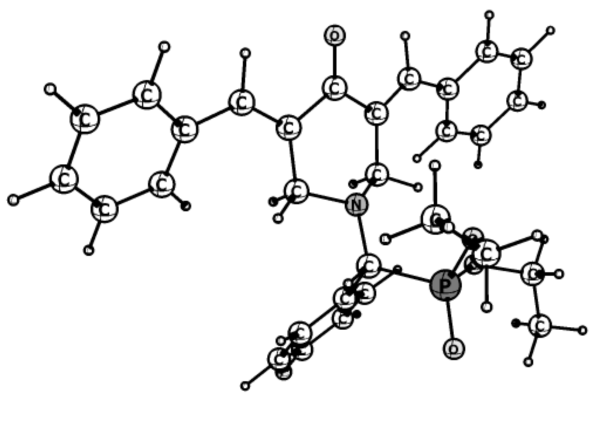

A15

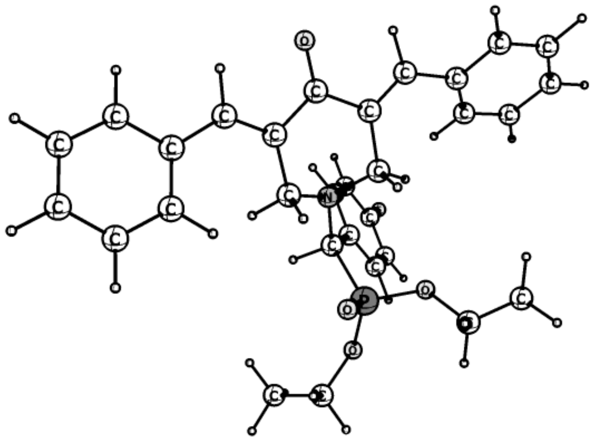

A16

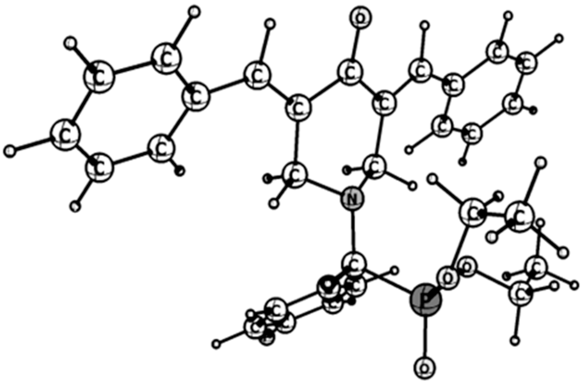	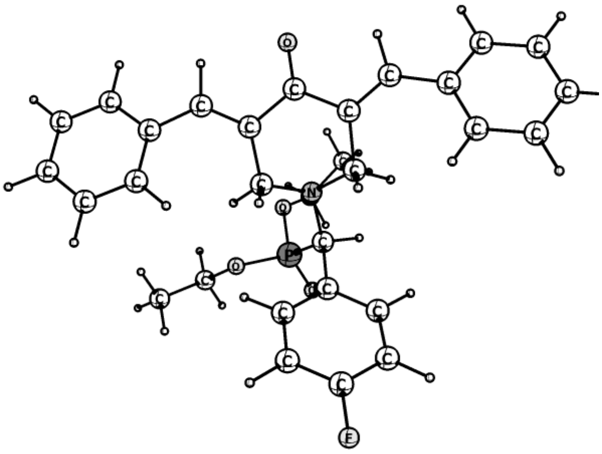
A17	A18
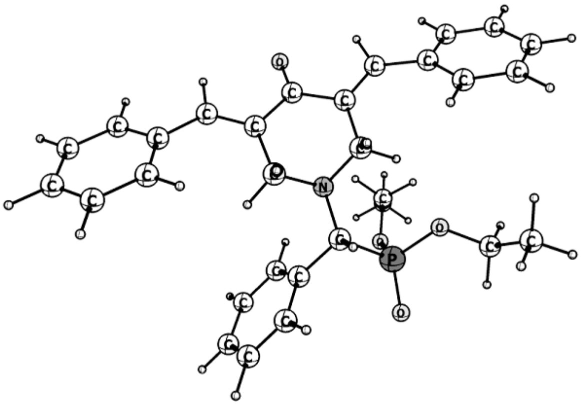	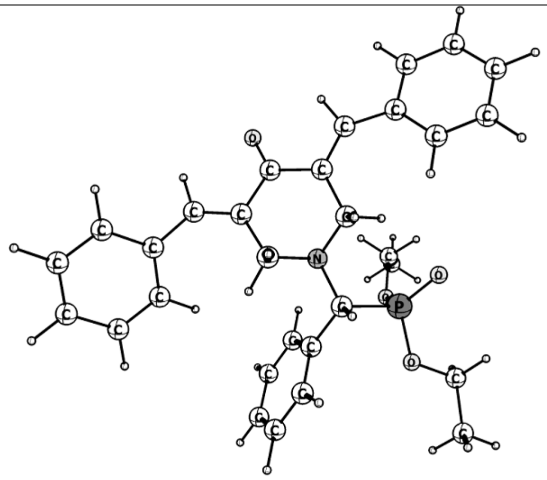
A19	A20
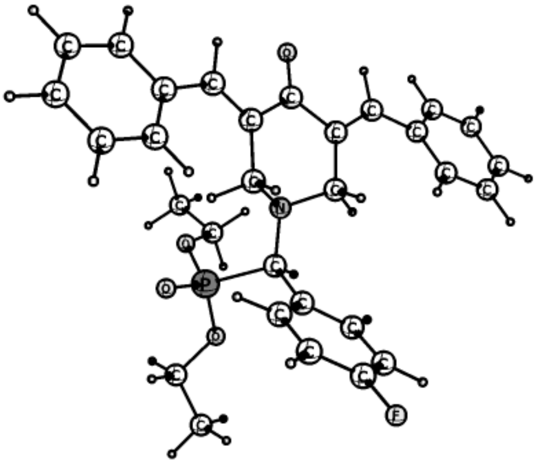	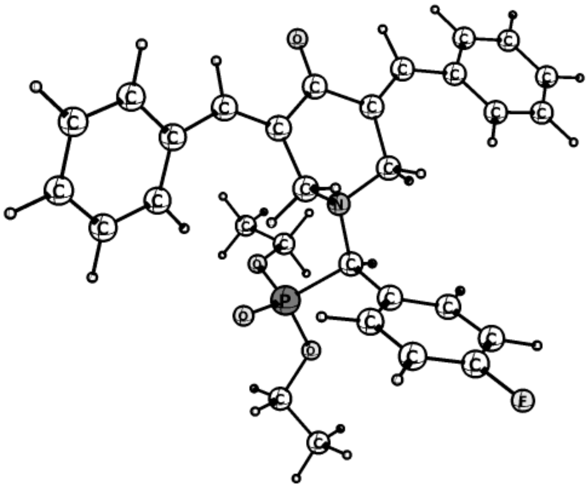
A21	A22

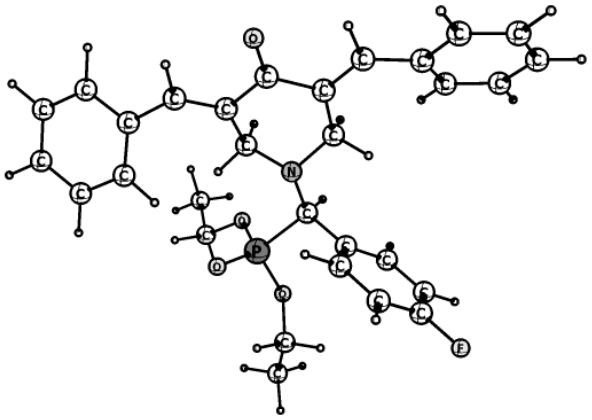	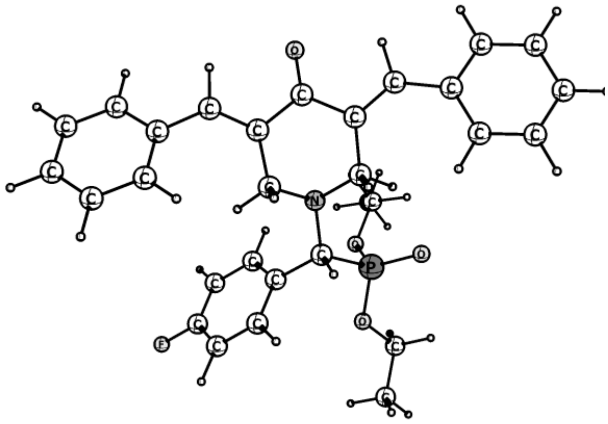
B1	B2
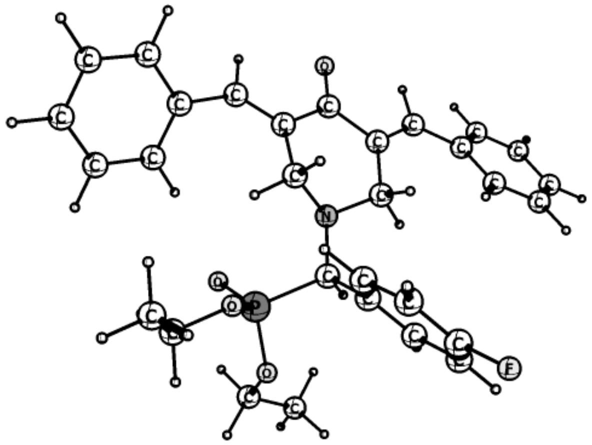	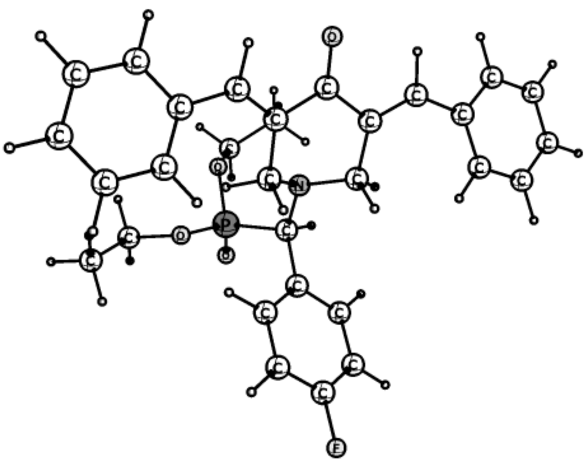
B3	B4
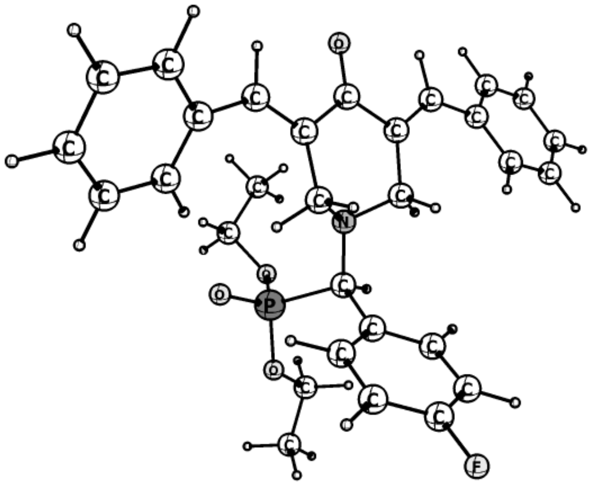	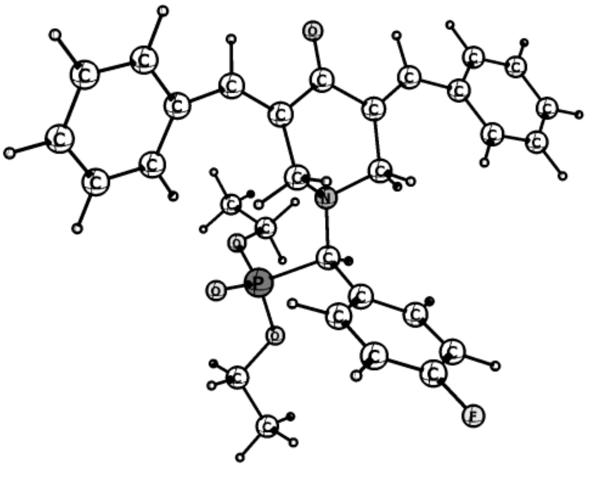
B5	B6

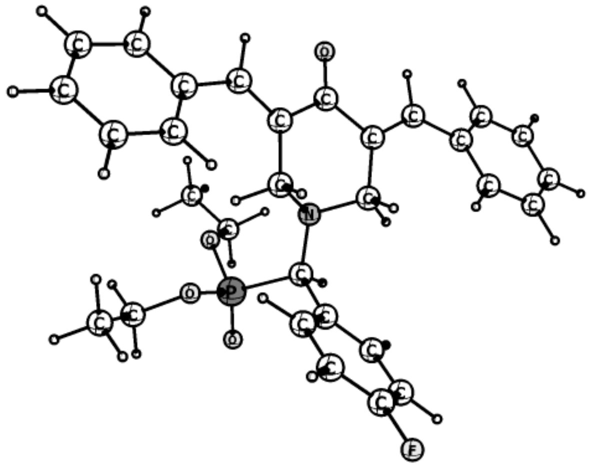	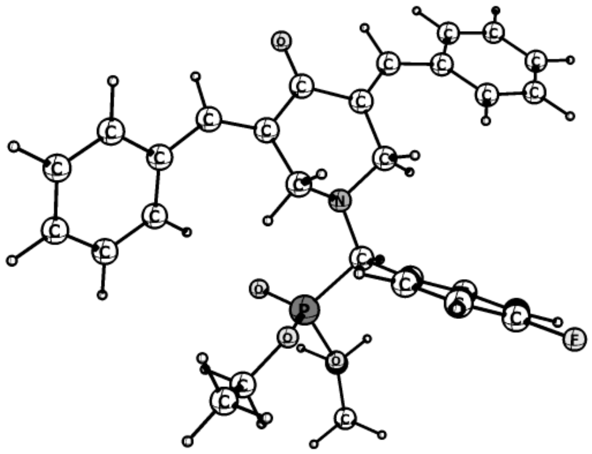
B7	B8
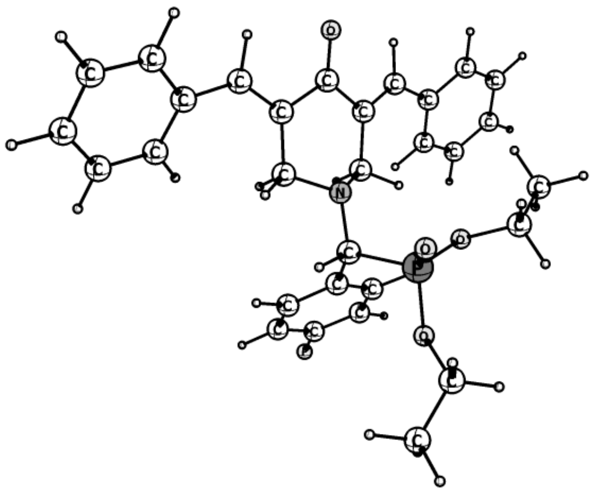	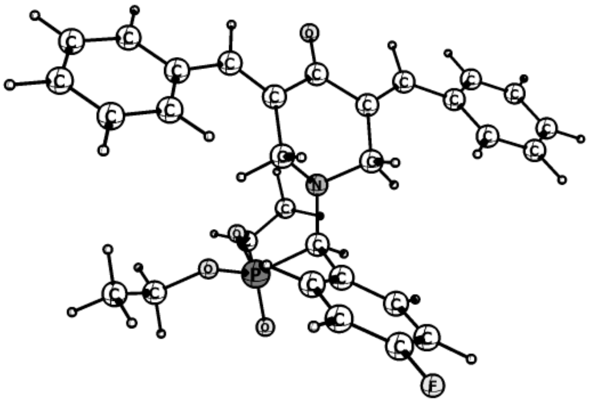
B9	B10
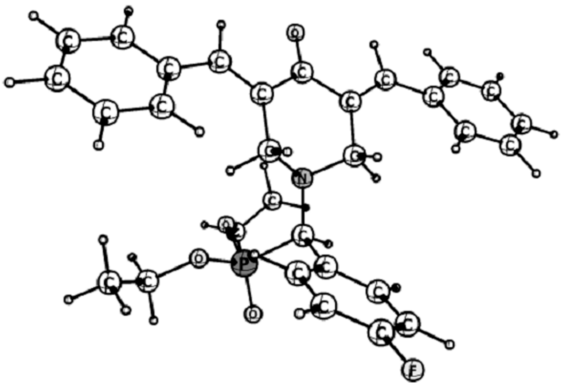	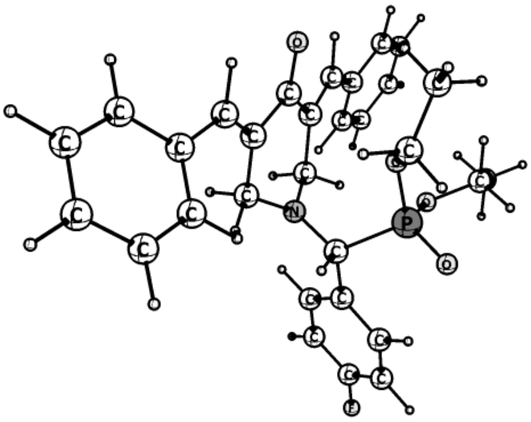
B11	B12

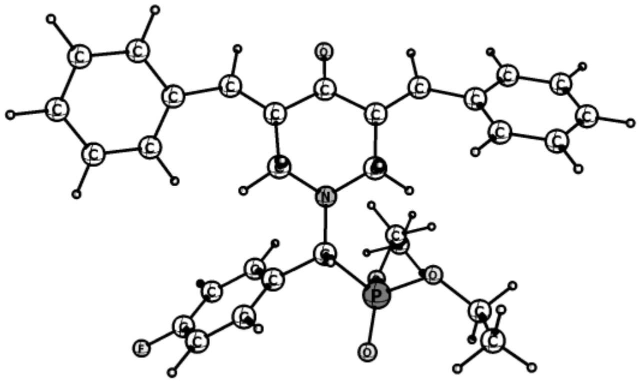	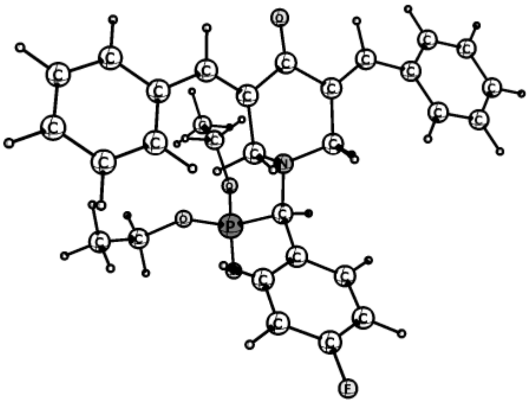
B13	B14
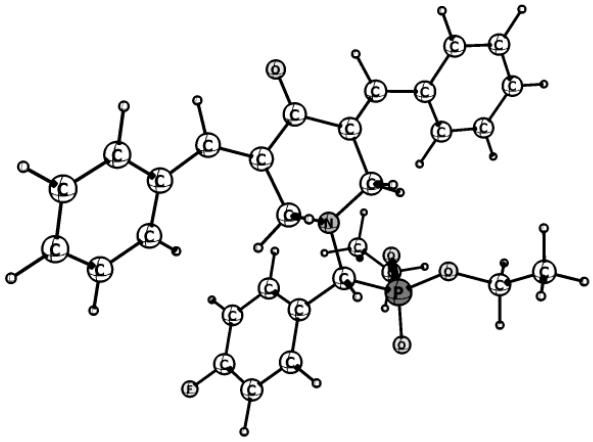	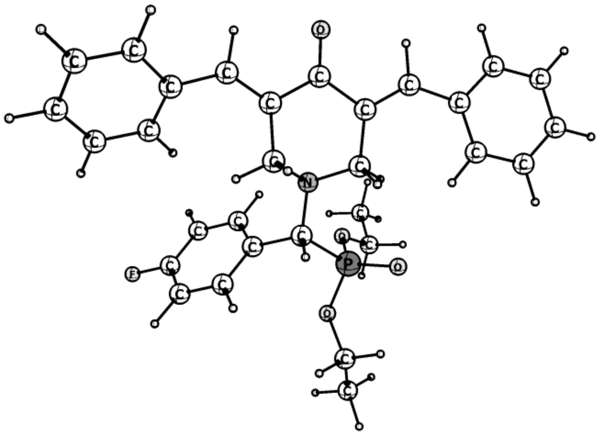
B15	B16
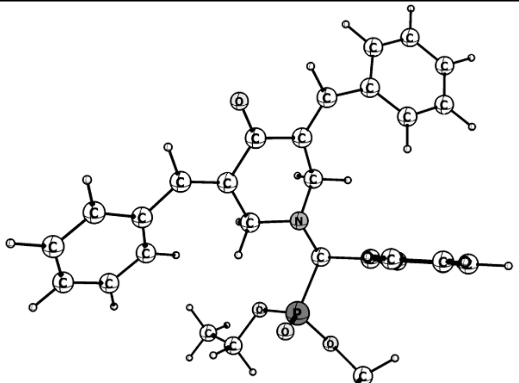	
B17	
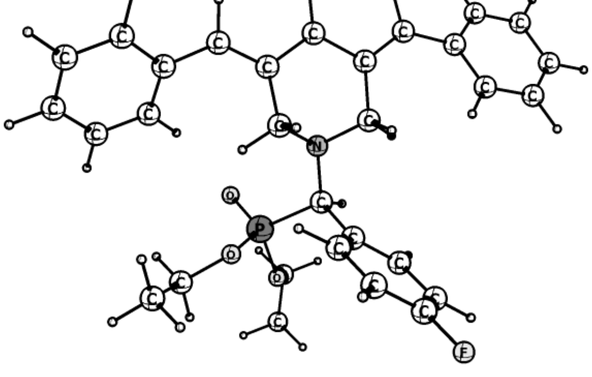	

Table S5. Values of dihedral angles, energies and intramolecular distances of the compound 1 molecule in the considered conformers corresponding to typical types of intramolecular motion

Conformer	Values of the dihedral angle, (°)		E, kJ/mol	r (H2-H14), Å	r (H6-H9/13), Å
	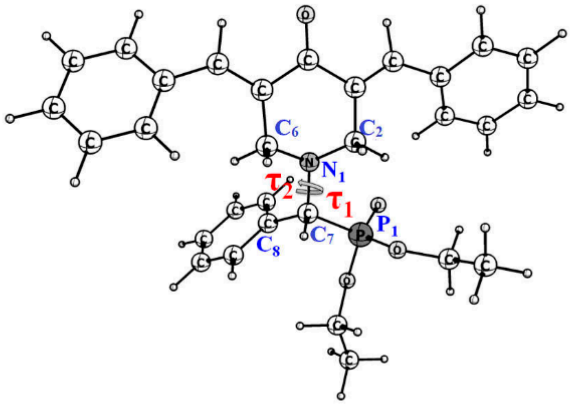				
	τ_1 (C_6-N_1-C_7-C_8)	τ_2 (C_2-N_1-C_7-P_1)			
A1	-57.6	-156.5	7.38	3.85	2.80
A2	-69.9	-164.0	21.45	3.85	2.76
A3	-71.3	-164.9	25.09	3.83	2.76
A4	-65.5	-158.6	19.65	3.85	2.81
A5	-75.3	-167.6	28.15	3.86	2.79
A6	-65.4	-158.2	18.23	3.85	2.81
A7	-67.8	-160.6	15.10	3.84	2.82
A8	-76.2	-168.4	26.27	3.85	2.79
A9	-76.2	-168.7	30.39	3.83	2.79
A10	-53.5	-153.4	2.04	3.85	2.87
A11	-53.9	-153.9	13.89	3.83	2.91
A12	-51.9	-151.9	9.28	3.84	3.00
A13	-62.5	-162.2	12.69	3.86	2.89
A14	-64.7	-164.1	10.31	3.84	2.97
A15	-52.8	-152.9	4.40	3.86	2.88
A16	-54.7	-154.4	2.58	3.86	2.95
A17	-43.1	-137.7	17.26	3.84	2.92
A18	-80.9	-105.8	35.99	3.85	2.85

A19	-54.1	-149.2	21.62	3.83	2.87
A20	-71.1	-99.0	25.61	3.84	2.95
A21	1.80	-99.8	31.36	3.85	3.20
A22	-78.6	-78.7	23.83	3.87	3.07
A23	74.2	76.0	0.0	3.84	3.29
A24	-43.5	-62.5	36.35	3.84	3.33
A25	75.7	78.3	14.36	3.86	3.26
A26	53.3	-58.9	11.21	3.84	3.38
A27	-43.7	-58.9	35.14	3.85	3.34
A28	53.7	-58.4	13.60	3.86	3.38
A29	51.6	-64.5	29.04	3.84	3.33
A30	-49.1	-77.0	36.03	3.85	3.40
A31	55.7	-57.5	16.93	3.85	3.45
A32	76.9	84.4	31.26	3.85	3.30
A33	77.1	79.8	11.85	3.84	3.25
A34	54.1	-62.3	36.31	3.86	3.40
A35	66.6	-42.5	17.90	3.84	3.58
A36	66.8	-41.9	16.32	3.85	3.52
A37	68.7	-41.8	24.01	3.85	3.58
A38	68.7	-43.7	34.78	3.85	3.59
A39	69.7	-41.2	25.35	3.85	3.51
A40	58.9	-53.4	18.75	3.84	3.49
A41	67.7	-43.8	27.31	3.85	3.49
A42	69.4	-42.2	11.93	3.84	3.51
A43	69.5	-42.9	19.74	3.86	3.58
A44	65.8	-42.4	11.27	3.84	3.60
A45	113.7	48.4	27.86	3.85	3.55
B1	149.9	57.6	12.95	3.82	3.87
B2	141.5	48.9	13.40	3.85	4.47

B3	143.0	51.4	14.12	3.84	4.53
B4	-164.7	-163.2	21.94	3.87	4.50
B5	-166.4	-176.9	14.93	3.87	4.46
B6	-164.6	-168.2	18.23	3.86	4.37
B7	154.7	65.0	20.66	3.85	4.43
B8	142.0	49.4	10.48	3.86	4.26
B9	91.9	82.4	14.17	3.82	4.52
B10	174.9	176.2	9.08	3.85	4.07
B11	165.3	163.0	25.47	3.86	4.70
B12	163.5	160.5	25.94	3.84	4.89
B13	159.4	157.0	20.49	3.83	4.90
B14	160.3	157.2	18.81	3.84	4.94
B15	167.3	165.7	27.18	3.87	4.93
B16	161.6	159.4	27.60	3.83	4.86
B17	69.4	-42.3	32.20	3.86	4.92

Table S6. Values of dihedral angles, energies and intramolecular distances of the *compound 2* molecule in the considered conformers corresponding to typical types of intramolecular motion

Conformer	Values of the dihedral angle, (°) 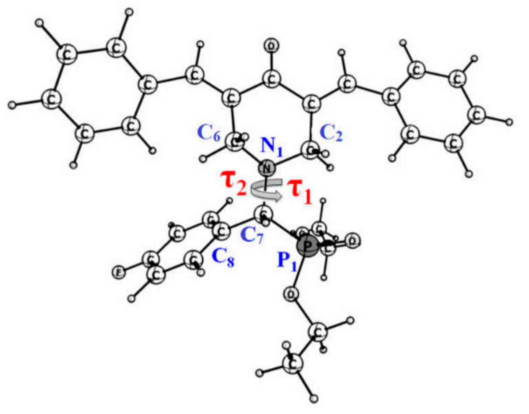		E, kJ/mol	r (H2-H14), Å	r (H6-H9/13), Å
	τ_1 (C_6-N_1-C_7-C_8)	τ_2 (C_2-N_1-C_7-P_1)			
A1	-67.7	-161.8	21.50	3.83	3.27
A2	67.5	-41.6	17.98	3.81	3.61
A3	75.1	77.4	14.58	3.84	3.55

A4	60.2	-51.8	18.58	3.82	3.40
A5	165.3	94.4	36.02	3.84	3.55
A6	70.5	-41.6	20.08	3.84	3.45
A7	69.8	-41.4	27.50	3.84	3.66
A8	-69.4	-163.1	25.35	3.83	3.36
A9	-42.6	-57.7	36.02	3.84	3.44
A10	70	-41.4	12.06	3.84	3.37
A11	-54.1	-153.6	0.0	3.84	3.37
A12	70.5	-41.6	20.08	3.84	2.98
A13	-62.4	-155.6	19.85	3.84	3.52
A14	153.8	63.8	20.33	3.86	3.44
A15	-53.2	-148.2	22.05	3.84	3.29
A16	-62.9	-155.8	18.25	3.84	3.49
A17	52.1	-64.0	28.33	3.84	3.52
A18	55.5	-57.9	17.07	3.83	3.34
A19	-62.079	-162.164	12.77	3.83	3.44
A20	-62.094	-162.167	12.78	3.83	3.29
A21	70	-41.4	12.06	3.84	3.85
A22	51.2	-61.8	9.19	3.84	3.36
B1	-75.9	-168.3	28.26	3.83	4.48
B2	-62.9	-155.8	18.25	3.84	4.50
B3	-53.1	-153.4	14.14	3.83	4.54
B4	-65.8	-165.1	10.25	3.85	4.48
B5	-66.7	-159.4	15.16	3.84	4.46
B6	-75.4	-167.7	26.55	3.83	4.55
B7	-44.7	-138.0	20.73	3.84	4.62
B8	-62.9	-155.8	18.25	3.84	4.50
B9	-65.8	-165.1	10.25	3.85	4.55
B10	-77.8	-103.4	35.36	3.86	4.51

B11	54.7	-61.7	36.25	3.84	4.72
B12	-53.2	-148.2	22.06	3.84	4.50
B13	69.6	-42.3	32.86	3.84	4.72
B14	67.5	-41.4	16.72	3.81	4.67
B15	-165.4	-168.9	18.86	3.85	4.51
B16	69.0	-41.6	23.85	3.82	4.71
B17	-42.6	-57.7	36.02	3.84	4.71

Table S7. Normalized integral intensities of the cross-peaks, cross-relaxation rates and inter-proton distances in the *compound 1* molecule used to calculate the conformer populations in $CDCl_3$

Groups	r_{calc}, Å	Dist.	Mixing Time	Relative Integral Intensity	Cross-Relaxation Rate, s^{-1}	Error	r_{exp}, Å
A+B	3.85	H2-H14	0.15	0.0006	$3.4 \cdot 10^{-3}$	$\pm 0.13 \cdot 10^{-3}$	3.85±0.06
			0.20	0.0006			
			0.25	0.0009			
			0.30	0.0010			
			0.35	0.0011			
			0.40	0.0013			
			0.45	0.0014			
			0.50	0.0016			
			0.55	0.0020			
			0.60	0.0020			
			0.65	0.0023			
			0.70	0.0023			
			0.75	0.0025			
A	3.07	H6-H9/13	0.15	0.0018	$4.4 \cdot 10^{-3}$	$\pm 0.07 \cdot 10^{-3}$	3.67±0.06
			0.20	0.0020			
			0.25	0.0023			
			0.30	0.0024			
			0.35	0.0027			
			0.40	0.0029			
			0.45	0.0032			
B	4.48		0.50	0.0034			
			0.55	0.0036			
			0.60	0.0038			
			0.65	0.0041			
			0.70	0.0042			
			0.75	0.0044			

Table S8. Normalized integral intensities of the cross-peaks, cross-relaxation rates and inter-proton distances in the *compound 2* molecule used to calculate the conformer populations in $CDCl_3$

Groups	r_{calc}, Å	Dist.	Mixing Time	Relative Integral Intensity	Cross-Relaxation Rate, s^{-1}	Error	r_{exp}, Å
			0.15	0.0007			
			0.20	0.0009			
			0.25	0.0011			
			0.30	0.0012			
			0.35	0.0014			
			0.40	0.0016			
A+B	3.84	H2-H14	0.45	0.0017	$3.4 \cdot 10^{-3}$	$\pm 0.06 \cdot 10^{-3}$	3.84±0.06
			0.50	0.0019			
			0.55	0.0020			
			0.60	0.0023			
			0.65	0.0024			
			0.70	0.0026			
			0.75	0.0028			
			0.15	0.0022			
			0.20	0.0026			
			0.25	0.0030			
A	3.40		0.30	0.0033			
			0.35	0.0036			
			0.40	0.0037			
		H6-H9/13	0.45	0.0042	$5.8 \cdot 10^{-3}$	$\pm 0.14 \cdot 10^{-3}$	3.51±0.05
			0.50	0.0045			
			0.55	0.0046			
B	4.57		0.60	0.0051			
			0.65	0.0053			
			0.70	0.0055			
			0.75	0.0058			

Table S9. Average integral intensity of distances derived from NOESY spectral analysis for compound 1

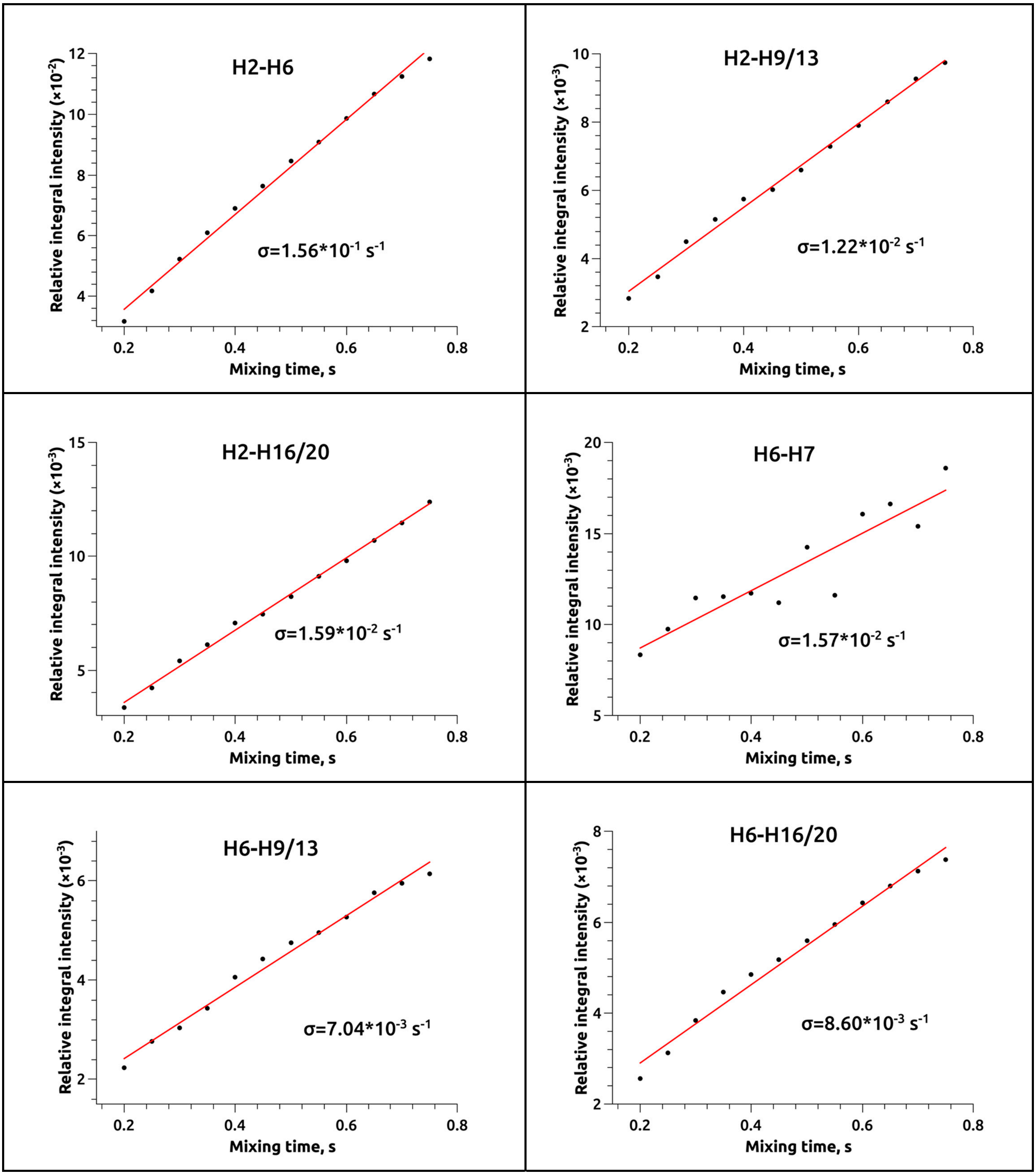

H2-H6
σ=1.56*10-1 s-1
Relative integral intensity (×10-2)
Mixing time, s
H2-H9/13
σ=1.22*10-2 s-1
Relative integral intensity (×10-3)
Mixing time, s
H2-H16/20
σ=1.59*10-2 s-1
Relative integral intensity (×10-3)
Mixing time, s
H6-H7
σ=1.57*10-2 s-1
Relative integral intensity (×10-3)
Mixing time, s
H6-H9/13
σ=7.04*10-3 s-1
Relative integral intensity (×10-3)
Mixing time, s
H6-H16/20
σ=8.60*10-3 s-1
Relative integral intensity (×10-3)
Mixing time, s

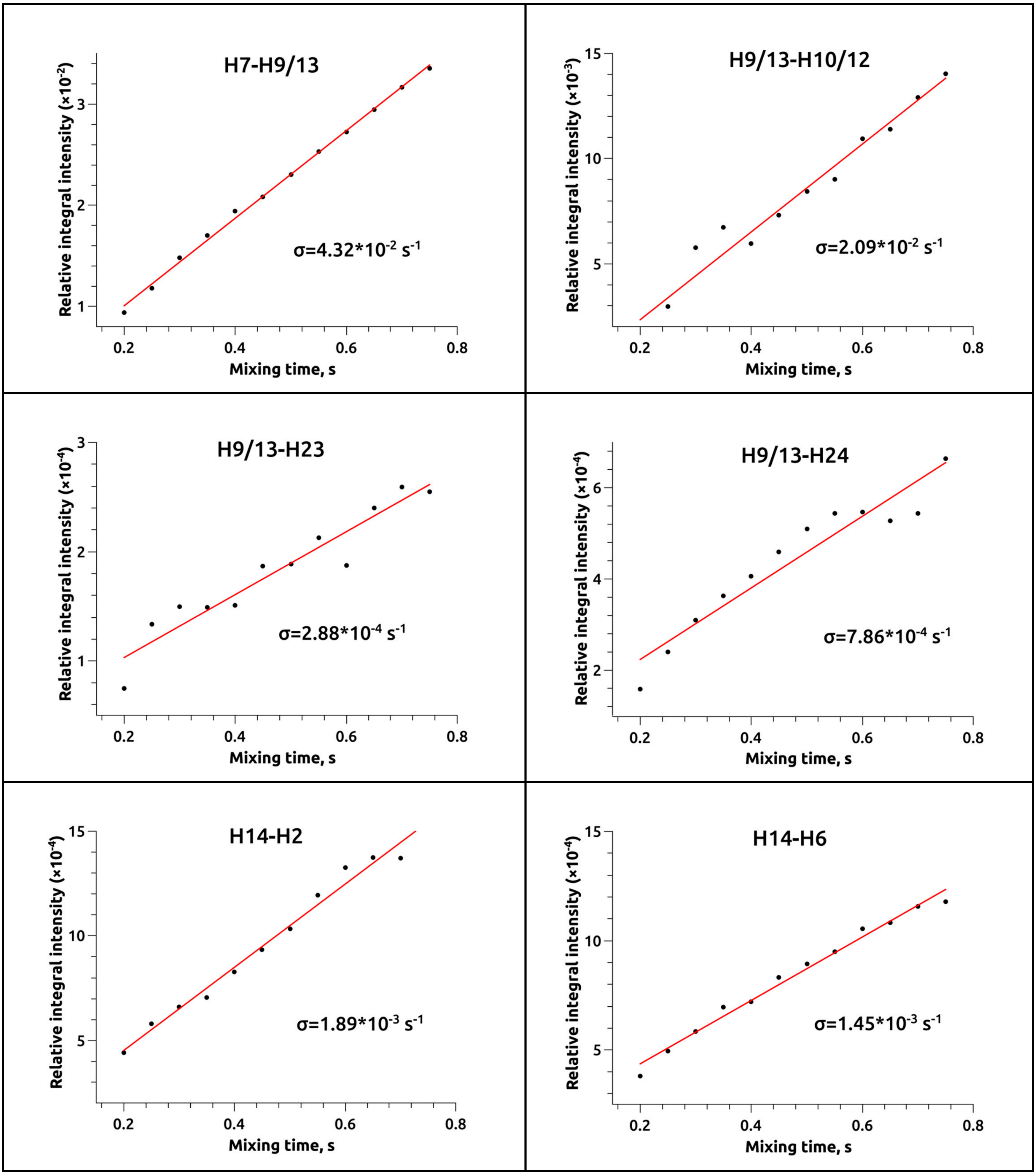

H7-H9/13
σ=4.32*10-2 s-1
H9/13-H10/12
σ=2.09*10-2 s-1
H9/13-H23
σ=2.88*10-4 s-1
H9/13-H24
σ=7.86*10-4 s-1
H14-H2
σ=1.89*10-3 s-1
H14-H6
σ=1.45*10-3 s-1
Relative integral intensity (×10-2)
Relative integral intensity (×10-3)
Relative integral intensity (×10-4)
Mixing time, s

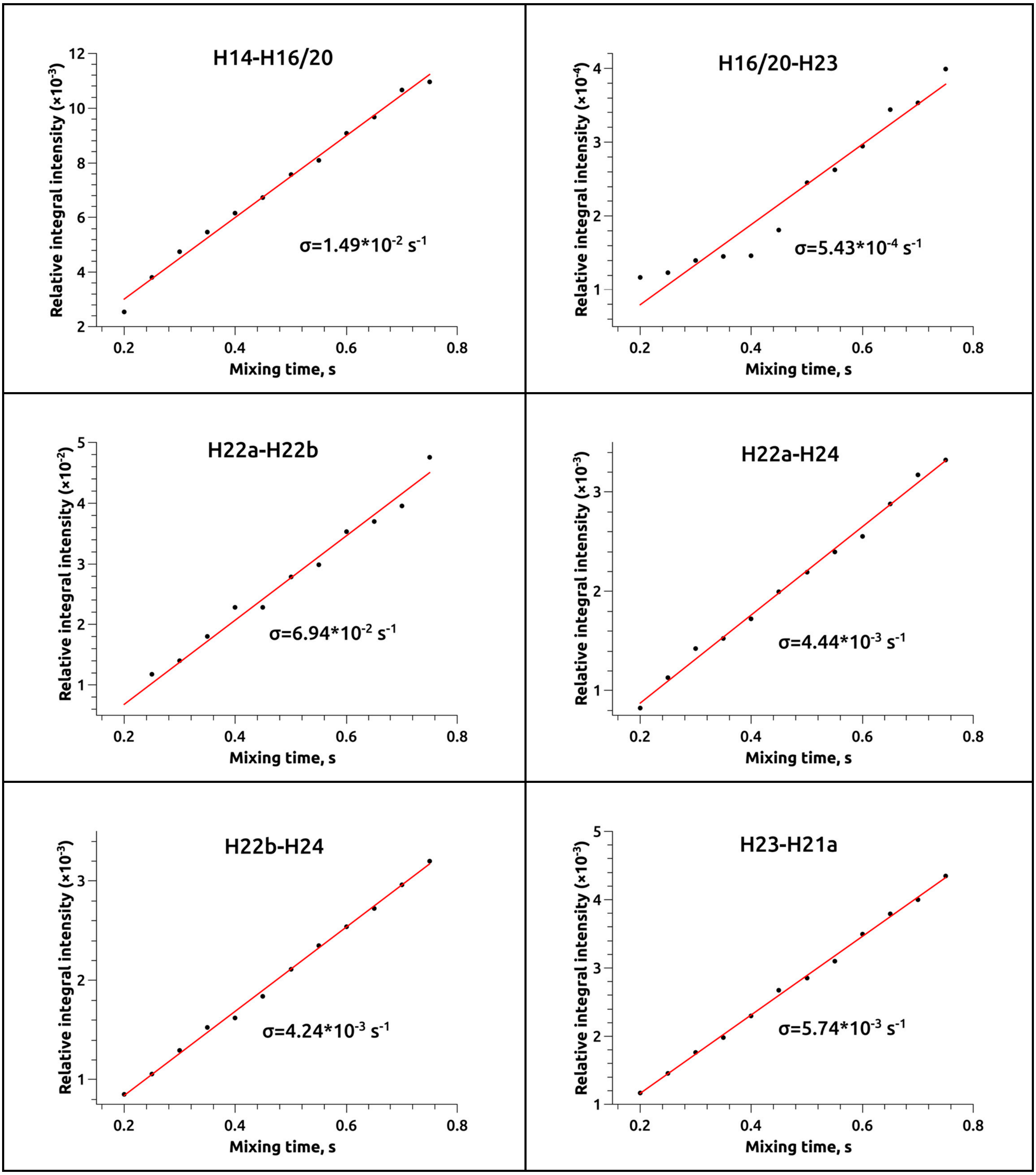

Table S10. Average integral intensity of distances derived from NOESY spectral analysis for compound 2.

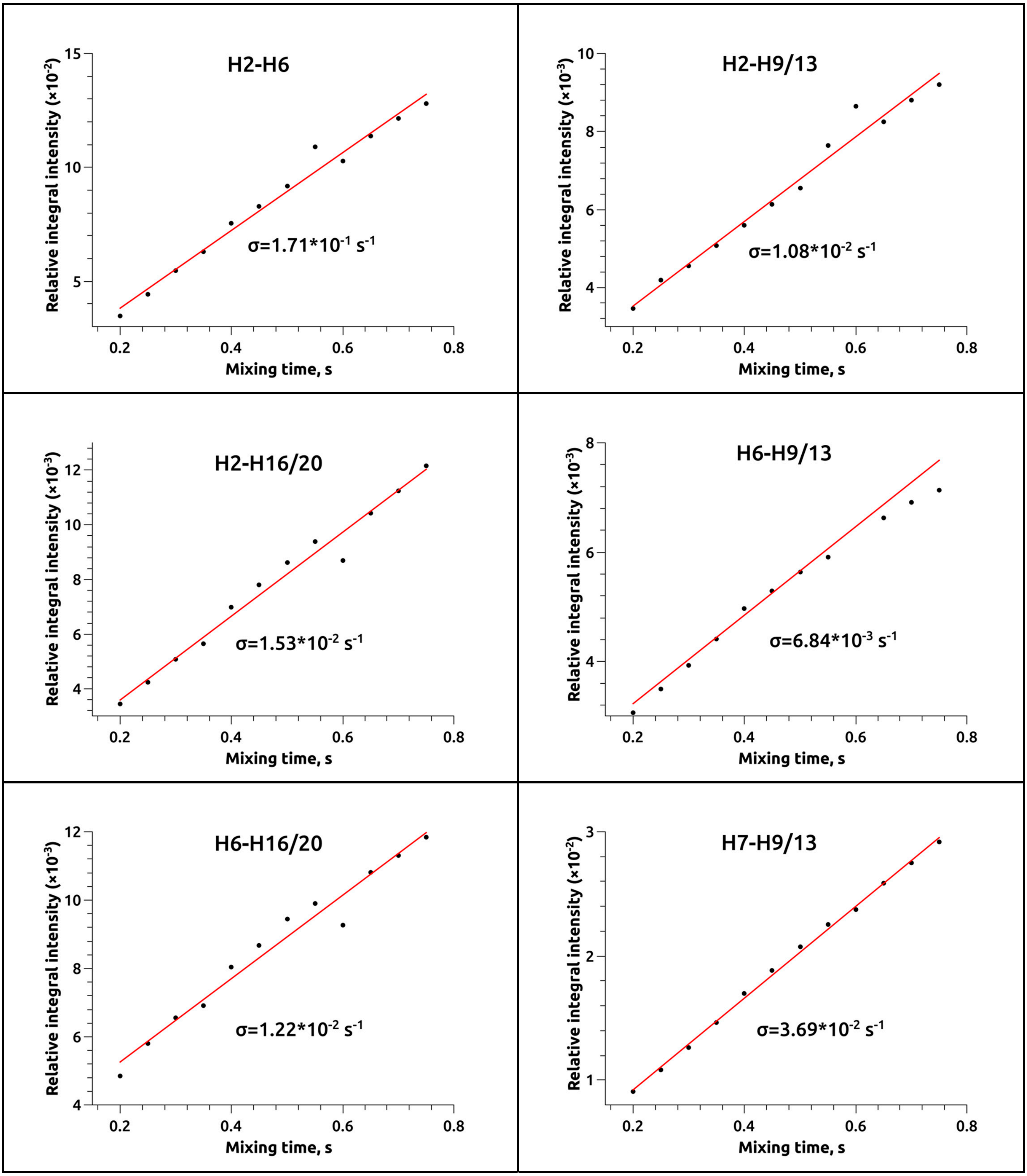
H2-H6
Relative integral intensity (×10⁻²)
σ=1.71*10⁻¹ s⁻¹
Mixing time, s
H2-H9/13
Relative integral intensity (×10⁻³)
σ=1.08*10⁻² s⁻¹
Mixing time, s
H2-H16/20
Relative integral intensity (×10⁻³)
σ=1.53*10⁻² s⁻¹
Mixing time, s
H6-H9/13
Relative integral intensity (×10⁻³)
σ=6.84*10⁻³ s⁻¹
Mixing time, s
H6-H16/20
Relative integral intensity (×10⁻³)
σ=1.22*10⁻² s⁻¹
Mixing time, s
H7-H9/13
Relative integral intensity (×10⁻²)
σ=3.69*10⁻² s⁻¹
Mixing time, s

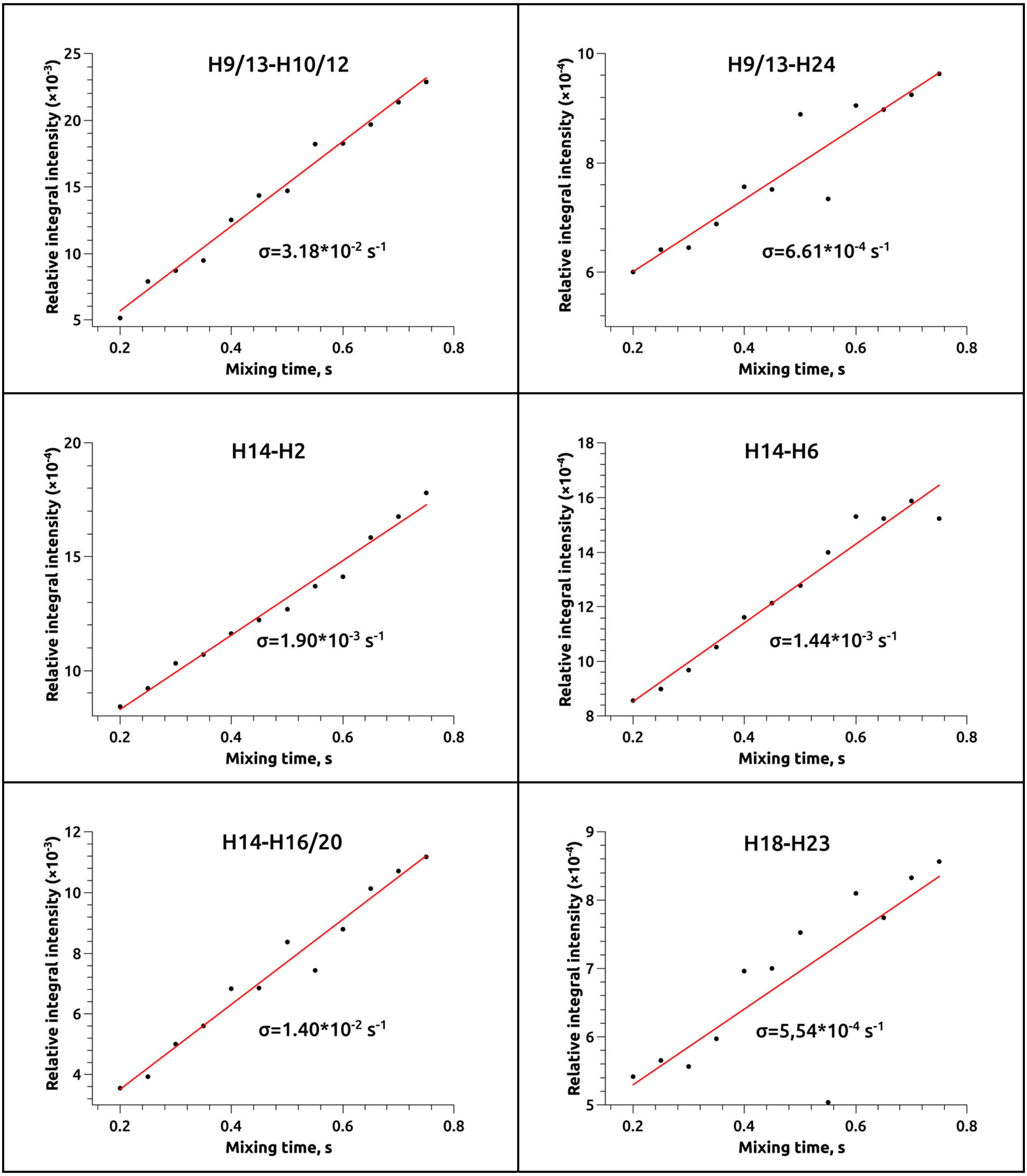
H9/13-H10/12
σ=3.18*10-2 s-1
H9/13-H24
σ=6.61*10-4 s-1
H14-H2
σ=1.90*10-3 s-1
H14-H6
σ=1.44*10-3 s-1
H14-H16/20
σ=1.40*10-2 s-1
H18-H23
σ=5,54*10-4 s-1
Relative integral intensity (×10-3)
Relative integral intensity (×10-4)
Mixing time, s

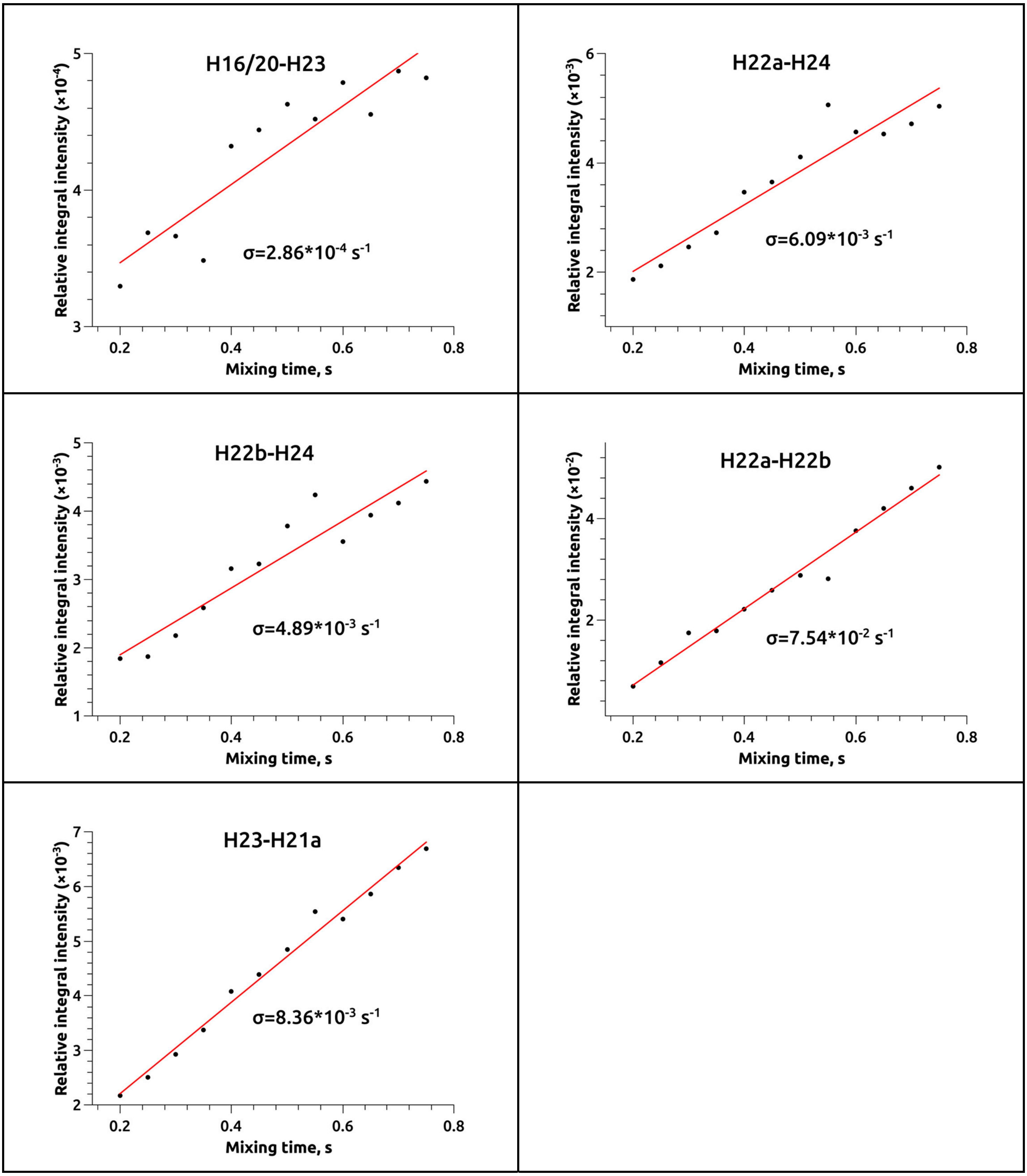

Table S11. Dependence of the cross-relaxation rate on the internuclear distance for conformers for compound 1.

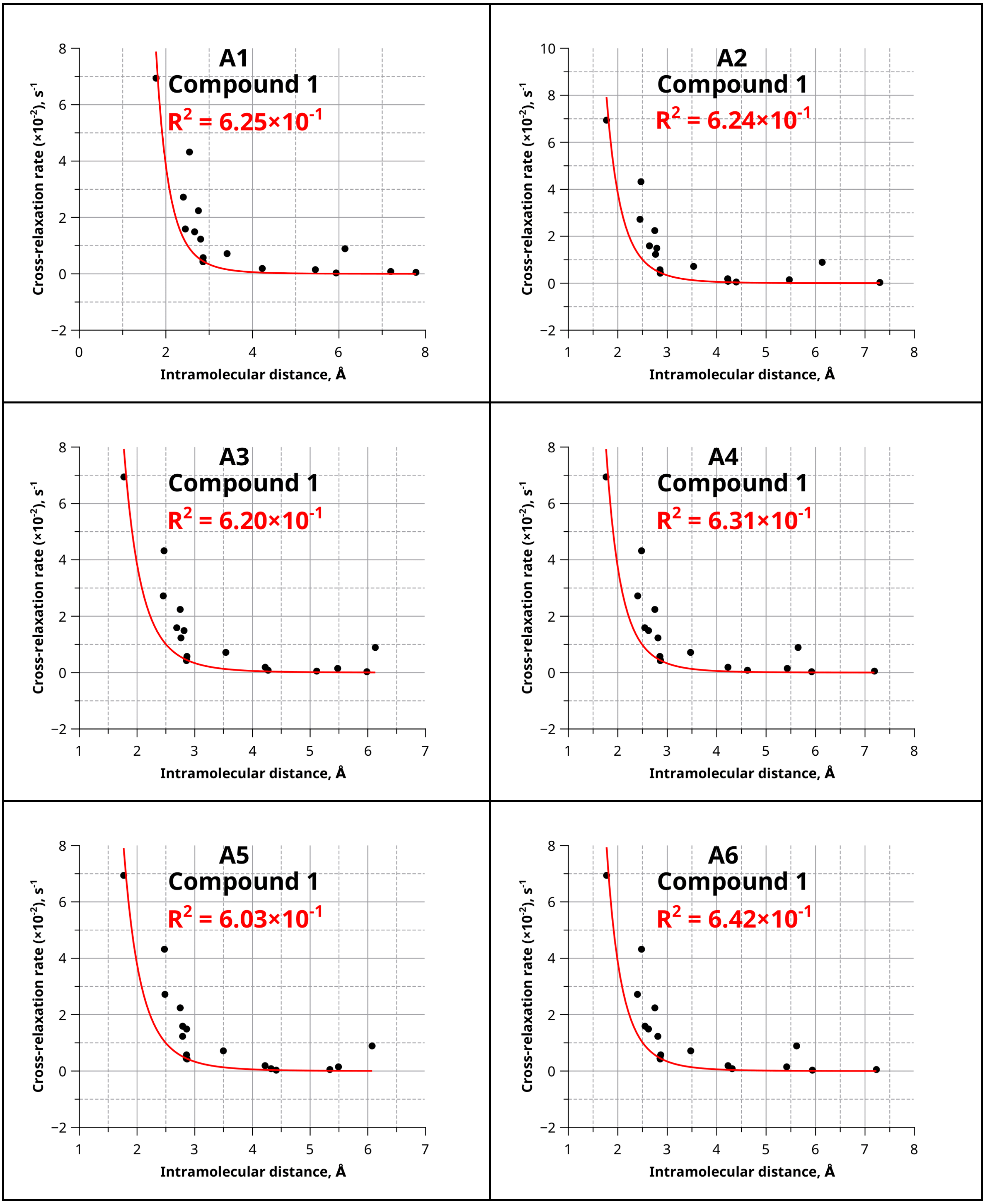

A1
Compound 1
R² = 6.25×10⁻¹
A2
Compound 1
R² = 6.24×10⁻¹
A3
Compound 1
R² = 6.20×10⁻¹
A4
Compound 1
R² = 6.31×10⁻¹
A5
Compound 1
R² = 6.03×10⁻¹
A6
Compound 1
R² = 6.42×10⁻¹
Cross-relaxation rate (×10⁻²), s⁻¹
Intramolecular distance, Å

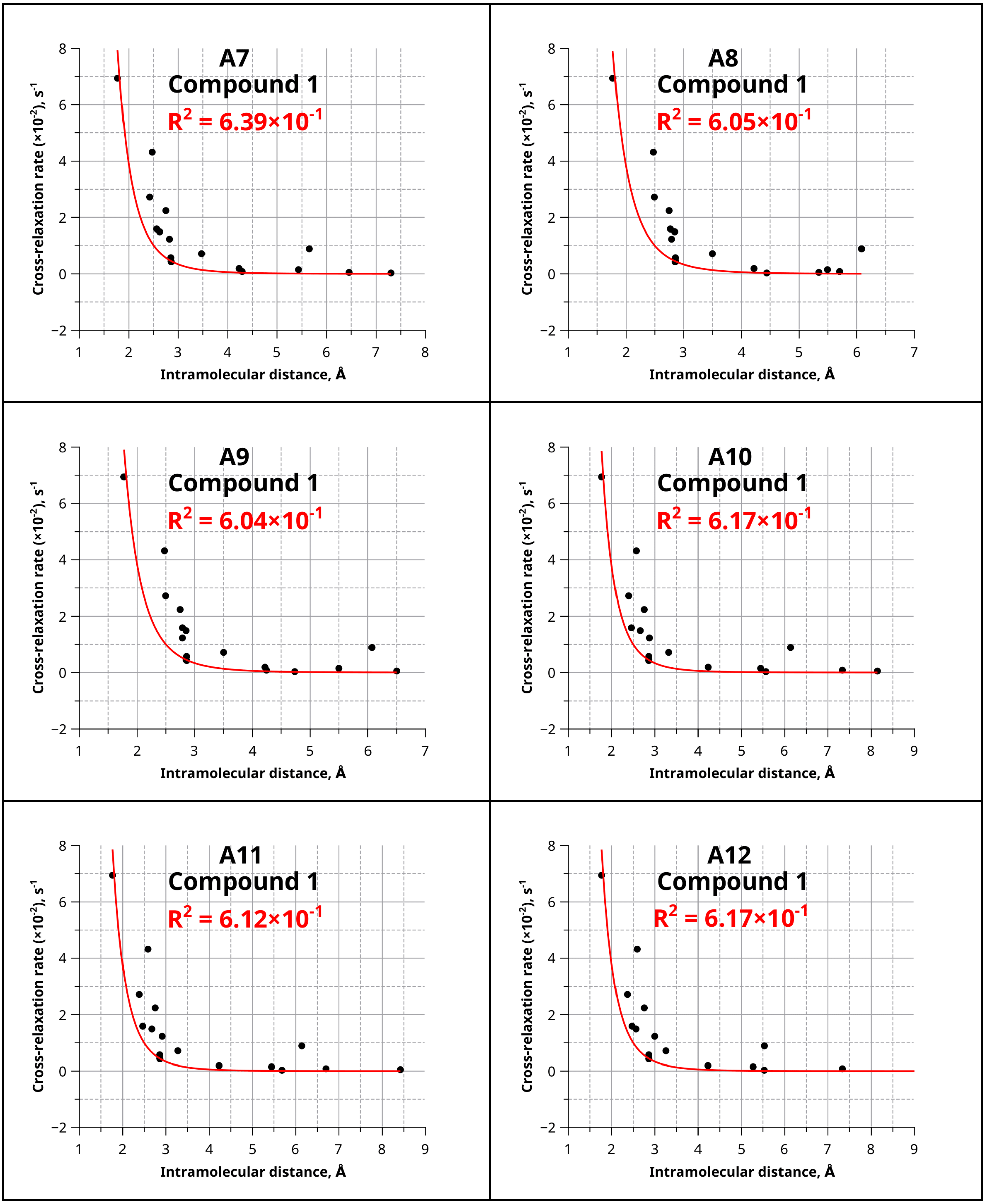
A7
Compound 1
R² = 6.39×10⁻¹
Cross-relaxation rate (×10⁻²), s⁻¹
Intramolecular distance, Å
A8
Compound 1
R² = 6.05×10⁻¹
Cross-relaxation rate (×10⁻²), s⁻¹
Intramolecular distance, Å
A9
Compound 1
R² = 6.04×10⁻¹
Cross-relaxation rate (×10⁻²), s⁻¹
Intramolecular distance, Å
A10
Compound 1
R² = 6.17×10⁻¹
Cross-relaxation rate (×10⁻²), s⁻¹
Intramolecular distance, Å
A11
Compound 1
R² = 6.12×10⁻¹
Cross-relaxation rate (×10⁻²), s⁻¹
Intramolecular distance, Å
A12
Compound 1
R² = 6.17×10⁻¹
Cross-relaxation rate (×10⁻²), s⁻¹
Intramolecular distance, Å

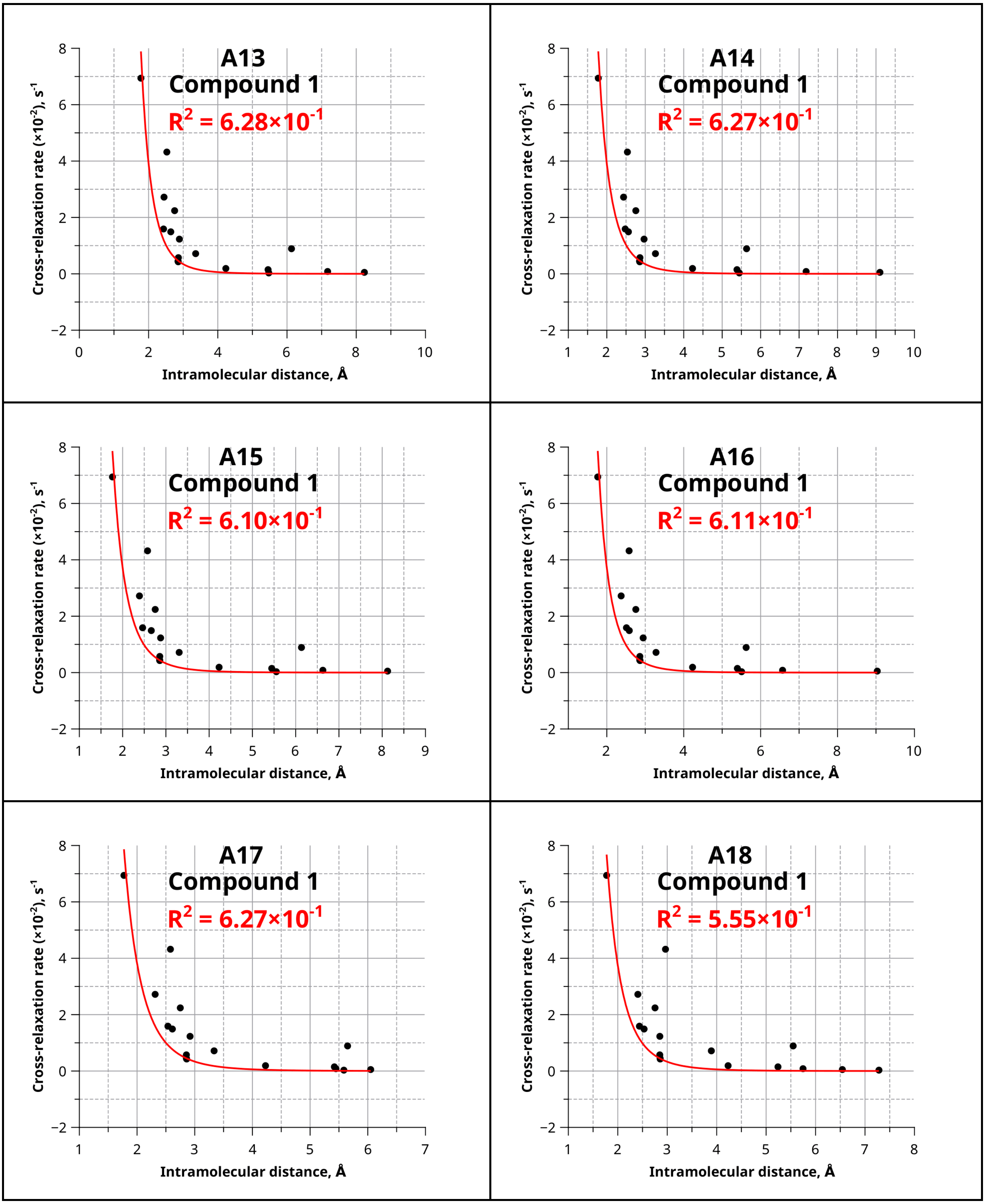
A13
Compound 1
R2 = 6.28×10-1
A14
Compound 1
R2 = 6.27×10-1
A15
Compound 1
R2 = 6.10×10-1
A16
Compound 1
R2 = 6.11×10-1
A17
Compound 1
R2 = 6.27×10-1
A18
Compound 1
R2 = 5.55×10-1
Cross-relaxation rate (×10-2), s-1
Intramolecular distance, Å

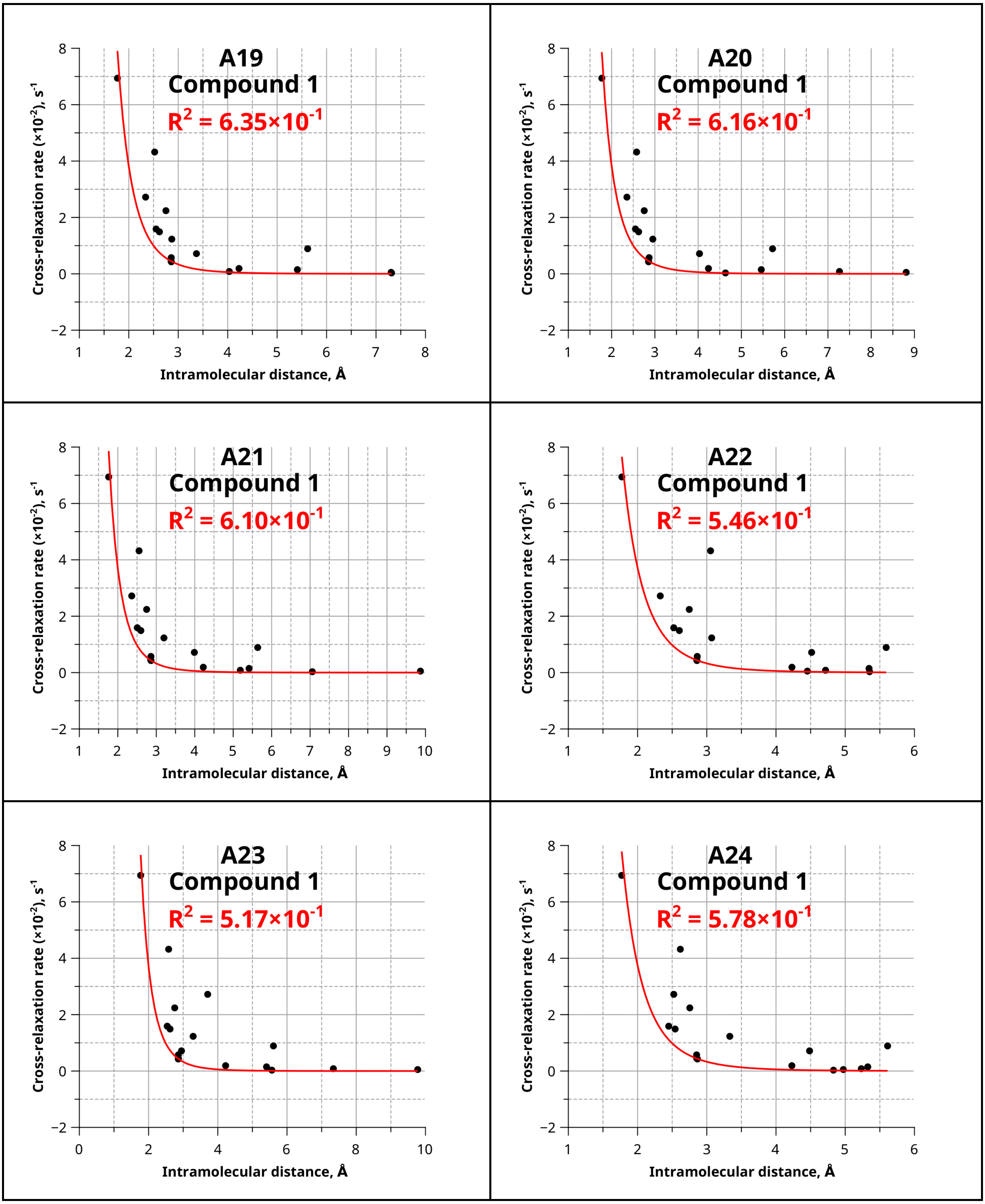
A19
Compound 1
R² = 6.35×10⁻¹
A20
Compound 1
R² = 6.16×10⁻¹
A21
Compound 1
R² = 6.10×10⁻¹
A22
Compound 1
R² = 5.46×10⁻¹
A23
Compound 1
R² = 5.17×10⁻¹
A24
Compound 1
R² = 5.78×10⁻¹
Cross-relaxation rate (×10⁻²), s⁻¹
Intramolecular distance, Å

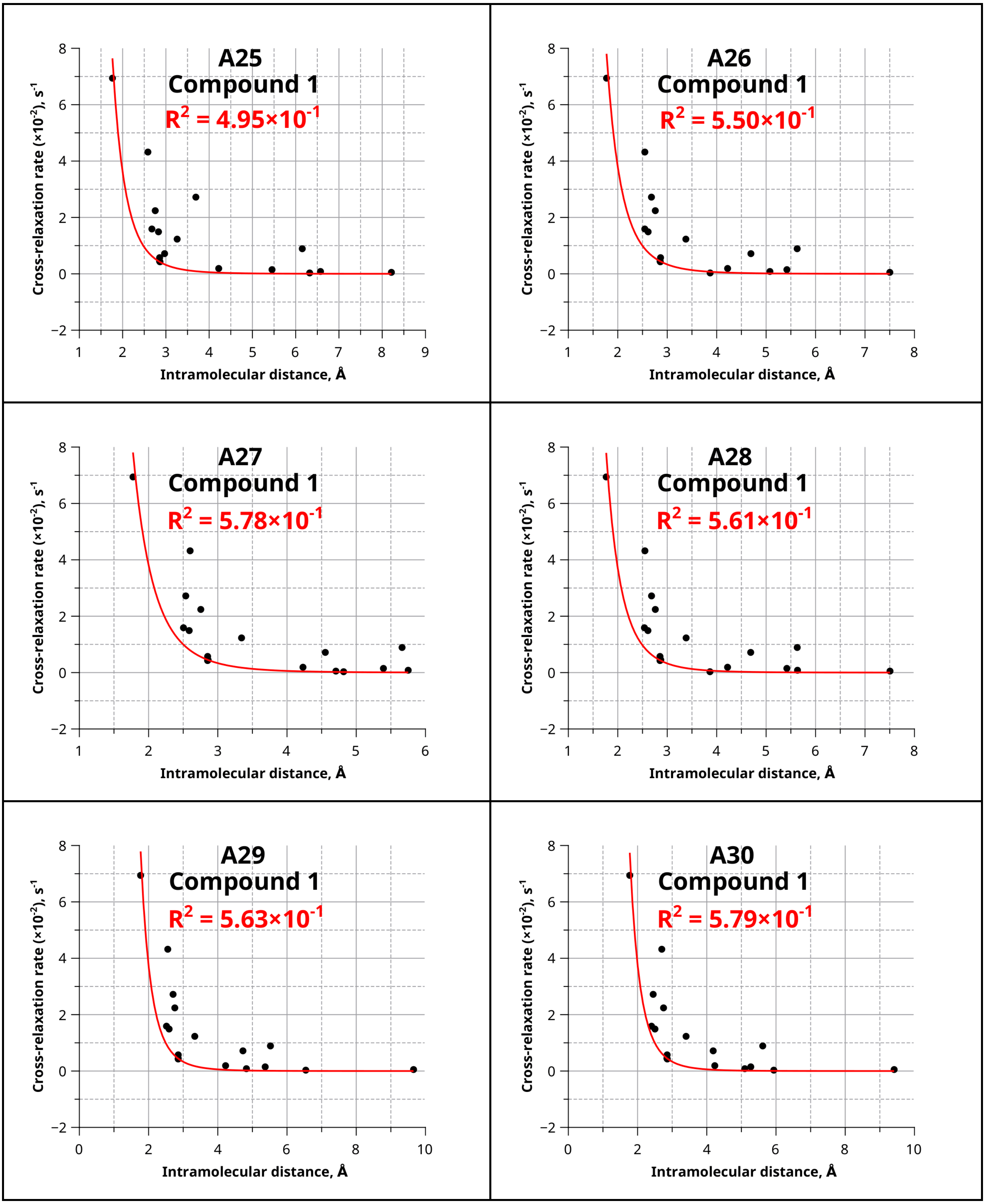
A25
Compound 1
R2 = 4.95×10-1
A26
Compound 1
R2 = 5.50×10-1
A27
Compound 1
R2 = 5.78×10-1
A28
Compound 1
R2 = 5.61×10-1
A29
Compound 1
R2 = 5.63×10-1
A30
Compound 1
R2 = 5.79×10-1
Cross-relaxation rate (×10-2), s-1
Intramolecular distance, Å

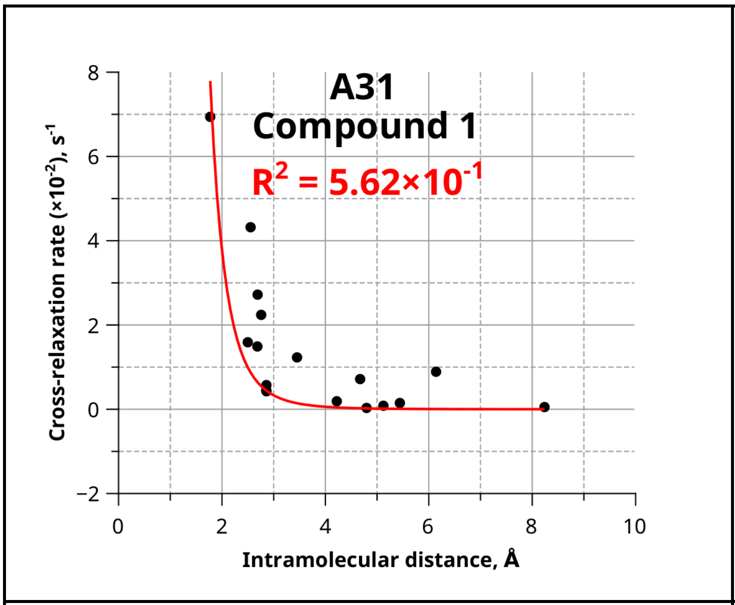
A31
Compound 1
R2 = 5.62×10-1
Cross-relaxation rate (×10-2), s-1
Intramolecular distance, Å
8
6
4
2
0
−2
0
2
4
6
8
10

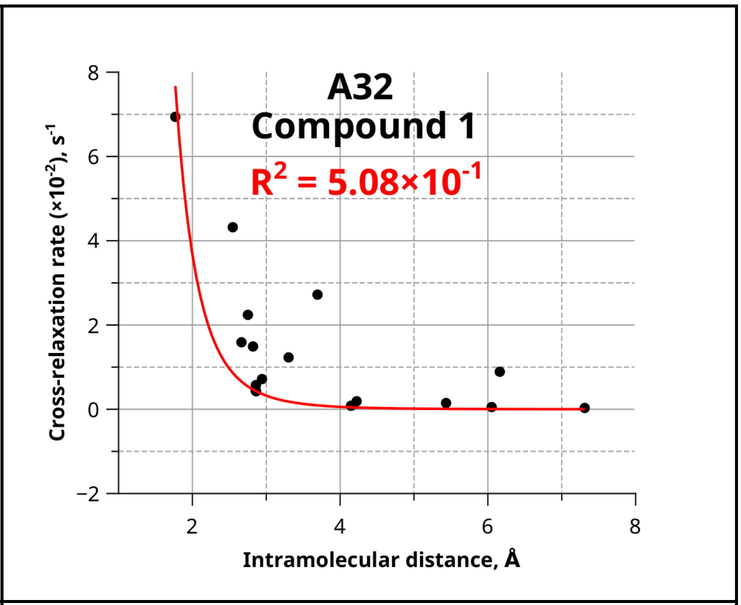
A32
Compound 1
R2 = 5.08×10-1
Cross-relaxation rate (×10-2), s-1
Intramolecular distance, Å
8
6
4
2
0
−2
2
4
6
8

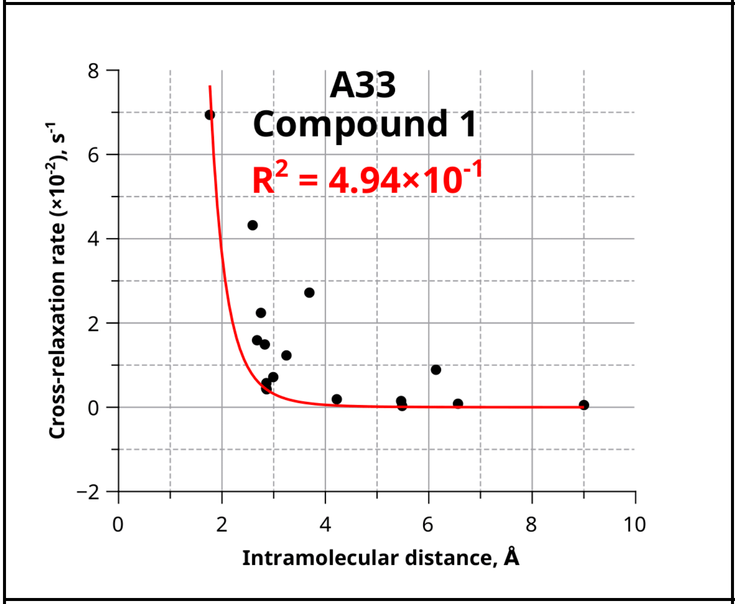
A33
Compound 1
R2 = 4.94×10-1
Cross-relaxation rate (×10-2), s-1
Intramolecular distance, Å
8
6
4
2
0
−2
0
2
4
6
8
10

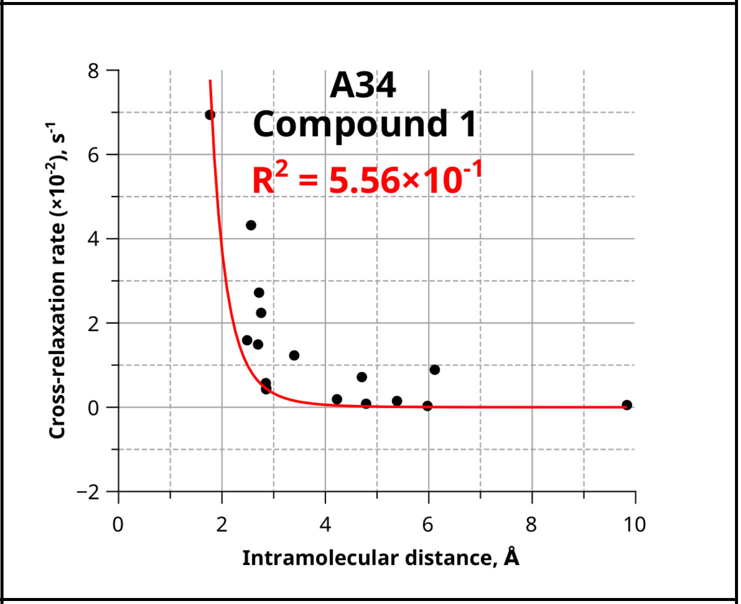
A34
Compound 1
R2 = 5.56×10-1
Cross-relaxation rate (×10-2), s-1
Intramolecular distance, Å
8
6
4
2
0
−2
0
2
4
6
8
10

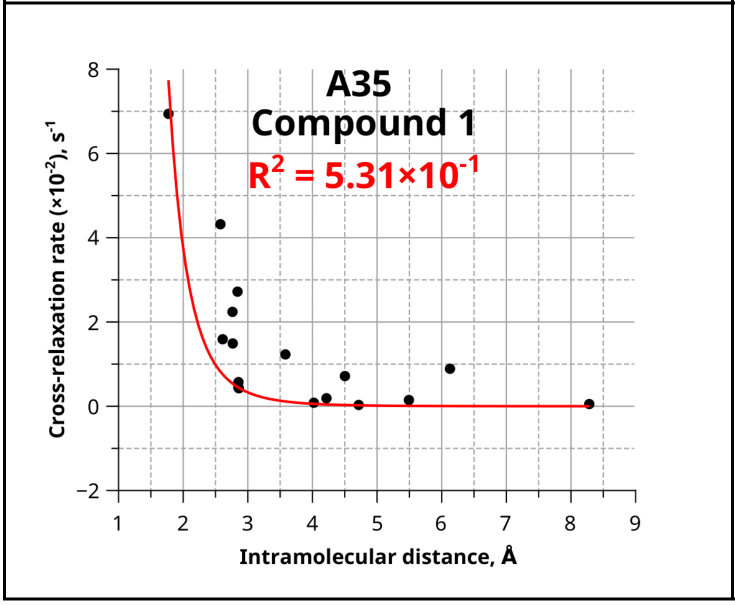
A35
Compound 1
R2 = 5.31×10-1
Cross-relaxation rate (×10-2), s-1
Intramolecular distance, Å
8
6
4
2
0
−2
1
2
3
4
5
6
7
8
9

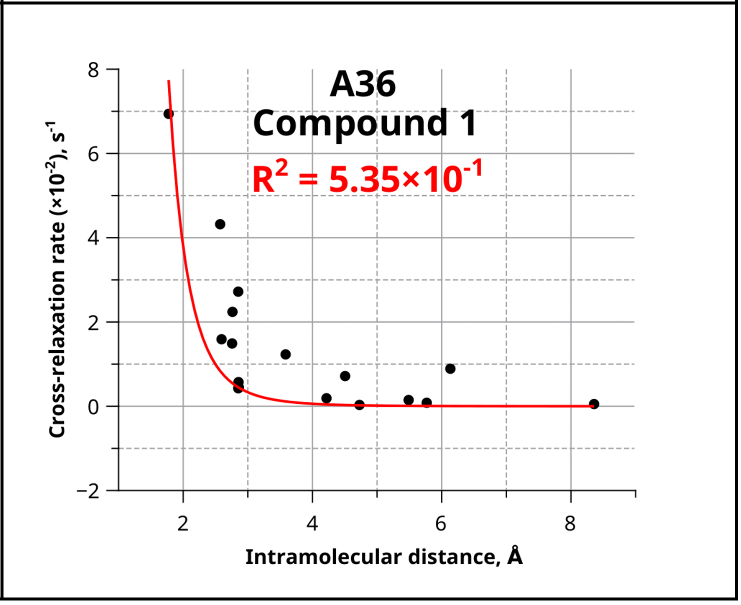
A36
Compound 1
R2 = 5.35×10-1
Cross-relaxation rate (×10-2), s-1
Intramolecular distance, Å
8
6
4
2
0
−2
2
4
6
8

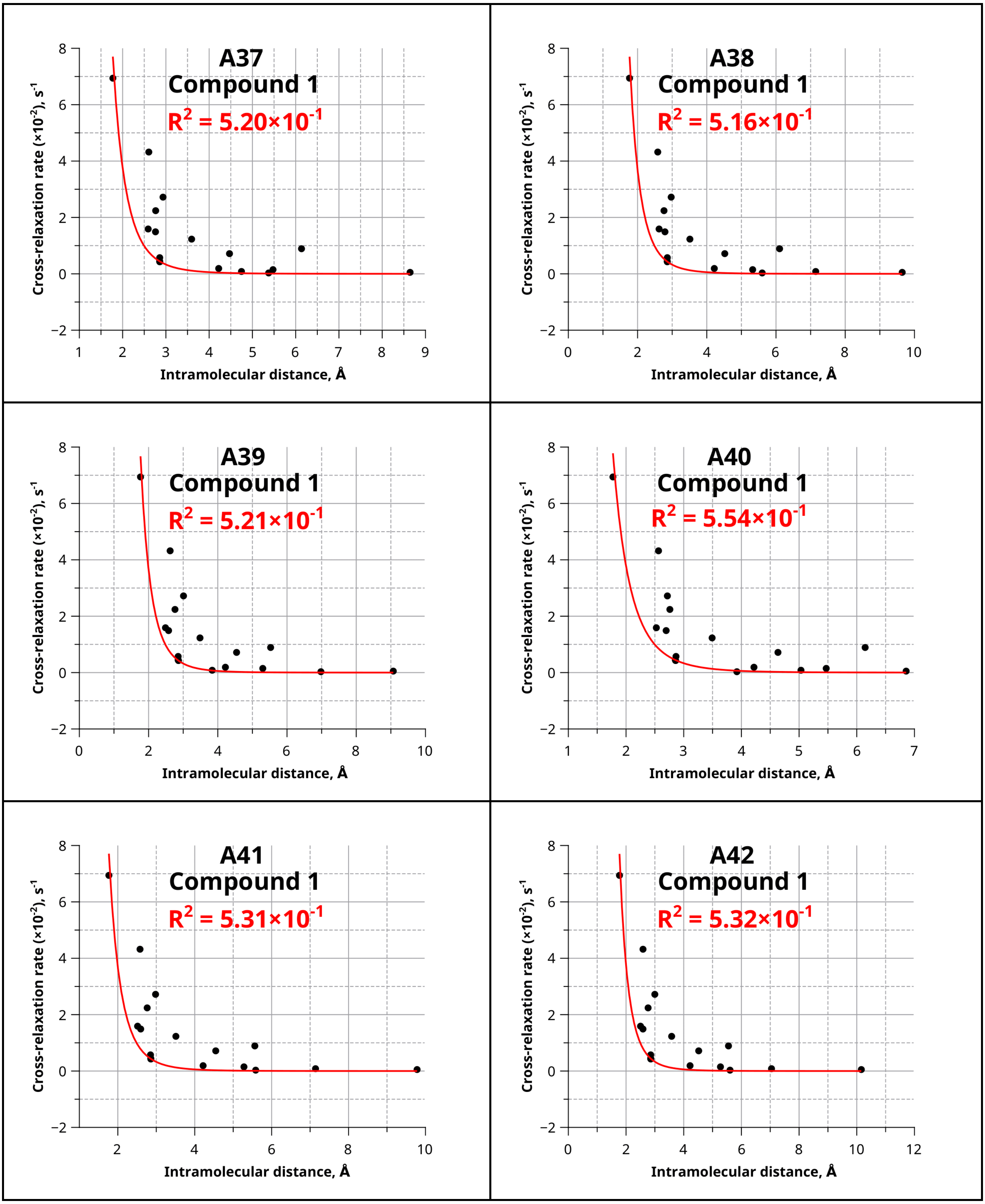
A37
Compound 1
R2 = 5.20×10-1
A38
Compound 1
R2 = 5.16×10-1
A39
Compound 1
R2 = 5.21×10-1
A40
Compound 1
R2 = 5.54×10-1
A41
Compound 1
R2 = 5.31×10-1
A42
Compound 1
R2 = 5.32×10-1
Cross-relaxation rate (×10-2), s-1
Intramolecular distance, Å

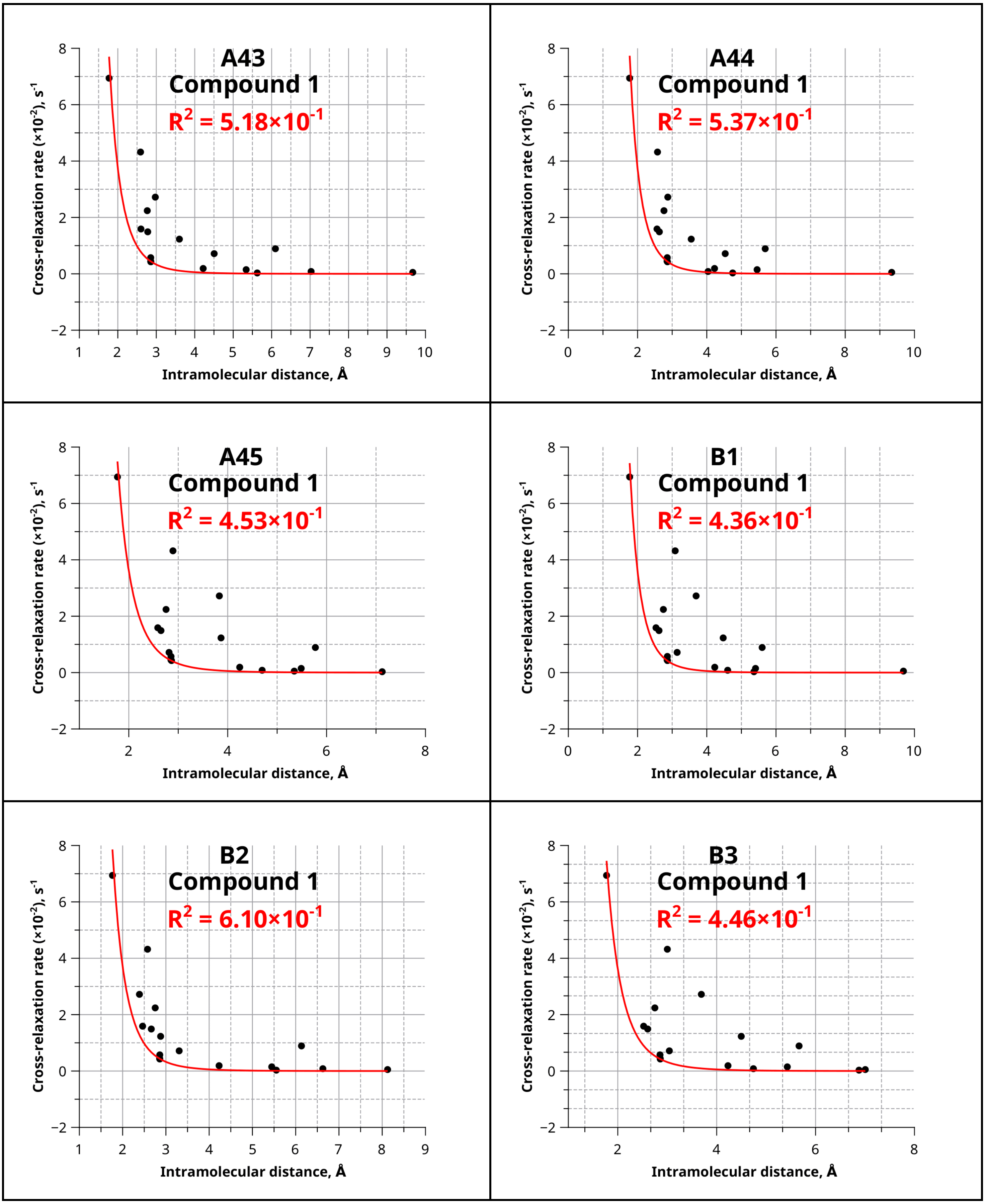
A43
Compound 1
R2 = 5.18×10-1
A44
Compound 1
R2 = 5.37×10-1
A45
Compound 1
R2 = 4.53×10-1
B1
Compound 1
R2 = 4.36×10-1
B2
Compound 1
R2 = 6.10×10-1
B3
Compound 1
R2 = 4.46×10-1
Cross-relaxation rate (×10-2), s-1
Intramolecular distance, Å

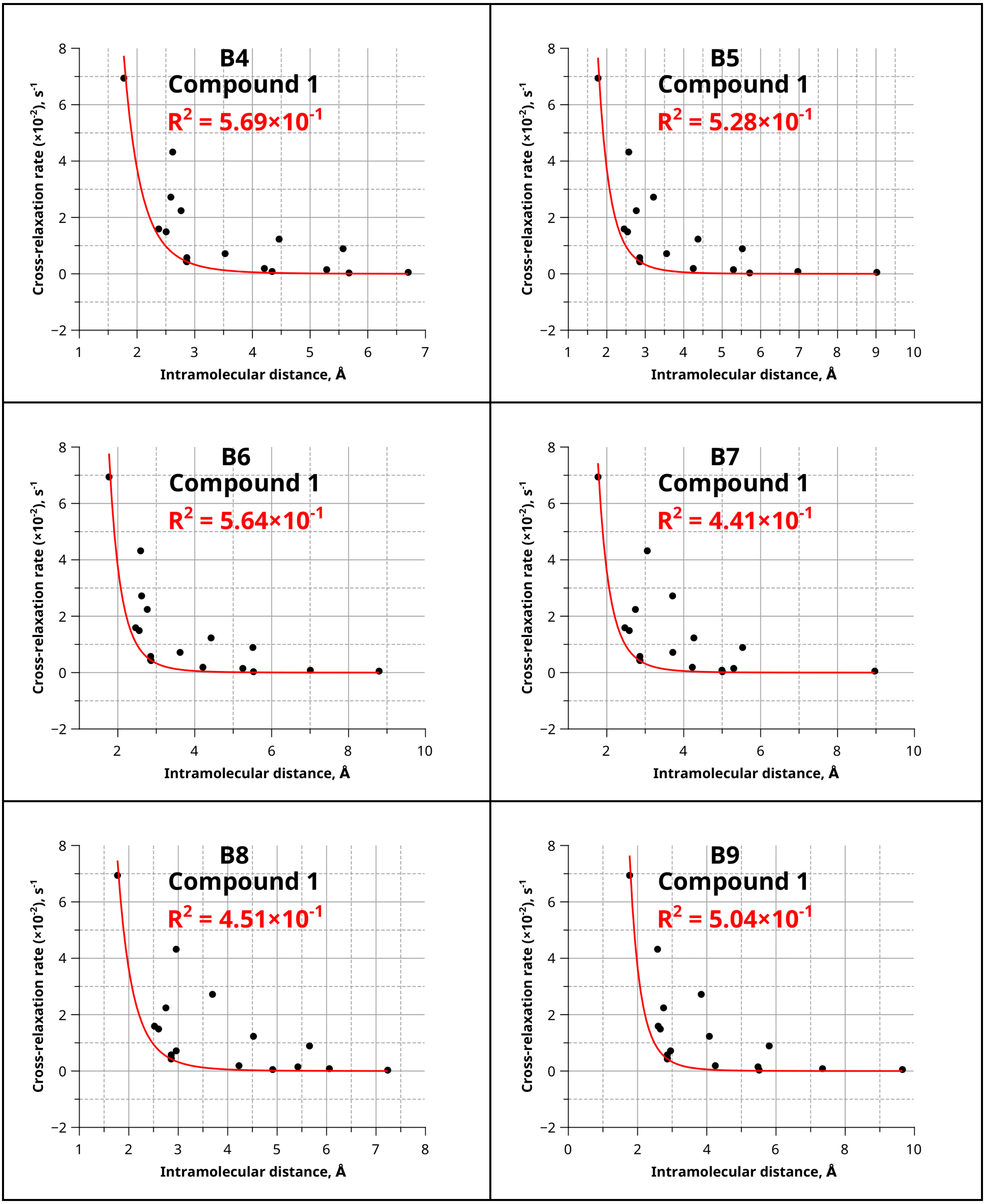
B4
Compound 1
R2 = 5.69×10-1
B5
Compound 1
R2 = 5.28×10-1
B6
Compound 1
R2 = 5.64×10-1
B7
Compound 1
R2 = 4.41×10-1
B8
Compound 1
R2 = 4.51×10-1
B9
Compound 1
R2 = 5.04×10-1
Cross-relaxation rate (×10-2), s-1
Intramolecular distance, Å

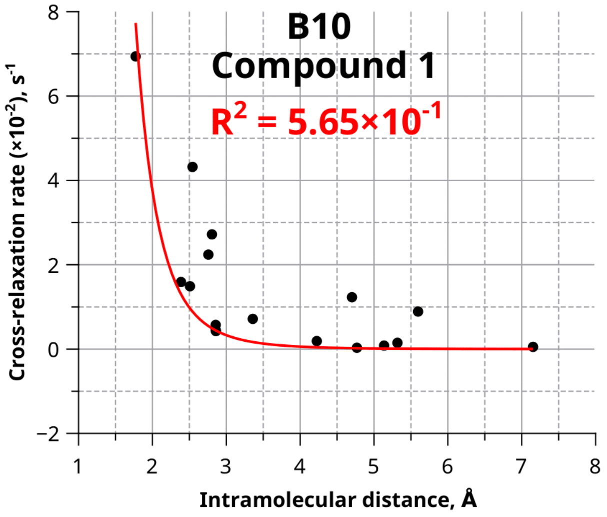
B10
Compound 1
R2 = 5.65×10-1
Cross-relaxation rate (×10-2), s-1
Intramolecular distance, Å

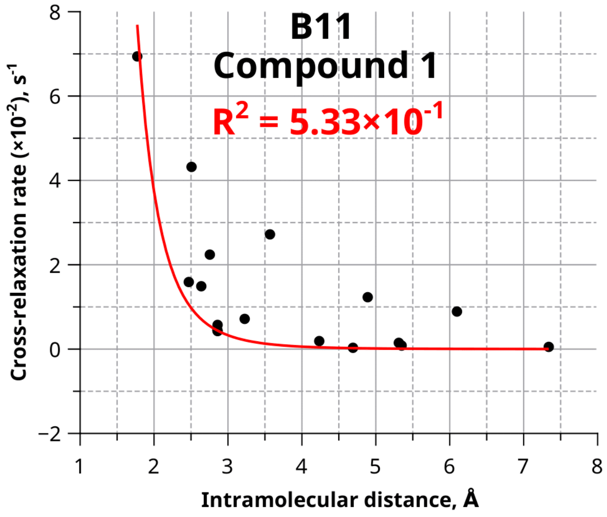
B11
Compound 1
R2 = 5.33×10-1
Cross-relaxation rate (×10-2), s-1
Intramolecular distance, Å

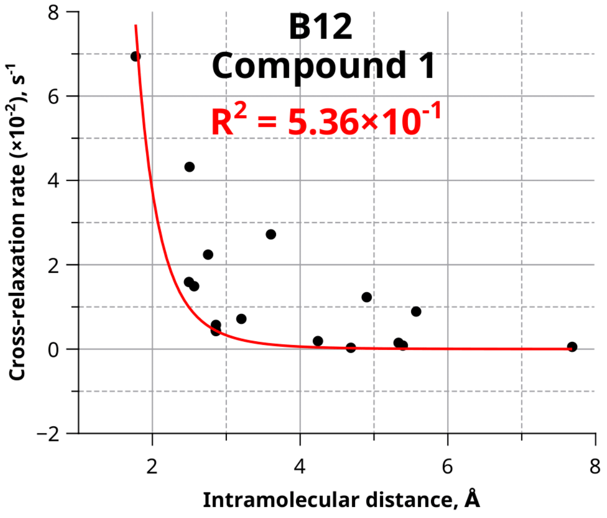
B12
Compound 1
R2 = 5.36×10-1
Cross-relaxation rate (×10-2), s-1
Intramolecular distance, Å

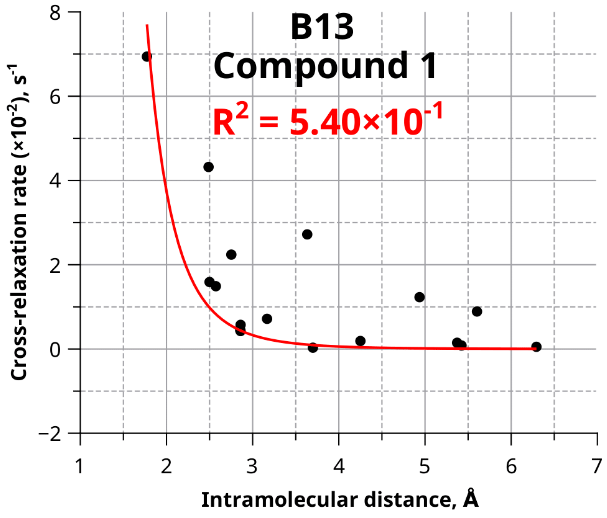
B13
Compound 1
R2 = 5.40×10-1
Cross-relaxation rate (×10-2), s-1
Intramolecular distance, Å

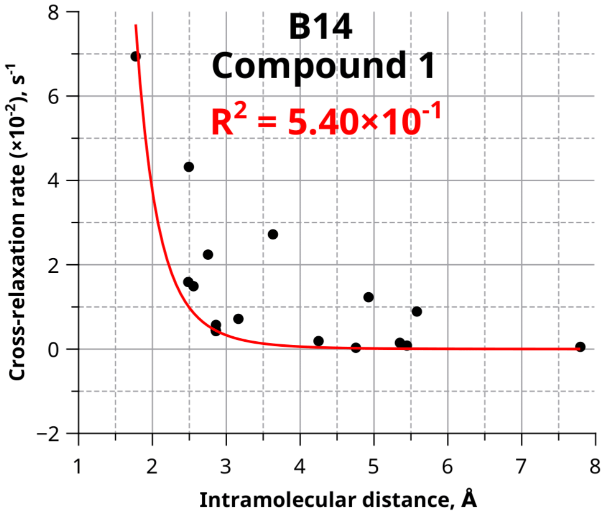
B14
Compound 1
R2 = 5.40×10-1
Cross-relaxation rate (×10-2), s-1
Intramolecular distance, Å

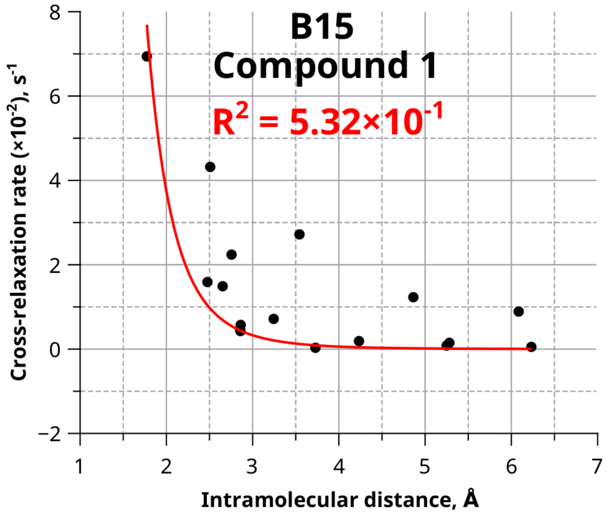
B15
Compound 1
R2 = 5.32×10-1
Cross-relaxation rate (×10-2), s-1
Intramolecular distance, Å

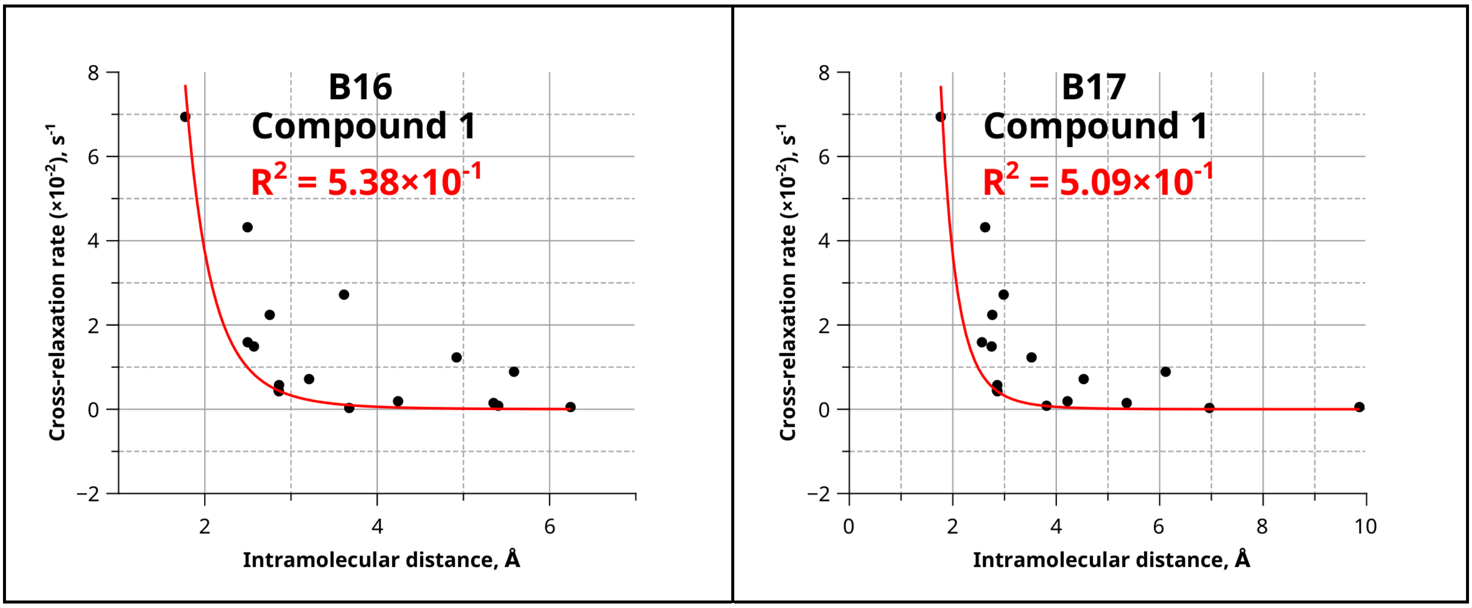

Table S12. Dependence of the cross-relaxation rate on the internuclear distance for conformers for compound 2.

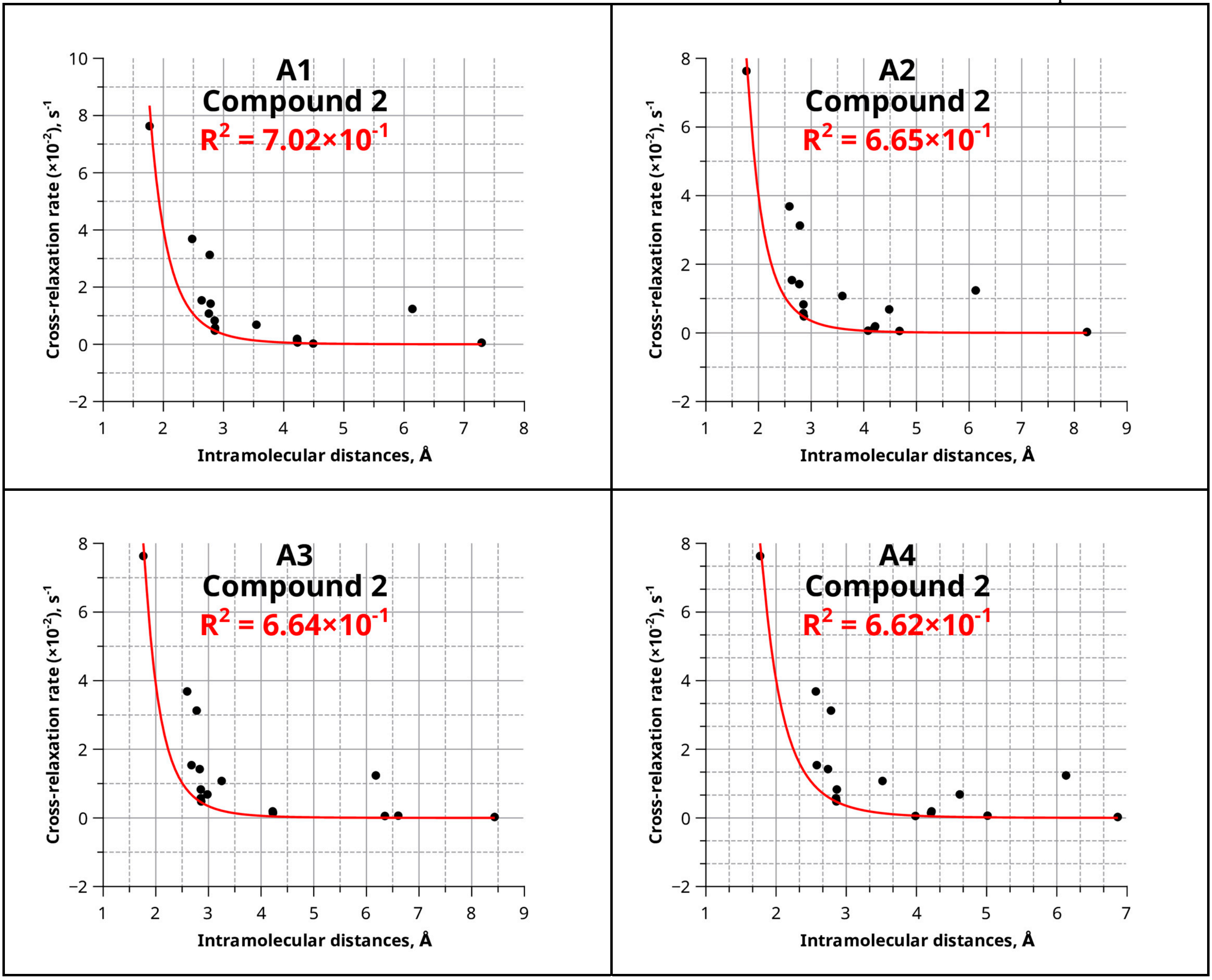

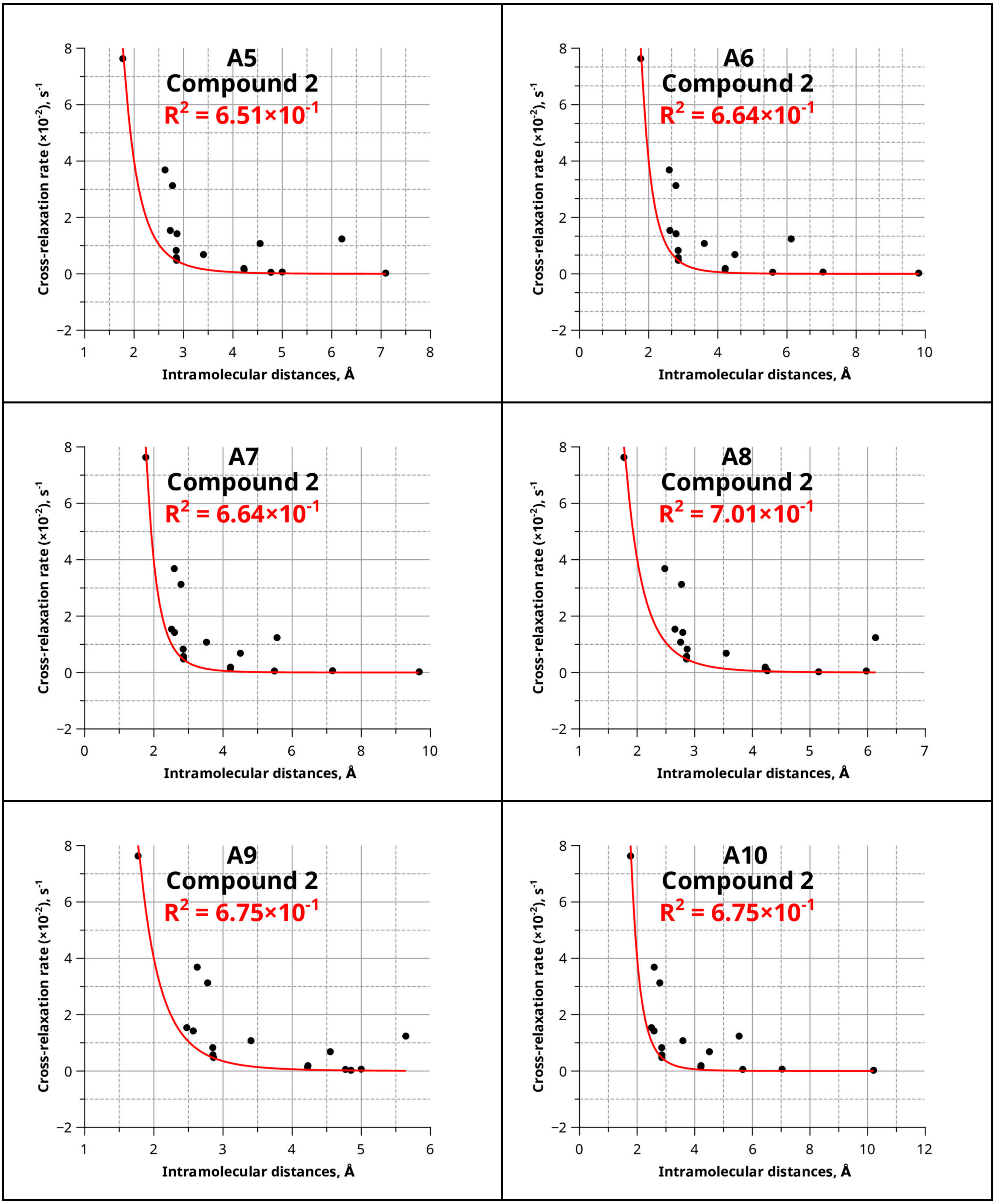
A5
Compound 2
R2 = 6.51×10-1
A6
Compound 2
R2 = 6.64×10-1
A7
Compound 2
R2 = 6.64×10-1
A8
Compound 2
R2 = 7.01×10-1
A9
Compound 2
R2 = 6.75×10-1
A10
Compound 2
R2 = 6.75×10-1
Cross-relaxation rate (×10-2), s-1
Intramolecular distances, Å

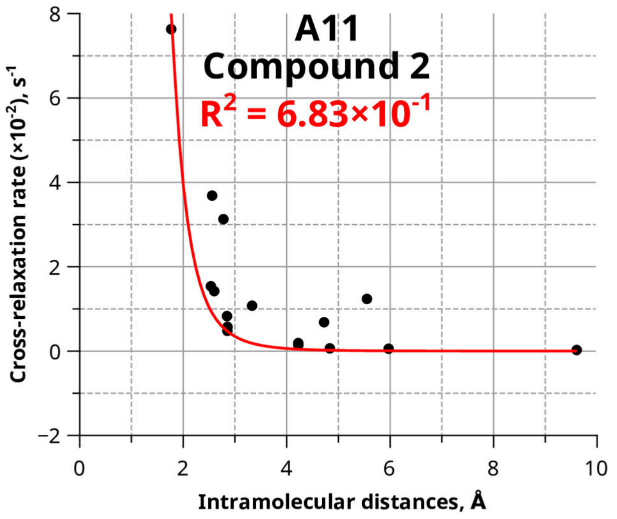
A11
Compound 2
R^2 = 6.83×10^{-1}
Cross-relaxation rate (×10^{-2}), s^{-1}
Intramolecular distances, Å

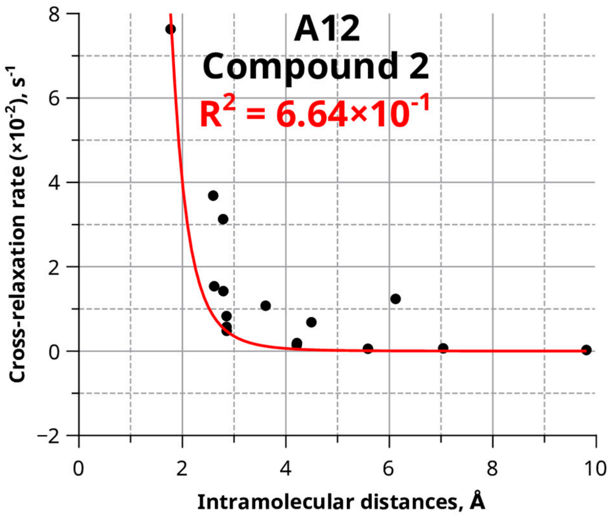
A12
Compound 2
R^2 = 6.64×10^{-1}
Cross-relaxation rate (×10^{-2}), s^{-1}
Intramolecular distances, Å

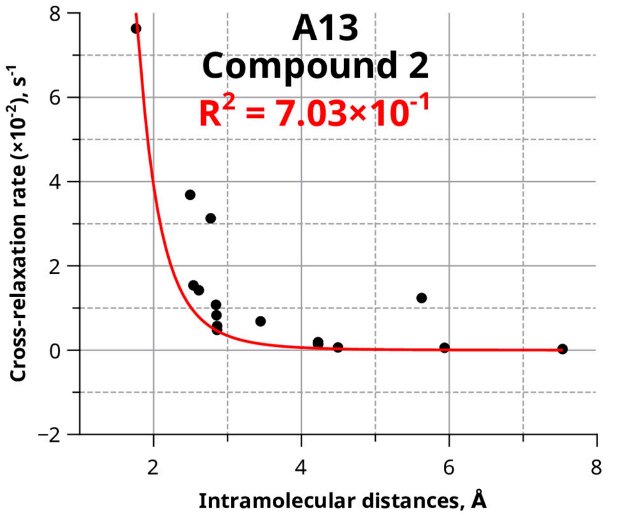
A13
Compound 2
R^2 = 7.03×10^{-1}
Cross-relaxation rate (×10^{-2}), s^{-1}
Intramolecular distances, Å

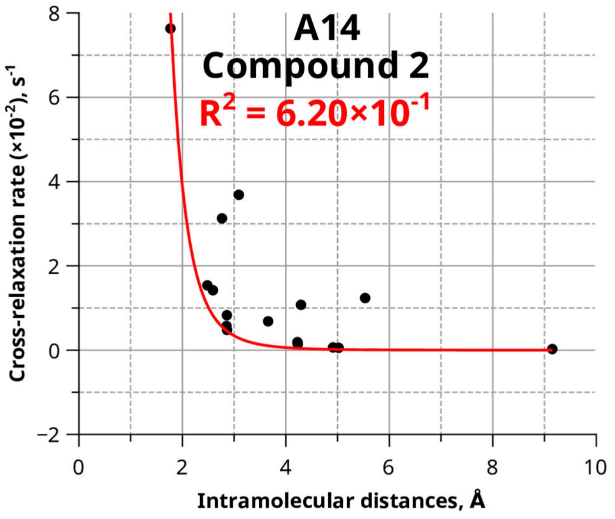
A14
Compound 2
R^2 = 6.20×10^{-1}
Cross-relaxation rate (×10^{-2}), s^{-1}
Intramolecular distances, Å

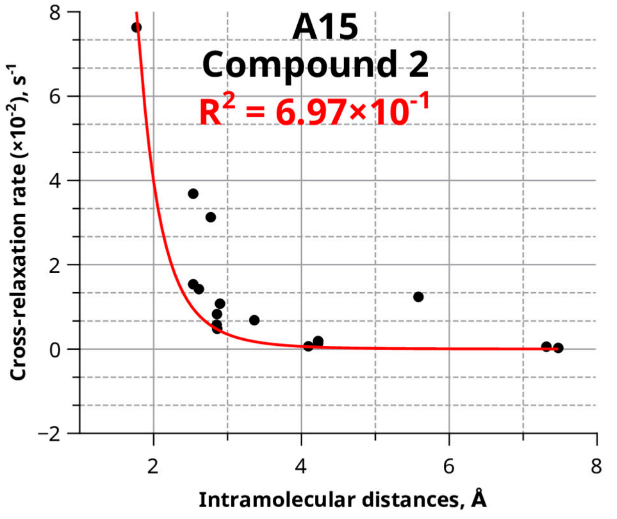
A15
Compound 2
R^2 = 6.97×10^{-1}
Cross-relaxation rate (×10^{-2}), s^{-1}
Intramolecular distances, Å

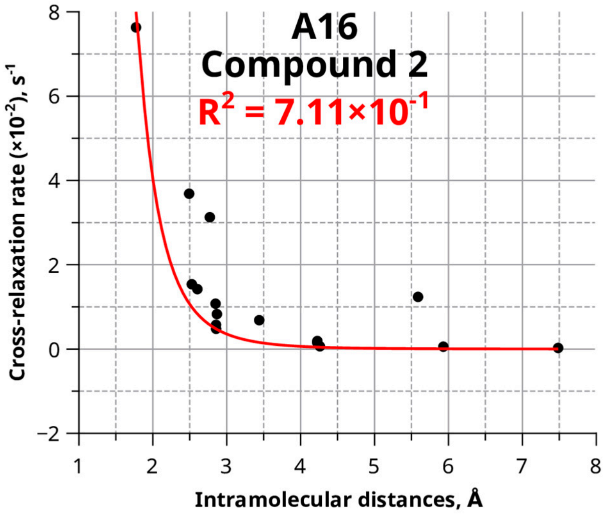
A16
Compound 2
R^2 = 7.11×10^{-1}
Cross-relaxation rate (×10^{-2}), s^{-1}
Intramolecular distances, Å

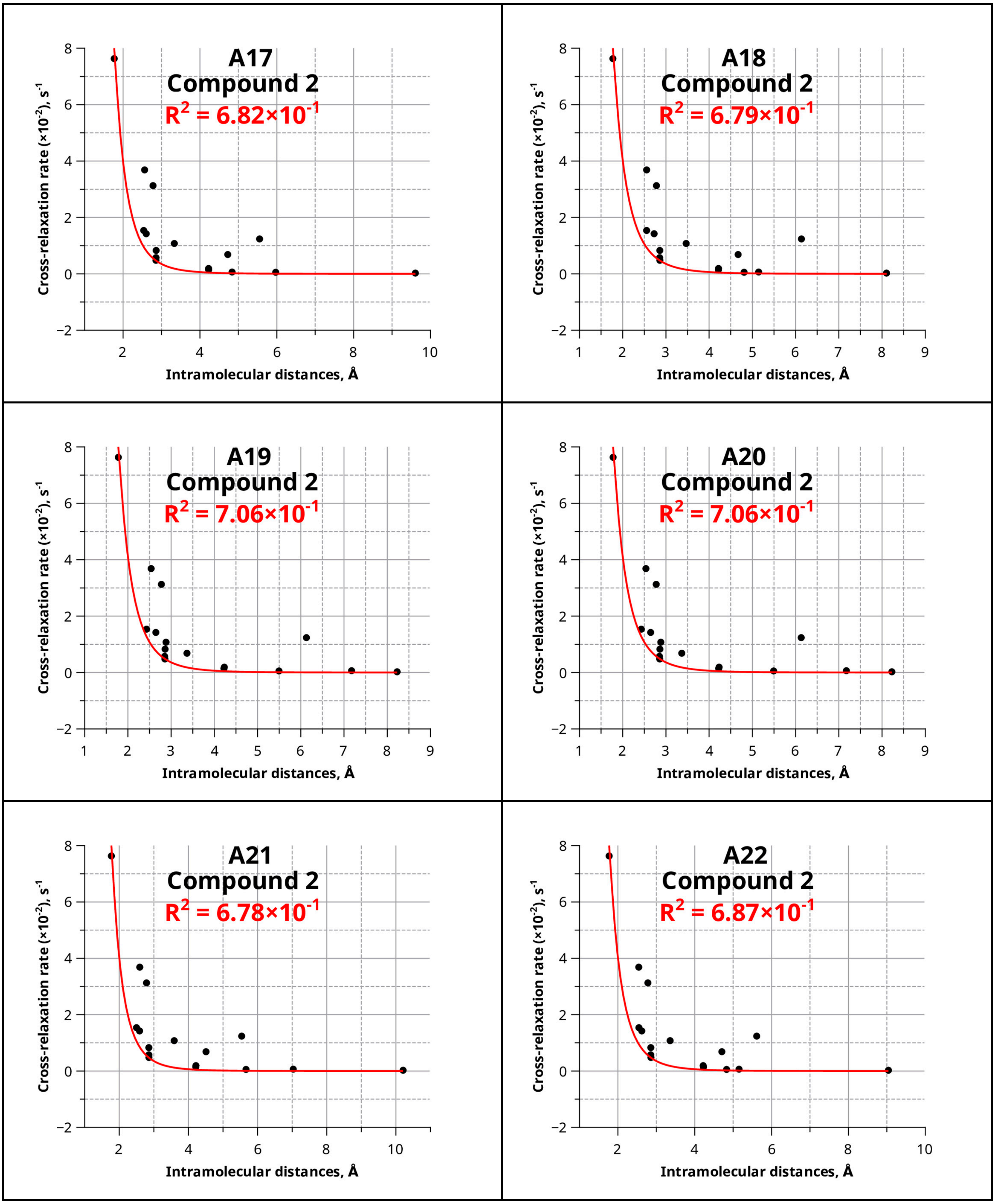
A17
Compound 2
R² = 6.82×10⁻¹
A18
Compound 2
R² = 6.79×10⁻¹
A19
Compound 2
R² = 7.06×10⁻¹
A20
Compound 2
R² = 7.06×10⁻¹
A21
Compound 2
R² = 6.78×10⁻¹
A22
Compound 2
R² = 6.87×10⁻¹
Cross-relaxation rate (×10⁻²), s⁻¹
Intramolecular distances, Å

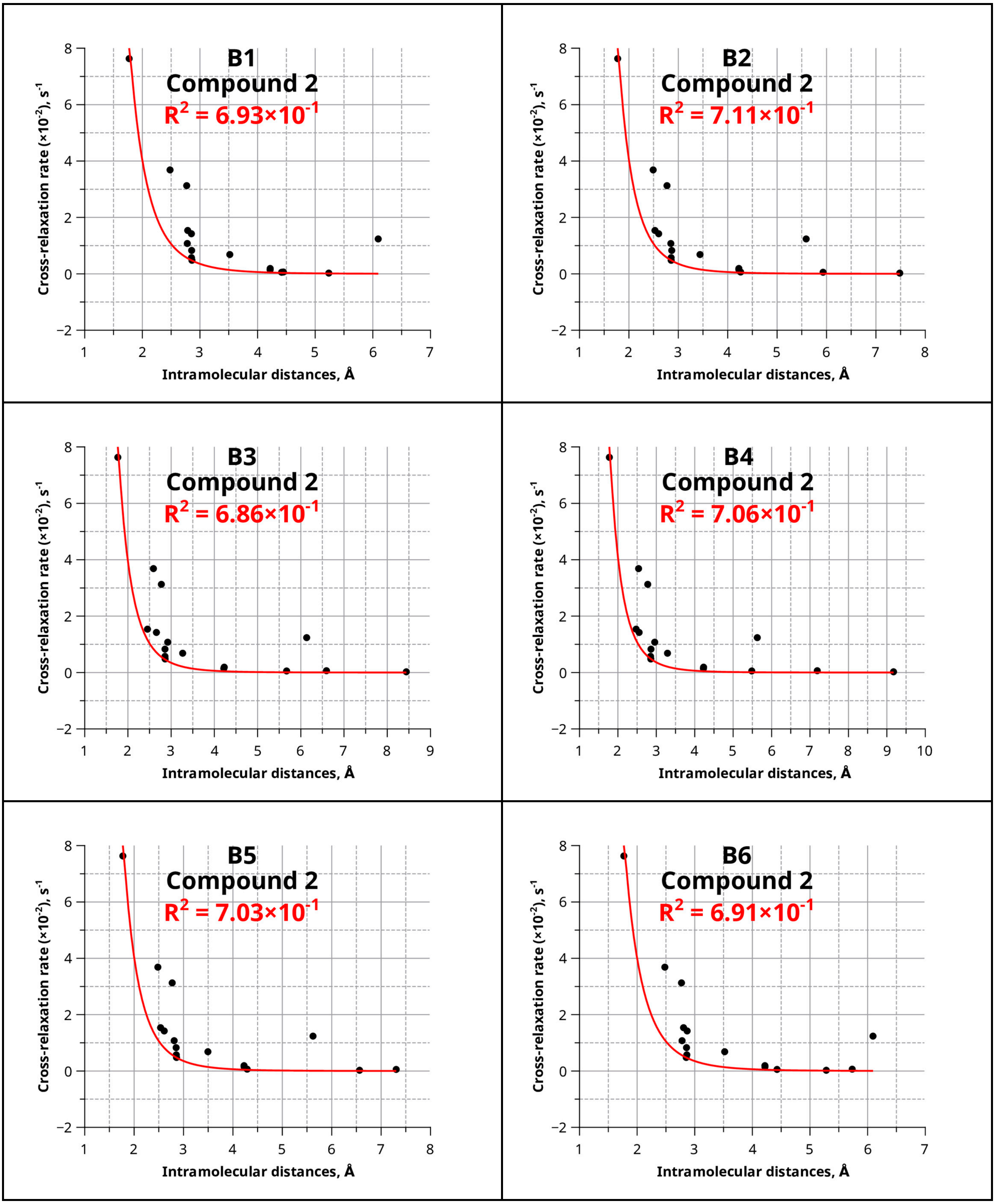

B1
Compound 2
R² = 6.93×10⁻¹
B2
Compound 2
R² = 7.11×10⁻¹
B3
Compound 2
R² = 6.86×10⁻¹
B4
Compound 2
R² = 7.06×10⁻¹
B5
Compound 2
R² = 7.03×10⁻¹
B6
Compound 2
R² = 6.91×10⁻¹
Cross-relaxation rate (×10⁻²), s⁻¹
Intramolecular distances, Å

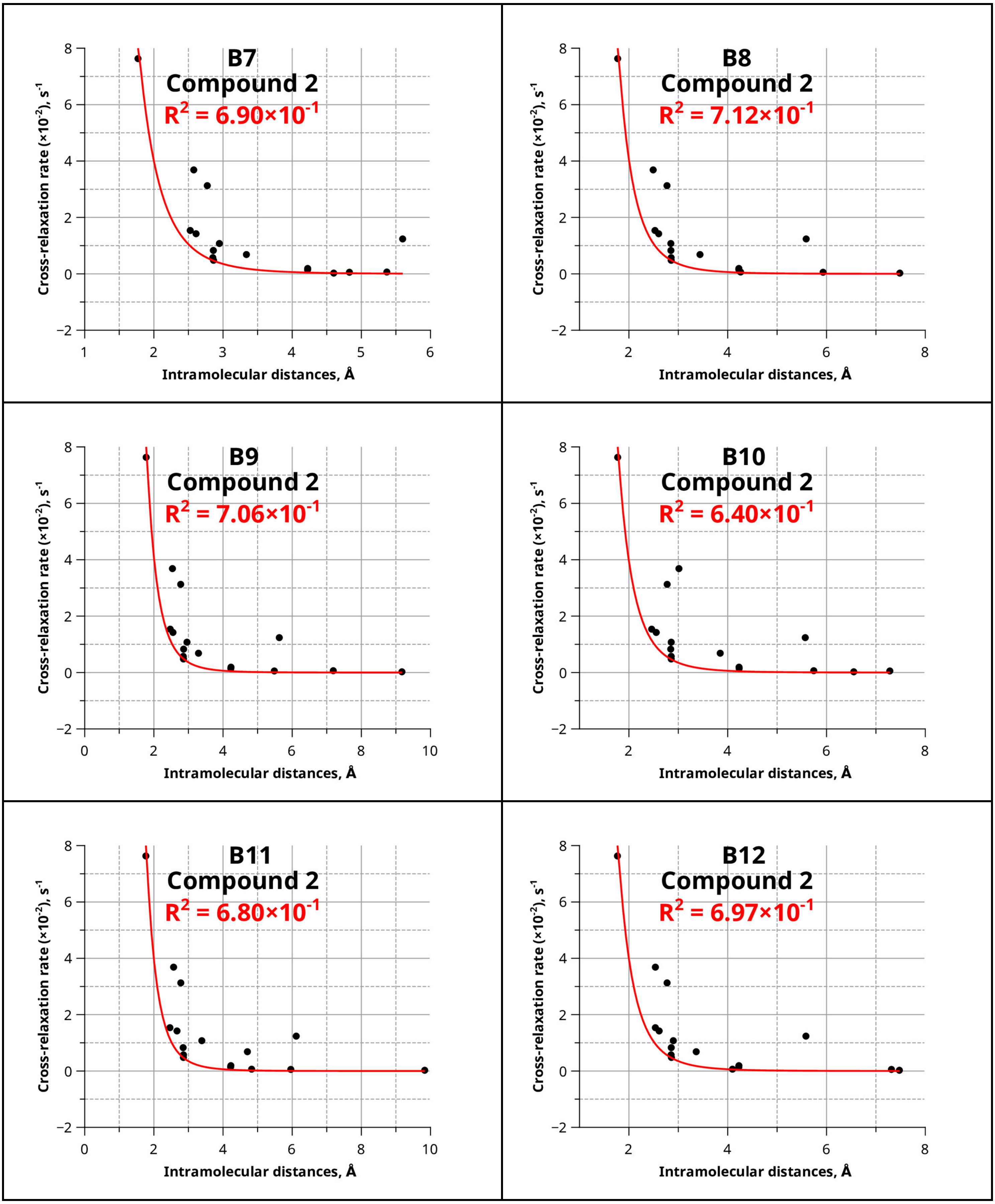

B7
Compound 2
R² = 6.90×10⁻¹
B8
Compound 2
R² = 7.12×10⁻¹
B9
Compound 2
R² = 7.06×10⁻¹
B10
Compound 2
R² = 6.40×10⁻¹
B11
Compound 2
R² = 6.80×10⁻¹
B12
Compound 2
R² = 6.97×10⁻¹
Cross-relaxation rate (×10⁻²), s⁻¹
Intramolecular distances, Å

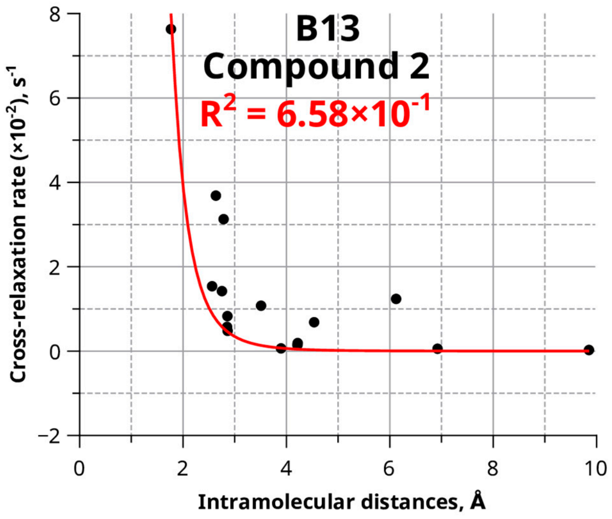
B13
Compound 2
$R^2 = 6.58×10^{-1}$
Cross-relaxation rate (×10^{-2}), s^{-1}
Intramolecular distances, Å

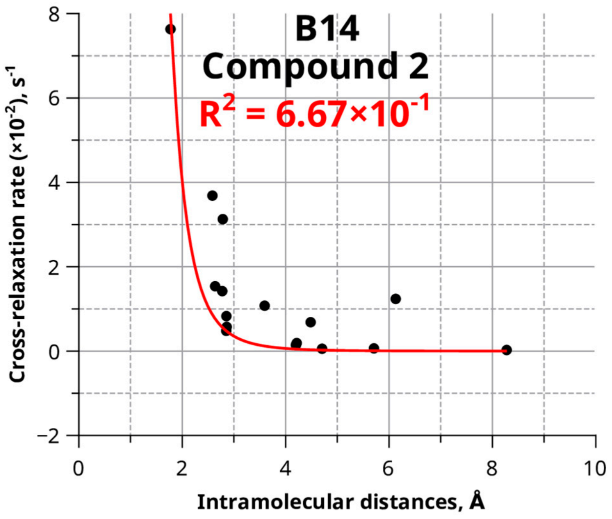
B14
Compound 2
$R^2 = 6.67×10^{-1}$
Cross-relaxation rate (×10^{-2}), s^{-1}
Intramolecular distances, Å

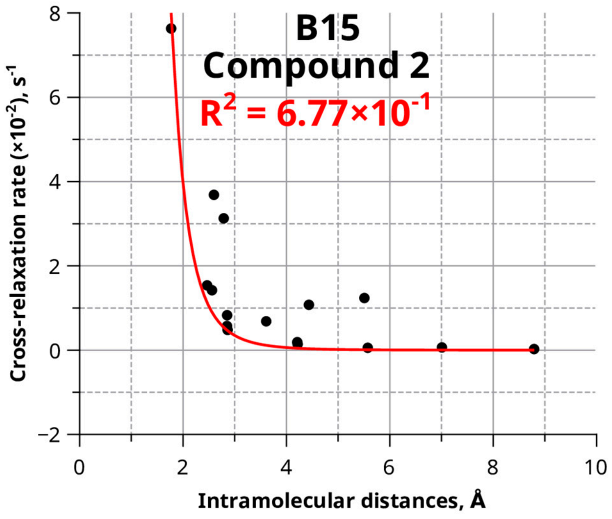
B15
Compound 2
$R^2 = 6.77×10^{-1}$
Cross-relaxation rate (×10^{-2}), s^{-1}
Intramolecular distances, Å

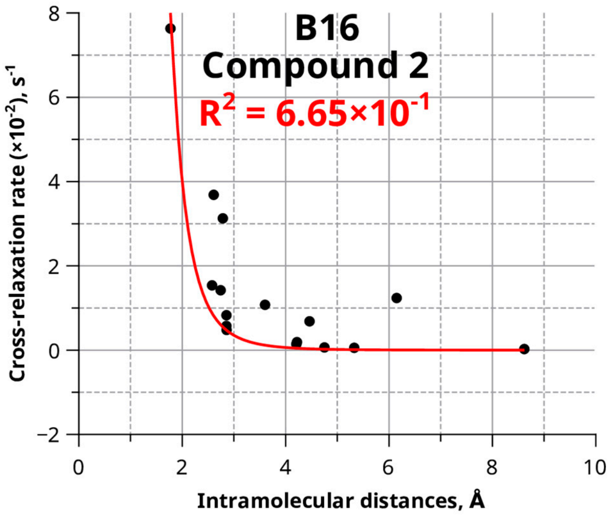
B16
Compound 2
$R^2 = 6.65×10^{-1}$
Cross-relaxation rate (×10^{-2}), s^{-1}
Intramolecular distances, Å

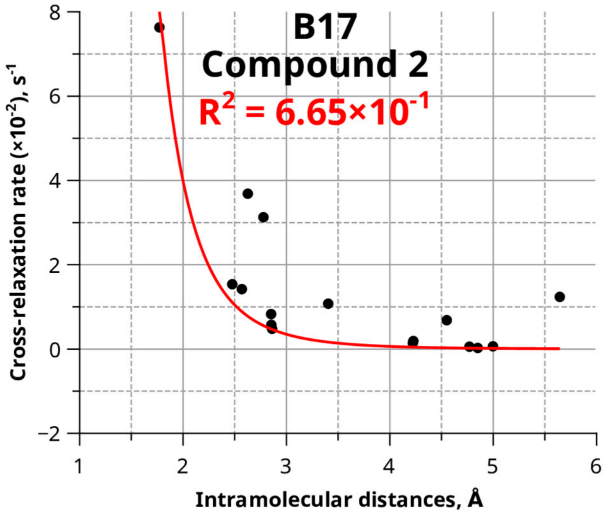
B17
Compound 2
$R^2 = 6.65×10^{-1}$
Cross-relaxation rate (×10^{-2}), s^{-1}
Intramolecular distances, Å

Table S13. Structural information in the form of XYZ for compound 1.

A1

C	-1.279845000	2.636155000	0.148521000
C	-2.279201000	1.527098000	-0.017577000
H	0.537562000	-3.747560000	-4.850084000
C	-0.741171000	-2.963407000	-1.811525000
C	-0.638692000	-3.560137000	-3.062382000
C	0.454125000	-3.285646000	-3.875464000
C	1.441954000	-2.417728000	-3.425289000
C	1.338272000	-1.822169000	-2.173560000
C	0.239385000	-2.082796000	-1.349144000
H	-1.592866000	-3.188566000	-1.181502000
H	-1.410240000	-4.239821000	-3.399183000
H	2.300256000	-2.204050000	-4.048382000
H	2.125282000	-1.164975000	-1.831766000
N	-0.383863000	-0.017901000	0.017449000
C	-1.782653000	0.143118000	-0.354960000
C	0.140897000	2.340511000	-0.215027000
C	0.447164000	0.946754000	-0.701698000
C	1.006508000	3.367916000	-0.151020000
C	0.059608000	-1.416632000	0.011611000
P	1.523322000	-1.623246000	1.117486000
O	0.859385000	-1.320451000	2.535384000
O	1.768156000	-3.211650000	1.179556000
C	1.671583000	-1.077516000	3.710152000

C	0.805768000	-0.402072000	4.749416000
C	2.863531000	-3.865609000	0.492172000
C	3.396529000	-4.975710000	1.371512000
H	2.048678000	-2.037150000	4.068963000
H	2.519187000	-0.452874000	3.428125000
H	3.631608000	-3.126016000	0.270508000
H	2.474086000	-4.258794000	-0.447275000
H	4.191119000	-5.510659000	0.848576000
H	3.805923000	-4.575791000	2.299120000
H	2.609360000	-5.687730000	1.619049000
H	-0.043823000	-1.030527000	5.015788000
H	1.390831000	-0.213522000	5.650842000
H	0.429047000	0.549364000	4.376010000
H	-1.930517000	-0.061701000	-1.430854000
H	0.261220000	0.896579000	-1.789024000
O	2.741666000	-0.842189000	0.778780000
H	-2.376868000	-0.591649000	0.190613000
H	1.487889000	0.700136000	-0.546662000
H	-0.713961000	-1.974379000	0.543476000
C	3.373164000	2.439231000	-0.193889000
C	4.254623000	4.845654000	-1.280665000
C	5.163641000	3.821063000	-1.041562000
C	4.717192000	2.626284000	-0.487667000
H	3.071741000	1.509867000	0.266287000
H	4.591006000	5.786067000	-1.696844000

H	6.212028000	3.957658000	-1.271417000
H	5.419072000	1.831289000	-0.273303000
C	2.440218000	3.455831000	-0.447158000
C	2.914461000	4.668355000	-0.972250000
H	2.212871000	5.474278000	-1.146385000
H	0.548941000	4.310896000	0.130954000
C	-3.570347000	1.852661000	0.181226000
C	-4.924030000	-0.133248000	-0.639425000
C	-4.787592000	1.051347000	0.102338000
C	-6.126984000	-0.825372000	-0.662255000
C	-5.916660000	1.518172000	0.796849000
C	-7.112753000	0.819045000	0.784827000
C	-7.222849000	-0.360267000	0.055447000
H	-6.210910000	-1.729436000	-1.250845000
H	-5.839324000	2.441205000	1.356805000
H	-7.962484000	1.197481000	1.337229000
H	-8.157371000	-0.904694000	0.036528000
H	-3.725058000	2.882295000	0.484901000
H	-4.102483000	-0.498951000	-1.235074000
O	-1.624168000	3.738093000	0.548915000

A2

C	1.060682000	-2.620608000	-0.386543000
C	2.166777000	-1.613514000	-0.286118000
H	1.029640000	5.177167000	-3.377636000

C	1.395369000	3.472072000	-0.472532000
C	1.629297000	4.424051000	-1.457318000
C	0.850102000	4.439755000	-2.606808000
C	-0.167136000	3.504127000	-2.756157000
C	-0.405789000	2.559684000	-1.765603000
C	0.379079000	2.524719000	-0.609270000
H	2.004721000	3.470996000	0.422541000
H	2.418421000	5.152193000	-1.324299000
H	-0.784584000	3.513163000	-3.644726000
H	-1.220215000	1.862856000	-1.888517000
N	0.443799000	0.076826000	0.161449000
C	1.819429000	-0.146358000	-0.270174000
C	-0.328242000	-2.097116000	-0.589041000
C	-0.487078000	-0.604292000	-0.734675000
C	-1.309138000	-3.007546000	-0.698076000
C	0.188684000	1.483393000	0.491744000
P	-1.359290000	1.765474000	1.474521000
O	-2.544164000	1.777072000	0.395946000
O	-1.683733000	0.393795000	2.251246000
C	-3.828146000	2.369661000	0.726945000
C	-4.561573000	2.666323000	-0.561413000
C	-0.955987000	0.030238000	3.450667000
C	-1.763531000	-1.001371000	4.206287000
H	-3.652377000	3.273671000	1.308815000
H	-4.380366000	1.659293000	1.344541000

H	0.010990000	-0.374313000	3.146954000
H	-0.797417000	0.927558000	4.049466000
H	-1.224571000	-1.302992000	5.105654000
H	-2.729508000	-0.594531000	4.504782000
H	-1.931989000	-1.887575000	3.595153000
H	-4.005767000	3.382098000	-1.166256000
H	-4.710379000	1.757699000	-1.144581000
H	-5.539698000	3.094039000	-0.335961000
H	1.994493000	0.294494000	-1.267137000
H	-1.500764000	-0.296967000	-0.511785000
O	-1.249386000	2.960380000	2.339178000
H	2.486498000	0.372914000	0.419778000
H	-0.297846000	-0.332931000	-1.787901000
H	0.928029000	1.728260000	1.260003000
C	-3.425800000	-3.683059000	-1.767575000
C	-4.885924000	-1.802813000	-0.351605000
C	-5.531063000	-2.633401000	-1.260930000
C	-4.794240000	-3.576882000	-1.968454000
H	-2.859358000	-4.430644000	-2.308105000
H	-5.456675000	-1.086847000	0.225095000
H	-6.600387000	-2.556961000	-1.405625000
H	-5.287604000	-4.238371000	-2.668138000
C	-2.755341000	-2.828481000	-0.881227000
C	-3.512979000	-1.893722000	-0.163683000
H	-3.035042000	-1.262250000	0.571432000

H	-0.970920000	-4.038852000	-0.676320000
C	3.416839000	-2.106453000	-0.188875000
C	4.972258000	-0.124067000	-0.503746000
C	4.708696000	-1.436975000	-0.080189000
C	6.237772000	0.428513000	-0.364465000
C	5.775397000	-2.172984000	0.463828000
C	7.034477000	-1.615295000	0.615541000
C	7.271007000	-0.307922000	0.203484000
H	6.418872000	1.438344000	-0.707984000
H	5.598971000	-3.194356000	0.775586000
H	7.834309000	-2.201996000	1.047226000
H	8.254617000	0.128694000	0.312798000
H	3.463936000	-3.189459000	-0.153406000
H	4.200002000	0.460279000	-0.978541000
O	1.284950000	-3.819077000	-0.310742000

A3

C	1.033067000	-2.645700000	-0.354131000
C	2.131586000	-1.627830000	-0.286135000
H	1.009919000	5.115747000	-3.441033000
C	-0.422391000	2.501810000	-1.820378000
C	-0.175809000	3.434558000	-2.819945000
C	0.824101000	4.387565000	-2.662928000
C	1.577796000	4.400904000	-1.496581000
C	1.336195000	3.460406000	-0.502612000
C	0.337040000	2.496298000	-0.646783000

H	-1.223548000	1.791024000	-1.949960000
H	-0.773530000	3.420869000	-3.721836000
H	2.353195000	5.142570000	-1.357683000
H	1.926195000	3.481538000	0.405066000
N	0.399928000	0.059338000	0.150385000
C	1.773345000	-0.162845000	-0.286056000
C	-0.363529000	-2.136268000	-0.537099000
C	-0.534792000	-0.648967000	-0.718992000
C	-1.341977000	-3.053356000	-0.593898000
C	0.140311000	1.466736000	0.465461000
P	-1.412368000	1.774674000	1.431322000
O	-2.570329000	1.746343000	0.321972000
O	-1.730066000	0.436010000	2.265405000
C	-3.894545000	2.247773000	0.639284000
C	-4.637854000	2.482700000	-0.656203000
C	-1.427086000	0.314572000	3.679029000
C	-0.159160000	-0.481994000	3.906658000
H	-3.788761000	3.167313000	1.214299000
H	-4.401398000	1.505295000	1.258445000
H	-1.360256000	1.313862000	4.107533000
H	-2.288393000	-0.192933000	4.111918000
H	-0.011309000	-0.629166000	4.978240000
H	-0.218953000	-1.458577000	3.428378000
H	0.714047000	0.038625000	3.514174000
H	-4.125731000	3.227013000	-1.265093000

H	-4.723537000	1.560178000	-1.230067000
H	-5.643173000	2.848127000	-0.441040000
H	1.941594000	0.265474000	-1.289835000
H	-1.548966000	-0.343783000	-0.495016000
O	-1.326043000	3.000028000	2.256299000
H	2.441524000	0.369279000	0.393127000
H	-0.358201000	-0.404338000	-1.781211000
H	0.874605000	1.721317000	1.235734000
C	-3.490028000	-3.730079000	-1.605473000
C	-4.907348000	-1.857238000	-0.136666000
C	-5.579782000	-2.684883000	-1.028740000
C	-4.864303000	-3.624428000	-1.762899000
H	-2.940148000	-4.474426000	-2.167138000
H	-5.460858000	-1.144284000	0.460385000
H	-6.653193000	-2.609178000	-1.139629000
H	-5.378622000	-4.283380000	-2.449792000
C	-2.793726000	-2.878552000	-0.736809000
C	-3.528961000	-1.947504000	0.007852000
H	-3.026355000	-1.318274000	0.728710000
H	-1.000211000	-4.082968000	-0.553572000
C	3.386908000	-2.110062000	-0.202780000
C	4.924146000	-0.119851000	-0.559526000
C	4.676303000	-1.431933000	-0.123953000
C	6.189470000	0.439683000	-0.449347000
C	5.757845000	-2.159286000	0.402345000

C	7.016963000	-1.594675000	0.524893000
C	7.238116000	-0.288465000	0.100796000
H	6.358204000	1.448649000	-0.801608000
H	5.592942000	-3.179729000	0.723318000
H	7.828528000	-2.174991000	0.943099000
H	8.221531000	0.153731000	0.187133000
H	3.442076000	-3.192176000	-0.154322000
H	4.139226000	0.458342000	-1.020683000
O	1.268460000	-3.841227000	-0.266535000

A4

C	-0.991415000	2.622987000	-0.086010000
C	-2.095709000	1.617251000	-0.202352000
H	-0.232356000	-4.692851000	-4.076557000
C	-1.142807000	-3.316632000	-1.117075000
C	-1.195912000	-4.133462000	-2.239858000
C	-0.195543000	-4.059206000	-3.200573000
C	0.858287000	-3.170851000	-3.022523000
C	0.912830000	-2.360246000	-1.895498000
C	-0.093478000	-2.415544000	-0.926866000
H	-1.923924000	-3.386951000	-0.370295000
H	-2.016451000	-4.828201000	-2.360794000
H	1.648136000	-3.114194000	-3.759985000
H	1.756253000	-1.701338000	-1.758827000
N	-0.376652000	-0.084146000	0.084820000

C	-1.721169000	0.173198000	-0.416503000
C	0.390711000	2.160052000	-0.427216000
C	0.605381000	0.687016000	-0.668083000
C	1.348798000	3.104078000	-0.470898000
C	-0.110903000	-1.509532000	0.302658000
P	1.274301000	-1.863741000	1.481953000
O	2.597498000	-1.516489000	0.644986000
O	1.276842000	-0.689446000	2.577704000
C	3.913117000	-1.725435000	1.225587000
C	4.462096000	-3.089280000	0.861039000
C	0.684459000	-0.845155000	3.889587000
C	-0.632753000	-0.102417000	3.980864000
H	4.528764000	-0.922342000	0.823728000
H	3.852868000	-1.595517000	2.306612000
H	1.412096000	-0.430257000	4.586778000
H	0.569708000	-1.907751000	4.102327000
H	-1.378390000	-0.531672000	3.311785000
H	-1.017569000	-0.163128000	5.000613000
H	-0.505792000	0.947418000	3.720097000
H	4.515518000	-3.207971000	-0.221151000
H	5.469571000	-3.198558000	1.267409000
H	3.832936000	-3.878685000	1.268461000
H	-1.808466000	-0.098989000	-1.483405000
H	0.544622000	0.464246000	-1.748050000
O	1.181954000	-3.225512000	2.054452000

H	-2.423524000	-0.462750000	0.123805000
H	1.603297000	0.408141000	-0.341126000
H	-0.945905000	-1.859168000	0.916948000
C	3.337311000	2.012020000	-1.602394000
C	4.988035000	3.970087000	-0.523012000
C	5.533812000	2.966797000	-1.317125000
C	4.700791000	1.996118000	-1.862028000
H	2.708050000	1.268915000	-2.067246000
H	5.624557000	4.738562000	-0.105059000
H	6.595944000	2.950177000	-1.521407000
H	5.113162000	1.226982000	-2.501726000
C	2.769999000	3.005887000	-0.789723000
C	3.624926000	3.994522000	-0.275058000
H	3.203529000	4.783746000	0.334135000
H	1.011080000	4.096349000	-0.191722000
C	-3.352630000	2.082235000	-0.075665000
C	-4.863011000	0.187198000	-0.828580000
C	-4.637818000	1.393965000	-0.146847000
C	-6.121937000	-0.396969000	-0.845915000
C	-5.735204000	1.998050000	0.489742000
C	-6.988371000	1.407199000	0.483132000
C	-7.187021000	0.202562000	-0.183587000
H	-6.273111000	-1.321534000	-1.387158000
H	-5.588355000	2.940521000	1.001264000
H	-7.813112000	1.889385000	0.990749000

H	-8.165758000	-0.257781000	-0.197908000
H	-3.415002000	3.141213000	0.150675000
H	-4.064051000	-0.280866000	-1.382148000
O	-1.216982000	3.780324000	0.234210000

A5

C	1.049495000	2.943556000	0.042486000
C	2.128469000	1.901603000	0.066220000
H	1.245651000	-4.259971000	4.144071000
C	-0.314979000	-1.930503000	2.233347000
C	0.005041000	-2.696178000	3.347122000
C	1.001949000	-3.662923000	3.275545000
C	1.680045000	-3.856546000	2.079367000
C	1.364804000	-3.081454000	0.969534000
C	0.365322000	-2.108648000	1.025276000
H	-1.111033000	-1.205787000	2.302844000
H	-0.532213000	-2.540864000	4.273422000
H	2.456090000	-4.607213000	2.008494000
H	1.904684000	-3.235484000	0.043336000
N	0.373615000	0.183641000	-0.112758000
C	1.752105000	0.456040000	0.274716000
C	-0.358805000	2.470710000	0.217649000
C	-0.555169000	1.032167000	0.625556000
C	-1.334547000	3.374326000	0.042388000
C	0.099722000	-1.251088000	-0.210935000

P	-1.518068000	-1.593285000	-1.050599000
O	-1.370341000	-3.124586000	-1.527557000
O	-2.557389000	-1.701982000	0.164968000
C	-1.320257000	-3.500360000	-2.926708000
C	-0.025036000	-4.226834000	-3.223312000
C	-3.938169000	-2.063244000	-0.084598000
C	-4.699623000	-1.960655000	1.217487000
H	-2.178237000	-4.150739000	-3.099485000
H	-1.435847000	-2.607041000	-3.538665000
H	-4.345834000	-1.390171000	-0.839505000
H	-3.959429000	-3.081714000	-0.475913000
H	-5.740097000	-2.247710000	1.058167000
H	-4.271801000	-2.623507000	1.969149000
H	-4.680848000	-0.940195000	1.598557000
H	0.093529000	-5.092090000	-2.571439000
H	-0.026469000	-4.573399000	-4.258190000
H	0.835320000	-3.570695000	-3.089252000
H	1.927318000	0.174941000	1.328416000
H	-0.392601000	0.955503000	1.715539000
O	-1.892409000	-0.658812000	-2.136182000
H	2.408487000	-0.176160000	-0.325675000
H	-1.575409000	0.717104000	0.438807000
H	0.789416000	-1.613397000	-0.980159000
C	-3.578174000	4.123298000	0.766785000
C	-4.823749000	2.071445000	-0.617007000

C	-5.589793000	2.980399000	0.104192000
C	-4.960636000	4.009463000	0.796640000
H	-3.093736000	4.936032000	1.292982000
H	-5.307076000	1.288512000	-1.186800000
H	-6.668603000	2.898594000	0.113034000
H	-5.547676000	4.731065000	1.349313000
C	-2.792883000	3.193442000	0.072676000
C	-3.437917000	2.169628000	-0.633194000
H	-2.861363000	1.473386000	-1.227758000
H	-0.990762000	4.388259000	-0.137683000
C	3.385463000	2.340186000	-0.137213000
C	4.911020000	0.393064000	0.436347000
C	4.655732000	1.623160000	-0.191068000
C	6.155070000	-0.214032000	0.333114000
C	5.709632000	2.224955000	-0.899640000
C	6.946299000	1.611156000	-1.014125000
C	7.173931000	0.384194000	-0.399204000
H	6.331350000	-1.156352000	0.834795000
H	5.540300000	3.184604000	-1.370698000
H	7.735922000	2.092313000	-1.575677000
H	8.140587000	-0.094742000	-0.479093000
H	3.457636000	3.405358000	-0.328710000
H	4.151309000	-0.076406000	1.041360000
O	1.311512000	4.124103000	-0.128222000

A6

C	0.916545000	-2.599563000	-0.070441000
C	2.051140000	-1.632263000	-0.212884000
H	0.288853000	4.771483000	-4.004093000
C	-0.871306000	2.453305000	-1.815623000
C	-0.820695000	3.272900000	-2.936299000
C	0.254932000	4.130710000	-3.133200000
C	1.280902000	4.165663000	-2.197649000
C	1.231380000	3.339936000	-1.081246000
C	0.160342000	2.469009000	-0.872589000
H	-1.730497000	1.818624000	-1.663330000
H	-1.630270000	3.247361000	-3.653841000
H	2.118638000	4.836813000	-2.333056000
H	2.032793000	3.379227000	-0.353971000
N	0.393895000	0.120197000	0.111199000
C	1.717861000	-0.177723000	-0.422062000
C	-0.453546000	-2.094927000	-0.402298000
C	-0.629760000	-0.613045000	-0.623733000
C	-1.434128000	-3.014739000	-0.459699000
C	0.181096000	1.551026000	0.347783000
P	-1.156064000	1.942569000	1.569881000
O	-2.520765000	1.673436000	0.768264000
O	-1.177699000	0.749530000	2.644630000
C	-3.788783000	2.006244000	1.392712000
C	-4.877156000	1.907445000	0.348152000

C	-0.557562000	0.861846000	3.948166000
C	0.736960000	0.076843000	4.002731000
H	-3.960475000	1.304784000	2.210561000
H	-3.718460000	3.013585000	1.803186000
H	-0.405406000	1.916248000	4.177393000
H	-1.285901000	0.457465000	4.650695000
H	1.484465000	0.495533000	3.329020000
H	1.140523000	0.106403000	5.016606000
H	0.572849000	-0.963495000	3.725140000
H	-4.932010000	0.900198000	-0.063941000
H	-5.840898000	2.145446000	0.800958000
H	-4.699036000	2.610992000	-0.464935000
H	1.788208000	0.091249000	-1.490931000
H	-0.589291000	-0.380551000	-1.702467000
O	-0.992161000	3.291011000	2.156617000
H	2.451894000	0.436606000	0.100597000
H	-1.611355000	-0.308367000	-0.269325000
H	1.043688000	1.866984000	0.941857000
C	-3.390207000	-1.866168000	-1.590216000
C	-5.089249000	-3.806928000	-0.554792000
C	-5.607033000	-2.784381000	-1.342984000
C	-4.749962000	-1.822480000	-1.865955000
H	-2.741899000	-1.127689000	-2.035868000
H	-5.744732000	-4.568223000	-0.153352000
H	-6.666137000	-2.745641000	-1.559552000

H	-5.140749000	-1.038619000	-2.501263000
C	-2.850703000	-2.881730000	-0.785883000
C	-3.729632000	-3.860305000	-0.292774000
H	-3.329618000	-4.665336000	0.310111000
H	-1.120529000	-4.017914000	-0.191577000
C	3.294161000	-2.138983000	-0.112113000
C	5.690261000	-2.130739000	0.400853000
C	4.599475000	-1.494076000	-0.214905000
C	6.961544000	-1.581134000	0.363781000
C	4.848799000	-0.298631000	-0.907946000
C	6.125312000	0.244151000	-0.955717000
C	7.184596000	-0.386712000	-0.313514000
H	7.781108000	-2.087687000	0.855813000
H	4.053572000	0.192726000	-1.446570000
H	6.294551000	1.160733000	-1.505124000
H	8.177254000	0.041320000	-0.351655000
H	3.325983000	-3.198559000	0.117706000
H	5.524058000	-3.065474000	0.920593000
O	1.108002000	-3.760656000	0.257997000

A7

C	0.981217000	-2.575175000	-0.215002000
C	2.116229000	-1.598175000	-0.247338000
H	0.689458000	5.112947000	-3.559628000
C	1.371047000	3.436121000	-0.695611000

C	1.515892000	4.355393000	-1.727121000
C	0.581430000	4.399611000	-2.753604000
C	-0.499131000	3.525468000	-2.732510000
C	-0.646002000	2.612327000	-1.695523000
C	0.293655000	2.548724000	-0.662538000
H	2.101582000	3.414899000	0.103331000
H	2.356177000	5.036927000	-1.725869000
H	-1.237947000	3.559858000	-3.522381000
H	-1.509678000	1.965695000	-1.677691000
N	0.438971000	0.123141000	0.148517000
C	1.786264000	-0.131397000	-0.347486000
C	-0.380517000	-2.049131000	-0.548576000
C	-0.549253000	-0.556142000	-0.681392000
C	-1.360541000	-2.961167000	-0.684766000
C	0.209970000	1.534583000	0.476414000
P	-1.228334000	1.824091000	1.610531000
O	-2.525708000	1.732653000	0.673590000
O	-1.405119000	0.490776000	2.489988000
C	-3.826165000	2.097392000	1.208333000
C	-4.785368000	2.267101000	0.052393000
C	-0.552731000	0.229523000	3.629533000
C	-1.222466000	-0.815553000	4.493763000
H	-4.151459000	1.302185000	1.880373000
H	-3.719733000	3.019162000	1.779967000
H	0.408730000	-0.130984000	3.258746000

H	-0.397930000	1.161142000	4.174634000
H	-0.586833000	-1.050623000	5.348833000
H	-2.180565000	-0.453731000	4.866464000
H	-1.391879000	-1.731926000	3.929181000
H	-4.873319000	1.342882000	-0.518488000
H	-5.773772000	2.530096000	0.432255000
H	-4.451926000	3.062094000	-0.614240000
H	1.903087000	0.225265000	-1.385923000
H	-0.457252000	-0.253401000	-1.739111000
O	-1.066666000	3.069200000	2.392080000
H	2.493548000	0.437924000	0.256965000
H	-1.546143000	-0.272811000	-0.353396000
H	1.020310000	1.795890000	1.163070000
C	-3.286340000	-1.744745000	-1.795300000
C	-5.016561000	-3.725788000	-0.896424000
C	-5.512341000	-2.660677000	-1.641141000
C	-4.639785000	-1.678392000	-2.096128000
H	-2.624634000	-0.989024000	-2.189398000
H	-5.684324000	-4.502523000	-0.548397000
H	-6.566515000	-2.604207000	-1.877015000
H	-5.013346000	-0.860937000	-2.698632000
C	-2.769318000	-2.803648000	-1.033454000
C	-3.662977000	-3.801104000	-0.609722000
H	-3.279581000	-4.638371000	-0.040918000
H	-1.054464000	-3.979808000	-0.471749000

C	3.357681000	-2.111589000	-0.157559000
C	4.930792000	-0.201960000	-0.726395000
C	4.665456000	-1.464194000	-0.170881000
C	6.209935000	0.335556000	-0.693734000
C	5.743447000	-2.168865000	0.391468000
C	7.017241000	-1.625736000	0.435040000
C	7.255869000	-0.366575000	-0.105800000
H	6.391844000	1.305673000	-1.136898000
H	5.564539000	-3.153381000	0.804155000
H	7.826369000	-2.186741000	0.883246000
H	8.250383000	0.058129000	-0.080839000
H	3.384886000	-3.186952000	-0.018796000
H	4.146965000	0.350579000	-1.220250000
O	1.166973000	-3.757628000	0.030240000

A8

C	1.211830000	2.906769000	-0.039234000
C	2.245147000	1.819593000	-0.006020000
H	1.208453000	-4.268128000	4.132988000
C	-0.293026000	-1.877248000	2.251117000
C	0.024182000	-2.648936000	3.361458000
C	0.966947000	-3.666683000	3.266891000
C	1.593716000	-3.905453000	2.051125000
C	1.281951000	-3.124209000	0.944551000
C	0.336728000	-2.100288000	1.023257000

H	-1.048334000	-1.111925000	2.339505000
H	-0.473146000	-2.458200000	4.303290000
H	2.326969000	-4.696149000	1.962288000
H	1.783259000	-3.313407000	0.003051000
N	0.413125000	0.179582000	-0.137059000
C	1.808373000	0.394505000	0.224117000
C	-0.212727000	2.501193000	0.169847000
C	-0.465245000	1.078671000	0.602888000
C	-1.148914000	3.447325000	0.002247000
C	0.072430000	-1.241283000	-0.212209000
P	-1.583796000	-1.526129000	-0.996843000
O	-1.513683000	-3.058213000	-1.493214000
O	-2.585982000	-1.635673000	0.245620000
C	-1.147200000	-3.385298000	-2.855248000
C	-1.694617000	-4.757341000	-3.180484000
C	-3.991799000	-1.921328000	0.033503000
C	-4.714585000	-1.750076000	1.350220000
H	-1.545960000	-2.620969000	-3.520929000
H	-0.057039000	-3.374434000	-2.930928000
H	-4.377317000	-1.239983000	-0.725468000
H	-4.078412000	-2.943568000	-0.336983000
H	-5.772711000	-1.982365000	1.220649000
H	-4.306200000	-2.420491000	2.106018000
H	-4.630828000	-0.724757000	1.708926000
H	-2.782873000	-4.763474000	-3.122647000

H	-1.403272000	-5.040735000	-4.193013000
H	-1.307370000	-5.504995000	-2.488587000
H	1.989409000	0.116276000	1.277580000
H	-0.289023000	1.010343000	1.691247000
O	-1.964001000	-0.566546000	-2.057665000
H	2.425589000	-0.272064000	-0.380757000
H	-1.500867000	0.805887000	0.435345000
H	0.724397000	-1.641058000	-0.997182000
C	-3.331062000	4.330218000	0.755791000
C	-4.716206000	2.308073000	-0.535656000
C	-5.415254000	3.275613000	0.177448000
C	-4.716220000	4.289787000	0.823228000
H	-2.791501000	5.131295000	1.244975000
H	-5.254040000	1.535800000	-1.070028000
H	-6.496254000	3.250402000	0.215352000
H	-5.250745000	5.056580000	1.368278000
C	-2.613013000	3.341136000	0.070438000
C	-3.328054000	2.333229000	-0.589392000
H	-2.804454000	1.592114000	-1.178649000
H	-0.760767000	4.440638000	-0.200903000
C	3.518903000	2.201929000	-0.219054000
C	4.963188000	0.192204000	0.355317000
C	4.759092000	1.434064000	-0.268202000
C	6.183130000	-0.462446000	0.255518000
C	5.839254000	1.996274000	-0.969998000

C	7.051607000	1.335319000	-1.080807000
C	7.228342000	0.098300000	-0.469363000
H	6.319904000	-1.412881000	0.754215000
H	5.709876000	2.963360000	-1.438447000
H	7.862083000	1.787421000	-1.636772000
H	8.176017000	-0.417606000	-0.546323000
H	3.634016000	3.260792000	-0.423928000
H	4.182939000	-0.250693000	0.954039000
O	1.522650000	4.070454000	-0.241480000

A9

C	1.149300000	2.926391000	0.033134000
C	2.187109000	1.842590000	0.045033000
H	1.215960000	-4.346327000	4.028941000
C	1.245764000	-3.132118000	0.865735000
C	1.572011000	-3.937853000	1.950327000
C	0.962985000	-3.725473000	3.179978000
C	0.023433000	-2.708806000	3.310636000
C	-0.308493000	-1.912384000	2.222228000
C	0.302932000	-2.109315000	0.980715000
H	1.732140000	-3.301214000	-0.087109000
H	2.302766000	-4.727257000	1.833605000
H	-0.459667000	-2.538025000	4.263649000
H	-1.060658000	-1.147715000	2.338177000
N	0.358507000	0.198708000	-0.124244000

C	1.756295000	0.408726000	0.230144000
C	-0.272878000	2.509649000	0.233585000
C	-0.514246000	1.079292000	0.645146000
C	-1.216100000	3.450446000	0.076141000
C	0.023079000	-1.222571000	-0.231578000
P	-1.643008000	-1.485719000	-1.001669000
O	-1.597506000	-3.018452000	-1.499584000
O	-2.624368000	-1.557415000	0.261056000
C	-1.593672000	-3.373079000	-2.905482000
C	-0.227317000	-3.866023000	-3.335960000
C	-4.039361000	-1.830139000	0.091020000
C	-4.416303000	-3.069009000	0.874505000
H	-2.340361000	-4.159932000	-3.009576000
H	-1.910420000	-2.509186000	-3.487864000
H	-4.567368000	-0.949287000	0.454160000
H	-4.266271000	-1.943005000	-0.969601000
H	-5.489022000	-3.250105000	0.785843000
H	-4.175574000	-2.946114000	1.930119000
H	-3.883969000	-3.941950000	0.498851000
H	0.103116000	-4.694257000	-2.709219000
H	-0.271542000	-4.213999000	-4.369486000
H	0.515504000	-3.069526000	-3.284040000
H	1.948708000	0.096649000	1.271949000
H	-1.551226000	0.805595000	0.489394000
O	-2.022834000	-0.528277000	-2.065352000

H	2.370483000	-0.234910000	-0.402309000
H	-0.321543000	0.992108000	1.729277000
H	0.665382000	-1.600145000	-1.033768000
C	-3.410537000	4.297887000	0.837665000
C	-4.769790000	2.287183000	-0.498771000
C	-5.481598000	3.233838000	0.229547000
C	-4.795540000	4.242227000	0.897983000
H	-2.881344000	5.094571000	1.345011000
H	-5.296495000	1.519287000	-1.050251000
H	-6.562463000	3.197201000	0.262220000
H	-5.340092000	4.992800000	1.455553000
C	-2.679610000	3.329462000	0.136637000
C	-3.381684000	2.327296000	-0.545522000
H	-2.848921000	1.602000000	-1.146108000
H	-0.835870000	4.449779000	-0.112090000
C	3.461397000	2.237445000	-0.141386000
C	4.909630000	0.212339000	0.370077000
C	4.707893000	1.480765000	-0.198660000
C	6.137179000	-0.427173000	0.266161000
C	5.797889000	2.083161000	-0.850444000
C	7.018244000	1.437933000	-0.965456000
C	7.192828000	0.175201000	-0.408489000
H	6.271450000	-1.399270000	0.721945000
H	5.669824000	3.070057000	-1.275978000
H	7.836335000	1.921976000	-1.481971000

H	8.146399000	-0.329219000	-0.488754000
H	3.572218000	3.302841000	-0.311818000
H	4.120773000	-0.265551000	0.929016000
O	1.454993000	4.095348000	-0.144985000

A10

C	1.120613000	-2.649999000	0.117758000
C	2.165189000	-1.588115000	-0.075107000
H	-0.777780000	3.532651000	-5.043970000
C	-1.497745000	1.893813000	-2.162032000
C	-1.668742000	2.419143000	-3.436914000
C	-0.640648000	3.124438000	-4.051558000
C	0.560592000	3.309793000	-3.378813000
C	0.729886000	2.784263000	-2.103549000
C	-0.290448000	2.063789000	-1.479077000
H	-2.314457000	1.367590000	-1.687861000
H	-2.611508000	2.280098000	-3.949261000
H	1.363894000	3.866754000	-3.842714000
H	1.664999000	2.945109000	-1.581263000
N	0.344573000	0.046569000	-0.040415000
C	1.726203000	-0.192562000	-0.442639000
C	-0.292321000	-2.292471000	-0.219517000
C	-0.545219000	-0.894908000	-0.724548000
C	-1.204428000	-3.275881000	-0.114515000
C	-0.039254000	1.468812000	-0.100030000

P	-1.395227000	1.732864000	1.122550000
O	-0.728872000	1.471452000	2.559101000
O	-1.504346000	3.326797000	1.090999000
C	-0.872358000	0.209277000	3.261168000
C	-0.561158000	0.443218000	4.722390000
C	-2.546294000	4.016228000	1.823860000
C	-2.556871000	5.461850000	1.381071000
H	-1.889261000	-0.154844000	3.120394000
H	-0.181071000	-0.505001000	2.815245000
H	-3.499583000	3.527863000	1.619680000
H	-2.331231000	3.929725000	2.890348000
H	-3.327652000	6.008881000	1.926070000
H	-1.595028000	5.934905000	1.577810000
H	-2.768041000	5.537866000	0.314928000
H	-1.260108000	1.154694000	5.162309000
H	-0.639827000	-0.498101000	5.268336000
H	0.450451000	0.828709000	4.848479000
H	2.367739000	0.530188000	0.063481000
H	-0.378743000	-0.869284000	-1.815025000
O	-2.655552000	0.979941000	0.893580000
H	1.850889000	-0.027342000	-1.527460000
H	-1.569777000	-0.597014000	-0.553619000
H	0.792683000	2.016864000	0.346056000
C	-3.525272000	-2.236935000	-0.111491000
C	-4.546376000	-4.618154000	-1.130849000

C	-5.399987000	-3.548259000	-0.885882000
C	-4.884087000	-2.366471000	-0.365447000
H	-3.169020000	-1.314815000	0.323791000
H	-4.937190000	-5.548564000	-1.521063000
H	-6.459476000	-3.639570000	-1.085134000
H	-5.542688000	-1.536520000	-0.146488000
C	-2.647962000	-3.300532000	-0.371113000
C	-3.191669000	-4.498575000	-0.861025000
H	-2.532940000	-5.339149000	-1.038731000
H	-0.784512000	-4.234914000	0.171965000
C	3.442251000	-1.961158000	0.131593000
C	4.873801000	-0.066532000	-0.762996000
C	4.688908000	-1.208667000	0.032303000
C	6.099584000	0.583403000	-0.802989000
C	5.793354000	-1.681239000	0.761282000
C	7.012423000	-1.023420000	0.732099000
C	7.170396000	0.116308000	-0.049661000
H	6.221145000	1.454913000	-1.432584000
H	5.678732000	-2.574515000	1.361670000
H	7.842858000	-1.403722000	1.311835000
H	8.123023000	0.627695000	-0.081818000
H	3.556174000	-2.988540000	0.460122000
H	4.071534000	0.295223000	-1.386865000
O	1.422269000	-3.761883000	0.525490000

A11

C	0.760901000	-2.751190000	0.126021000
C	1.900493000	-1.799779000	-0.103127000
H	-0.670025000	3.634320000	-4.947310000
C	-1.464226000	2.071829000	-2.042711000
C	-1.622474000	2.623480000	-3.307839000
C	-0.542586000	3.204278000	-3.962915000
C	0.699350000	3.235907000	-3.341535000
C	0.856115000	2.683018000	-2.075782000
C	-0.218429000	2.088190000	-1.409783000
H	-2.318930000	1.644038000	-1.537263000
H	-2.595649000	2.604319000	-3.779998000
H	1.545258000	3.692306000	-3.838393000
H	1.827035000	2.719239000	-1.596400000
N	0.252951000	0.006566000	-0.006010000
C	1.590828000	-0.365283000	-0.451299000
C	-0.616607000	-2.257140000	-0.180065000
C	-0.747550000	-0.835652000	-0.664809000
C	-1.616065000	-3.151241000	-0.072429000
C	0.010649000	1.457593000	-0.041449000
P	-1.289779000	1.868044000	1.210376000
O	-0.514062000	1.631688000	2.592857000
O	-1.489485000	3.455868000	1.128863000
C	-1.039770000	0.806628000	3.666596000
C	-0.011424000	-0.232137000	4.055515000

C	-0.468304000	4.426372000	1.444555000
C	-1.133273000	5.776091000	1.604298000
H	-1.259914000	1.479543000	4.495937000
H	-1.970545000	0.350206000	3.334605000
H	0.040796000	4.125084000	2.360771000
H	0.258850000	4.447785000	0.629666000
H	-0.382228000	6.534982000	1.829063000
H	-1.650054000	6.061479000	0.688728000
H	-1.857997000	5.754402000	2.417524000
H	0.921167000	0.239390000	4.365953000
H	-0.388427000	-0.826732000	4.889511000
H	0.197770000	-0.901148000	3.222170000
H	1.700758000	-0.205994000	-1.538879000
H	-0.618241000	-0.814168000	-1.760705000
O	-2.580694000	1.157858000	1.064213000
H	2.316241000	0.285814000	0.038897000
H	-1.731031000	-0.442604000	-0.452690000
H	0.912024000	1.909396000	0.379451000
C	-3.723153000	-4.152985000	-0.823868000
C	-5.192372000	-1.883665000	-0.231706000
C	-5.828225000	-2.991817000	-0.780659000
C	-5.086717000	-4.130898000	-1.074212000
H	-3.152200000	-5.047327000	-1.039237000
H	-5.763530000	-1.001440000	0.025141000
H	-6.894278000	-2.973247000	-0.964402000

H	-5.572166000	-5.005193000	-1.487329000
C	-3.058778000	-3.030085000	-0.305915000
C	-3.823879000	-1.895460000	0.002501000
H	-3.371170000	-1.028170000	0.460420000
H	-1.286564000	-4.150533000	0.193854000
C	3.139658000	-2.302465000	0.052936000
C	4.721537000	-0.553828000	-0.886056000
C	4.452253000	-1.680961000	-0.093330000
C	6.004954000	-0.032262000	-0.971706000
C	5.531327000	-2.273297000	0.584405000
C	6.809483000	-1.743823000	0.509413000
C	7.052105000	-0.616330000	-0.268388000
H	6.189713000	0.829965000	-1.598787000
H	5.350244000	-3.157296000	1.182025000
H	7.619397000	-2.215037000	1.050131000
H	8.050310000	-0.205223000	-0.336502000
H	3.160073000	-3.338099000	0.374631000
H	3.935853000	-0.103550000	-1.472245000
O	0.961559000	-3.885620000	0.533836000

A12

C	0.754439000	-2.813165000	0.169632000
C	1.890547000	-1.882189000	-0.132544000
H	-1.415964000	3.066639000	-5.193030000
C	-1.695037000	1.925912000	-2.009572000

C	-2.074627000	2.342255000	-3.280109000
C	-1.117307000	2.741873000	-4.205277000
C	0.225582000	2.730241000	-3.849332000
C	0.603667000	2.316300000	-2.577975000
C	-0.346866000	1.902856000	-1.642268000
H	-2.450416000	1.639906000	-1.290352000
H	-3.123629000	2.359931000	-3.544910000
H	0.979057000	3.048214000	-4.557738000
H	1.652116000	2.322645000	-2.307037000
N	0.309622000	-0.038532000	-0.112355000
C	1.537497000	-0.542976000	-0.723950000
C	-0.631075000	-2.308143000	-0.090308000
C	-0.827570000	-0.870469000	-0.506762000
C	-1.625983000	-3.202870000	0.077767000
C	0.122602000	1.416408000	-0.276927000
P	-0.874716000	2.027575000	1.149585000
O	0.025009000	1.737902000	2.448829000
O	-0.690367000	3.605753000	0.964047000
C	-0.201786000	0.576414000	3.289326000
C	0.380703000	0.858229000	4.656141000
C	-1.429220000	4.540915000	1.786547000
C	-1.329347000	5.909625000	1.151385000
H	-1.273128000	0.386277000	3.339970000
H	0.282302000	-0.277490000	2.816181000
H	-2.464658000	4.206125000	1.855346000

H	-0.993585000	4.535316000	2.787240000
H	-1.764432000	5.904151000	0.152539000
H	-1.867070000	6.638992000	1.759076000
H	-0.289410000	6.226329000	1.073889000
H	-0.113480000	1.710068000	5.123508000
H	0.245051000	-0.012706000	5.299200000
H	1.447616000	1.071183000	4.589593000
H	1.424074000	-0.648897000	-1.817498000
H	-0.981905000	-0.819518000	-1.597788000
O	-2.268421000	1.529123000	1.253509000
H	2.337380000	0.172421000	-0.565556000
H	-1.725071000	-0.474913000	-0.034572000
H	1.101511000	1.861147000	-0.091297000
C	-3.720061000	-2.113978000	-0.863446000
C	-5.247403000	-4.053184000	0.417480000
C	-5.873086000	-3.062561000	-0.332231000
C	-5.102616000	-2.101699000	-0.976827000
H	-3.149575000	-1.371144000	-1.396679000
H	-5.836305000	-4.812468000	0.914506000
H	-6.951045000	-3.045164000	-0.421044000
H	-5.580934000	-1.337799000	-1.575136000
C	-3.069089000	-3.096255000	-0.099340000
C	-3.866361000	-4.073762000	0.521481000
H	-3.382669000	-4.851806000	1.097881000
H	-1.289481000	-4.172111000	0.429522000

C	3.133161000	-2.335285000	0.093058000
C	5.480492000	-2.349265000	-0.681687000
C	4.424696000	-1.654061000	-0.077435000
C	6.723966000	-1.756982000	-0.850055000
C	4.673868000	-0.359828000	0.396268000
C	5.922998000	0.226561000	0.242794000
C	6.949872000	-0.464286000	-0.390593000
H	7.520382000	-2.308795000	-1.331450000
H	3.896636000	0.174971000	0.924243000
H	6.097327000	1.222551000	0.628015000
H	7.921557000	-0.004767000	-0.511736000
H	3.188066000	-3.367601000	0.424332000
H	5.314769000	-3.361003000	-1.029046000
O	0.958576000	-3.935825000	0.606252000

A13

C	0.927975000	-2.755983000	0.126883000
C	2.023563000	-1.742278000	-0.052608000
H	0.066293000	3.826673000	-4.855741000
C	-1.114305000	1.965426000	-2.275185000
C	-1.065824000	2.580983000	-3.520385000
C	0.034127000	3.347973000	-3.886133000
C	1.088362000	3.502261000	-2.994422000
C	1.040159000	2.884691000	-1.750756000
C	-0.054857000	2.104172000	-1.373842000

H	-1.990929000	1.402003000	-1.991321000
H	-1.896243000	2.465087000	-4.204179000
H	1.945902000	4.104581000	-3.263883000
H	1.863541000	3.016781000	-1.059275000
N	0.281922000	-0.029322000	-0.005141000
C	1.652194000	-0.323709000	-0.406510000
C	-0.465198000	-2.329529000	-0.210050000
C	-0.646183000	-0.918240000	-0.707207000
C	-1.425198000	-3.266361000	-0.106378000
C	-0.031759000	1.405156000	-0.017779000
P	-1.554231000	1.719132000	0.987384000
O	-1.352670000	0.981930000	2.394365000
O	-1.365314000	3.289937000	1.271232000
C	-0.168831000	1.098776000	3.212593000
C	-0.475107000	0.510074000	4.572169000
C	-2.496725000	4.126508000	1.624110000
C	-2.132707000	5.564881000	1.332648000
H	0.637891000	0.550922000	2.724035000
H	0.111541000	2.151254000	3.293770000
H	-3.362257000	3.803617000	1.046769000
H	-2.714291000	3.979985000	2.683768000
H	-2.962905000	6.217484000	1.607177000
H	-1.254758000	5.868914000	1.902637000
H	-1.921906000	5.702551000	0.272656000
H	-0.765341000	-0.535688000	4.479324000

H	0.409444000	0.567806000	5.208259000
H	-1.286181000	1.052481000	5.057414000
H	1.789969000	-0.149417000	-1.487879000
H	-0.466268000	-0.892093000	-1.795861000
O	-2.853812000	1.329034000	0.394125000
H	2.324985000	0.364256000	0.108723000
H	-1.658957000	-0.572766000	-0.552899000
H	0.758992000	1.883176000	0.566075000
C	-3.694924000	-2.113839000	-0.130761000
C	-4.828521000	-4.469458000	-1.086831000
C	-5.629978000	-3.353726000	-0.871858000
C	-5.058050000	-2.184315000	-0.383100000
H	-3.299015000	-1.196419000	0.277790000
H	-5.263312000	-5.390399000	-1.452230000
H	-6.692582000	-3.399368000	-1.070220000
H	-5.674300000	-1.316670000	-0.189215000
C	-2.868398000	-3.224239000	-0.359978000
C	-3.469795000	-4.407362000	-0.818866000
H	-2.852007000	-5.282957000	-0.973002000
H	-1.051936000	-4.244598000	0.179495000
C	3.280728000	-2.177356000	0.153848000
C	4.802058000	-0.339439000	-0.711707000
C	4.561775000	-1.482891000	0.066896000
C	6.056912000	0.253309000	-0.739556000
C	5.641193000	-2.016704000	0.791183000

C	6.889737000	-1.416242000	0.773768000
C	7.102880000	-0.273944000	0.008967000
H	6.220502000	1.126715000	-1.356901000
H	5.483772000	-2.912102000	1.378497000
H	7.700108000	-1.843494000	1.349216000
H	8.078567000	0.192492000	-0.014301000
H	3.344639000	-3.212448000	0.471607000
H	4.019370000	0.068370000	-1.332026000
O	1.177529000	-3.884653000	0.523032000

A14

C	0.731929000	-2.781793000	0.235902000
C	1.876800000	-1.849267000	-0.023719000
H	0.098095000	3.678903000	-4.900120000
C	-1.114017000	2.030188000	-2.192490000
C	-1.077137000	2.602943000	-3.458572000
C	0.074542000	3.233304000	-3.914597000
C	1.192135000	3.296434000	-3.091376000
C	1.154929000	2.722580000	-1.826629000
C	0.008185000	2.075857000	-1.360110000
H	-2.027173000	1.579115000	-1.833225000
H	-1.956751000	2.562612000	-4.087371000
H	2.090849000	3.794394000	-3.430661000
H	2.028627000	2.784008000	-1.189283000
N	0.246824000	-0.043621000	0.046323000

C	1.557474000	-0.442745000	-0.455999000
C	-0.632656000	-2.309459000	-0.151807000
C	-0.806218000	-0.865038000	-0.548489000
C	-1.616230000	-3.229770000	-0.109412000
C	0.041719000	1.412621000	0.014364000
P	-1.397661000	1.890682000	1.077758000
O	-1.227552000	1.118638000	2.469752000
O	-1.005176000	3.425260000	1.365233000
C	-0.017485000	1.122956000	3.257725000
C	-0.341533000	0.554852000	4.622012000
C	-2.021635000	4.395583000	1.724393000
C	-1.512301000	5.774115000	1.368127000
H	0.724627000	0.508976000	2.745982000
H	0.357075000	2.145741000	3.336028000
H	-2.939040000	4.152617000	1.189643000
H	-2.213185000	4.309037000	2.795608000
H	-2.253103000	6.524248000	1.649381000
H	-0.583566000	5.996004000	1.893955000
H	-1.330901000	5.853697000	0.296784000
H	-0.726321000	-0.460242000	4.532137000
H	0.560017000	0.529222000	5.236028000
H	-1.088932000	1.164276000	5.129457000
H	1.609058000	-0.349799000	-1.554707000
H	-0.806428000	-0.769640000	-1.648115000
O	-2.753347000	1.674048000	0.528048000

H	2.307284000	0.238037000	-0.051483000
H	-1.772309000	-0.505567000	-0.199179000
H	0.897311000	1.837778000	0.544961000
C	-3.626931000	-2.161199000	-1.237153000
C	-5.233031000	-4.161844000	-0.165208000
C	-5.801355000	-3.163928000	-0.950018000
C	-4.990988000	-2.171401000	-1.489031000
H	-3.022692000	-1.392532000	-1.691041000
H	-5.852328000	-4.945019000	0.251339000
H	-6.865050000	-3.164509000	-1.146823000
H	-5.422841000	-1.398937000	-2.111046000
C	-3.035282000	-3.149758000	-0.434384000
C	-3.869533000	-4.158250000	0.078575000
H	-3.430059000	-4.940676000	0.683759000
H	-1.295062000	-4.198545000	0.257586000
C	3.114387000	-2.338857000	0.175487000
C	4.694658000	-0.651772000	-0.868747000
C	4.424560000	-1.717816000	0.004065000
C	5.973313000	-0.121400000	-0.971373000
C	5.497479000	-2.244050000	0.742921000
C	6.770691000	-1.705427000	0.649479000
C	7.014001000	-0.636849000	-0.207329000
H	6.159578000	0.692710000	-1.659297000
H	5.315419000	-3.082713000	1.402504000
H	7.576353000	-2.123988000	1.237783000

H	8.008525000	-0.219422000	-0.289523000
H	3.135420000	-3.353404000	0.558642000
H	3.914190000	-0.258534000	-1.501695000
O	0.918280000	-3.893897000	0.707183000

A15

C	1.100437000	-2.629371000	0.056743000
C	2.137632000	-1.559411000	-0.132932000
H	-0.969079000	3.635063000	-4.924659000
C	-1.600877000	1.956868000	-2.044774000
C	-1.812025000	2.502684000	-3.304909000
C	-0.800821000	3.210861000	-3.943818000
C	0.423938000	3.378378000	-3.309946000
C	0.633284000	2.832457000	-2.049271000
C	-0.369836000	2.108721000	-1.401137000
H	-2.403932000	1.427585000	-1.550871000
H	-2.772647000	2.377198000	-3.786686000
H	1.214598000	3.937442000	-3.792666000
H	1.586790000	2.979946000	-1.557274000
N	0.313543000	0.068058000	-0.021306000
C	1.686669000	-0.157386000	-0.459208000
C	-0.320578000	-2.268171000	-0.240663000
C	-0.586798000	-0.860647000	-0.709775000
C	-1.228064000	-3.255975000	-0.136712000
C	-0.075182000	1.489907000	-0.041509000

P	-1.394998000	1.722119000	1.226683000
O	-0.672408000	1.454318000	2.633042000
O	-1.532091000	3.314498000	1.210720000
C	-0.847517000	0.225448000	3.385702000
C	0.497446000	-0.236457000	3.899643000
C	-2.551563000	3.981187000	1.994037000
C	-2.596095000	5.432659000	1.572903000
H	-1.529324000	0.450222000	4.206727000
H	-1.315689000	-0.525155000	2.752143000
H	-3.506867000	3.484847000	1.820457000
H	-2.293652000	3.882587000	3.050069000
H	-3.350853000	5.962932000	2.155560000
H	-1.632597000	5.914314000	1.738133000
H	-2.850099000	5.520739000	0.517084000
H	0.965315000	0.534400000	4.512196000
H	0.368937000	-1.130702000	4.511700000
H	1.162843000	-0.480395000	3.072799000
H	1.787023000	0.032154000	-1.542662000
H	-0.439935000	-0.809994000	-1.802204000
O	-2.647630000	0.947591000	1.031080000
H	2.337488000	0.555955000	0.048314000
H	-1.609186000	-0.570414000	-0.514625000
H	0.768301000	2.031911000	0.389761000
C	-3.228897000	-4.465830000	-0.867794000
C	-4.914274000	-2.350876000	-0.282562000

C	-5.439192000	-3.521022000	-0.819969000
C	-4.589091000	-4.582437000	-1.109935000
H	-2.572461000	-5.300108000	-1.080378000
H	-5.569714000	-1.528561000	-0.028187000
H	-6.502736000	-3.610382000	-0.997314000
H	-4.986673000	-5.504181000	-1.513632000
C	-2.676978000	-3.278458000	-0.361427000
C	-3.550350000	-2.223807000	-0.056136000
H	-3.186480000	-1.311892000	0.394289000
H	-0.799840000	-4.219986000	0.119149000
C	3.419924000	-1.932237000	0.039153000
C	4.827164000	-0.011859000	-0.839579000
C	4.661905000	-1.173001000	-0.067757000
C	6.050008000	0.642963000	-0.889063000
C	5.782313000	-1.658666000	0.627505000
C	6.998581000	-0.996171000	0.589139000
C	7.137300000	0.161985000	-0.168788000
H	6.156212000	1.529370000	-1.500368000
H	5.682511000	-2.566042000	1.209099000
H	7.841693000	-1.387035000	1.142973000
H	8.087571000	0.677221000	-0.208246000
H	3.544074000	-2.966669000	0.340818000
H	4.011473000	0.361853000	-1.438544000
O	1.414169000	-3.749711000	0.430529000

A16

C	0.992472000	-2.651480000	0.129482000
C	2.061226000	-1.631950000	-0.125069000
H	-1.020073000	3.650236000	-4.846332000
C	-1.606331000	2.022499000	-1.927595000
C	-1.845897000	2.571587000	-3.181450000
C	-0.829596000	3.222704000	-3.871002000
C	0.429524000	3.329804000	-3.293942000
C	0.667478000	2.780826000	-2.039583000
C	-0.340938000	2.113763000	-1.341141000
H	-2.412374000	1.542556000	-1.389753000
H	-2.833196000	2.495954000	-3.617775000
H	1.225158000	3.844980000	-3.815777000
H	1.648714000	2.881890000	-1.592530000
N	0.302883000	0.048184000	0.012236000
C	1.633964000	-0.240382000	-0.512653000
C	-0.409641000	-2.270366000	-0.224982000
C	-0.689547000	-0.836657000	-0.598785000
C	-1.328093000	-3.254788000	-0.171328000
C	-0.018573000	1.487271000	0.009576000
P	-1.275391000	1.814961000	1.322140000
O	-0.529402000	1.497385000	2.706664000
O	-1.284910000	3.415053000	1.294914000
C	-0.832179000	0.329082000	3.512927000
C	0.449491000	-0.403683000	3.841504000

C	-2.229620000	4.161766000	2.098466000
C	-2.229567000	5.595751000	1.618523000
H	-1.321491000	0.694578000	4.416536000
H	-1.538523000	-0.302509000	2.977206000
H	-3.214962000	3.705922000	1.995927000
H	-1.920737000	4.094110000	3.143326000
H	-2.924803000	6.185765000	2.217647000
H	-1.236731000	6.035631000	1.710835000
H	-2.537576000	5.653636000	0.575057000
H	1.146108000	0.248928000	4.368156000
H	0.230163000	-1.259557000	4.482178000
H	0.928098000	-0.767131000	2.933704000
H	1.663652000	-0.115218000	-1.609570000
H	-0.694307000	-0.720801000	-1.696026000
O	-2.588597000	1.141156000	1.168818000
H	2.336968000	0.480496000	-0.094662000
H	-1.675589000	-0.553478000	-0.237687000
H	0.870351000	1.986189000	0.399297000
C	-3.405351000	-2.331083000	-1.300003000
C	-4.894410000	-4.371437000	-0.137643000
C	-5.522389000	-3.430961000	-0.947563000
C	-4.770116000	-2.419045000	-1.532827000
H	-2.842778000	-1.552994000	-1.790777000
H	-5.468122000	-5.170001000	0.313521000
H	-6.586970000	-3.492336000	-1.129451000

H	-5.248397000	-1.694476000	-2.178253000
C	-2.755184000	-3.260162000	-0.472319000
C	-3.529481000	-4.291911000	0.085354000
H	-3.042664000	-5.031121000	0.708423000
H	-0.938600000	-4.203264000	0.182594000
C	3.334444000	-2.034173000	0.043360000
C	4.769189000	-0.220552000	-0.995341000
C	4.591805000	-1.313633000	-0.132757000
C	6.002228000	0.407084000	-1.106051000
C	5.710164000	-1.765094000	0.587676000
C	6.937203000	-1.129258000	0.487132000
C	7.087953000	-0.035676000	-0.359359000
H	6.117804000	1.240862000	-1.785868000
H	5.600362000	-2.622724000	1.238855000
H	7.779249000	-1.491064000	1.061937000
H	8.046492000	0.457876000	-0.446923000
H	3.436602000	-3.052835000	0.401761000
H	3.953122000	0.117626000	-1.615076000
O	1.265952000	-3.757158000	0.572865000

A17

C	-1.325720000	-2.564380000	-0.313664000
C	-2.359572000	-1.511191000	-0.063397000
H	1.623942000	3.098437000	4.972986000
C	-0.463570000	2.555302000	2.359521000

C	-0.038983000	2.956428000	3.620920000
C	1.287700000	2.784607000	3.993878000
C	2.182876000	2.216382000	3.094503000
C	1.755337000	1.815794000	1.835134000
C	0.421857000	1.972970000	1.450071000
H	-1.497159000	2.707268000	2.074337000
H	-0.743211000	3.408015000	4.307050000
H	3.221587000	2.091656000	3.370963000
H	2.470289000	1.395112000	1.141790000
N	-0.516876000	0.085511000	0.014766000
C	-1.897828000	-0.157934000	0.414939000
C	0.070196000	-2.271026000	0.143215000
C	0.376210000	-0.882551000	0.642285000
C	0.956059000	-3.281081000	0.059944000
C	-0.109993000	1.501588000	0.104724000
P	0.880889000	1.892016000	-1.409038000
O	1.522269000	3.303135000	-0.998100000
O	2.142503000	0.887464000	-1.423210000
C	2.156760000	4.140955000	-1.992609000
C	2.282953000	5.537859000	-1.426524000
C	2.325022000	-0.097356000	-2.477323000
C	3.560269000	0.231964000	-3.287965000
H	3.137938000	3.719490000	-2.221144000
H	1.552312000	4.128882000	-2.899900000
H	2.427741000	-1.059478000	-1.977228000

H	1.432764000	-0.112635000	-3.099788000
H	3.730994000	-0.550092000	-4.030086000
H	3.440101000	1.178474000	-3.815178000
H	4.441616000	0.295326000	-2.649504000
H	2.870913000	5.533694000	-0.509012000
H	2.777488000	6.186861000	-2.150928000
H	1.300758000	5.954632000	-1.205282000
H	-2.012956000	-0.075868000	1.511347000
H	0.292635000	-0.841986000	1.742268000
O	0.099832000	1.888618000	-2.665656000
H	-2.528821000	0.610457000	-0.031094000
H	1.403403000	-0.627779000	0.396673000
H	-1.012315000	2.078952000	-0.103465000
C	2.976221000	-2.541083000	1.404721000
C	4.513074000	-4.437720000	0.073677000
C	5.102842000	-3.615558000	1.028315000
C	4.326394000	-2.676174000	1.697239000
H	2.389200000	-1.829385000	1.964029000
H	5.104769000	-5.181922000	-0.442492000
H	6.154949000	-3.715764000	1.258848000
H	4.772216000	-2.049965000	2.458595000
C	2.366303000	-3.348933000	0.431841000
C	3.162504000	-4.312939000	-0.209752000
H	2.704936000	-4.962381000	-0.944910000
H	0.559664000	-4.184945000	-0.390142000

C	-3.637492000	-1.849856000	-0.317141000
C	-5.052921000	-0.015120000	0.712899000
C	-4.875101000	-1.090962000	-0.171157000
C	-6.269101000	0.648676000	0.791951000
C	-5.976498000	-1.485516000	-0.949047000
C	-7.186017000	-0.813285000	-0.879914000
C	-7.336919000	0.261101000	-0.009300000
H	-6.385743000	1.468677000	1.488142000
H	-5.866384000	-2.327310000	-1.620423000
H	-8.014235000	-1.131134000	-1.499018000
H	-8.281963000	0.783635000	0.053096000
H	-3.759597000	-2.844950000	-0.730922000
H	-4.252090000	0.282218000	1.371865000
O	-1.615618000	-3.633067000	-0.829430000

A18

C	0.728050000	-2.168613000	-0.232451000
C	1.915794000	-1.318360000	-0.585946000
H	-1.853783000	6.175839000	-2.171663000
C	-0.363824000	4.113084000	0.073774000
C	-0.906942000	5.271542000	-0.465736000
C	-1.430984000	5.271887000	-1.753706000
C	-1.399571000	4.101848000	-2.499668000
C	-0.852047000	2.941184000	-1.962524000
C	-0.334256000	2.928524000	-0.669111000

H	0.028307000	4.122858000	1.080986000
H	-0.917927000	6.178441000	0.124533000
H	-1.795551000	4.088939000	-3.506834000
H	-0.807595000	2.043725000	-2.560671000
N	0.361382000	0.550174000	-1.023360000
C	1.627702000	-0.056908000	-1.370709000
C	-0.623205000	-1.712707000	-0.700808000
C	-0.768687000	-0.317979000	-1.283462000
C	-1.624077000	-2.604187000	-0.559652000
C	0.309945000	1.656999000	-0.094418000
P	-0.331814000	1.293241000	1.626477000
O	-1.888530000	1.029698000	1.357816000
O	0.215279000	-0.170814000	2.004553000
C	-2.804197000	0.826343000	2.464095000
C	-4.217047000	0.946239000	1.939114000
C	1.262251000	-0.387594000	2.987063000
C	0.912198000	-1.604865000	3.814849000
H	-2.619543000	-0.164415000	2.882389000
H	-2.596513000	1.575227000	3.228708000
H	2.191525000	-0.546307000	2.438784000
H	1.357135000	0.508284000	3.598498000
H	0.793030000	-2.482065000	3.180427000
H	1.712897000	-1.801102000	4.530323000
H	-0.010610000	-1.443609000	4.372613000
H	-4.412073000	0.198767000	1.170699000

H	-4.925389000	0.793157000	2.754842000
H	-4.388271000	1.935299000	1.515256000
H	1.593850000	-0.321798000	-2.438099000
H	-0.898011000	-0.401406000	-2.373704000
O	-0.004570000	2.324566000	2.641199000
H	2.422906000	0.674599000	-1.275965000
H	-1.672420000	0.146166000	-0.896423000
H	1.347710000	1.913632000	0.137725000
C	-3.638495000	-1.598515000	-1.750953000
C	-5.235343000	-3.560368000	-0.597009000
C	-5.807314000	-2.607643000	-1.433583000
C	-5.000889000	-1.634280000	-2.012333000
H	-3.040945000	-0.844249000	-2.235518000
H	-5.850019000	-4.329389000	-0.148500000
H	-6.868882000	-2.629193000	-1.640131000
H	-5.434248000	-0.898729000	-2.676962000
C	-3.041192000	-2.543785000	-0.899505000
C	-3.873767000	-3.531619000	-0.343441000
H	-3.432679000	-4.281117000	0.300898000
H	-1.323741000	-3.530647000	-0.082509000
C	3.127387000	-1.799055000	-0.263164000
C	5.491183000	-2.105753000	-0.884265000
C	4.465222000	-1.234941000	-0.490431000
C	6.779550000	-1.643230000	-1.108278000
C	4.793194000	0.110878000	-0.281298000

C	6.086250000	0.571171000	-0.488581000
C	7.082573000	-0.300233000	-0.913892000
H	7.550231000	-2.333343000	-1.424878000
H	4.043201000	0.797421000	0.084328000
H	6.317878000	1.612534000	-0.308437000
H	8.088644000	0.061438000	-1.077874000
H	3.117024000	-2.782366000	0.196956000
H	5.264877000	-3.155051000	-1.023967000
O	0.866537000	-3.206669000	0.397270000

A19

C	1.007931000	-2.490416000	-0.293889000
C	2.135991000	-1.507570000	-0.336771000
H	-0.469740000	4.794459000	-4.006869000
C	-1.239903000	2.599418000	-1.538251000
C	-1.400109000	3.401553000	-2.661203000
C	-0.341450000	4.167391000	-3.134834000
C	0.878768000	4.130309000	-2.472422000
C	1.035794000	3.326607000	-1.349940000
C	-0.015569000	2.544747000	-0.867886000
H	-2.079714000	2.029102000	-1.169355000
H	-2.358473000	3.435059000	-3.162499000
H	1.706750000	4.731922000	-2.823229000
H	1.986293000	3.318432000	-0.831265000
N	0.462017000	0.218172000	0.076670000

C	1.797029000	-0.044207000	-0.455888000
C	-0.360574000	-1.968013000	-0.604467000
C	-0.542801000	-0.475953000	-0.725803000
C	-1.337016000	-2.885353000	-0.736301000
C	0.223948000	1.647702000	0.341690000
P	-0.936642000	1.968318000	1.748314000
O	-2.332768000	1.280238000	1.309766000
O	-0.392870000	1.103584000	2.982687000
C	-3.560050000	1.668029000	1.985611000
C	-4.732091000	1.327261000	1.093194000
C	-0.528106000	-0.323879000	3.172911000
C	-0.445015000	-0.612477000	4.656163000
H	-3.615557000	1.125094000	2.931201000
H	-3.512888000	2.734617000	2.201658000
H	0.270627000	-0.817941000	2.623255000
H	-1.480633000	-0.653930000	2.757293000
H	0.503883000	-0.264936000	5.063776000
H	-1.251946000	-0.119066000	5.197777000
H	-0.519401000	-1.687767000	4.826282000
H	-4.749479000	0.264745000	0.852168000
H	-5.663774000	1.579880000	1.601892000
H	-4.688824000	1.893630000	0.163037000
H	1.877604000	0.291061000	-1.504903000
H	-1.531893000	-0.203131000	-0.367836000
O	-1.020874000	3.398985000	2.096079000

H	2.520553000	0.536779000	0.116146000
H	-0.483977000	-0.165840000	-1.782979000
H	1.144513000	2.004722000	0.810550000
C	-3.290020000	-1.667728000	-1.798570000
C	-4.987533000	-3.682351000	-0.910286000
C	-5.504590000	-2.608079000	-1.627129000
C	-4.648540000	-1.609155000	-2.077090000
H	-2.642258000	-0.898128000	-2.188872000
H	-5.642452000	-4.472262000	-0.567574000
H	-6.562740000	-2.557447000	-1.845811000
H	-5.039257000	-0.783297000	-2.656600000
C	-2.751560000	-2.735344000	-1.063712000
C	-3.629119000	-3.749624000	-0.645329000
H	-3.229054000	-4.594057000	-0.098990000
H	-1.020506000	-3.904060000	-0.539322000
C	3.380662000	-2.010642000	-0.233262000
C	4.941540000	-0.112210000	-0.860240000
C	4.681705000	-1.349935000	-0.250501000
C	6.212512000	0.444286000	-0.828093000
C	5.756626000	-2.013772000	0.364266000
C	7.021774000	-1.450798000	0.408042000
C	7.254746000	-0.214851000	-0.186302000
H	6.391521000	1.394401000	-1.313664000
H	5.582316000	-2.980430000	0.818946000
H	7.828866000	-1.978440000	0.898385000

H	8.242796000	0.224574000	-0.161043000
H	3.416076000	-3.083451000	-0.077141000
H	4.160054000	0.402528000	-1.397342000
O	1.201057000	-3.672618000	-0.052373000

A20

C	1.002528000	-2.204478000	0.001991000
C	2.046401000	-1.276166000	-0.546324000
H	-3.678981000	4.544745000	-3.029643000
C	-0.732482000	3.340908000	-1.867960000
C	-1.616598000	4.141402000	-2.577630000
C	-2.986803000	3.922481000	-2.478137000
C	-3.458733000	2.903713000	-1.661395000
C	-2.571583000	2.103889000	-0.946436000
C	-1.194251000	2.310705000	-1.045289000
H	0.332711000	3.517376000	-1.953053000
H	-1.237437000	4.937247000	-3.205142000
H	-4.522894000	2.729003000	-1.572563000
H	-2.953827000	1.330419000	-0.294926000
N	0.196157000	0.268097000	-1.132215000
C	1.580919000	-0.081430000	-1.352316000
C	-0.404759000	-2.031899000	-0.477167000
C	-0.715057000	-0.834914000	-1.353337000
C	-1.301275000	-2.944602000	-0.057353000
C	-0.168540000	1.427683000	-0.337427000

P	-0.652148000	1.111100000	1.432494000
O	0.682602000	0.410872000	1.975349000
O	-0.598421000	2.579629000	2.099308000
C	0.726199000	-0.110850000	3.329792000
C	2.038912000	-0.834413000	3.525742000
C	-1.743540000	3.196142000	2.736692000
C	-1.869561000	4.628594000	2.264317000
H	0.630182000	0.728815000	4.021622000
H	-0.123511000	-0.778633000	3.470416000
H	-2.634284000	2.611808000	2.510879000
H	-1.572351000	3.152913000	3.813527000
H	-2.700240000	5.113929000	2.780022000
H	-0.959691000	5.189487000	2.478443000
H	-2.058773000	4.670609000	1.192261000
H	2.880210000	-0.163806000	3.350595000
H	2.100195000	-1.201495000	4.551795000
H	2.115245000	-1.684120000	2.849376000
H	1.715221000	-0.290711000	-2.424861000
H	-0.657712000	-1.118104000	-2.414486000
O	-1.918763000	0.381372000	1.685896000
H	2.208306000	0.780644000	-1.127261000
H	-1.735836000	-0.505454000	-1.177080000
H	0.740626000	2.020528000	-0.231626000
C	-3.337974000	-2.618812000	-1.528131000
C	-4.882604000	-3.930512000	0.373627000

C	-5.477412000	-3.448422000	-0.787844000
C	-4.697371000	-2.801651000	-1.739481000
H	-2.749533000	-2.143235000	-2.297475000
H	-5.478658000	-4.444423000	1.115939000
H	-6.537300000	-3.586124000	-0.954974000
H	-5.148073000	-2.443131000	-2.655476000
C	-2.722306000	-3.083927000	-0.355650000
C	-3.523040000	-3.761188000	0.578466000
H	-3.062927000	-4.143901000	1.480115000
H	-0.907601000	-3.676616000	0.639584000
C	3.327341000	-1.576650000	-0.261917000
C	4.737537000	-0.045312000	-1.720586000
C	4.574987000	-0.913470000	-0.628591000
C	5.967166000	0.539240000	-1.990396000
C	5.707025000	-1.185286000	0.159036000
C	6.931102000	-0.592109000	-0.103761000
C	7.066443000	0.277752000	-1.180638000
H	6.068718000	1.197143000	-2.843389000
H	5.609843000	-1.868782000	0.992611000
H	7.782382000	-0.813304000	0.526206000
H	8.021973000	0.737670000	-1.393929000
H	3.450582000	-2.442724000	0.379257000
H	3.912549000	0.149957000	-2.387054000
O	1.302580000	-3.090548000	0.791572000

A21

C	0.258646000	-2.559553000	0.316557000
C	1.433538000	-1.853061000	-0.283829000
H	-4.643198000	3.412307000	-2.868902000
C	-2.432573000	2.483230000	-0.469810000
C	-3.609858000	2.946277000	-1.043763000
C	-3.727561000	3.045080000	-2.425012000
C	-2.655915000	2.681643000	-3.230985000
C	-1.478685000	2.221189000	-2.654428000
C	-1.350047000	2.109532000	-1.268750000
H	-2.354021000	2.428294000	0.606792000
H	-4.433967000	3.238672000	-0.406993000
H	-2.730848000	2.764390000	-4.307106000
H	-0.644252000	1.952674000	-3.289985000
N	0.007814000	0.132171000	-0.379388000
C	1.202165000	-0.507995000	-0.920623000
C	-1.095556000	-1.999962000	0.013005000
C	-1.178069000	-0.662510000	-0.678328000
C	-2.152787000	-2.739759000	0.399835000
C	-0.043662000	1.584873000	-0.697715000
P	0.523109000	2.578434000	0.763635000
O	-0.326008000	2.115653000	2.046209000
O	2.006864000	1.976744000	0.978543000
C	-0.150209000	0.900759000	2.812617000
C	-0.411314000	1.210218000	4.271544000

C	3.003748000	2.746730000	1.699374000
C	4.376230000	2.330205000	1.219609000
H	-0.852680000	0.159611000	2.434454000
H	0.857600000	0.518917000	2.658165000
H	2.819366000	3.805948000	1.523699000
H	2.883860000	2.545754000	2.765947000
H	4.499135000	2.550115000	0.159426000
H	5.140255000	2.877928000	1.773702000
H	4.539692000	1.263761000	1.372665000
H	-1.417223000	1.605903000	4.410089000
H	-0.315868000	0.298173000	4.863205000
H	0.299954000	1.944041000	4.650573000
H	1.120280000	-0.607124000	-2.020514000
H	-1.306639000	-0.798276000	-1.767749000
O	0.462634000	4.040668000	0.575134000
H	2.057885000	0.131900000	-0.717757000
H	-2.060689000	-0.135210000	-0.328911000
H	0.719097000	1.799244000	-1.452142000
C	-4.172608000	-1.707156000	-0.737519000
C	-5.818663000	-3.020028000	1.078488000
C	-6.378326000	-2.199441000	0.104465000
C	-5.550091000	-1.553535000	-0.806232000
H	-3.557491000	-1.219604000	-1.477253000
H	-6.454775000	-3.537568000	1.784065000
H	-7.451314000	-2.074369000	0.048220000

H	-5.977961000	-0.929598000	-1.579431000
C	-3.587029000	-2.517033000	0.248737000
C	-4.444432000	-3.184495000	1.139997000
H	-4.013762000	-3.832445000	1.892535000
H	-1.888693000	-3.640980000	0.942786000
C	2.624378000	-2.474303000	-0.186623000
C	4.191347000	-1.202549000	-1.725898000
C	3.948079000	-2.094045000	-0.668657000
C	5.487448000	-0.902907000	-2.121034000
C	5.059998000	-2.687113000	-0.046714000
C	6.354007000	-2.376758000	-0.432768000
C	6.573827000	-1.479127000	-1.472480000
H	5.649672000	-0.220917000	-2.945147000
H	4.893413000	-3.394733000	0.755205000
H	7.191654000	-2.841068000	0.070297000
H	7.582207000	-1.240794000	-1.782969000
H	2.595471000	-3.403655000	0.372034000
H	3.367486000	-0.766232000	-2.268244000
O	0.397620000	-3.568966000	0.991626000

A22

C	-0.788257000	-2.932251000	0.016553000
C	-1.953580000	-1.991024000	-0.057836000
H	2.658403000	2.300563000	4.993708000
C	1.008108000	2.863193000	2.083549000

C	1.723802000	3.128691000	3.243009000
C	2.096607000	2.093290000	4.092692000
C	1.735242000	0.792147000	3.773532000
C	1.010966000	0.525962000	2.616128000
C	0.645928000	1.555227000	1.749809000
H	0.730387000	3.683475000	1.437543000
H	1.991096000	4.149793000	3.481851000
H	2.010677000	-0.024741000	4.427550000
H	0.709596000	-0.485564000	2.391058000
N	-0.391384000	-0.159690000	0.262535000
C	-1.660094000	-0.541537000	-0.353564000
C	0.581285000	-2.356939000	-0.190488000
C	0.724870000	-0.865024000	-0.364190000
C	1.601173000	-3.236837000	-0.189537000
C	-0.205064000	1.261334000	0.510294000
P	0.275035000	2.210365000	-1.036031000
O	-0.156494000	3.749413000	-0.780678000
O	1.874306000	2.256319000	-0.955963000
C	-1.161250000	4.411558000	-1.591336000
C	-2.492457000	4.468833000	-0.870751000
C	2.649905000	2.891514000	-2.000986000
C	4.115525000	2.721199000	-1.670002000
H	-0.770598000	5.412280000	-1.772947000
H	-1.246623000	3.886493000	-2.540940000
H	2.395658000	2.429937000	-2.955835000

H	2.377718000	3.947954000	-2.041766000
H	4.725532000	3.195389000	-2.440296000
H	4.351216000	3.179700000	-0.710267000
H	4.381725000	1.665848000	-1.620423000
H	-2.391667000	4.950472000	0.101902000
H	-3.207422000	5.041485000	-1.464159000
H	-2.903480000	3.469408000	-0.726434000
H	-2.446177000	0.097356000	0.035986000
H	1.653282000	-0.530805000	0.096720000
O	-0.298040000	1.652363000	-2.285646000
H	-1.637599000	-0.382203000	-1.441865000
H	0.786010000	-0.612950000	-1.434945000
H	-1.201137000	1.667897000	0.709193000
C	3.613559000	-1.970937000	-1.073595000
C	5.271205000	-3.923097000	0.009502000
C	5.826116000	-2.843249000	-0.669378000
C	4.990757000	-1.876313000	-1.216863000
H	2.987194000	-1.223800000	-1.535192000
H	5.910908000	-4.688144000	0.428845000
H	6.899000000	-2.762653000	-0.781356000
H	5.414176000	-1.046382000	-1.766989000
C	3.035070000	-3.045275000	-0.379531000
C	3.895853000	-4.027044000	0.139736000
H	3.467644000	-4.875490000	0.657746000
H	1.303965000	-4.260056000	0.013557000

C	-3.176695000	-2.526922000	0.074708000
C	-4.782761000	-0.730948000	0.825578000
C	-4.491832000	-1.869253000	0.063837000
C	-6.051753000	-0.167775000	0.809187000
C	-5.526155000	-2.439903000	-0.689467000
C	-6.789742000	-1.867410000	-0.719302000
C	-7.057440000	-0.726070000	0.028359000
H	-6.258062000	0.703811000	1.416350000
H	-5.327580000	-3.335572000	-1.263935000
H	-7.568987000	-2.318083000	-1.319492000
H	-8.044750000	-0.284397000	0.014313000
H	-3.194876000	-3.607698000	0.173172000
H	-4.021288000	-0.308968000	1.466522000
O	-0.951413000	-4.123448000	0.231746000

A23

C	0.984420000	-2.670640000	0.199089000
C	-0.402752000	-2.285585000	-0.207757000
H	-0.781917000	3.558391000	-4.984275000
C	-1.486799000	1.985847000	-2.061392000
C	-1.676312000	2.515437000	-3.332183000
C	-0.630278000	3.146079000	-3.995655000
C	0.608349000	3.252880000	-3.375597000
C	0.796457000	2.723520000	-2.104495000
C	-0.242308000	2.076599000	-1.431691000

H	-2.315746000	1.523912000	-1.543339000
H	-2.648114000	2.440883000	-3.802197000
H	1.426587000	3.752560000	-3.877153000
H	1.761863000	2.824430000	-1.623995000
N	0.330325000	0.029059000	-0.016120000
C	-0.653855000	-0.860717000	-0.632670000
C	2.072375000	-1.672635000	-0.057989000
C	1.673001000	-0.286733000	-0.492488000
C	3.335912000	-2.086939000	0.149800000
C	0.025040000	1.471152000	-0.059505000
P	-1.271866000	1.836322000	1.202364000
O	-0.612383000	1.457189000	2.615835000
O	-1.196938000	3.433885000	1.218139000
C	-0.874280000	0.189696000	3.272268000
C	-0.687930000	0.377339000	4.761416000
C	-2.170907000	4.214782000	1.952157000
C	-2.052706000	5.655248000	1.507403000
H	-1.888189000	-0.126884000	3.030724000
H	-0.174225000	-0.542794000	2.871778000
H	-3.164658000	3.813397000	1.751963000
H	-1.960943000	4.111246000	3.018212000
H	-2.261513000	5.749013000	0.442172000
H	-2.767738000	6.270714000	2.055439000
H	-1.050797000	6.038947000	1.698748000
H	0.320536000	0.724220000	4.986623000

H	-0.845679000	-0.573095000	5.273588000
H	-1.399861000	1.102395000	5.156029000
H	-1.647481000	-0.555032000	-0.312229000
H	2.371017000	0.436691000	-0.070547000
O	-2.610376000	1.237360000	0.974696000
H	-0.623968000	-0.777277000	-1.732472000
H	1.737591000	-0.188835000	-1.590620000
H	0.905042000	1.967983000	0.352687000
C	5.705103000	-1.842625000	0.732295000
C	6.060513000	0.293219000	-0.995197000
C	7.125782000	-0.151604000	-0.220768000
C	6.943220000	-1.226941000	0.642669000
H	5.570575000	-2.686153000	1.397103000
H	6.200823000	1.112656000	-1.687682000
H	8.093052000	0.326134000	-0.299617000
H	7.769147000	-1.590276000	1.239470000
C	4.606877000	-1.388484000	-0.016945000
C	4.816414000	-0.314071000	-0.895810000
H	4.016843000	0.025346000	-1.535936000
H	3.415848000	-3.096841000	0.537346000
C	-1.333573000	-3.258022000	-0.149693000
C	-3.549801000	-4.269745000	0.073633000
C	-2.751965000	-3.258666000	-0.488849000
C	-4.909284000	-4.342972000	-0.182207000
C	-3.372363000	-2.344527000	-1.355184000

C	-4.731672000	-2.425941000	-1.620017000
C	-5.507883000	-3.416781000	-1.029937000
H	-5.501522000	-5.125457000	0.273173000
H	-2.790615000	-1.583476000	-1.850263000
H	-5.186774000	-1.712878000	-2.294345000
H	-6.568093000	-3.473137000	-1.237180000
H	-0.964363000	-4.199057000	0.243678000
H	-3.085828000	-4.997634000	0.726749000
O	1.231203000	-3.764353000	0.685774000

A24

C	0.328198000	-2.261875000	0.408416000
C	1.548092000	-1.746538000	-0.287451000
H	-4.167237000	4.212144000	-2.308829000
C	-2.119425000	2.407714000	-0.293486000
C	-3.236679000	3.126484000	-0.706490000
C	-3.299434000	3.649627000	-1.991183000
C	-2.237732000	3.447318000	-2.866679000
C	-1.125491000	2.727296000	-2.453154000
C	-1.048715000	2.201393000	-1.161826000
H	-2.083999000	2.009798000	0.710455000
H	-4.057958000	3.277498000	-0.018641000
H	-2.274800000	3.851862000	-3.869510000
H	-0.304457000	2.565020000	-3.140224000
N	0.132487000	0.036160000	-1.288567000

C	1.347278000	-0.746254000	-1.411922000
C	-1.007965000	-1.868638000	-0.146979000
C	-1.060649000	-0.779943000	-1.199362000
C	-2.070936000	-2.545165000	0.330972000
C	0.191643000	1.402311000	-0.791968000
P	0.813258000	1.617182000	0.954495000
O	0.536360000	3.162913000	1.316872000
O	-0.224331000	0.786019000	1.851224000
C	1.565126000	4.051790000	1.817552000
C	1.601175000	5.309141000	0.975166000
C	0.095668000	0.471465000	3.235796000
C	-0.912068000	-0.530653000	3.749853000
H	1.305959000	4.282591000	2.851852000
H	2.519926000	3.528569000	1.806545000
H	1.108789000	0.073094000	3.270888000
H	0.061427000	1.399511000	3.810676000
H	-0.708261000	-0.739224000	4.801591000
H	-1.926834000	-0.140805000	3.668887000
H	-0.842571000	-1.464715000	3.195325000
H	0.627522000	5.798309000	0.963613000
H	2.332958000	6.007258000	1.385707000
H	1.885128000	5.082817000	-0.052680000
H	1.291096000	-1.304813000	-2.357958000
H	-1.218208000	-1.244582000	-2.184459000
O	2.239286000	1.231373000	1.111173000

H	2.199656000	-0.080517000	-1.495052000
H	-1.921873000	-0.140306000	-1.024179000
H	1.027480000	1.871886000	-1.323993000
C	-4.050430000	-1.867452000	-1.120046000
C	-5.751694000	-3.044708000	0.737033000
C	-6.281223000	-2.431230000	-0.393382000
C	-5.423441000	-1.851556000	-1.321069000
H	-3.413980000	-1.432714000	-1.873162000
H	-6.407689000	-3.509269000	1.461145000
H	-7.350696000	-2.413929000	-0.554801000
H	-5.824854000	-1.387042000	-2.211959000
C	-3.494717000	-2.468866000	0.021717000
C	-4.380794000	-3.069655000	0.933736000
H	-3.973981000	-3.556513000	1.810660000
H	-1.824143000	-3.264259000	1.104508000
C	2.724864000	-2.285089000	0.069014000
C	4.922285000	-3.023214000	-0.799750000
C	4.071422000	-1.972809000	-0.432258000
C	6.206085000	-2.771872000	-1.262663000
C	4.561508000	-0.661936000	-0.493474000
C	5.853267000	-0.416297000	-0.940932000
C	6.675645000	-1.465059000	-1.337810000
H	6.843423000	-3.596051000	-1.554846000
H	3.943410000	0.151757000	-0.138415000
H	6.223055000	0.600448000	-0.968525000

H	7.679986000	-1.267363000	-1.688297000
H	2.667665000	-3.076591000	0.809672000
H	4.565138000	-4.042955000	-0.729748000
O	0.417118000	-2.989825000	1.388492000

A25

C	-0.744128000	-2.743696000	-0.300191000
C	0.635615000	-2.259184000	0.018780000
H	1.180361000	2.972933000	5.324137000
C	1.622968000	1.884473000	2.141174000
C	1.935321000	2.266260000	3.440332000
C	0.934111000	2.674814000	4.313785000
C	-0.384301000	2.706550000	3.876982000
C	-0.694814000	2.327058000	2.576808000
C	0.299683000	1.905461000	1.692074000
H	2.413741000	1.591895000	1.463902000
H	2.966312000	2.250263000	3.768388000
H	-1.171121000	3.031576000	4.544762000
H	-1.724185000	2.367857000	2.242799000
N	-0.289111000	0.012842000	0.079604000
C	0.823235000	-0.840848000	0.498553000
C	-1.884956000	-1.823716000	0.019056000
C	-1.544552000	-0.490222000	0.632692000
C	-3.124787000	-2.287141000	-0.202586000
C	-0.096933000	1.459564000	0.289843000

P	0.992481000	2.112524000	-1.049629000
O	0.146404000	1.922687000	-2.401401000
O	0.859894000	3.680492000	-0.761406000
C	0.502198000	0.950972000	-3.419482000
C	-0.699276000	0.092176000	-3.746423000
C	1.747219000	4.646823000	-1.376266000
C	1.016277000	5.441935000	-2.438820000
H	0.827140000	1.521145000	-4.290696000
H	1.345273000	0.358023000	-3.069320000
H	2.085365000	5.291099000	-0.565548000
H	2.616889000	4.129360000	-1.781792000
H	1.681666000	6.201264000	-2.853863000
H	0.680117000	4.797111000	-3.249890000
H	0.146332000	5.943298000	-2.014869000
H	-1.535061000	0.705263000	-4.084495000
H	-0.444186000	-0.607696000	-4.544048000
H	-1.013054000	-0.479426000	-2.874565000
H	0.924892000	-0.831693000	1.597038000
H	-2.331154000	0.232615000	0.444361000
O	2.371314000	1.567760000	-1.119901000
H	1.744569000	-0.435920000	0.084541000
H	-1.480162000	-0.603447000	1.729496000
H	-1.059105000	1.921146000	0.062236000
C	-4.699992000	-0.328110000	-0.444225000
C	-6.710877000	-1.785252000	0.796899000

C	-6.963400000	-0.486442000	0.369740000
C	-5.956105000	0.234478000	-0.261537000
H	-3.939524000	0.231530000	-0.970706000
H	-7.491890000	-2.360491000	1.276122000
H	-7.940457000	-0.045408000	0.514091000
H	-6.151196000	1.235716000	-0.622409000
C	-4.423754000	-1.628334000	-0.003089000
C	-5.460668000	-2.353598000	0.598864000
H	-5.274598000	-3.370143000	0.920971000
H	-3.170291000	-3.314292000	-0.550727000
C	1.630626000	-3.152589000	-0.154026000
C	3.882496000	-4.009423000	-0.568556000
C	3.067800000	-3.060951000	0.074122000
C	5.259498000	-3.999361000	-0.418936000
C	3.695559000	-2.120929000	0.907478000
C	5.073758000	-2.119563000	1.066078000
C	5.862654000	-3.049837000	0.399253000
H	5.862598000	-4.735165000	-0.933925000
H	3.109233000	-1.404292000	1.459244000
H	5.534034000	-1.388812000	1.717593000
H	6.937174000	-3.041142000	0.523858000
H	1.301276000	-4.104847000	-0.555460000
H	3.416067000	-4.755719000	-1.198668000
O	-0.941779000	-3.844634000	-0.791743000

A26

C	0.037082000	-2.847970000	0.389573000
C	1.281788000	-2.169457000	-0.080928000
H	-4.813992000	3.659088000	0.653523000
C	-1.966481000	1.854257000	1.023942000
C	-3.168864000	2.465700000	1.352374000
C	-3.877146000	3.183921000	0.394986000
C	-3.375833000	3.281892000	-0.896900000
C	-2.174613000	2.665330000	-1.225524000
C	-1.455499000	1.947592000	-0.271613000
H	-1.421515000	1.294275000	1.769284000
H	-3.554594000	2.380038000	2.359598000
H	-3.920090000	3.834447000	-1.651318000
H	-1.789286000	2.746330000	-2.233937000
N	-0.063440000	-0.117320000	-0.231441000
C	1.166266000	-0.787862000	-0.666511000
C	-1.258119000	-2.239765000	-0.046408000
C	-1.211956000	-0.892117000	-0.714631000
C	-2.374291000	-2.947515000	0.208347000
C	-0.138223000	1.292512000	-0.651224000
P	1.286110000	2.306297000	0.003311000
O	0.806413000	3.754994000	-0.475316000
O	1.130026000	2.355777000	1.599721000
C	1.677868000	4.899777000	-0.312511000
C	1.049339000	6.076254000	-1.024779000

C	2.123841000	1.813023000	2.508478000
C	1.514168000	0.716321000	3.354763000
H	1.788046000	5.099661000	0.755124000
H	2.657653000	4.656685000	-0.724596000
H	2.973648000	1.455814000	1.929247000
H	2.454133000	2.648591000	3.126122000
H	1.173577000	-0.112862000	2.735863000
H	2.260304000	0.338074000	4.055787000
H	0.667008000	1.092171000	3.928643000
H	0.065899000	6.298809000	-0.612078000
H	1.681173000	6.958021000	-0.908203000
H	0.940101000	5.870085000	-2.089230000
H	1.199874000	-0.836574000	-1.772371000
H	-1.160046000	-0.999764000	-1.815205000
O	2.644018000	1.915942000	-0.452916000
H	2.020492000	-0.192566000	-0.364761000
H	-2.126985000	-0.351477000	-0.495204000
H	-0.018951000	1.383438000	-1.744372000
C	-4.213260000	-1.866898000	-1.163852000
C	-6.102258000	-3.091756000	0.466374000
C	-6.517220000	-2.270960000	-0.577278000
C	-5.567429000	-1.668983000	-1.394505000
H	-3.498379000	-1.416562000	-1.834941000
H	-6.833150000	-3.575885000	1.100289000
H	-7.571577000	-2.112529000	-0.759998000

H	-5.881923000	-1.046688000	-2.221720000
C	-3.773903000	-2.676935000	-0.105193000
C	-4.750816000	-3.299873000	0.689498000
H	-4.432304000	-3.947432000	1.496214000
H	-2.201032000	-3.861903000	0.765790000
C	2.426517000	-2.872074000	0.041974000
C	4.788255000	-3.470832000	0.149111000
C	3.807474000	-2.580481000	-0.325144000
C	6.132206000	-3.286317000	-0.129947000
C	4.238262000	-1.501637000	-1.116888000
C	5.584723000	-1.320688000	-1.397990000
C	6.537481000	-2.205637000	-0.906047000
H	6.863015000	-3.985784000	0.253640000
H	3.533591000	-0.796331000	-1.524082000
H	5.889283000	-0.479872000	-2.006546000
H	7.585486000	-2.057217000	-1.129785000
H	2.292484000	-3.837567000	0.517626000
H	4.476778000	-4.315409000	0.750130000
O	0.071735000	-3.871118000	1.057585000

A27

C	0.495914000	-2.216067000	0.500423000
C	1.691265000	-1.676653000	-0.220890000
H	-4.473891000	3.666221000	-2.562404000
C	-1.300786000	2.481925000	-2.646843000

C	-2.472158000	3.084681000	-3.085027000
C	-3.559856000	3.195482000	-2.225664000
C	-3.464222000	2.698993000	-0.932193000
C	-2.288644000	2.097893000	-0.494833000
C	-1.190948000	1.983438000	-1.347020000
H	-0.459106000	2.389779000	-3.321848000
H	-2.534887000	3.468835000	-4.094568000
H	-4.305821000	2.778147000	-0.257093000
H	-2.228198000	1.719611000	0.515404000
N	0.177999000	-0.075439000	-1.390943000
C	1.456743000	-0.760777000	-1.411112000
C	-0.855807000	-1.934313000	-0.083271000
C	-0.941756000	-0.986749000	-1.260845000
C	-1.899584000	-2.560908000	0.492489000
C	0.113799000	1.312867000	-0.947909000
P	0.713549000	1.680728000	0.784873000
O	0.271978000	3.193057000	1.134950000
O	-0.233547000	0.815846000	1.739548000
C	1.181565000	4.310993000	1.004923000
C	0.913839000	5.291379000	2.126313000
C	0.073958000	0.699904000	3.158765000
C	-0.795182000	-0.384123000	3.752999000
H	2.204828000	3.938785000	1.034520000
H	1.001508000	4.774296000	0.032721000
H	1.132572000	0.463961000	3.262010000

H	-0.118966000	1.668376000	3.623238000
H	-0.593166000	-0.461338000	4.822799000
H	-1.852242000	-0.152657000	3.620997000
H	-0.580077000	-1.346100000	3.291033000
H	1.115053000	4.835503000	3.095628000
H	1.560406000	6.163701000	2.016775000
H	-0.122624000	5.627800000	2.109362000
H	1.489821000	-1.375371000	-2.322327000
H	-0.988550000	-1.573593000	-2.189929000
O	2.175300000	1.459587000	0.925893000
H	2.254369000	-0.030942000	-1.499303000
H	-1.866244000	-0.417493000	-1.214440000
H	0.904647000	1.828042000	-1.506329000
C	-3.874759000	-2.210474000	-1.067325000
C	-5.586785000	-2.905035000	1.009703000
C	-6.110776000	-2.557479000	-0.231196000
C	-5.248070000	-2.220763000	-1.267815000
H	-3.230242000	-1.976452000	-1.899521000
H	-6.247593000	-3.182239000	1.820257000
H	-7.180448000	-2.561327000	-0.392235000
H	-5.645760000	-1.969971000	-2.242253000
C	-3.325887000	-2.540610000	0.182371000
C	-4.215559000	-2.907317000	1.206993000
H	-3.812284000	-3.189417000	2.171049000
H	-1.636749000	-3.163648000	1.355176000

C	2.892257000	-2.126980000	0.175563000
C	5.131810000	-2.787576000	-0.650448000
C	4.223022000	-1.768637000	-0.337430000
C	6.401805000	-2.488694000	-1.123328000
C	4.640089000	-0.437345000	-0.463202000
C	5.918437000	-0.143056000	-0.919796000
C	6.798946000	-1.163260000	-1.262691000
H	7.084851000	-3.289779000	-1.373461000
H	3.975329000	0.357714000	-0.152722000
H	6.231450000	0.889996000	-0.997616000
H	7.792316000	-0.928161000	-1.621284000
H	2.872825000	-2.874493000	0.962392000
H	4.831199000	-3.820814000	-0.530547000
O	0.618823000	-2.871521000	1.526834000

A28

C	-0.056676000	-2.818363000	0.471329000
C	1.202724000	-2.192792000	-0.032371000
H	-4.719045000	3.831357000	0.500971000
C	-1.916119000	1.967746000	0.917490000
C	-3.096441000	2.627159000	1.233133000
C	-3.799695000	3.318226000	0.252357000
C	-3.315622000	3.341158000	-1.049576000
C	-2.136704000	2.676684000	-1.365132000
C	-1.423175000	1.984868000	-0.388153000

H	-1.373883000	1.430193000	1.681117000
H	-3.468285000	2.601205000	2.248852000
H	-3.856116000	3.872466000	-1.821763000
H	-1.764704000	2.698920000	-2.381541000
N	-0.088929000	-0.113936000	-0.267210000
C	1.119954000	-0.835365000	-0.677304000
C	-1.337860000	-2.195786000	0.015473000
C	-1.261803000	-0.877754000	-0.706763000
C	-2.470088000	-2.865808000	0.300301000
C	-0.130050000	1.275399000	-0.753279000
P	1.330134000	2.281391000	-0.171130000
O	0.876551000	3.717995000	-0.705101000
O	1.203155000	2.385615000	1.425333000
C	1.820317000	4.810531000	-0.838015000
C	1.648350000	5.810059000	0.287771000
C	2.194988000	1.843677000	2.336639000
C	1.560366000	0.811075000	3.243388000
H	2.831975000	4.406422000	-0.875171000
H	1.599610000	5.265736000	-1.802615000
H	2.572281000	2.690393000	2.910792000
H	3.017452000	1.427690000	1.757142000
H	1.172504000	-0.029486000	2.669367000
H	2.306026000	0.433509000	3.945286000
H	0.742340000	1.246920000	3.817102000
H	1.879116000	5.361154000	1.253115000

H	2.320126000	6.656372000	0.132600000
H	0.625185000	6.183417000	0.319085000
H	1.148260000	-0.932940000	-1.780056000
H	-1.224787000	-1.031464000	-1.802497000
O	2.670941000	1.842599000	-0.635531000
H	1.991123000	-0.250585000	-0.404524000
H	-2.159796000	-0.304201000	-0.500532000
H	-0.024802000	1.310147000	-1.851144000
C	-4.851862000	-3.149741000	0.798331000
C	-5.639620000	-1.566364000	-1.332864000
C	-6.599522000	-2.121091000	-0.494268000
C	-6.199152000	-2.918323000	0.573085000
H	-4.544874000	-3.778974000	1.623805000
H	-5.943327000	-0.962855000	-2.177836000
H	-7.650745000	-1.944458000	-0.678388000
H	-6.938296000	-3.365839000	1.224105000
C	-3.864171000	-2.574043000	-0.018487000
C	-4.289344000	-1.787519000	-1.100557000
H	-3.567372000	-1.374038000	-1.787482000
H	-2.316751000	-3.762218000	0.891694000
C	2.329310000	-2.919228000	0.116586000
C	4.672968000	-3.581133000	0.236802000
C	3.715927000	-2.681338000	-0.267262000
C	6.020208000	-3.447097000	-0.054709000
C	4.173835000	-1.646215000	-1.101157000

C	5.523606000	-1.515847000	-1.394399000
C	6.452777000	-2.409154000	-0.873158000
H	6.732174000	-4.152777000	0.352229000
H	3.488177000	-0.935862000	-1.531742000
H	5.849346000	-0.708017000	-2.035783000
H	7.503429000	-2.300288000	-1.106956000
H	2.171564000	-3.859320000	0.633925000
H	4.340333000	-4.392864000	0.870858000
O	-0.044682000	-3.813052000	1.181803000

A29

C	-0.368486000	-2.647050000	0.442013000
C	0.933655000	-2.074372000	-0.023793000
H	-4.650339000	4.216849000	0.732222000
C	-1.986276000	2.145624000	1.057802000
C	-3.128891000	2.855178000	1.401216000
C	-3.761690000	3.662159000	0.462269000
C	-3.244498000	3.752961000	-0.823733000
C	-2.103192000	3.039199000	-1.167317000
C	-1.463449000	2.227385000	-0.233704000
H	-1.498334000	1.520162000	1.791491000
H	-3.523793000	2.780282000	2.405636000
H	-3.726263000	4.381814000	-1.560491000
H	-1.698018000	3.124176000	-2.167174000
N	-0.234076000	0.061475000	-0.221973000

C	0.914080000	-0.721236000	-0.687361000
C	-1.605280000	-1.957301000	-0.032646000
C	-1.455215000	-0.609330000	-0.686159000
C	-2.771584000	-2.596731000	0.180355000
C	-0.195167000	1.479466000	-0.619615000
P	1.196357000	2.510964000	0.086266000
O	1.284739000	2.225288000	1.659950000
O	2.536927000	1.882344000	-0.562374000
C	1.818214000	1.038975000	2.293650000
C	2.083436000	1.363469000	3.747453000
C	3.721528000	2.710634000	-0.726012000
C	4.270212000	2.525571000	-2.123522000
H	1.091209000	0.234178000	2.192199000
H	2.735776000	0.743275000	1.783642000
H	4.442190000	2.392356000	0.028288000
H	3.450865000	3.749271000	-0.543810000
H	5.183106000	3.112192000	-2.240088000
H	4.509566000	1.479793000	-2.314683000
H	3.550128000	2.862301000	-2.868775000
H	1.165876000	1.669422000	4.249047000
H	2.475716000	0.480989000	4.255201000
H	2.810768000	2.169579000	3.840321000
H	1.834701000	-0.195172000	-0.462445000
H	-1.436166000	-0.705521000	-1.788476000
O	1.016752000	3.947208000	-0.197175000

H	0.879015000	-0.825014000	-1.788882000
H	-2.316752000	0.002818000	-0.440300000
H	-0.061694000	1.583147000	-1.708932000
C	-4.502387000	-1.359732000	-1.202610000
C	-6.507454000	-2.565921000	0.298452000
C	-6.843044000	-1.676296000	-0.716900000
C	-5.835740000	-1.083501000	-1.469558000
H	-3.743391000	-0.911127000	-1.823796000
H	-7.283888000	-3.042080000	0.882166000
H	-7.880940000	-1.455861000	-0.927352000
H	-6.088979000	-0.405730000	-2.273794000
C	-4.141958000	-2.241548000	-0.171348000
C	-5.176915000	-2.851713000	0.557655000
H	-4.920407000	-3.551883000	1.342206000
H	-2.672096000	-3.524761000	0.733053000
C	2.030809000	-2.820209000	0.204805000
C	3.878054000	-1.799618000	-1.202039000
C	3.432008000	-2.604032000	-0.141908000
C	5.232993000	-1.667008000	-1.472440000
C	4.401210000	-3.282693000	0.616118000
C	5.754169000	-3.136292000	0.355628000
C	6.177034000	-2.324648000	-0.691834000
H	5.553508000	-1.055191000	-2.305268000
H	4.075791000	-3.926777000	1.422864000
H	6.479067000	-3.663215000	0.961564000

H	7.231667000	-2.216788000	-0.906649000
H	1.837697000	-3.729583000	0.764001000
H	3.166546000	-1.301610000	-1.842172000
O	-0.415418000	-3.651767000	1.135787000

A30

C	0.183995000	-2.099784000	0.134238000
C	1.383351000	-1.516443000	-0.549041000
H	-3.930861000	4.987370000	-1.923282000
C	-2.080630000	2.971311000	0.082898000
C	-3.118117000	3.804020000	-0.326787000
C	-3.126471000	4.336054000	-1.608368000
C	-2.088984000	4.030689000	-2.484165000
C	-1.058228000	3.198049000	-2.074090000
C	-1.038230000	2.657748000	-0.785849000
H	-2.085980000	2.580730000	1.090006000
H	-3.917030000	4.038468000	0.363888000
H	-2.081653000	4.444596000	-3.483878000
H	-0.256911000	2.957159000	-2.761531000
N	-0.013827000	0.440431000	-1.092008000
C	1.148327000	-0.333894000	-1.465140000
C	-1.162369000	-1.620325000	-0.311674000
C	-1.246246000	-0.316249000	-1.075554000
C	-2.203707000	-2.422054000	-0.011050000
C	0.113935000	1.724375000	-0.426599000

P	0.528963000	1.718753000	1.398827000
O	-0.438521000	0.571045000	1.966628000
O	1.991963000	1.032341000	1.440145000
C	-0.357361000	0.172245000	3.360415000
C	-1.398019000	-0.893428000	3.618647000
C	3.139105000	1.703255000	2.016740000
C	4.044032000	2.258621000	0.935900000
H	0.646823000	-0.211016000	3.548627000
H	-0.519006000	1.054235000	3.981455000
H	2.789128000	2.492031000	2.680367000
H	3.653862000	0.944476000	2.605475000
H	4.933462000	2.698495000	1.390960000
H	4.364098000	1.473736000	0.250807000
H	3.537846000	3.040296000	0.368333000
H	-1.208786000	-1.772592000	3.005005000
H	-1.361296000	-1.188531000	4.668757000
H	-2.398828000	-0.519510000	3.403274000
H	1.014185000	-0.686438000	-2.499948000
H	-1.543430000	-0.526269000	-2.114725000
O	0.483272000	3.025115000	2.097348000
H	2.022933000	0.314192000	-1.467086000
H	-2.034266000	0.302608000	-0.652037000
H	1.019900000	2.183930000	-0.833955000
C	-4.464917000	-3.275109000	0.329073000
C	-5.620835000	-1.371590000	-1.310757000

C	-6.423951000	-2.314489000	-0.679597000
C	-5.837392000	-3.270461000	0.142785000
H	-4.014011000	-4.024445000	0.966750000
H	-6.066800000	-0.629433000	-1.959523000
H	-7.494971000	-2.307421000	-0.831190000
H	-6.449819000	-4.013722000	0.635652000
C	-3.632266000	-2.323377000	-0.287570000
C	-4.246391000	-1.371482000	-1.120385000
H	-3.657680000	-0.633609000	-1.638639000
H	-1.929705000	-3.302699000	0.559283000
C	2.570763000	-2.085483000	-0.274834000
C	4.173959000	-1.184630000	-2.021038000
C	3.906543000	-1.787224000	-0.781559000
C	5.478188000	-0.937688000	-2.427543000
C	5.004371000	-2.156656000	0.013689000
C	6.305538000	-1.898558000	-0.386915000
C	6.548779000	-1.283018000	-1.610600000
H	5.659328000	-0.481930000	-3.391994000
H	4.820572000	-2.646966000	0.961002000
H	7.131603000	-2.185531000	0.250065000
H	7.563347000	-1.088013000	-1.930963000
H	2.531189000	-2.871549000	0.471767000
H	3.363372000	-0.942677000	-2.691001000
O	0.298423000	-2.967353000	0.989418000

A31

C	-0.009089000	-2.886185000	0.456479000
C	1.243385000	-2.228043000	-0.026731000
H	-4.772347000	3.699656000	0.536924000
C	-1.930258000	1.891992000	0.939292000
C	-3.120885000	2.530091000	1.261907000
C	-3.846175000	3.201560000	0.282957000
C	-3.373325000	3.226170000	-1.023171000
C	-2.183047000	2.584834000	-1.344640000
C	-1.447018000	1.913214000	-0.370324000
H	-1.371492000	1.370156000	1.702177000
H	-3.481930000	2.504917000	2.281601000
H	-3.931305000	3.740659000	-1.794176000
H	-1.820930000	2.607777000	-2.364510000
N	-0.073861000	-0.153824000	-0.245323000
C	1.160776000	-0.848108000	-0.624373000
C	-1.301812000	-2.243755000	0.059829000
C	-1.211606000	-0.955811000	-0.714999000
C	-2.425943000	-2.920714000	0.351190000
C	-0.140468000	1.232616000	-0.739924000
P	1.301470000	2.260090000	-0.146810000
O	0.807013000	3.710225000	-0.595123000
O	1.208045000	2.338386000	1.455672000
C	1.703396000	4.846164000	-0.508364000
C	1.062543000	6.007537000	-1.233902000

C	1.994983000	1.469869000	2.311888000
C	2.115280000	2.124120000	3.669966000
H	1.860926000	5.077372000	0.546382000
H	2.660492000	4.572113000	-0.952579000
H	2.970912000	1.316391000	1.852971000
H	1.483823000	0.508701000	2.380057000
H	1.132717000	2.296503000	4.108695000
H	2.681529000	1.475673000	4.340192000
H	2.634193000	3.079818000	3.597177000
H	0.099467000	6.258044000	-0.790315000
H	1.710362000	6.883160000	-1.171106000
H	0.908301000	5.769110000	-2.285977000
H	1.235817000	-0.908877000	-1.727113000
H	-1.095576000	-1.181768000	-1.792038000
O	2.637435000	1.860566000	-0.654226000
H	2.013287000	-0.262603000	-0.298916000
H	-2.121482000	-0.381778000	-0.620244000
H	-0.037080000	1.266505000	-1.837876000
C	-4.412238000	-1.348723000	0.145154000
C	-6.028359000	-3.522658000	-0.475543000
C	-6.576744000	-2.245906000	-0.429031000
C	-5.764780000	-1.164396000	-0.105799000
H	-3.814801000	-0.495840000	0.430540000
H	-6.652741000	-4.374514000	-0.710110000
H	-7.629458000	-2.096673000	-0.628311000

H	-6.185973000	-0.170003000	-0.041418000
C	-3.837624000	-2.625734000	0.082340000
C	-4.680512000	-3.709339000	-0.208364000
H	-4.261596000	-4.707213000	-0.232399000
H	-2.249697000	-3.880185000	0.827076000
C	2.377884000	-2.947537000	0.098853000
C	4.218039000	-1.600312000	-1.045987000
C	3.763398000	-2.680794000	-0.269172000
C	5.567963000	-1.448233000	-1.328320000
C	4.724464000	-3.600429000	0.190182000
C	6.071556000	-3.444615000	-0.090508000
C	6.500466000	-2.363106000	-0.852679000
H	5.890737000	-0.605929000	-1.925298000
H	4.394614000	-4.445501000	0.780594000
H	6.786337000	-4.166739000	0.281076000
H	7.551077000	-2.236805000	-1.077790000
H	2.228447000	-3.909054000	0.577762000
H	3.531696000	-0.870098000	-1.440315000
O	0.017652000	-3.911700000	1.120859000

A32

C	-0.939789000	2.552989000	-0.166054000
C	0.429876000	2.022178000	-0.459508000
H	0.823725000	-4.211934000	-4.584935000
C	-0.931975000	-3.028513000	-1.939724000

C	-0.674694000	-3.672656000	-3.143675000
C	0.618017000	-3.707463000	-3.650415000
C	1.647446000	-3.099989000	-2.941313000
C	1.386774000	-2.456445000	-1.737982000
C	0.090268000	-2.405935000	-1.221063000
H	-1.941237000	-3.019418000	-1.547701000
H	-1.482625000	-4.152186000	-3.680121000
H	2.659991000	-3.134891000	-3.321289000
H	2.201580000	-2.007266000	-1.188553000
N	-0.457614000	-0.216284000	-0.022712000
C	0.605143000	0.539843000	-0.681932000
C	-2.081535000	1.586531000	-0.251059000
C	-1.754173000	0.154097000	-0.589422000
C	-3.317150000	2.086838000	-0.090862000
C	-0.254776000	-1.673118000	0.070095000
P	0.770333000	-2.177413000	1.526291000
O	2.186511000	-1.421533000	1.328982000
O	0.085848000	-1.489467000	2.803025000
C	3.339115000	-1.874922000	2.089806000
C	4.587048000	-1.290539000	1.467758000
C	0.186629000	-0.103224000	3.204003000
C	-0.149133000	-0.013638000	4.676904000
H	3.222986000	-1.542374000	3.123379000
H	3.351609000	-2.964072000	2.079734000
H	1.196929000	0.255958000	3.005531000

H	-0.504002000	0.480030000	2.598004000
H	0.551953000	-0.595140000	5.275369000
H	-0.103177000	1.026994000	5.001907000
H	-1.154467000	-0.388609000	4.866980000
H	4.551193000	-0.201580000	1.457953000
H	5.460934000	-1.600612000	2.042836000
H	4.712319000	-1.640822000	0.443469000
H	1.563807000	0.226412000	-0.276569000
H	-2.514112000	-0.515481000	-0.201510000
O	0.853414000	-3.640577000	1.690796000
H	0.624276000	0.321012000	-1.763095000
H	-1.760328000	0.030240000	-1.686822000
H	-1.215584000	-2.073538000	0.403457000
C	-4.879152000	0.218491000	0.582534000
C	-6.927357000	1.406997000	-0.867193000
C	-7.164010000	0.218810000	-0.184865000
C	-6.138162000	-0.365578000	0.549301000
H	-4.104263000	-0.227539000	1.189951000
H	-7.722830000	1.877875000	-1.429269000
H	-8.143206000	-0.239809000	-0.212406000
H	-6.320486000	-1.274742000	1.106728000
C	-4.618731000	1.404618000	-0.115202000
C	-5.673994000	1.999934000	-0.819464000
H	-5.499626000	2.931948000	-1.341631000
H	-3.358002000	3.163593000	0.040165000

C	1.425322000	2.929492000	-0.487105000
C	3.714618000	3.770231000	-0.258054000
C	2.850934000	2.782392000	-0.760991000
C	5.082857000	3.706759000	-0.466806000
C	3.416540000	1.746236000	-1.520793000
C	4.785510000	1.692514000	-1.742867000
C	5.625737000	2.663760000	-1.210242000
H	5.725658000	4.475552000	-0.059281000
H	2.784444000	0.998319000	-1.973353000
H	5.197318000	0.891613000	-2.342472000
H	6.692206000	2.616146000	-1.384702000
H	1.113514000	3.937933000	-0.237224000
H	3.295043000	4.591055000	0.309218000
O	-1.123328000	3.726125000	0.120697000

A33

C	-0.829370000	-2.760436000	-0.154148000
C	0.569285000	-2.294702000	0.106159000
H	1.509924000	3.165974000	5.066623000
C	1.762260000	1.949331000	1.909365000
C	2.151279000	2.381496000	3.171584000
C	1.204042000	2.829046000	4.085195000
C	-0.137651000	2.849285000	3.725519000
C	-0.524884000	2.419400000	2.462100000
C	0.415082000	1.958327000	1.538293000

H	2.511193000	1.623889000	1.200073000
H	3.199756000	2.373817000	3.438956000
H	-0.883148000	3.204428000	4.424648000
H	-1.572052000	2.450639000	2.188051000
N	-0.298035000	0.001842000	0.060384000
C	0.806763000	-0.855025000	0.492253000
C	-1.938019000	-1.791564000	0.130281000
C	-1.547446000	-0.442671000	0.674877000
C	-3.194303000	-2.223140000	-0.060287000
C	-0.065492000	1.452879000	0.183649000
P	0.959016000	1.999314000	-1.250992000
O	0.021093000	1.779115000	-2.535129000
O	0.896688000	3.585758000	-1.041273000
C	0.302785000	0.784824000	-3.554275000
C	-0.897890000	-0.117325000	-3.735597000
C	1.652097000	4.473444000	-1.899006000
C	1.645601000	5.851683000	-1.276646000
H	0.522090000	1.336652000	-4.469166000
H	1.193795000	0.227219000	-3.270759000
H	2.666293000	4.085625000	-2.000410000
H	1.182140000	4.482304000	-2.884448000
H	2.198954000	6.546138000	-1.910626000
H	0.626803000	6.222794000	-1.166949000
H	2.114285000	5.832968000	-0.293291000
H	-1.780348000	0.460417000	-4.011774000

H	-0.699345000	-0.838320000	-4.530546000
H	-1.110219000	-0.665195000	-2.819066000
H	1.723423000	-0.500803000	0.024654000
H	-2.322319000	0.290868000	0.480242000
O	2.311516000	1.402315000	-1.377521000
H	0.946385000	-0.781990000	1.584118000
H	-1.449813000	-0.511006000	1.772995000
H	-1.028715000	1.924123000	-0.017553000
C	-4.701818000	-0.223692000	-0.386144000
C	-6.753327000	-1.548581000	0.935088000
C	-6.963115000	-0.263254000	0.448169000
C	-5.935972000	0.390974000	-0.222548000
H	-3.925955000	0.282845000	-0.943004000
H	-7.550390000	-2.072383000	1.445802000
H	-7.922876000	0.218668000	0.577135000
H	-6.098729000	1.380440000	-0.629087000
C	-4.468252000	-1.510582000	0.114880000
C	-5.525326000	-2.169416000	0.756754000
H	-5.372329000	-3.175452000	1.125858000
H	-3.277692000	-3.263756000	-0.357848000
C	1.536241000	-3.224174000	-0.032488000
C	3.754430000	-4.166878000	-0.441972000
C	2.980181000	-3.158343000	0.157761000
C	5.134354000	-4.185371000	-0.323447000
C	3.651610000	-2.186017000	0.916711000

C	5.032684000	-2.212960000	1.045084000
C	5.781111000	-3.204108000	0.420667000
H	5.705799000	-4.967905000	-0.804545000
H	3.097024000	-1.420678000	1.434907000
H	5.527151000	-1.456312000	1.639494000
H	6.858118000	-3.217416000	0.521037000
H	1.172649000	-4.189581000	-0.367402000
H	3.253906000	-4.937730000	-1.013652000
O	-1.066347000	-3.883262000	-0.573484000

A34

C	-0.383289000	-2.715582000	0.409809000
C	0.925699000	-2.135439000	-0.030421000
H	-4.581600000	4.205954000	0.807020000
C	-2.086081000	3.009162000	-1.148967000
C	-3.214518000	3.730756000	-0.780131000
C	-3.704612000	3.642874000	0.516842000
C	-3.057467000	2.830106000	1.441399000
C	-1.927984000	2.111500000	1.071935000
C	-1.432145000	2.191401000	-0.230479000
H	-1.703317000	3.091026000	-2.157712000
H	-3.707822000	4.363009000	-1.506152000
H	-3.428881000	2.759614000	2.455155000
H	-1.428432000	1.481426000	1.793684000
N	-0.219018000	0.022723000	-0.222341000

C	0.933222000	-0.766582000	-0.662452000
C	-1.617655000	-1.976497000	0.003832000
C	-1.432968000	-0.656216000	-0.697984000
C	-2.790623000	-2.598226000	0.219690000
C	-0.177894000	1.432950000	-0.642337000
P	1.237096000	2.470993000	0.002908000
O	1.357137000	2.234696000	1.582632000
O	2.557789000	1.810281000	-0.651616000
C	1.879016000	1.060172000	2.247216000
C	2.150945000	1.422042000	3.690916000
C	3.745110000	2.626178000	-0.860496000
C	3.819583000	3.105676000	-2.294218000
H	1.143397000	0.260227000	2.169901000
H	2.792679000	0.741606000	1.744102000
H	3.732324000	3.468909000	-0.170382000
H	4.584225000	1.978728000	-0.608963000
H	4.745075000	3.663849000	-2.448140000
H	3.808859000	2.263840000	-2.987067000
H	2.980088000	3.761483000	-2.518620000
H	1.237838000	1.750805000	4.186149000
H	2.536307000	0.550304000	4.221932000
H	2.886382000	2.223196000	3.759389000
H	0.932633000	-0.849787000	-1.766253000
H	-1.368024000	-0.825738000	-1.789459000
O	1.062859000	3.900477000	-0.316940000

H	1.851860000	-0.255940000	-0.398613000
H	-2.286978000	-0.015037000	-0.540994000
H	-0.074079000	1.520569000	-1.736562000
C	-5.066704000	-3.199343000	-0.476735000
C	-5.989195000	-0.599774000	-0.233510000
C	-6.854362000	-1.600268000	-0.662378000
C	-6.387578000	-2.904612000	-0.779517000
H	-4.712170000	-4.219077000	-0.555859000
H	-6.348235000	0.413638000	-0.113998000
H	-7.886212000	-1.367968000	-0.889238000
H	-7.055220000	-3.694565000	-1.096835000
C	-4.169246000	-2.196243000	-0.079991000
C	-4.662731000	-0.890860000	0.052203000
H	-4.024407000	-0.100816000	0.418504000
H	-2.692806000	-3.591573000	0.645959000
C	2.018956000	-2.888238000	0.197500000
C	3.895307000	-1.820840000	-1.136544000
C	3.426716000	-2.659872000	-0.113234000
C	5.255870000	-1.673576000	-1.368733000
C	4.380523000	-3.358807000	0.646199000
C	5.738691000	-3.198906000	0.423730000
C	6.183438000	-2.351973000	-0.586198000
H	5.592962000	-1.032506000	-2.172532000
H	4.038656000	-4.029037000	1.424324000
H	6.450984000	-3.742068000	1.030275000

H	7.242606000	-2.232676000	-0.770596000
H	1.816190000	-3.812717000	0.727636000
H	3.198649000	-1.305034000	-1.778437000
O	-0.438411000	-3.754626000	1.050107000

A35

C	-0.091864000	-3.008185000	0.263031000
C	1.177401000	-2.291686000	-0.070519000
H	-4.564521000	3.595199000	1.569945000
C	-1.728558000	1.742965000	1.384475000
C	-2.844430000	2.358784000	1.936164000
C	-3.695871000	3.116257000	1.138021000
C	-3.426237000	3.248969000	-0.218391000
C	-2.309197000	2.631354000	-0.769122000
C	-1.447342000	1.877401000	0.024225000
H	-1.070316000	1.147794000	2.000152000
H	-3.050052000	2.246821000	2.992571000
H	-4.087017000	3.827452000	-0.850317000
H	-2.109010000	2.733941000	-1.828141000
N	-0.137409000	-0.200517000	-0.182681000
C	1.128568000	-0.852235000	-0.513988000
C	-1.369144000	-2.306501000	-0.076672000
C	-1.245165000	-0.971118000	-0.757843000
C	-2.514219000	-2.959470000	0.179888000
C	-0.224155000	1.209396000	-0.583448000

P	1.314257000	2.198663000	-0.248194000
O	0.822896000	3.713715000	-0.457342000
O	1.485141000	2.033414000	1.333984000
C	1.499446000	4.642568000	-1.341051000
C	0.534276000	5.155916000	-2.388226000
C	2.616585000	2.620506000	2.022762000
C	2.690117000	2.023243000	3.409874000
H	1.860001000	5.456163000	-0.710884000
H	2.354771000	4.142712000	-1.792581000
H	2.466884000	3.701181000	2.065996000
H	3.521684000	2.414394000	1.450532000
H	2.837177000	0.945028000	3.359238000
H	3.527940000	2.460476000	3.955001000
H	1.775307000	2.223053000	3.967177000
H	-0.341978000	5.607028000	-1.923504000
H	1.027166000	5.912169000	-3.001725000
H	0.204226000	4.350491000	-3.044628000
H	1.325407000	-0.789539000	-1.599594000
H	-1.075702000	-1.127874000	-1.840601000
O	2.508263000	1.863084000	-1.063499000
H	1.935609000	-0.313791000	-0.026620000
H	-2.162107000	-0.402798000	-0.670780000
H	-0.294935000	1.302994000	-1.682371000
C	-4.402849000	-1.277688000	0.255538000
C	-6.147947000	-3.207383000	-0.719571000

C	-6.616999000	-1.927489000	-0.445093000
C	-5.741334000	-0.969669000	0.053704000
H	-3.748705000	-0.529460000	0.679090000
H	-6.823642000	-3.965093000	-1.093631000
H	-7.658689000	-1.683070000	-0.604516000
H	-6.100844000	0.021737000	0.294943000
C	-3.909594000	-2.556344000	-0.035347000
C	-4.814185000	-3.520684000	-0.502186000
H	-4.457048000	-4.522412000	-0.704418000
H	-2.379474000	-3.961926000	0.574134000
C	2.313321000	-3.010240000	0.047171000
C	4.195287000	-1.521173000	-0.822117000
C	3.714087000	-2.682791000	-0.191011000
C	5.558362000	-1.317585000	-0.986351000
C	4.663053000	-3.626304000	0.244935000
C	6.022441000	-3.417713000	0.083573000
C	6.477469000	-2.257074000	-0.533485000
H	5.900670000	-0.415428000	-1.475356000
H	4.313130000	-4.532688000	0.721946000
H	6.726772000	-4.160187000	0.434497000
H	7.538127000	-2.089496000	-0.665578000
H	2.151735000	-4.021161000	0.404883000
H	3.523488000	-0.768724000	-1.200312000
O	-0.089547000	-4.117397000	0.776044000

A36

C	-0.298316000	-3.008319000	0.316420000
C	1.009952000	-2.378994000	-0.042194000
H	-4.338853000	3.890407000	1.493972000
C	-2.175074000	2.732796000	-0.843297000
C	-3.242566000	3.435585000	-0.296116000
C	-3.508313000	3.346185000	1.064501000
C	-2.702745000	2.547533000	1.870286000
C	-1.635566000	1.847823000	1.322417000
C	-1.358541000	1.937132000	-0.042346000
H	-1.980763000	2.798299000	-1.906856000
H	-3.868544000	4.045471000	-0.933929000
H	-2.906234000	2.469501000	2.930153000
H	-1.012937000	1.221828000	1.944709000
N	-0.179210000	-0.221463000	-0.215962000
C	1.042353000	-0.956158000	-0.538350000
C	-1.534047000	-2.246311000	-0.047188000
C	-1.335359000	-0.938787000	-0.763377000
C	-2.714029000	-2.828994000	0.220674000
C	-0.187180000	1.181625000	-0.647601000
P	1.413231000	2.086488000	-0.359859000
O	1.039145000	3.640116000	-0.529346000
O	1.617193000	1.939881000	1.216701000
C	1.379942000	4.395724000	-1.716837000
C	1.888506000	5.759809000	-1.303405000

C	2.798190000	2.475548000	1.865933000
C	2.801834000	2.003656000	3.302099000
H	2.127487000	3.844400000	-2.285409000
H	0.472473000	4.484936000	-2.316685000
H	2.757470000	3.564368000	1.806286000
H	3.680272000	2.127931000	1.327452000
H	2.835595000	0.915910000	3.352640000
H	3.678387000	2.399886000	3.816810000
H	1.910539000	2.348239000	3.825726000
H	2.810433000	5.673047000	-0.728571000
H	2.094181000	6.361004000	-2.190633000
H	1.148884000	6.282854000	-0.697596000
H	1.228353000	-0.943529000	-1.627490000
H	-1.185192000	-1.132226000	-1.843060000
O	2.545500000	1.666035000	-1.221153000
H	1.886133000	-0.450041000	-0.079402000
H	-2.215270000	-0.313881000	-0.684064000
H	-0.269063000	1.253474000	-1.747980000
C	-5.037260000	-3.293290000	-0.449943000
C	-5.833775000	-0.679675000	0.006149000
C	-6.756038000	-1.609334000	-0.461010000
C	-6.352506000	-2.920861000	-0.685576000
H	-4.731294000	-4.318777000	-0.613204000
H	-6.143199000	0.336976000	0.208815000
H	-7.783258000	-1.317799000	-0.634499000

H	-7.064886000	-3.656497000	-1.034760000
C	-4.085448000	-2.360086000	-0.014496000
C	-4.513382000	-1.047645000	0.225885000
H	-3.823468000	-0.317830000	0.624169000
H	-2.635101000	-3.826152000	0.642453000
C	2.103274000	-3.155332000	0.105323000
C	4.062364000	-1.818045000	-0.835713000
C	3.520064000	-2.915135000	-0.142058000
C	5.434567000	-1.695220000	-1.004577000
C	4.417396000	-3.880215000	0.351745000
C	5.786207000	-3.751447000	0.186629000
C	6.302499000	-2.653428000	-0.493523000
H	5.824497000	-0.841813000	-1.542957000
H	4.019826000	-4.738964000	0.877145000
H	6.450107000	-4.508023000	0.583289000
H	7.370721000	-2.548992000	-0.629498000
H	1.885386000	-4.140595000	0.502570000
H	3.430422000	-1.055265000	-1.259927000
O	-0.359228000	-4.097540000	0.867203000

A37

C	0.357294000	2.818843000	0.389146000
C	-0.958853000	2.171116000	0.083868000
H	4.410693000	-3.962919000	1.769030000
C	1.765151000	-1.867606000	1.431194000

C	2.822208000	-2.539240000	2.032302000
C	3.588623000	-3.439281000	1.299422000
C	3.291892000	-3.662259000	-0.039743000
C	2.235147000	-2.989344000	-0.640545000
C	1.463450000	-2.086261000	0.086829000
H	1.170844000	-1.162444000	1.994171000
H	3.046781000	-2.360027000	3.075506000
H	3.881701000	-4.360727000	-0.618228000
H	2.003195000	-3.175558000	-1.681072000
N	0.247857000	0.040805000	-0.165057000
C	-0.987676000	0.761119000	-0.454424000
C	1.588044000	2.080471000	-0.030006000
C	1.383024000	0.770278000	-0.739217000
C	2.768534000	2.682172000	0.191486000
C	0.289794000	-1.368039000	-0.568065000
P	-1.221281000	-2.406191000	-0.269196000
O	-1.665488000	-1.960473000	1.200306000
O	-2.386252000	-1.816751000	-1.224141000
C	-2.686942000	-2.697789000	1.920138000
C	-2.621276000	-2.297700000	3.376573000
C	-2.975813000	-2.607352000	-2.288304000
C	-2.461798000	-2.163149000	-3.642278000
H	-3.656544000	-2.449276000	1.484607000
H	-2.505117000	-3.764369000	1.788132000
H	-2.758443000	-3.658164000	-2.107261000

H	-4.050819000	-2.448681000	-2.206081000
H	-2.972706000	-2.720234000	-4.429666000
H	-2.642631000	-1.100391000	-3.803789000
H	-1.392460000	-2.353140000	-3.737035000
H	-2.782409000	-1.226479000	3.493624000
H	-3.393581000	-2.825836000	3.937848000
H	-1.651460000	-2.552004000	3.802769000
H	-1.184540000	0.782150000	-1.542549000
H	1.205705000	0.960238000	-1.815456000
O	-0.990278000	-3.851103000	-0.487179000
H	-1.817070000	0.233849000	0.005600000
H	2.270023000	0.153269000	-0.680495000
H	0.402223000	-1.464635000	-1.662688000
C	4.593409000	0.929316000	0.127394000
C	6.366807000	2.829226000	-0.856517000
C	6.798668000	1.524329000	-0.646488000
C	5.909806000	0.581304000	-0.142816000
H	3.931715000	0.189692000	0.554342000
H	7.053936000	3.575071000	-1.233527000
H	7.822744000	1.248365000	-0.859458000
H	6.242355000	-0.430044000	0.049176000
C	4.136588000	2.234723000	-0.097830000
C	5.056148000	3.182100000	-0.570427000
H	4.728629000	4.202701000	-0.722187000
H	2.690898000	3.677080000	0.618701000

C	-2.053218000	2.919333000	0.329328000
C	-4.034301000	1.623917000	-0.610863000
C	-3.478084000	2.658223000	0.161022000
C	-5.410178000	1.467253000	-0.703376000
C	-4.366728000	3.532252000	0.812619000
C	-5.739388000	3.368375000	0.726656000
C	-6.269074000	2.330395000	-0.032663000
H	-5.814239000	0.667033000	-1.309304000
H	-3.959868000	4.346627000	1.398030000
H	-6.396754000	4.052974000	1.245835000
H	-7.340426000	2.201223000	-0.108385000
H	-1.828209000	3.891116000	0.754961000
H	-3.400948000	0.947168000	-1.160386000
O	0.422451000	3.904983000	0.945024000

A38

C	-0.597675000	-3.085286000	-0.100959000
C	0.761563000	-2.461752000	-0.083813000
H	-3.859324000	3.825647000	2.547527000
C	-2.103939000	2.794596000	-0.161824000
C	-3.066934000	3.466626000	0.584608000
C	-3.112560000	3.304794000	1.963192000
C	-2.195576000	2.464845000	2.588802000
C	-1.236344000	1.793748000	1.843092000
C	-1.178185000	1.957887000	0.457793000

H	-2.084439000	2.909900000	-1.238797000
H	-3.783051000	4.107375000	0.087388000
H	-2.226494000	2.334131000	3.662281000
H	-0.524650000	1.139477000	2.326494000
N	-0.230197000	-0.226050000	-0.133778000
C	0.892999000	-1.046558000	-0.589030000
C	-1.757277000	-2.160080000	-0.321397000
C	-1.467757000	-0.736937000	-0.727441000
C	-2.984105000	-2.693716000	-0.203303000
C	-0.123416000	1.223332000	-0.355538000
P	1.553027000	1.915942000	0.060755000
O	2.335992000	1.676397000	-1.329929000
O	1.381462000	3.506135000	0.167612000
C	3.783069000	1.775416000	-1.386459000
C	4.242707000	1.234345000	-2.721439000
C	1.076649000	4.401874000	-0.922489000
C	1.674454000	5.756792000	-0.608971000
H	4.066580000	2.823143000	-1.266183000
H	4.204949000	1.209628000	-0.556714000
H	1.479565000	3.995675000	-1.851221000
H	-0.007976000	4.469273000	-1.013666000
H	1.429728000	6.462393000	-1.404551000
H	1.277577000	6.143149000	0.329062000
H	2.759033000	5.694546000	-0.523465000
H	3.800710000	1.795636000	-3.544716000

H	5.328215000	1.313534000	-2.795494000
H	3.968293000	0.185697000	-2.829453000
H	0.937819000	-1.067983000	-1.694262000
H	-1.395788000	-0.697587000	-1.832632000
O	2.205130000	1.378101000	1.272176000
H	1.822783000	-0.618074000	-0.244719000
H	-2.287256000	-0.084875000	-0.452396000
H	-0.265448000	1.478689000	-1.419763000
C	-4.637796000	-0.842859000	0.275114000
C	-6.589939000	-2.228603000	-1.134500000
C	-6.896246000	-1.002595000	-0.554414000
C	-5.918332000	-0.319291000	0.159551000
H	-3.902795000	-0.314760000	0.865373000
H	-7.347461000	-2.776197000	-1.679365000
H	-7.892377000	-0.590616000	-0.645070000
H	-6.153355000	0.622512000	0.637144000
C	-4.307721000	-2.067484000	-0.319354000
C	-5.315308000	-2.760682000	-1.004501000
H	-5.086240000	-3.722701000	-1.444870000
H	-2.993570000	-3.764593000	-0.025812000
C	1.773684000	-3.234110000	0.346574000
C	4.134689000	-3.919504000	0.225366000
C	3.195260000	-2.929441000	0.548831000
C	5.492988000	-3.706868000	0.409215000
C	3.660782000	-1.733570000	1.113180000

C	5.020303000	-1.531550000	1.313470000
C	5.942127000	-2.508415000	0.953384000
H	6.199576000	-4.480858000	0.140164000
H	2.968847000	-0.965876000	1.428819000
H	5.354763000	-0.607719000	1.766657000
H	6.999908000	-2.344780000	1.111376000
H	1.492398000	-4.261282000	0.557221000
H	3.788601000	-4.860926000	-0.182176000
O	-0.763348000	-4.277895000	0.101451000

A39

C	0.696850000	-2.732442000	-0.479520000
C	-0.674044000	-2.205443000	-0.189829000
H	3.671115000	4.530697000	-2.062566000
C	1.437403000	2.047803000	-1.472788000
C	2.321328000	2.862460000	-2.170143000
C	2.980637000	3.898950000	-1.519849000
C	2.751627000	4.115099000	-0.165949000
C	1.868670000	3.300501000	0.530526000
C	1.203915000	2.259016000	-0.114124000
H	0.940318000	1.226567000	-1.968989000
H	2.498792000	2.682727000	-3.222339000
H	3.260450000	4.918687000	0.349570000
H	1.683951000	3.483733000	1.580853000
N	0.306977000	-0.012918000	0.210637000

C	-0.779020000	-0.916731000	0.587055000
C	1.852993000	-1.928832000	0.026768000
C	1.582452000	-0.584516000	0.648335000
C	3.075239000	-2.474241000	-0.129565000
C	0.219392000	1.382705000	0.653068000
P	-1.428836000	2.234752000	0.569219000
O	-2.115678000	1.776268000	-0.812954000
O	-2.257520000	1.465041000	1.711321000
C	-2.602796000	2.738208000	-1.788743000
C	-2.295298000	2.232364000	-3.180208000
C	-3.524023000	1.994650000	2.176754000
C	-3.911350000	1.256028000	3.438097000
H	-3.678520000	2.837734000	-1.635426000
H	-2.138390000	3.702477000	-1.591893000
H	-4.268821000	1.847161000	1.391716000
H	-3.410213000	3.063876000	2.354312000
H	-3.161892000	1.401482000	4.215675000
H	-4.866542000	1.632858000	3.806906000
H	-4.013405000	0.187948000	3.248711000
H	-2.744622000	1.254587000	-3.353113000
H	-2.698407000	2.928880000	-3.917434000
H	-1.220388000	2.153175000	-3.336667000
H	-0.749553000	-1.141624000	1.669240000
H	1.592609000	-0.665361000	1.752877000
O	-1.351896000	3.700520000	0.751427000

H	-1.731074000	-0.444530000	0.404671000
H	2.381992000	0.102177000	0.377538000
H	0.453652000	1.474341000	1.727535000
C	5.502577000	-2.567058000	-0.417657000
C	5.964904000	-0.655085000	1.530982000
C	7.036845000	-1.209717000	0.841250000
C	6.799818000	-2.171909000	-0.134979000
H	5.323031000	-3.322997000	-1.171194000
H	6.142197000	0.079651000	2.305059000
H	8.048591000	-0.902837000	1.069966000
H	7.627247000	-2.618683000	-0.669830000
C	4.401902000	-1.999227000	0.247129000
C	4.664163000	-1.040913000	1.239760000
H	3.855874000	-0.615615000	1.812915000
H	3.069215000	-3.424172000	-0.653050000
C	-1.706036000	-2.971184000	-0.582720000
C	-3.952905000	-3.890122000	-0.187242000
C	-3.155194000	-2.770262000	-0.464240000
C	-5.331257000	-3.781832000	-0.076742000
C	-3.796629000	-1.542215000	-0.675408000
C	-5.178147000	-1.439287000	-0.583290000
C	-5.950974000	-2.552858000	-0.272806000
H	-5.922773000	-4.658812000	0.150257000
H	-3.220312000	-0.670505000	-0.948304000
H	-5.655016000	-0.485134000	-0.765903000

H	-7.026875000	-2.466863000	-0.200204000
H	-1.413886000	-3.915511000	-1.031209000
H	-3.477113000	-4.852766000	-0.050250000
O	0.864373000	-3.776538000	-1.091757000

A40

C	0.079963000	-2.886267000	0.393258000
C	1.319495000	-2.165930000	-0.030465000
H	-4.874362000	3.531286000	0.753892000
C	-1.973790000	1.797707000	1.053073000
C	-3.180159000	2.384291000	1.413367000
C	-3.935859000	3.073686000	0.470626000
C	-3.477876000	3.167954000	-0.837878000
C	-2.271684000	2.578849000	-1.197150000
C	-1.505163000	1.891019000	-0.258378000
H	-1.390095000	1.260464000	1.785751000
H	-3.530009000	2.304101000	2.434137000
H	-4.060022000	3.696229000	-1.581217000
H	-1.920282000	2.657489000	-2.217927000
N	-0.066008000	-0.126209000	-0.204820000
C	1.213690000	-0.753693000	-0.543224000
C	-1.223158000	-2.266439000	-0.003218000
C	-1.148574000	-0.959672000	-0.746012000
C	-2.335441000	-2.973296000	0.259704000
C	-0.180472000	1.266456000	-0.664748000

P	1.234762000	2.342715000	-0.097958000
O	0.684331000	3.762277000	-0.587035000
O	1.142676000	2.419936000	1.502752000
C	1.527249000	4.937023000	-0.504532000
C	0.841344000	6.063191000	-1.244564000
C	2.198088000	1.952566000	2.382763000
C	1.667261000	0.877311000	3.306379000
H	1.666847000	5.186484000	0.549146000
H	2.499186000	4.703066000	-0.939614000
H	3.030574000	1.596511000	1.777951000
H	2.530935000	2.824555000	2.946374000
H	2.459029000	0.555520000	3.985082000
H	0.837037000	1.252647000	3.904703000
H	1.324154000	0.009540000	2.744160000
H	-0.134760000	6.275297000	-0.809501000
H	1.449852000	6.966843000	-1.185925000
H	0.704942000	5.808615000	-2.295301000
H	1.366427000	-0.739950000	-1.639093000
H	-0.971711000	-1.156898000	-1.820403000
O	2.584265000	1.990069000	-0.606043000
H	2.019522000	-0.161607000	-0.123428000
H	-2.084117000	-0.422456000	-0.684794000
H	-0.096777000	1.327247000	-1.763608000
C	-4.569082000	-3.783777000	-0.355389000
C	-5.700970000	-1.264106000	-0.169204000

C	-6.489613000	-2.347241000	-0.540983000
C	-5.917462000	-3.611332000	-0.629550000
H	-4.131648000	-4.772339000	-0.412278000
H	-6.140921000	-0.280573000	-0.071946000
H	-7.542960000	-2.209713000	-0.745338000
H	-6.523880000	-4.464816000	-0.902263000
C	-3.749692000	-2.696676000	-0.015525000
C	-4.347784000	-1.433424000	0.089121000
H	-3.768368000	-0.581987000	0.413537000
H	-2.144751000	-3.940723000	0.713598000
C	2.471216000	-2.863271000	0.061696000
C	4.296175000	-1.375471000	-0.926536000
C	3.852908000	-2.540447000	-0.274261000
C	5.644607000	-1.179494000	-1.189091000
C	4.824823000	-3.493373000	0.083828000
C	6.169887000	-3.294225000	-0.178370000
C	6.587071000	-2.131407000	-0.817432000
H	5.957666000	-0.272890000	-1.689258000
H	4.504630000	-4.400863000	0.579242000
H	6.892290000	-4.045188000	0.112545000
H	7.636144000	-1.970345000	-1.027062000
H	2.341584000	-3.858570000	0.472331000
H	3.605163000	-0.610547000	-1.238084000
O	0.127143000	-3.940626000	1.009559000

A41

C	-0.740407000	-2.998687000	0.075081000
C	0.648487000	-2.453309000	0.002885000
H	-3.616773000	4.012293000	2.729594000
C	-1.968977000	2.919815000	-0.022028000
C	-2.882148000	3.632318000	0.748627000
C	-2.906589000	3.462543000	2.126590000
C	-2.017873000	2.575865000	2.727096000
C	-1.108243000	1.864275000	1.957418000
C	-1.072563000	2.034243000	0.572405000
H	-1.965564000	3.042538000	-1.098512000
H	-3.577486000	4.309842000	0.270984000
H	-2.033603000	2.437935000	3.799992000
H	-0.420251000	1.172131000	2.422109000
N	-0.245911000	-0.189716000	-0.060488000
C	0.819216000	-1.063297000	-0.554017000
C	-1.856140000	-2.057053000	-0.262499000
C	-1.529997000	-0.627152000	-0.608933000
C	-3.101769000	-2.566695000	-0.203825000
C	-0.074582000	1.254722000	-0.270312000
P	1.643596000	1.861349000	0.109080000
O	2.375211000	1.611036000	-1.308143000
O	1.552208000	3.455683000	0.247577000
C	3.822285000	1.666552000	-1.405676000
C	4.225574000	1.138988000	-2.764007000

C	1.259021000	4.385433000	-0.816643000
C	1.912722000	5.710189000	-0.486149000
H	4.142047000	2.702268000	-1.272782000
H	4.249889000	1.070834000	-0.600181000
H	0.176134000	4.494770000	-0.884320000
H	1.628322000	3.985191000	-1.761901000
H	1.679701000	6.441657000	-1.261632000
H	1.547818000	6.090072000	0.467357000
H	2.995643000	5.606273000	-0.422885000
H	3.778997000	1.731378000	-3.562670000
H	5.310532000	1.184185000	-2.868311000
H	3.913355000	0.102336000	-2.884578000
H	1.782906000	-0.668465000	-0.267353000
H	-1.527158000	-0.499401000	-1.709918000
O	2.305035000	1.272110000	1.291049000
H	0.808287000	-1.111752000	-1.659078000
H	-2.312513000	0.021634000	-0.220724000
H	-0.229299000	1.527153000	-1.328789000
C	-5.525764000	-2.570063000	0.135728000
C	-5.908770000	-0.342381000	-1.463123000
C	-6.999034000	-0.940170000	-0.841751000
C	-6.801804000	-2.060866000	-0.041647000
H	-5.378355000	-3.448415000	0.750723000
H	-6.056175000	0.516673000	-2.104169000
H	-7.994675000	-0.543769000	-0.989015000

H	-7.644336000	-2.541868000	0.437023000
C	-4.405689000	-1.963497000	-0.458093000
C	-4.628415000	-0.843463000	-1.274601000
H	-3.805872000	-0.379571000	-1.795553000
H	-3.138552000	-3.600960000	0.121030000
C	1.641795000	-3.268819000	0.394332000
C	3.949876000	-4.080920000	0.118988000
C	3.088360000	-3.038746000	0.491617000
C	5.326818000	-3.939157000	0.207325000
C	3.652947000	-1.863281000	1.005553000
C	5.031929000	-1.732451000	1.110895000
C	5.874433000	-2.760895000	0.703377000
H	5.971825000	-4.752476000	-0.098026000
H	3.024259000	-1.057491000	1.357192000
H	5.445096000	-0.823905000	1.528860000
H	6.947672000	-2.652638000	0.787668000
H	1.318638000	-4.270783000	0.659278000
H	3.527512000	-5.006854000	-0.250460000
O	-0.961052000	-4.154739000	0.403092000

A42

C	0.900824000	-2.956550000	-0.260878000
C	-0.509989000	-2.486068000	-0.117321000
H	3.266685000	4.441174000	-2.425526000
C	1.098954000	1.929567000	-1.719296000

C	1.920922000	2.765874000	-2.464123000
C	2.623394000	3.794078000	-1.844309000
C	2.499797000	3.981247000	-0.472538000
C	1.677621000	3.143629000	0.272507000
C	0.970500000	2.111510000	-0.341719000
H	0.558240000	1.123958000	-2.195174000
H	2.014262000	2.612738000	-3.531066000
H	3.048125000	4.773588000	0.019606000
H	1.584597000	3.293232000	1.340930000
N	0.284791000	-0.200926000	0.135461000
C	-0.730942000	-1.161713000	0.566649000
C	1.979839000	-2.001710000	0.153336000
C	1.596483000	-0.623336000	0.627220000
C	3.245112000	-2.451775000	0.045867000
C	0.054849000	1.208282000	0.471313000
P	-1.698691000	1.771385000	0.221994000
O	-2.350094000	1.342095000	1.623577000
O	-1.655176000	3.369416000	0.374275000
C	-3.760285000	1.560188000	1.878226000
C	-4.106365000	0.913965000	3.200665000
C	-1.729611000	4.260294000	-0.769850000
C	-2.506837000	5.494962000	-0.369692000
H	-3.942739000	2.635756000	1.903608000
H	-4.335334000	1.126931000	1.059444000
H	-0.709089000	4.511746000	-1.057904000

H	-2.208343000	3.732517000	-1.593192000
H	-2.534727000	6.196773000	-1.204811000
H	-3.532658000	5.242375000	-0.101293000
H	-2.038298000	5.992113000	0.479628000
H	-3.515303000	1.344117000	4.008945000
H	-5.162269000	1.074173000	3.423379000
H	-3.921951000	-0.159210000	3.167896000
H	-0.696109000	-1.307126000	1.662259000
H	1.606258000	-0.590543000	1.734574000
O	-2.384689000	1.299115000	-1.004208000
H	-1.716804000	-0.786398000	0.334692000
H	2.341517000	0.093539000	0.286749000
H	0.237612000	1.406403000	1.540308000
C	4.707345000	-0.754658000	1.243105000
C	6.923143000	-1.797878000	-0.071710000
C	7.079196000	-0.730642000	0.806804000
C	5.968166000	-0.220349000	1.468155000
H	3.869236000	-0.360425000	1.795386000
H	7.782582000	-2.211536000	-0.582200000
H	8.059396000	-0.308505000	0.983313000
H	6.083970000	0.595833000	2.168895000
C	4.525354000	-1.820367000	0.347311000
C	5.666783000	-2.340214000	-0.287070000
H	5.551335000	-3.177244000	-0.963511000
H	3.321918000	-3.451483000	-0.367868000

C	-1.473970000	-3.307747000	-0.565650000
C	-3.733537000	-4.256740000	-0.340389000
C	-2.931313000	-3.139228000	-0.614479000
C	-5.117345000	-4.175039000	-0.385783000
C	-3.564137000	-1.945022000	-0.986917000
C	-4.949693000	-1.872156000	-1.049632000
C	-5.732287000	-2.978937000	-0.739686000
H	-5.715256000	-5.047546000	-0.157756000
H	-2.984984000	-1.075011000	-1.262007000
H	-5.415518000	-0.944685000	-1.355464000
H	-6.811245000	-2.915395000	-0.789372000
H	-1.111625000	-4.265118000	-0.927143000
H	-3.258834000	-5.194608000	-0.081163000
O	1.167503000	-4.064032000	-0.702767000

A43

C	0.717396000	-3.079383000	-0.092999000
C	-0.663956000	-2.507769000	-0.031065000
H	3.674493000	4.189875000	-2.127301000
C	1.306014000	1.827999000	-1.561912000
C	2.220967000	2.611393000	-2.254641000
C	2.960617000	3.582412000	-1.587095000
C	2.780391000	3.765076000	-0.220857000
C	1.864589000	2.981044000	0.471612000
C	1.119490000	2.006732000	-0.190128000

H	0.734863000	1.066726000	-2.073899000
H	2.356095000	2.463041000	-3.317817000
H	3.356421000	4.512815000	0.308025000
H	1.728869000	3.126146000	1.535880000
N	0.257630000	-0.256294000	0.215809000
C	-0.838252000	-1.147560000	0.596979000
C	1.848358000	-2.142051000	0.212349000
C	1.511320000	-0.779776000	0.763258000
C	3.091331000	-2.617079000	0.029471000
C	0.104798000	1.160492000	0.563905000
P	-1.594195000	1.840848000	0.236082000
O	-2.336546000	1.448165000	1.602025000
O	-1.449662000	3.431309000	0.404747000
C	-3.742022000	1.747972000	1.791110000
C	-4.175793000	1.157711000	3.113808000
C	-1.410142000	4.333783000	-0.731736000
C	-2.140478000	5.606379000	-0.363116000
H	-3.867668000	2.831953000	1.781484000
H	-4.305494000	1.323161000	0.959986000
H	-0.362426000	4.530994000	-0.958223000
H	-1.868713000	3.840489000	-1.587413000
H	-2.082805000	6.315186000	-1.190808000
H	-3.192078000	5.408149000	-0.155856000
H	-1.694938000	6.070527000	0.516715000
H	-3.596589000	1.579116000	3.935133000

H	-5.230093000	1.378215000	3.287025000
H	-4.046923000	0.075938000	3.116485000
H	-1.781933000	-0.719005000	0.292724000
H	1.434453000	-0.856261000	1.865638000
O	-2.251210000	1.422570000	-1.025064000
H	-0.880694000	-1.266396000	1.695754000
H	2.308166000	-0.074824000	0.561832000
H	0.245013000	1.333485000	1.643381000
C	5.437069000	-2.699744000	0.780782000
C	5.942644000	-0.117775000	-0.087858000
C	6.954376000	-0.849210000	0.524121000
C	6.696694000	-2.145470000	0.955881000
H	5.246433000	-3.714894000	1.104797000
H	6.139444000	0.882193000	-0.450823000
H	7.938568000	-0.418288000	0.650033000
H	7.480519000	-2.729568000	1.419598000
C	4.395552000	-1.963174000	0.199179000
C	4.677013000	-0.665393000	-0.247309000
H	3.916002000	-0.092783000	-0.757959000
H	3.134238000	-3.662846000	-0.258510000
C	-1.657701000	-3.277750000	-0.506314000
C	-3.986218000	-4.063741000	-0.392338000
C	-3.094459000	-3.011433000	-0.647015000
C	-5.356334000	-3.890414000	-0.521022000
C	-3.621457000	-1.788817000	-1.086524000

C	-4.992794000	-1.624416000	-1.232134000
C	-5.866135000	-2.666493000	-0.940591000
H	-6.024985000	-4.713477000	-0.306118000
H	-2.968984000	-0.968087000	-1.348346000
H	-5.375402000	-0.676765000	-1.587400000
H	-6.933568000	-2.531585000	-1.054693000
H	-1.345707000	-4.271587000	-0.811974000
H	-3.592785000	-5.023385000	-0.081710000
O	0.921227000	-4.238068000	-0.419751000

A44

C	-0.244479000	-2.907921000	0.383189000
C	1.049732000	-2.289196000	-0.032823000
H	-4.284377000	3.845169000	1.859851000
C	-1.590326000	1.815373000	1.503689000
C	-2.641966000	2.482781000	2.117252000
C	-3.464421000	3.327918000	1.379715000
C	-3.231657000	3.496531000	0.020513000
C	-2.180022000	2.826096000	-0.592861000
C	-1.347100000	1.983881000	0.140369000
H	-0.955870000	1.151658000	2.072599000
H	-2.821495000	2.340886000	3.174790000
H	-3.871327000	4.143942000	-0.564595000
H	-2.006378000	2.958386000	-1.653407000
N	-0.163341000	-0.159217000	-0.168428000

C	1.042965000	-0.881242000	-0.566145000
C	-1.491568000	-2.207066000	-0.054080000
C	-1.345714000	-0.850526000	-0.687743000
C	-2.655753000	-2.844580000	0.167482000
C	-0.193105000	1.261742000	-0.537398000
P	1.413306000	2.154631000	-0.255922000
O	0.997504000	3.699820000	-0.394458000
O	1.655647000	1.924429000	1.308683000
C	1.704217000	4.627602000	-1.254735000
C	0.715033000	5.344979000	-2.147913000
C	2.856010000	2.411249000	1.956824000
C	2.941117000	1.783400000	3.329790000
H	2.218843000	5.331474000	-0.599303000
H	2.448066000	4.080179000	-1.831279000
H	3.718267000	2.148397000	1.343136000
H	2.791719000	3.499249000	2.024307000
H	3.832542000	2.144130000	3.845089000
H	2.068621000	2.041985000	3.929151000
H	3.001594000	0.698272000	3.255212000
H	-0.051992000	5.844871000	-1.557191000
H	1.236186000	6.096157000	-2.743999000
H	0.227828000	4.648111000	-2.830074000
H	1.155308000	-0.885610000	-1.665983000
H	-1.282553000	-0.934719000	-1.790181000
O	2.540959000	1.779829000	-1.145583000

H	1.910303000	-0.359499000	-0.175566000
H	-2.226605000	-0.252302000	-0.468456000
H	-0.310889000	1.382224000	-1.629700000
C	-4.385643000	-1.629289000	-1.229552000
C	-6.389301000	-2.709003000	0.364963000
C	-6.723148000	-1.859374000	-0.684774000
C	-5.716781000	-1.330516000	-1.484581000
H	-3.626018000	-1.235803000	-1.887222000
H	-7.165673000	-3.136711000	0.985235000
H	-7.759300000	-1.622295000	-0.885650000
H	-5.969382000	-0.687337000	-2.317115000
C	-4.027153000	-2.470051000	-0.164663000
C	-5.061420000	-3.018596000	0.611566000
H	-4.806781000	-3.688899000	1.422393000
H	-2.554691000	-3.780914000	0.705872000
C	2.149275000	-3.059028000	0.095089000
C	4.061533000	-1.747007000	-0.967411000
C	3.554022000	-2.824312000	-0.219220000
C	5.424412000	-1.625468000	-1.199823000
C	4.475172000	-3.772528000	0.262518000
C	5.835045000	-3.645783000	0.033187000
C	6.317229000	-2.566927000	-0.700430000
H	5.787658000	-0.786789000	-1.778509000
H	4.104006000	-4.616451000	0.829642000
H	6.518320000	-4.389302000	0.421579000

H	7.378023000	-2.464357000	-0.886645000
H	1.950645000	-4.031823000	0.531319000
H	3.407611000	-0.998651000	-1.383770000
O	-0.286065000	-3.957505000	1.008150000

A45

C	0.894037000	-2.227653000	-0.329249000
C	-0.480080000	-1.797133000	-0.751573000
H	-1.699316000	6.208206000	-1.886082000
C	-0.264363000	4.048191000	0.302324000
C	-0.765235000	5.237895000	-0.209787000
C	-1.311042000	5.280107000	-1.488126000
C	-1.344625000	4.121052000	-2.251764000
C	-0.840034000	2.929383000	-1.741043000
C	-0.299784000	2.874715000	-0.457844000
H	0.144177000	4.023873000	1.302629000
H	-0.724233000	6.135927000	0.392511000
H	-1.757790000	4.141505000	-3.251826000
H	-0.846409000	2.040658000	-2.354008000
N	0.411832000	0.504381000	-0.867065000
C	-0.670018000	-0.376312000	-1.246992000
C	2.042119000	-1.319023000	-0.650326000
C	1.712568000	0.021544000	-1.270180000
C	3.273412000	-1.752755000	-0.324586000
C	0.287561000	1.570248000	0.097807000

P	-0.531200000	1.137716000	1.722198000
O	-2.061938000	0.770338000	1.375454000
O	0.158040000	-0.273081000	2.029199000
C	-3.190932000	1.431760000	2.004120000
C	-4.112370000	1.993784000	0.942974000
C	-0.140246000	-0.984745000	3.259133000
C	1.011106000	-1.913409000	3.573277000
H	-3.698233000	0.674256000	2.602551000
H	-2.816233000	2.208099000	2.668806000
H	-0.293177000	-0.257391000	4.057477000
H	-1.066059000	-1.540845000	3.103549000
H	0.787977000	-2.473654000	4.483049000
H	1.172220000	-2.620006000	2.760522000
H	1.927937000	-1.347167000	3.736201000
H	-4.459783000	1.207646000	0.272590000
H	-4.984114000	2.446270000	1.419435000
H	-3.608884000	2.759538000	0.353500000
H	-1.609258000	0.017815000	-0.867701000
H	2.470518000	0.756244000	-1.003664000
O	-0.396166000	2.160089000	2.787575000
H	-0.750483000	-0.384492000	-2.345882000
H	1.735307000	-0.055829000	-2.368414000
H	1.298502000	1.800832000	0.443288000
C	5.611562000	-1.457995000	0.365747000
C	6.140755000	0.320570000	-1.691556000

C	7.141383000	0.002474000	-0.780779000
C	6.871774000	-0.893848000	0.248531000
H	5.409778000	-2.162724000	1.162153000
H	6.348979000	0.997265000	-2.509659000
H	8.126470000	0.438083000	-0.880013000
H	7.647351000	-1.158714000	0.954719000
C	4.576910000	-1.124456000	-0.523590000
C	4.874282000	-0.233597000	-1.566504000
H	4.126088000	-0.001768000	-2.309168000
H	3.296798000	-2.703957000	0.196480000
C	-1.453512000	-2.721431000	-0.640539000
C	-3.482265000	-1.738382000	-1.817880000
C	-2.876626000	-2.670435000	-0.958927000
C	-4.848180000	-1.774339000	-2.060625000
C	-3.701783000	-3.649673000	-0.379051000
C	-5.067133000	-3.678107000	-0.612834000
C	-5.648787000	-2.735965000	-1.454660000
H	-5.288716000	-1.050358000	-2.733324000
H	-3.253067000	-4.391514000	0.268955000
H	-5.677368000	-4.439213000	-0.145116000
H	-6.713201000	-2.758174000	-1.646025000
H	-1.125632000	-3.661348000	-0.209722000
H	-2.885841000	-0.998196000	-2.326106000
O	1.076084000	-3.300108000	0.230493000

B1

C	-0.832867000	-2.875061000	0.110619000
C	0.538100000	-2.338694000	-0.172968000
H	3.111947000	2.003840000	4.905232000
C	1.316161000	2.742614000	2.122892000
C	2.129227000	2.931105000	3.232529000
C	2.474700000	1.856408000	4.043516000
C	1.989235000	0.593217000	3.736201000
C	1.168424000	0.404410000	2.629664000
C	0.826702000	1.473655000	1.802059000
H	1.061839000	3.594327000	1.509433000
H	2.494497000	3.923615000	3.461982000
H	2.242937000	-0.253709000	4.360122000
H	0.775847000	-0.577067000	2.412062000
N	-0.373458000	-0.143841000	0.330759000
C	0.690801000	-0.852473000	-0.375070000
C	-1.984458000	-1.927550000	-0.031305000
C	-1.684401000	-0.463571000	-0.226250000
C	-3.217698000	-2.461073000	0.049157000
C	-0.137015000	1.259781000	0.629383000
P	0.211018000	2.273575000	-0.908923000
O	-0.109790000	3.813936000	-0.531784000
O	1.808569000	2.247877000	-1.020364000
C	-1.183677000	4.560811000	-1.158476000
C	-2.283473000	4.852258000	-0.158525000

C	2.480711000	2.906493000	-2.121998000
C	3.959737000	2.609588000	-2.019015000
H	-0.731445000	5.483945000	-1.520911000
H	-1.553310000	3.993852000	-2.011136000
H	2.060496000	2.539633000	-3.059155000
H	2.289923000	3.979012000	-2.051308000
H	4.492449000	3.102258000	-2.833761000
H	4.362625000	2.970812000	-1.073456000
H	4.145370000	1.538037000	-2.084636000
H	-1.892651000	5.380547000	0.710908000
H	-3.047436000	5.477454000	-0.624149000
H	-2.762558000	3.933516000	0.180911000
H	0.681820000	-0.602339000	-1.448421000
H	-1.742373000	-0.180947000	-1.286782000
O	-0.530915000	1.807202000	-2.106800000
H	1.650987000	-0.525056000	0.020074000
H	-2.435047000	0.127149000	0.300552000
H	-1.104234000	1.669003000	0.938733000
C	-5.617908000	-2.483728000	0.548650000
C	-6.076409000	-0.125408000	-0.828741000
C	-7.131285000	-0.765880000	-0.188799000
C	-6.896395000	-1.951967000	0.499152000
H	-5.442302000	-3.412430000	1.076117000
H	-6.254774000	0.783649000	-1.387793000
H	-8.129681000	-0.352332000	-0.237325000

H	-7.712267000	-2.466199000	0.989448000
C	-4.532048000	-1.836048000	-0.063991000
C	-4.792499000	-0.649423000	-0.767947000
H	-4.000062000	-0.152075000	-1.305468000
H	-3.233321000	-3.523446000	0.266954000
C	1.538403000	-3.238998000	-0.206813000
C	3.520933000	-2.027417000	-1.222767000
C	2.963325000	-3.077903000	-0.477307000
C	4.889835000	-1.958335000	-1.440493000
C	3.834717000	-4.063733000	0.015359000
C	5.202674000	-3.985768000	-0.190211000
C	5.737763000	-2.928746000	-0.919015000
H	5.296521000	-1.145870000	-2.028085000
H	3.421406000	-4.894304000	0.572954000
H	5.851582000	-4.753442000	0.209786000
H	6.804258000	-2.868638000	-1.089555000
H	1.233431000	-4.251279000	0.035834000
H	2.882088000	-1.279294000	-1.665630000
O	-1.007551000	-4.047809000	0.406207000

B2

C	1.106278000	2.668223000	0.125185000
C	-0.289229000	2.232221000	-0.203881000
H	-2.807915000	-1.926514000	5.188158000
C	-1.180889000	-0.418795000	2.619903000

C	-1.926789000	-0.557868000	3.785757000
C	-2.230523000	-1.819294000	4.279568000
C	-1.774064000	-2.943467000	3.600651000
C	-1.033495000	-2.807737000	2.433514000
C	-0.735845000	-1.540018000	1.921863000
H	-0.920048000	0.566983000	2.267397000
H	-2.262784000	0.326753000	4.310480000
H	-1.989234000	-3.933033000	3.981906000
H	-0.688819000	-3.690226000	1.912871000
N	0.445235000	-0.028131000	0.318309000
C	-0.550411000	0.760052000	-0.397759000
C	2.189093000	1.637177000	0.030975000
C	1.785450000	0.202882000	-0.201836000
C	3.452497000	2.072368000	0.192619000
C	0.114399000	-1.402230000	0.655014000
P	-0.579824000	-2.472605000	-0.721121000
O	-2.063341000	-1.935003000	-1.051347000
O	0.285464000	-1.943327000	-1.968194000
C	-3.252497000	-2.751392000	-0.871894000
C	-4.200533000	-2.086533000	0.102324000
C	0.216294000	-2.632636000	-3.244840000
C	1.355907000	-3.619339000	-3.392287000
H	-2.949857000	-3.740778000	-0.533600000
H	-3.705503000	-2.846176000	-1.859392000
H	-0.748988000	-3.132090000	-3.337470000

H	0.265270000	-1.847671000	-3.998030000
H	1.311650000	-4.087839000	-4.377317000
H	2.317703000	-3.115032000	-3.297765000
H	1.288567000	-4.396501000	-2.633093000
H	-3.754052000	-2.010689000	1.092832000
H	-4.471273000	-1.085941000	-0.234306000
H	-5.113844000	-2.679174000	0.182270000
H	-0.554484000	0.506977000	-1.470415000
H	2.478972000	-0.453864000	0.323015000
O	-0.521194000	-3.935659000	-0.482259000
H	-1.538473000	0.509474000	-0.018519000
H	1.850089000	-0.054339000	-1.271165000
H	1.060722000	-1.910643000	0.856291000
C	4.938475000	0.156248000	-0.551486000
C	7.047930000	1.288107000	0.860485000
C	7.237702000	0.093533000	0.173372000
C	6.181133000	-0.461873000	-0.539151000
H	4.146141000	-0.275851000	-1.142857000
H	7.866737000	1.735769000	1.407772000
H	8.203597000	-0.393289000	0.182761000
H	6.326709000	-1.378488000	-1.095243000
C	4.721660000	1.352639000	0.150378000
C	5.811458000	1.912803000	0.837736000
H	5.671481000	2.846886000	1.366266000
H	3.534266000	3.128053000	0.427891000

C	-1.216375000	3.205132000	-0.290314000
C	-3.261669000	2.116934000	-1.329316000
C	-2.638042000	3.152739000	-0.614851000
C	-4.622122000	2.160131000	-1.601007000
C	-3.434631000	4.237070000	-0.208835000
C	-4.795057000	4.271737000	-0.468600000
C	-5.396718000	3.229645000	-1.166226000
H	-5.078505000	1.356487000	-2.163674000
H	-2.968583000	5.055689000	0.324295000
H	-5.385284000	5.114820000	-0.135159000
H	-6.456867000	3.256552000	-1.379472000
H	-0.842916000	4.195186000	-0.051986000
H	-2.683242000	1.287183000	-1.703799000
O	1.356965000	3.827308000	0.419968000

B3

C	1.245405000	2.612072000	0.070935000
C	-0.173580000	2.223491000	-0.211842000
H	-2.813521000	-1.555087000	5.355314000
C	-1.052005000	-0.258799000	2.760563000
C	-1.788620000	-0.305437000	3.939624000
C	-2.242913000	-1.519031000	4.436844000
C	-1.948375000	-2.689239000	3.746388000
C	-1.215995000	-2.645356000	2.567023000
C	-0.764004000	-1.424999000	2.054202000

H	-0.677598000	0.687051000	2.400777000
H	-1.999924000	0.613308000	4.470801000
H	-2.283991000	-3.644471000	4.128423000
H	-0.993389000	-3.564669000	2.042943000
N	0.480251000	-0.044313000	0.384193000
C	-0.501836000	0.757265000	-0.334990000
C	2.283062000	1.534200000	-0.011570000
C	1.815732000	0.109301000	-0.172849000
C	3.567224000	1.925366000	0.087331000
C	0.093575000	-1.386341000	0.784436000
P	-0.620711000	-2.491356000	-0.550800000
O	-2.091956000	-1.893727000	-0.797826000
O	0.240315000	-2.049811000	-1.841729000
C	-3.232924000	-2.729921000	-1.119372000
C	-4.414235000	-2.300458000	-0.276990000
C	0.677890000	-3.010858000	-2.835444000
C	-0.280730000	-3.071773000	-4.007308000
H	-3.438087000	-2.600244000	-2.183393000
H	-2.968844000	-3.770695000	-0.937704000
H	1.659909000	-2.662930000	-3.153489000
H	0.785116000	-3.983804000	-2.358125000
H	0.120851000	-3.734894000	-4.775718000
H	-1.251765000	-3.461959000	-3.702216000
H	-0.423776000	-2.084621000	-4.446530000
H	-4.651503000	-1.249713000	-0.442463000

H	-5.289512000	-2.896671000	-0.541063000
H	-4.205345000	-2.443683000	0.782447000
H	-0.549988000	0.460382000	-1.395464000
H	1.847168000	-0.192548000	-1.231945000
O	-0.583534000	-3.951552000	-0.286796000
H	-1.486454000	0.564347000	0.085582000
H	2.493198000	-0.553662000	0.365331000
H	1.020311000	-1.920090000	1.013843000
C	5.946752000	1.719667000	0.632849000
C	6.174411000	-0.742972000	-0.613897000
C	7.281492000	-0.182895000	0.013225000
C	7.162715000	1.055613000	0.635341000
H	5.861801000	2.687449000	1.109926000
H	6.263901000	-1.695583000	-1.119062000
H	8.231003000	-0.700973000	0.006734000
H	8.020803000	1.506471000	1.115723000
C	4.807771000	1.158564000	0.031050000
C	4.952696000	-0.084479000	-0.605707000
H	4.119090000	-0.524830000	-1.130339000
H	3.696282000	2.986290000	0.272272000
C	-1.059483000	3.231570000	-0.326118000
C	-3.175998000	2.184434000	-1.260694000
C	-2.488269000	3.228934000	-0.621544000
C	-4.538514000	2.277382000	-1.507997000
C	-3.223670000	4.372683000	-0.266048000

C	-4.586199000	4.458289000	-0.501836000
C	-5.251789000	3.407189000	-1.123724000
H	-5.045079000	1.464636000	-2.011601000
H	-2.707982000	5.197433000	0.208726000
H	-5.128419000	5.347184000	-0.208242000
H	-6.314041000	3.472629000	-1.317274000
H	-0.638492000	4.213979000	-0.141569000
H	-2.645844000	1.306887000	-1.595041000
O	1.550863000	3.769197000	0.318071000

B4

C	-0.349543000	-2.947725000	0.476874000
C	-1.559709000	-2.219839000	-0.017611000
H	-4.041555000	3.974884000	1.884486000
C	-1.546392000	1.721677000	1.437290000
C	-2.545439000	2.444376000	2.077973000
C	-3.260214000	3.415939000	1.387087000
C	-2.974107000	3.653936000	0.047727000
C	-1.975913000	2.929793000	-0.591241000
C	-1.245650000	1.961314000	0.096410000
H	-1.007770000	0.945658000	1.962332000
H	-2.771227000	2.241303000	3.116540000
H	-3.533681000	4.398663000	-0.502736000
H	-1.761074000	3.118904000	-1.635080000
N	-0.155115000	-0.227881000	-0.186615000

C	-1.342828000	-0.905004000	-0.714200000
C	0.974998000	-2.394047000	0.068016000
C	1.041946000	-1.003070000	-0.506686000
C	2.039274000	-3.205301000	0.236590000
C	-0.145433000	1.179382000	-0.605756000
P	1.516766000	2.008134000	-0.430858000
O	1.119233000	3.551106000	-0.637612000
O	1.985360000	1.862260000	1.092359000
C	2.039589000	4.485130000	-1.260295000
C	1.238467000	5.512647000	-2.028475000
C	1.726001000	2.756446000	2.198521000
C	3.026548000	3.037153000	2.921345000
H	2.623561000	4.956255000	-0.467467000
H	2.717474000	3.931256000	-1.908013000
H	1.268396000	3.671850000	1.826145000
H	1.012500000	2.260160000	2.855087000
H	2.835743000	3.669919000	3.790030000
H	3.484351000	2.110053000	3.264527000
H	3.735391000	3.549154000	2.270676000
H	0.534801000	6.027561000	-1.374852000
H	1.911825000	6.253758000	-2.462067000
H	0.680064000	5.043327000	-2.838125000
H	-1.243035000	-1.047855000	-1.807268000
H	1.907508000	-0.502084000	-0.084255000
O	2.542890000	1.511047000	-1.373716000

H	-2.208640000	-0.265456000	-0.558927000
H	1.208403000	-1.044371000	-1.597182000
H	-0.304844000	1.268260000	-1.694306000
C	4.026512000	-1.994154000	-0.808778000
C	5.690884000	-3.974041000	0.223200000
C	6.234866000	-2.925099000	-0.510612000
C	5.396338000	-1.942848000	-1.025071000
H	3.417964000	-1.213985000	-1.235725000
H	6.331461000	-4.748142000	0.624435000
H	7.301660000	-2.876486000	-0.684113000
H	5.807850000	-1.125620000	-1.602036000
C	3.456950000	-3.040998000	-0.061608000
C	4.323933000	-4.030817000	0.437085000
H	3.904458000	-4.851268000	1.005023000
H	1.792237000	-4.156520000	0.695004000
C	-2.753901000	-2.794583000	0.213929000
C	-5.171677000	-2.817356000	0.631831000
C	-4.101000000	-2.375165000	-0.162444000
C	-6.478366000	-2.455810000	0.345513000
C	-4.402125000	-1.589370000	-1.285289000
C	-5.712578000	-1.239245000	-1.579560000
C	-6.754758000	-1.660621000	-0.762016000
H	-7.283382000	-2.801137000	0.980474000
H	-3.614853000	-1.280616000	-1.955848000
H	-5.920932000	-0.640381000	-2.456252000

H	-7.774500000	-1.383635000	-0.993393000
H	-2.703715000	-3.705731000	0.800644000
H	-4.962695000	-3.445561000	1.488169000
O	-0.446164000	-3.968805000	1.142061000

B5

C	-0.081413000	-2.481999000	0.606930000
C	1.273004000	-2.120291000	0.070574000
H	4.800185000	3.808196000	-1.089123000
C	1.871533000	2.193134000	-1.654103000
C	3.002619000	2.958554000	-1.906506000
C	3.917831000	3.214524000	-0.890152000
C	3.694880000	2.699375000	0.381045000
C	2.562224000	1.933245000	0.633227000
C	1.641126000	1.672888000	-0.379092000
H	1.155401000	1.993538000	-2.438783000
H	3.169197000	3.357956000	-2.898279000
H	4.405136000	2.886372000	1.175456000
H	2.394128000	1.530596000	1.623906000
N	0.230571000	-0.199339000	-1.104555000
C	1.322184000	-1.172927000	-1.108195000
C	-1.275129000	-1.949394000	-0.128782000
C	-1.046426000	-0.899954000	-1.197868000
C	-2.466554000	-2.471163000	0.224335000
C	0.389542000	0.855106000	-0.094714000

P	-1.051441000	2.029853000	-0.043461000
O	-2.035883000	1.264610000	0.962919000
O	-0.560310000	3.252130000	0.879481000
C	-3.327658000	1.832270000	1.295806000
C	-3.926265000	1.020153000	2.421792000
C	-0.163440000	4.532515000	0.326853000
C	-0.669800000	5.631861000	1.235263000
H	-3.186868000	2.872491000	1.593978000
H	-3.955170000	1.808491000	0.404618000
H	0.924810000	4.540392000	0.267757000
H	-0.571146000	4.623243000	-0.678915000
H	-0.336849000	6.601075000	0.860048000
H	-1.759159000	5.637094000	1.274618000
H	-0.286811000	5.506905000	2.248107000
H	-3.285115000	1.048651000	3.302464000
H	-4.901522000	1.429188000	2.690580000
H	-4.060517000	-0.018066000	2.122234000
H	1.247987000	-1.727784000	-2.050336000
H	-1.062011000	-1.372870000	-2.187299000
O	-1.614078000	2.440210000	-1.350419000
H	2.271730000	-0.644159000	-1.130600000
H	-1.856166000	-0.175580000	-1.201135000
H	0.460214000	0.470970000	0.932901000
C	-4.880928000	-2.701205000	0.521175000
C	-5.464616000	-1.439537000	-1.871234000

C	-6.500110000	-1.884647000	-1.057206000
C	-6.201435000	-2.521315000	0.142865000
H	-4.653536000	-3.205832000	1.451369000
H	-5.686840000	-0.957983000	-2.814162000
H	-7.529225000	-1.746460000	-1.360443000
H	-6.997785000	-2.883391000	0.779407000
C	-3.817200000	-2.237933000	-0.273923000
C	-4.141585000	-1.611844000	-1.489519000
H	-3.365305000	-1.276528000	-2.157550000
H	-2.412451000	-3.180421000	1.043402000
C	2.334416000	-2.652922000	0.705556000
C	4.308766000	-2.305178000	-0.844923000
C	3.763399000	-2.543740000	0.426032000
C	5.682159000	-2.222766000	-1.026350000
C	4.654823000	-2.728275000	1.495938000
C	6.025369000	-2.633198000	1.316444000
C	6.545682000	-2.375625000	0.052242000
H	6.079998000	-2.046299000	-2.016826000
H	4.254363000	-2.940729000	2.478893000
H	6.689397000	-2.769029000	2.159679000
H	7.615493000	-2.309486000	-0.093582000
H	2.084915000	-3.242896000	1.581587000
H	3.661466000	-2.220549000	-1.704147000
O	-0.203769000	-3.205423000	1.584635000

B6

C	0.393231000	-3.016002000	-0.417807000
C	1.591281000	-2.249200000	0.045701000
H	4.343118000	3.791206000	-1.846843000
C	1.648805000	1.750810000	-1.553461000
C	2.709640000	2.426029000	-2.144329000
C	3.516313000	3.268023000	-1.385141000
C	3.256397000	3.430401000	-0.029234000
C	2.194431000	2.754546000	0.561512000
C	1.382046000	1.909875000	-0.192517000
H	1.021014000	1.093239000	-2.137675000
H	2.905844000	2.294935000	-3.200186000
H	3.882224000	4.077674000	0.570767000
H	1.995653000	2.884237000	1.617848000
N	0.213448000	-0.232700000	0.074784000
C	1.333868000	-0.926462000	0.713924000
C	-0.946480000	-2.385840000	-0.190298000
C	-1.024751000	-0.966792000	0.320477000
C	-2.014124000	-3.155529000	-0.481037000
C	0.206330000	1.186366000	0.446499000
P	-1.349010000	2.094632000	-0.016799000
O	-2.287701000	1.746781000	1.240616000
O	-1.016531000	3.641283000	0.271220000
C	-3.640548000	2.262127000	1.295016000
C	-4.247565000	1.857269000	2.619680000

C	-0.769308000	4.592087000	-0.797142000
C	-1.250144000	5.953914000	-0.346510000
H	-3.605277000	3.348469000	1.197995000
H	-4.201662000	1.856962000	0.452330000
H	0.302459000	4.599105000	-0.995544000
H	-1.289637000	4.255922000	-1.692644000
H	-0.744846000	6.262095000	0.568704000
H	-1.037414000	6.692575000	-1.120994000
H	-2.324919000	5.949910000	-0.164587000
H	-3.672879000	2.266102000	3.450350000
H	-5.268741000	2.235089000	2.688669000
H	-4.275911000	0.772639000	2.722452000
H	2.213094000	-0.295545000	0.676378000
H	-1.836444000	-0.460362000	-0.197039000
O	-1.909078000	1.829770000	-1.360457000
H	1.116221000	-1.094456000	1.786399000
H	-1.270520000	-0.968994000	1.398218000
H	0.268031000	1.320537000	1.538657000
C	-4.295728000	-3.764570000	-1.109685000
C	-5.428522000	-1.773680000	0.442457000
C	-6.246296000	-2.611233000	-0.307453000
C	-5.672192000	-3.611666000	-1.084497000
H	-3.853838000	-4.550172000	-1.708783000
H	-5.869518000	-1.005822000	1.064381000
H	-7.321022000	-2.491655000	-0.278960000

H	-6.298354000	-4.275981000	-1.664929000
C	-3.448222000	-2.912619000	-0.380102000
C	-4.048830000	-1.917187000	0.407906000
H	-3.443267000	-1.268361000	1.019733000
H	-1.754205000	-4.126760000	-0.887664000
C	2.789822000	-2.835782000	-0.102873000
C	4.580901000	-1.052512000	-0.029567000
C	4.132209000	-2.351029000	0.244151000
C	5.873620000	-0.664696000	0.295924000
C	5.035991000	-3.252310000	0.824030000
C	6.322070000	-2.859135000	1.164445000
C	6.745620000	-1.560557000	0.904139000
H	6.202006000	0.339840000	0.064617000
H	4.715683000	-4.268435000	1.015866000
H	6.997137000	-3.569180000	1.623459000
H	7.751104000	-1.253830000	1.159417000
H	2.751725000	-3.841115000	-0.510559000
H	3.931873000	-0.352608000	-0.535874000
O	0.504948000	-4.112298000	-0.945220000

B7

C	-0.862082000	-2.879148000	0.040598000
C	0.521613000	-2.335653000	-0.158447000
H	3.763050000	1.654561000	4.440264000
C	2.033794000	2.401131000	1.615414000

C	2.998440000	2.490152000	2.614718000
C	3.010834000	1.584521000	3.665835000
C	2.044779000	0.585289000	3.712368000
C	1.082052000	0.496924000	2.716493000
C	1.061858000	1.401739000	1.650100000
H	2.051282000	3.122359000	0.814461000
H	3.743864000	3.272919000	2.563827000
H	2.038299000	-0.127897000	4.526081000
H	0.339315000	-0.286923000	2.749450000
N	-0.358098000	-0.130670000	0.332105000
C	0.646674000	-0.859240000	-0.435913000
C	-2.002156000	-1.906874000	-0.020781000
C	-1.706539000	-0.432177000	-0.136298000
C	-3.240098000	-2.434880000	0.044276000
C	-0.072467000	1.268476000	0.626748000
P	-0.000650000	2.348544000	-0.894891000
O	0.060921000	3.875876000	-0.373664000
O	1.470220000	2.088825000	-1.477554000
C	-0.988766000	4.840242000	-0.641258000
C	-1.729587000	5.186808000	0.633539000
C	1.858695000	2.639917000	-2.761773000
C	3.194204000	2.046076000	-3.148995000
H	-0.487587000	5.716735000	-1.051754000
H	-1.657229000	4.432595000	-1.397839000
H	1.085170000	2.401960000	-3.492069000

H	1.923436000	3.725476000	-2.663844000
H	3.516037000	2.458250000	-4.106592000
H	3.953570000	2.276798000	-2.402357000
H	3.124362000	0.963399000	-3.246914000
H	-1.041952000	5.544065000	1.399728000
H	-2.459345000	5.973203000	0.432548000
H	-2.266466000	4.322589000	1.025670000
H	0.520766000	-0.684163000	-1.517917000
H	-1.850682000	-0.089366000	-1.169938000
O	-1.115187000	2.103084000	-1.843267000
H	1.631655000	-0.488777000	-0.178821000
H	-2.415102000	0.125916000	0.478281000
H	-0.977627000	1.660776000	1.105475000
C	-4.826309000	-0.540287000	-0.546277000
C	-6.920098000	-2.011512000	0.544521000
C	-7.165032000	-0.750882000	0.009777000
C	-6.115379000	-0.025649000	-0.542335000
H	-4.040341000	0.033374000	-1.011420000
H	-7.731343000	-2.590564000	0.965242000
H	-8.166967000	-0.342977000	0.011837000
H	-6.301061000	0.945877000	-0.980857000
C	-4.555440000	-1.804655000	0.003120000
C	-5.636673000	-2.532628000	0.529402000
H	-5.452259000	-3.518522000	0.936460000
H	-3.258083000	-3.510507000	0.181569000

C	1.530726000	-3.221128000	-0.142724000
C	3.701159000	-3.925871000	-1.076389000
C	2.976620000	-3.012182000	-0.298077000
C	5.070830000	-3.791354000	-1.250775000
C	3.683547000	-1.985671000	0.341491000
C	5.057023000	-1.862084000	0.180639000
C	5.754988000	-2.755566000	-0.624107000
H	5.606239000	-4.501548000	-1.866898000
H	3.165594000	-1.303861000	1.000585000
H	5.583914000	-1.068971000	0.694335000
H	6.824759000	-2.654737000	-0.749506000
H	1.222082000	-4.255929000	-0.032128000
H	3.175460000	-4.742696000	-1.554257000
O	-1.052158000	-4.070273000	0.235309000

B8

C	1.202235000	2.593381000	0.137562000
C	-0.212989000	2.198155000	-0.155826000
H	-2.822389000	-1.718873000	5.339667000
C	-1.147088000	-0.345626000	2.727380000
C	-1.884686000	-0.424073000	3.904182000
C	-2.251618000	-1.658398000	4.422594000
C	-1.867433000	-2.816736000	3.756758000
C	-1.134265000	-2.741645000	2.579452000
C	-0.771785000	-1.500957000	2.043625000

H	-0.836549000	0.618395000	2.355477000
H	-2.164071000	0.486407000	4.417790000
H	-2.132570000	-3.786585000	4.156820000
H	-0.843091000	-3.650597000	2.071745000
N	0.454046000	-0.074695000	0.397042000
C	-0.523862000	0.732432000	-0.322053000
C	2.248493000	1.525225000	0.042254000
C	1.794841000	0.098550000	-0.142010000
C	3.529069000	1.925108000	0.153890000
C	0.079662000	-1.428208000	0.770244000
P	-0.659953000	-2.506234000	-0.575246000
O	-2.129256000	-1.935645000	-0.920172000
O	0.191731000	-2.031146000	-1.852077000
C	-3.337741000	-2.591454000	-0.452439000
C	-4.385156000	-2.519723000	-1.541686000
C	0.078886000	-2.760841000	-3.101680000
C	1.221456000	-2.348594000	-4.002309000
H	-3.099228000	-3.621881000	-0.194309000
H	-3.665740000	-2.070969000	0.447413000
H	0.106020000	-3.828729000	-2.884919000
H	-0.885542000	-2.519557000	-3.551733000
H	2.181161000	-2.596180000	-3.549350000
H	1.146732000	-2.875865000	-4.954529000
H	1.196621000	-1.277476000	-4.201686000
H	-4.057539000	-3.053520000	-2.433768000

H	-5.311941000	-2.976389000	-1.190461000
H	-4.596333000	-1.485521000	-1.813382000
H	-0.549542000	0.461489000	-1.389840000
H	1.841551000	-0.191437000	-1.203607000
O	-0.635859000	-3.962536000	-0.297891000
H	-1.514699000	0.520976000	0.073658000
H	2.472128000	-0.564398000	0.396339000
H	1.009352000	-1.961663000	0.983644000
C	5.907115000	1.733600000	0.708764000
C	6.162021000	-0.713913000	-0.562083000
C	7.260952000	-0.151213000	0.076943000
C	7.128542000	1.079728000	0.711203000
H	5.811457000	2.695531000	1.195564000
H	6.262073000	-1.660523000	-1.076415000
H	8.214728000	-0.661407000	0.070450000
H	7.980147000	1.532598000	1.201142000
C	4.776115000	1.169311000	0.094875000
C	4.934738000	-0.065856000	-0.553980000
H	4.108005000	-0.508212000	-1.087649000
H	3.648580000	2.984225000	0.354901000
C	-1.111294000	3.198143000	-0.236944000
C	-3.209152000	2.159939000	-1.220570000
C	-2.540699000	3.184269000	-0.530426000
C	-4.573281000	2.239810000	-1.463609000
C	-3.296670000	4.295354000	-0.119239000

C	-4.660841000	4.366760000	-0.350333000
C	-5.307190000	3.335495000	-1.023727000
H	-5.064790000	1.444639000	-2.008476000
H	-2.795837000	5.105512000	0.394938000
H	-5.219309000	5.229906000	-0.013540000
H	-6.370509000	3.390931000	-1.214515000
H	-0.702959000	4.179589000	-0.021267000
H	-2.662489000	1.310798000	-1.599400000
O	1.496795000	3.748954000	0.404371000

68

symmetry c1

C	1.202235000	2.593381000	0.137562000
C	-0.212989000	2.198155000	-0.155826000
H	-2.822389000	-1.718873000	5.339667000
C	-1.147088000	-0.345626000	2.727380000
C	-1.884686000	-0.424073000	3.904182000
C	-2.251618000	-1.658398000	4.422594000
C	-1.867433000	-2.816736000	3.756758000
C	-1.134265000	-2.741645000	2.579452000
C	-0.771785000	-1.500957000	2.043625000
H	-0.836549000	0.618395000	2.355477000
H	-2.164071000	0.486407000	4.417790000
H	-2.132570000	-3.786585000	4.156820000
H	-0.843091000	-3.650597000	2.071745000

N	0.454046000	-0.074695000	0.397042000
C	-0.523862000	0.732432000	-0.322053000
C	2.248493000	1.525225000	0.042254000
C	1.794841000	0.098550000	-0.142010000
C	3.529069000	1.925108000	0.153890000
C	0.079662000	-1.428208000	0.770244000
P	-0.659953000	-2.506234000	-0.575246000
O	-2.129256000	-1.935645000	-0.920172000
O	0.191731000	-2.031146000	-1.852077000
C	-3.337741000	-2.591454000	-0.452439000
C	-4.385156000	-2.519723000	-1.541686000
C	0.078886000	-2.760841000	-3.101680000
C	1.221456000	-2.348594000	-4.002309000
H	-3.099228000	-3.621881000	-0.194309000
H	-3.665740000	-2.070969000	0.447413000
H	0.106020000	-3.828729000	-2.884919000
H	-0.885542000	-2.519557000	-3.551733000
H	2.181161000	-2.596180000	-3.549350000
H	1.146732000	-2.875865000	-4.954529000
H	1.196621000	-1.277476000	-4.201686000
H	-4.057539000	-3.053520000	-2.433768000
H	-5.311941000	-2.976389000	-1.190461000
H	-4.596333000	-1.485521000	-1.813382000
H	-0.549542000	0.461489000	-1.389840000
H	1.841551000	-0.191437000	-1.203607000

O	-0.635859000	-3.962536000	-0.297891000
H	-1.514699000	0.520976000	0.073658000
H	2.472128000	-0.564398000	0.396339000
H	1.009352000	-1.961663000	0.983644000
C	5.907115000	1.733600000	0.708764000
C	6.162021000	-0.713913000	-0.562083000
C	7.260952000	-0.151213000	0.076943000
C	7.128542000	1.079728000	0.711203000
H	5.811457000	2.695531000	1.195564000
H	6.262073000	-1.660523000	-1.076415000
H	8.214728000	-0.661407000	0.070450000
H	7.980147000	1.532598000	1.201142000
C	4.776115000	1.169311000	0.094875000
C	4.934738000	-0.065856000	-0.553980000
H	4.108005000	-0.508212000	-1.087649000
H	3.648580000	2.984225000	0.354901000
C	-1.111294000	3.198143000	-0.236944000
C	-3.209152000	2.159939000	-1.220570000
C	-2.540699000	3.184269000	-0.530426000
C	-4.573281000	2.239810000	-1.463609000
C	-3.296670000	4.295354000	-0.119239000
C	-4.660841000	4.366760000	-0.350333000
C	-5.307190000	3.335495000	-1.023727000
H	-5.064790000	1.444639000	-2.008476000
H	-2.795837000	5.105512000	0.394938000

H	-5.219309000	5.229906000	-0.013540000
H	-6.370509000	3.390931000	-1.214515000
H	-0.702959000	4.179589000	-0.021267000
H	-2.662489000	1.310798000	-1.599400000
O	1.496795000	3.748954000	0.4043710009

B9

C	-0.385904000	-2.570049000	-0.206726000
C	0.916047000	-1.961828000	-0.635560000
H	1.210430000	-1.140422000	4.972536000
C	-0.802260000	-0.002948000	2.496367000
C	-0.429324000	-0.572611000	3.706750000
C	0.916031000	-0.696181000	4.031046000
C	1.880400000	-0.239381000	3.140884000
C	1.506011000	0.331375000	1.930866000
C	0.156965000	0.447428000	1.587481000
H	-1.853214000	0.090100000	2.253462000
H	-1.189040000	-0.918236000	4.395173000
H	2.930252000	-0.323160000	3.388557000
H	2.266695000	0.704368000	1.258900000
N	-0.283462000	0.213255000	-0.946622000
C	0.904629000	-0.574552000	-1.247751000
C	-1.646680000	-1.820580000	-0.529583000
C	-1.504846000	-0.508683000	-1.268653000
C	-2.802483000	-2.364422000	-0.106829000

C	-0.298825000	1.049974000	0.264952000
P	0.573601000	2.612705000	-0.188028000
O	-0.351515000	3.298978000	-1.314845000
O	0.262983000	3.519252000	1.092973000
C	-0.015228000	3.234887000	-2.720199000
C	-0.609124000	4.443094000	-3.411216000
C	0.887572000	4.813896000	1.260721000
C	0.675407000	5.259585000	2.690222000
H	1.069047000	3.206951000	-2.821599000
H	-0.425144000	2.307056000	-3.121504000
H	1.947759000	4.725078000	1.021829000
H	0.428448000	5.510996000	0.557095000
H	1.124231000	6.242562000	2.841563000
H	-0.387520000	5.327299000	2.921134000
H	1.135881000	4.558600000	3.385530000
H	-0.184270000	5.365566000	-3.015440000
H	-0.396447000	4.398839000	-4.480669000
H	-1.690415000	4.476501000	-3.277494000
H	0.962212000	-0.660569000	-2.340865000
H	-1.497014000	-0.689865000	-2.351466000
O	1.998657000	2.484925000	-0.584068000
H	1.782733000	-0.009787000	-0.945157000
H	-2.359872000	0.136482000	-1.071723000
H	-1.333773000	1.369009000	0.392672000
C	-4.631660000	-1.156698000	-1.376144000

C	-6.442190000	-1.858546000	0.611465000
C	-6.866736000	-1.098425000	-0.473686000
C	-5.958677000	-0.760105000	-1.470209000
H	-3.954545000	-0.916939000	-2.181932000
H	-7.144587000	-2.140606000	1.384432000
H	-7.899453000	-0.785677000	-0.549280000
H	-6.286745000	-0.191976000	-2.330587000
C	-4.179076000	-1.905532000	-0.278798000
C	-5.120610000	-2.266143000	0.699120000
H	-4.797338000	-2.867831000	1.538856000
H	-2.688528000	-3.268487000	0.482176000
C	2.013263000	-2.719362000	-0.436333000
C	4.354741000	-3.314434000	-0.073930000
C	3.423264000	-2.485437000	-0.724501000
C	5.717838000	-3.165820000	-0.269713000
C	3.924607000	-1.518106000	-1.611661000
C	5.289998000	-1.375754000	-1.813367000
C	6.193021000	-2.190690000	-1.140530000
H	6.410257000	-3.813228000	0.251742000
H	3.253293000	-0.885338000	-2.168542000
H	5.649560000	-0.623990000	-2.503190000
H	7.256456000	-2.073021000	-1.300074000
H	1.811176000	-3.664168000	0.056480000
H	3.989512000	-4.079379000	0.599084000
O	-0.429728000	-3.649880000	0.363211000

B10

C	-0.027236000	-2.857812000	0.444705000
C	-1.315060000	-2.236123000	0.005363000
H	-4.744311000	3.774715000	0.479951000
C	-1.943215000	1.916478000	0.930027000
C	-3.132159000	2.567393000	1.229893000
C	-3.818170000	3.268053000	0.243657000
C	-3.307873000	3.308714000	-1.047642000
C	-2.120159000	2.652305000	-1.347325000
C	-1.423337000	1.950921000	-0.365069000
H	-1.416688000	1.371207000	1.699433000
H	-3.524514000	2.527072000	2.237366000
H	-3.834427000	3.847704000	-1.824107000
H	-1.728199000	2.688026000	-2.355834000
N	-0.077121000	-0.146031000	-0.256101000
C	-1.252065000	-0.906247000	-0.696801000
C	1.225186000	-2.221218000	-0.062183000
C	1.128039000	-0.859212000	-0.695073000
C	2.357473000	-2.940967000	0.075319000
C	-0.119937000	1.255024000	-0.715269000
P	1.321345000	2.239245000	-0.051862000
O	0.874691000	3.709602000	-0.484688000
O	1.177007000	2.283772000	1.547433000
C	1.792640000	4.822475000	-0.343130000

C	1.247160000	5.987957000	-1.136850000
C	1.907140000	1.367118000	2.404365000
C	1.999260000	1.978772000	3.784308000
H	1.869214000	5.067152000	0.717478000
H	2.774867000	4.515285000	-0.703035000
H	1.365620000	0.420664000	2.425629000
H	2.893295000	1.196876000	1.974247000
H	2.548235000	2.919979000	3.757417000
H	1.007615000	2.168431000	4.194627000
H	2.522594000	1.294597000	4.453932000
H	0.257681000	6.271656000	-0.779601000
H	1.911499000	6.847134000	-1.032694000
H	1.174788000	5.735829000	-2.194544000
H	-1.224974000	-1.041889000	-1.795045000
H	2.002374000	-0.273436000	-0.434605000
O	2.665366000	1.821083000	-0.522446000
H	-2.150651000	-0.340639000	-0.472590000
H	1.133218000	-0.947474000	-1.798885000
H	0.011545000	1.314490000	-1.808886000
C	4.183140000	-1.652850000	-1.153620000
C	6.053322000	-3.429650000	-0.108942000
C	6.471132000	-2.389089000	-0.931733000
C	5.530104000	-1.507958000	-1.452407000
H	3.487030000	-0.952629000	-1.583961000
H	6.774772000	-4.126061000	0.297253000

H	7.519660000	-2.268867000	-1.169448000
H	5.844527000	-0.698426000	-2.097256000
C	3.740082000	-2.690180000	-0.314737000
C	4.708731000	-3.578214000	0.187825000
H	4.387471000	-4.391925000	0.825201000
H	2.209471000	-3.885421000	0.587651000
C	-2.441167000	-2.917611000	0.287208000
C	-4.817357000	-3.228149000	0.794767000
C	-3.839255000	-2.630878000	-0.018136000
C	-6.167786000	-3.003667000	0.581775000
C	-4.277324000	-1.829169000	-1.083777000
C	-5.630733000	-1.614853000	-1.303997000
C	-6.581061000	-2.191573000	-0.469317000
H	-6.899286000	-3.468197000	1.229503000
H	-3.562982000	-1.398037000	-1.767822000
H	-5.944375000	-0.999408000	-2.136635000
H	-7.634761000	-2.020312000	-0.644079000
H	-2.278101000	-3.821693000	0.864163000
H	-4.500292000	-3.868932000	1.607446000
O	-0.005734000	-3.859789000	1.144595000

B11

C	-0.234476000	-2.547547000	-0.682575000
C	-1.452635000	-1.944810000	-0.043263000
H	-4.498256000	3.469778000	-1.302366000

C	-1.773351000	1.510061000	-1.775035000
C	-2.945146000	2.198420000	-2.067301000
C	-3.586073000	2.933189000	-1.078278000
C	-3.047057000	2.971848000	0.203675000
C	-1.873816000	2.287269000	0.492265000
C	-1.219903000	1.547242000	-0.495892000
H	-1.282986000	0.936312000	-2.550447000
H	-3.357294000	2.155108000	-3.066484000
H	-3.538567000	3.541682000	0.981192000
H	-1.457527000	2.332283000	1.487477000
N	0.011577000	-0.139216000	0.911586000
C	-1.237497000	-0.899686000	1.034622000
C	1.103011000	-2.139999000	-0.141228000
C	1.134525000	-1.089406000	0.955458000
C	2.160029000	-2.833546000	-0.607408000
C	0.076175000	0.805622000	-0.225321000
P	1.460468000	2.016572000	0.073499000
O	1.228989000	3.026367000	-1.140612000
O	1.067557000	2.904183000	1.355495000
C	2.206094000	4.052365000	-1.446347000
C	1.879170000	4.622875000	-2.807717000
C	1.584072000	2.604427000	2.676795000
C	1.581295000	3.879246000	3.491170000
H	2.149494000	4.818979000	-0.671779000
H	3.200694000	3.606466000	-1.425258000

H	0.941673000	1.844468000	3.123599000
H	2.588511000	2.195207000	2.576290000
H	1.942287000	3.671663000	4.499773000
H	2.231919000	4.629351000	3.041852000
H	0.576231000	4.294250000	3.565616000
H	0.875265000	5.046254000	-2.819610000
H	2.590278000	5.411946000	-3.056804000
H	1.939044000	3.851332000	-3.574803000
H	-2.073494000	-0.217845000	1.097681000
H	1.072910000	-1.591970000	1.926565000
O	2.823263000	1.432622000	0.168203000
H	-1.184200000	-1.404913000	2.005123000
H	2.065613000	-0.540914000	0.948313000
H	0.365772000	0.302089000	-1.158370000
C	4.293014000	-1.594427000	0.010582000
C	5.669224000	-4.014779000	-0.084342000
C	6.347802000	-2.844646000	0.237869000
C	5.656041000	-1.638652000	0.270531000
H	3.796857000	-0.635182000	0.005672000
H	6.198980000	-4.957107000	-0.129426000
H	7.409151000	-2.870259000	0.446565000
H	6.180627000	-0.718825000	0.492744000
C	3.590481000	-2.769139000	-0.297116000
C	4.312096000	-3.972051000	-0.363820000
H	3.790235000	-4.882390000	-0.630537000

H	1.892829000	-3.623328000	-1.302575000
C	-2.635176000	-2.472584000	-0.408524000
C	-4.897759000	-3.254283000	0.136006000
C	-4.003489000	-2.177967000	0.030513000
C	-6.209701000	-3.063372000	0.542786000
C	-4.491852000	-0.891980000	0.298999000
C	-5.810195000	-0.699590000	0.687799000
C	-6.671572000	-1.782550000	0.824333000
H	-6.874679000	-3.912403000	0.629344000
H	-3.856814000	-0.030204000	0.161883000
H	-6.167546000	0.304003000	0.876577000
H	-7.696958000	-1.628042000	1.132492000
H	-2.555855000	-3.296572000	-1.110791000
H	-4.547614000	-4.251643000	-0.097533000
O	-0.335387000	-3.339790000	-1.608207000

B12

C	-0.245882000	-2.552656000	-0.648468000
C	-1.461280000	-1.935804000	-0.021287000
H	-4.451592000	3.375615000	-1.557941000
C	-1.695084000	1.428009000	-1.870562000
C	-2.861816000	2.094920000	-2.225853000
C	-3.543487000	2.855172000	-1.284581000
C	-3.050366000	2.939939000	0.013417000
C	-1.882371000	2.276176000	0.365003000

C	-1.186282000	1.511651000	-0.574919000
H	-1.173078000	0.834687000	-2.609711000
H	-3.237839000	2.015411000	-3.237009000
H	-3.573173000	3.530415000	0.754284000
H	-1.500836000	2.360540000	1.371404000
N	0.020003000	-0.136085000	0.908587000
C	-1.236021000	-0.880337000	1.043905000
C	1.086727000	-2.178335000	-0.074107000
C	1.139037000	-1.088703000	0.979462000
C	2.147457000	-2.855865000	-0.559094000
C	0.108637000	0.795639000	-0.239707000
P	1.466968000	2.025141000	0.105024000
O	1.287684000	3.008736000	-1.140922000
O	0.994326000	2.935442000	1.346681000
C	2.277728000	4.028012000	-1.423452000
C	2.012826000	4.570290000	-2.809811000
C	1.465916000	2.677649000	2.692272000
C	1.425947000	3.974405000	3.470596000
H	2.187177000	4.811115000	-0.668634000
H	3.270204000	3.583172000	-1.348229000
H	0.813625000	1.925122000	3.137824000
H	2.475751000	2.273050000	2.638046000
H	1.751412000	3.798037000	4.497159000
H	2.087825000	4.716218000	3.023910000
H	0.416454000	4.384644000	3.497093000

H	1.010790000	4.993271000	-2.875683000
H	2.735150000	5.353919000	-3.043456000
H	2.106093000	3.782699000	-3.557055000
H	-1.187004000	-1.373565000	2.021053000
H	1.107886000	-1.529468000	1.981962000
O	2.823657000	1.453231000	0.286796000
H	-2.065544000	-0.189714000	1.099137000
H	2.073778000	-0.541703000	0.899136000
H	0.443207000	0.284323000	-1.152381000
C	4.101051000	-2.298722000	0.966148000
C	5.831346000	-3.335695000	-0.947856000
C	6.341123000	-2.812984000	0.235936000
C	5.469480000	-2.303592000	1.191768000
H	3.449039000	-1.918781000	1.735804000
H	6.499547000	-3.744173000	-1.694226000
H	7.407789000	-2.810846000	0.416127000
H	5.857473000	-1.908299000	2.121016000
C	3.566548000	-2.807042000	-0.228367000
C	4.464044000	-3.341783000	-1.168958000
H	4.070258000	-3.757738000	-2.087404000
H	1.898232000	-3.555534000	-1.350022000
C	-2.646199000	-2.460682000	-0.382282000
C	-4.911641000	-3.222193000	0.182945000
C	-4.012134000	-2.152794000	0.055249000
C	-6.221450000	-3.016510000	0.589500000

C	-4.492370000	-0.859448000	0.301246000
C	-5.808659000	-0.652626000	0.689770000
C	-6.675587000	-1.728106000	0.848344000
H	-6.890841000	-3.860098000	0.693554000
H	-3.852414000	-0.004258000	0.146610000
H	-6.160149000	0.356242000	0.860719000
H	-7.699365000	-1.562268000	1.155981000
H	-2.572165000	-3.291013000	-1.077585000
H	-4.567521000	-4.225654000	-0.032817000
O	-0.345435000	-3.339633000	-1.579013000

B13

C	-0.123527000	-2.524475000	-0.641216000
C	-1.351559000	-1.976889000	0.022011000
H	-4.545654000	3.011490000	-1.796301000
C	-1.632229000	1.285018000	-1.923921000
C	-2.826699000	1.850171000	-2.353684000
C	-3.613543000	2.572636000	-1.466394000
C	-3.195009000	2.725349000	-0.149314000
C	-1.998339000	2.164810000	0.276697000
C	-1.199137000	1.434894000	-0.606698000
H	-1.028164000	0.719954000	-2.621681000
H	-3.142183000	1.720376000	-3.380345000
H	-3.800950000	3.286545000	0.549758000
H	-1.674937000	2.305319000	1.296935000

N	0.062069000	-0.130024000	0.939562000
C	-1.166441000	-0.914650000	1.083419000
C	1.200871000	-2.105884000	-0.078007000
C	1.215562000	-1.039237000	0.999414000
C	2.284491000	-2.718582000	-0.596648000
C	0.126034000	0.822502000	-0.193891000
P	1.380604000	2.136673000	0.218846000
O	1.249676000	3.056109000	-1.083549000
O	0.768938000	3.053586000	1.393648000
C	2.166647000	4.152937000	-1.308170000
C	1.982540000	4.638404000	-2.728525000
C	1.351193000	3.090013000	2.720577000
C	0.435647000	2.419848000	3.723747000
H	1.940064000	4.942560000	-0.589075000
H	3.184252000	3.802242000	-1.133186000
H	2.328368000	2.611352000	2.687077000
H	1.487164000	4.145971000	2.954567000
H	0.883766000	2.464045000	4.718160000
H	-0.533152000	2.918591000	3.765616000
H	0.278145000	1.375107000	3.456884000
H	0.956970000	4.966338000	-2.895789000
H	2.650622000	5.479267000	-2.920999000
H	2.212965000	3.846006000	-3.440128000
H	-1.111075000	-1.375305000	2.076100000
H	1.199382000	-1.501054000	1.992874000

O	2.748191000	1.646886000	0.524237000
H	-2.025716000	-0.251419000	1.098312000
H	2.127851000	-0.453308000	0.936075000
H	0.549511000	0.347304000	-1.088178000
C	4.225849000	-2.116949000	0.925680000
C	5.984054000	-2.986718000	-1.045200000
C	6.480469000	-2.482004000	0.152067000
C	5.595392000	-2.056457000	1.136101000
H	3.564121000	-1.801851000	1.716185000
H	6.663467000	-3.330789000	-1.813599000
H	7.547745000	-2.429656000	0.320702000
H	5.973529000	-1.676048000	2.075550000
C	3.703974000	-2.607623000	-0.281733000
C	4.616218000	-3.058008000	-1.251673000
H	4.233372000	-3.460202000	-2.180837000
H	2.058080000	-3.407191000	-1.404024000
C	-2.532310000	-2.466757000	-0.402328000
C	-4.241463000	-1.735355000	1.322907000
C	-3.895192000	-2.192963000	0.041779000
C	-5.567716000	-1.512752000	1.665493000
C	-4.938237000	-2.442807000	-0.865698000
C	-6.260973000	-2.207313000	-0.527640000
C	-6.581953000	-1.737552000	0.741716000
H	-5.810917000	-1.169469000	2.662341000
H	-4.693683000	-2.818711000	-1.850780000

H	-7.043308000	-2.398205000	-1.250097000
H	-7.614184000	-1.561080000	1.012891000
H	-2.456218000	-3.165458000	-1.228933000
H	-3.477546000	-1.586127000	2.069946000
O	-0.203419000	-3.298850000	-1.584444000

B14

C	-0.121720000	-2.577722000	-0.567446000
C	-1.343311000	-2.015526000	0.095052000
H	-4.638305000	2.898174000	-1.758763000
C	-1.720434000	1.183344000	-1.934889000
C	-2.931591000	1.730745000	-2.340429000
C	-3.693245000	2.473374000	-1.447888000
C	-3.232932000	2.664070000	-0.149790000
C	-2.019720000	2.121408000	0.251529000
C	-1.245503000	1.371358000	-0.637271000
H	-1.136919000	0.600853000	-2.635816000
H	-3.279977000	1.570812000	-3.352083000
H	-3.818954000	3.241190000	0.553265000
H	-1.664710000	2.290890000	1.257157000
N	0.069884000	-0.129295000	0.923220000
C	-1.145741000	-0.922771000	1.122311000
C	1.207122000	-2.142420000	-0.028366000
C	1.235332000	-1.022470000	0.993597000
C	2.282362000	-2.797734000	-0.511757000

C	0.095431000	0.778055000	-0.250193000
P	1.347429000	2.112960000	0.096120000
O	1.137598000	3.050711000	-1.179918000
O	0.778610000	3.018710000	1.301255000
C	2.054282000	4.136987000	-1.458187000
C	1.827506000	4.592346000	-2.882206000
C	1.234239000	2.829133000	2.663111000
C	1.146465000	4.153819000	3.388920000
H	1.854062000	4.942064000	-0.749012000
H	3.074569000	3.783334000	-1.307439000
H	0.594716000	2.076932000	3.127192000
H	2.255170000	2.450372000	2.641086000
H	1.455724000	4.026403000	4.427717000
H	1.798512000	4.894258000	2.925777000
H	0.126704000	4.538800000	3.379890000
H	0.798440000	4.921045000	-3.024736000
H	2.492092000	5.426286000	-3.113220000
H	2.033101000	3.784128000	-3.583539000
H	-2.012007000	-0.268730000	1.140302000
H	1.253493000	-1.434702000	2.008673000
O	2.734730000	1.641541000	0.331388000
H	-1.059356000	-1.353889000	2.125886000
H	2.138047000	-0.430257000	0.874192000
H	0.492551000	0.272174000	-1.139794000
C	4.244070000	-2.110548000	0.950719000

C	5.971584000	-3.199200000	-0.936819000
C	6.485709000	-2.602791000	0.209622000
C	5.615559000	-2.067223000	1.152452000
H	3.595231000	-1.707093000	1.710743000
H	6.638678000	-3.628334000	-1.672516000
H	7.554608000	-2.563540000	0.371365000
H	6.006975000	-1.613634000	2.053188000
C	3.704308000	-2.693903000	-0.206827000
C	4.601641000	-3.251737000	-1.134309000
H	4.205173000	-3.724301000	-2.023747000
H	2.044129000	-3.527869000	-1.278123000
C	-2.527316000	-2.521968000	-0.299275000
C	-4.939748000	-2.511729000	-0.730340000
C	-3.884771000	-2.241912000	0.157264000
C	-6.258041000	-2.272011000	-0.378208000
C	-4.213593000	-1.759901000	1.433881000
C	-5.535334000	-1.533126000	1.790933000
C	-6.562004000	-1.778074000	0.886171000
H	-7.050162000	-2.478553000	-1.085544000
H	-3.438874000	-1.595530000	2.166599000
H	-5.765291000	-1.171061000	2.784271000
H	-7.590707000	-1.598491000	1.168480000
H	-2.459538000	-3.242902000	-1.107357000
H	-4.708238000	-2.906578000	-1.711155000
O	-0.210089000	-3.377567000	-1.488409000

B15

C	-0.125946000	-2.456980000	-0.794705000
C	-1.366445000	-1.932693000	-0.129694000
H	-4.640135000	3.473443000	-0.932999000
C	-1.886906000	1.618009000	-1.614915000
C	-3.083615000	2.292820000	-1.826105000
C	-3.708750000	2.947089000	-0.772405000
C	-3.129350000	2.918095000	0.492021000
C	-1.931078000	2.247413000	0.699877000
C	-1.292793000	1.589365000	-0.353914000
H	-1.408031000	1.108039000	-2.440475000
H	-3.527323000	2.301593000	-2.812586000
H	-3.609050000	3.424314000	1.319366000
H	-1.482669000	2.238170000	1.681822000
N	0.027377000	-0.127972000	0.920611000
C	-1.184961000	-0.951054000	1.011648000
C	1.194356000	-2.038480000	-0.220658000
C	1.191946000	-1.026744000	0.913276000
C	2.272846000	-2.694338000	-0.691634000
C	0.030187000	0.867731000	-0.170270000
P	1.400158000	2.104728000	0.092297000
O	1.113908000	3.078032000	-1.142731000
O	1.020701000	2.989717000	1.379319000
C	2.039746000	4.139983000	-1.477883000

C	1.668675000	4.675772000	-2.842270000
C	1.812258000	2.976744000	2.594745000
C	1.060047000	2.289461000	3.714816000
H	1.961535000	4.916207000	-0.714291000
H	3.053494000	3.738731000	-1.466730000
H	2.762658000	2.487595000	2.388243000
H	2.002946000	4.023333000	2.831838000
H	1.664851000	2.299961000	4.623411000
H	0.120224000	2.800052000	3.926479000
H	0.840689000	1.254622000	3.453568000
H	0.647137000	5.054816000	-2.844860000
H	2.340832000	5.491165000	-3.113737000
H	1.751062000	3.895933000	-3.598803000
H	-2.049588000	-0.313576000	1.129424000
H	1.168708000	-1.567221000	1.865547000
O	2.780102000	1.556265000	0.156367000
H	-1.093159000	-1.514419000	1.946524000
H	2.096490000	-0.435566000	0.913883000
H	0.290445000	0.414263000	-1.137414000
C	4.443026000	-3.794095000	-0.415889000
C	5.721568000	-1.442979000	0.285613000
C	6.439353000	-2.633609000	0.251956000
C	5.793738000	-3.812550000	-0.104190000
H	3.947151000	-4.711069000	-0.708278000
H	6.221359000	-0.516014000	0.533911000

H	7.495619000	-2.640282000	0.486303000
H	6.344361000	-4.742798000	-0.150189000
C	3.694831000	-2.607795000	-0.348351000
C	4.364468000	-1.423497000	-0.006561000
H	3.846625000	-0.475740000	-0.010129000
H	2.036227000	-3.466445000	-1.417012000
C	-2.530015000	-2.471573000	-0.537792000
C	-4.773195000	-3.360318000	-0.095067000
C	-3.910696000	-2.253071000	-0.094085000
C	-6.093905000	-3.245420000	0.311594000
C	-4.440929000	-1.011055000	0.281546000
C	-5.767723000	-0.893320000	0.670872000
C	-6.596941000	-2.009151000	0.700950000
H	-6.733397000	-4.118109000	0.314412000
H	-3.832229000	-0.121634000	0.228738000
H	-6.156970000	0.078434000	0.944108000
H	-7.629123000	-1.913118000	1.010186000
H	-2.418942000	-3.245494000	-1.290920000
H	-4.390624000	-4.322157000	-0.411860000
O	-0.194459000	-3.198352000	-1.764375000

B16

C	-0.215913000	-2.470507000	-0.746072000
C	-1.442126000	-1.887723000	-0.107754000
H	-4.408385000	3.391480000	-1.644076000

C	-1.612801000	1.489569000	-1.881234000
C	-2.776896000	2.142306000	-2.269823000
C	-3.502338000	2.881759000	-1.344847000
C	-3.055536000	2.959668000	-0.029865000
C	-1.890428000	2.309498000	0.355489000
C	-1.150268000	1.566335000	-0.567729000
H	-1.056558000	0.912635000	-2.608273000
H	-3.116341000	2.068314000	-3.294248000
H	-3.612540000	3.534231000	0.698514000
H	-1.544814000	2.388849000	1.374747000
N	0.021941000	-0.118593000	0.907615000
C	-1.238346000	-0.861530000	0.990576000
C	1.107570000	-2.115344000	-0.139088000
C	1.137864000	-1.075126000	0.963699000
C	2.179201000	-2.761974000	-0.641716000
C	0.140999000	0.860744000	-0.196382000
P	1.474684000	2.090351000	0.232546000
O	1.374917000	3.048828000	-1.044234000
O	0.929384000	3.006631000	1.438943000
C	2.359830000	4.086179000	-1.266071000
C	2.168488000	4.626688000	-2.665375000
C	1.528408000	2.979368000	2.758847000
C	0.573211000	2.370504000	3.764048000
H	2.210925000	4.867103000	-0.517773000
H	3.354591000	3.659967000	-1.133059000

H	2.464647000	2.426227000	2.707399000
H	1.750526000	4.017816000	3.005131000
H	1.034857000	2.364510000	4.753188000
H	-0.351447000	2.945187000	3.825184000
H	0.328317000	1.345567000	3.486581000
H	1.164703000	5.031751000	-2.790515000
H	2.889313000	5.423424000	-2.855152000
H	2.320036000	3.842090000	-3.406199000
H	-2.065652000	-0.168272000	1.043429000
H	1.082107000	-1.560605000	1.944246000
O	2.817881000	1.519930000	0.502369000
H	-1.216921000	-1.380949000	1.955183000
H	2.074242000	-0.526120000	0.932222000
H	0.523306000	0.389189000	-1.111582000
C	4.104543000	-2.271040000	0.940166000
C	5.873818000	-3.181121000	-1.002562000
C	6.360580000	-2.719260000	0.215802000
C	5.469595000	-2.273495000	1.185451000
H	3.437014000	-1.942033000	1.720008000
H	6.557302000	-3.540522000	-1.760257000
H	7.424487000	-2.715570000	0.411603000
H	5.839596000	-1.926425000	2.140919000
C	3.593227000	-2.717572000	-0.288714000
C	4.509938000	-3.189930000	-1.243948000
H	4.134067000	-3.559001000	-2.189563000

H	1.946111000	-3.425072000	-1.468307000
C	-2.618384000	-2.417651000	-0.489493000
C	-4.875704000	-3.227036000	0.039757000
C	-3.989418000	-2.142497000	-0.046282000
C	-6.188507000	-3.053059000	0.451428000
C	-4.486597000	-0.865376000	0.246753000
C	-5.805762000	-0.689589000	0.640544000
C	-6.659153000	-1.781114000	0.757709000
H	-6.847335000	-3.908272000	0.522947000
H	-3.857443000	0.003054000	0.125216000
H	-6.170181000	0.307644000	0.848663000
H	-7.685151000	-1.639871000	1.070173000
H	-2.531040000	-3.225181000	-1.209596000
H	-4.518602000	-4.217372000	-0.212402000
O	-0.299952000	-3.217758000	-1.710296000

B17

C	-0.555666000	2.833527000	-0.366458000
C	0.793028000	2.223322000	-0.128587000
H	-4.020793000	-4.296828000	-1.756831000
C	-2.022979000	-3.144239000	0.727052000
C	-2.986319000	-3.912618000	0.086562000
C	-3.269749000	-3.699229000	-1.257545000
C	-2.583458000	-2.711585000	-1.954950000
C	-1.618935000	-1.943336000	-1.313384000

C	-1.330338000	-2.152254000	0.035351000
H	-1.798963000	-3.324615000	1.770104000
H	-3.514909000	-4.678644000	0.638052000
H	-2.800139000	-2.535865000	-3.000485000
H	-1.096733000	-1.161078000	-1.845427000
N	-0.285592000	0.063298000	0.276113000
C	0.865728000	0.903278000	0.601395000
C	-1.748927000	2.031225000	0.051351000
C	-1.503083000	0.724078000	0.757909000
C	-2.955083000	2.581726000	-0.168736000
C	-0.257025000	-1.328021000	0.737864000
P	1.334572000	-2.272866000	0.572488000
O	1.979496000	-1.845596000	-0.840117000
O	2.258007000	-1.556655000	1.675619000
C	2.371630000	-2.828037000	-1.837788000
C	1.999323000	-2.317948000	-3.211934000
C	3.516764000	-2.153971000	2.075355000
C	3.996839000	-1.454390000	3.326971000
H	3.450551000	-2.964614000	-1.748905000
H	1.886005000	-3.774170000	-1.606798000
H	3.358811000	-3.219015000	2.243975000
H	4.231582000	-2.030281000	1.258992000
H	4.948673000	-1.882070000	3.645554000
H	4.141768000	-0.389637000	3.147037000
H	3.277870000	-1.575862000	4.136749000

H	2.469484000	-1.356216000	-3.416500000
H	2.335430000	-3.030250000	-3.967454000
H	0.920220000	-2.203894000	-3.306050000
H	0.912782000	1.100758000	1.688328000
H	-1.419057000	0.915737000	1.845157000
O	1.184293000	-3.733336000	0.752213000
H	1.777895000	0.386259000	0.349389000
H	-2.342916000	0.053634000	0.631625000
H	-0.436698000	-1.393663000	1.824445000
C	-4.708001000	0.753147000	-0.124015000
C	-6.559224000	2.574706000	0.864993000
C	-6.938178000	1.255223000	0.644580000
C	-6.010599000	0.351680000	0.138212000
H	-4.015849000	0.042472000	-0.551909000
H	-7.276638000	3.290207000	1.244296000
H	-7.951113000	0.937073000	0.851754000
H	-6.302475000	-0.670711000	-0.061463000
C	-4.303999000	2.074243000	0.111155000
C	-5.262693000	2.980960000	0.585983000
H	-4.976712000	4.012842000	0.745123000
H	-2.919985000	3.583868000	-0.584281000
C	1.851443000	2.948674000	-0.529267000
C	4.153611000	3.744056000	-0.199814000
C	3.291683000	2.671876000	-0.473726000
C	5.528399000	3.567792000	-0.149813000

C	3.864333000	1.421393000	-0.744129000
C	5.241808000	1.251066000	-0.713520000
C	6.079597000	2.317421000	-0.405674000
H	6.170228000	4.408949000	0.076008000
H	3.236863000	0.585391000	-1.015912000
H	5.664169000	0.281278000	-0.942071000
H	7.152131000	2.178677000	-0.380922000
H	1.594274000	3.923692000	-0.930999000
H	3.730936000	4.723859000	-0.017776000
O	-0.677510000	3.926840000	-0.897122000

Table S14. Structural information in the form of XYZ for compound 2.

A1

C	0.987993000	-2.702900000	-0.727627000
C	2.112817000	-1.744512000	-0.473868000
F	0.908986000	5.616873000	-2.747394000
C	1.378386000	3.336120000	0.014142000
C	1.578890000	4.416491000	-0.836082000
C	0.724145000	4.575784000	-1.910183000
C	-0.320073000	3.700066000	-2.148450000
C	-0.510350000	2.632141000	-1.281759000
C	0.340088000	2.425487000	-0.191066000
H	2.039996000	3.206512000	0.860914000
H	2.378266000	5.125179000	-0.673437000
H	-0.973500000	3.863454000	-2.993579000
H	-1.344191000	1.969605000	-1.451140000

N	0.427086000	-0.105960000	0.220513000
C	1.793268000	-0.286553000	-0.258975000
C	-0.391384000	-2.130160000	-0.843138000
C	-0.525955000	-0.630185000	-0.754231000
C	-1.387691000	-2.995779000	-1.090681000
C	0.195483000	1.238738000	0.758490000
P	-1.305575000	1.362265000	1.844042000
O	-2.536589000	1.566536000	0.838360000
O	-1.615834000	-0.118535000	2.389568000
C	-3.796044000	2.100630000	1.328436000
C	-4.592181000	2.607379000	0.147530000
C	-0.825203000	-0.697495000	3.457809000
C	-1.616544000	-1.825732000	4.080560000
H	-3.579619000	2.895784000	2.040783000
H	-4.324163000	1.300270000	1.849477000
H	0.105764000	-1.064502000	3.022777000
H	-0.599013000	0.080644000	4.187347000
H	-1.031517000	-2.289701000	4.876042000
H	-2.547616000	-1.456042000	4.509630000
H	-1.852172000	-2.589207000	3.339717000
H	-4.062229000	3.412112000	-0.361148000
H	-4.783236000	1.808103000	-0.568419000
H	-5.551800000	2.994931000	0.493107000
H	1.961373000	0.280962000	-1.191291000
H	-1.531843000	-0.345312000	-0.472245000

O	-1.138854000	2.393536000	2.891066000
H	2.477785000	0.126172000	0.483417000
H	-0.345011000	-0.203576000	-1.756417000
H	0.969674000	1.372396000	1.519317000
C	-3.513204000	-3.463279000	-2.248348000
C	-4.944662000	-1.798077000	-0.560151000
C	-5.601418000	-2.467413000	-1.586721000
C	-4.879153000	-3.304217000	-2.430238000
H	-2.958324000	-4.128280000	-2.897791000
H	-5.504701000	-1.169660000	0.119825000
H	-6.668797000	-2.350720000	-1.718187000
H	-5.382014000	-3.842192000	-3.222831000
C	-2.830388000	-2.766674000	-1.241290000
C	-3.574140000	-1.941206000	-0.388065000
H	-3.088165000	-1.441150000	0.437284000
H	-1.066505000	-4.023072000	-1.231550000
C	3.349618000	-2.275478000	-0.421841000
C	4.956581000	-0.310389000	-0.455834000
C	4.651446000	-1.656342000	-0.196324000
C	6.227444000	0.191176000	-0.211562000
C	5.682810000	-2.477411000	0.290605000
C	6.946721000	-1.972127000	0.547984000
C	7.224253000	-0.631654000	0.300250000
H	6.442254000	1.228776000	-0.430153000
H	5.474524000	-3.523574000	0.474041000

H	7.718811000	-2.624506000	0.933399000
H	8.212272000	-0.235198000	0.491676000
H	3.373454000	-3.354167000	-0.532518000
H	4.213134000	0.341844000	-0.886852000
O	1.190142000	-3.902610000	-0.837132000

A2

C	0.417842000	-3.099844000	0.279511000
C	1.600258000	-2.238335000	-0.028352000
F	-5.042437000	3.034471000	1.438742000
C	-1.806172000	1.432438000	1.252159000
C	-2.995193000	1.918716000	1.778320000
C	-3.882255000	2.563813000	0.933761000
C	-3.620356000	2.731615000	-0.413076000
C	-2.425478000	2.236723000	-0.921827000
C	-1.505597000	1.587407000	-0.101834000
H	-1.103203000	0.918352000	1.890481000
H	-3.238953000	1.803537000	2.824879000
H	-4.341450000	3.231647000	-1.043622000
H	-2.211884000	2.357555000	-1.975759000
N	0.045110000	-0.322935000	-0.226394000
C	1.393237000	-0.820799000	-0.496745000
C	-0.924222000	-2.561552000	-0.105231000
C	-0.939879000	-1.236551000	-0.816604000
C	-1.991071000	-3.337568000	0.144705000

C	-0.199801000	1.055607000	-0.669272000
P	1.199030000	2.234073000	-0.335025000
O	0.533388000	3.661624000	-0.652974000
O	1.317803000	2.170563000	1.259509000
C	1.159421000	4.640163000	-1.521241000
C	0.189380000	5.057338000	-2.605677000
C	2.344200000	2.918074000	1.957750000
C	2.343154000	2.485844000	3.406448000
H	1.426274000	5.488735000	-0.890209000
H	2.070391000	4.212583000	-1.936536000
H	2.117972000	3.982327000	1.864664000
H	3.305135000	2.721603000	1.481537000
H	2.563735000	1.422509000	3.493151000
H	3.104004000	3.041347000	3.956650000
H	1.375315000	2.678720000	3.868431000
H	-0.736467000	5.438766000	-2.175871000
H	0.636561000	5.845723000	-3.213604000
H	-0.049938000	4.219804000	-3.261322000
H	1.626006000	-0.746827000	-1.574141000
H	-0.714033000	-1.395028000	-1.888635000
O	2.459919000	2.006995000	-1.084000000
H	2.107771000	-0.182111000	0.013559000
H	-1.922340000	-0.783262000	-0.774264000
H	-0.257820000	1.111679000	-1.771515000
C	-4.062292000	-1.886748000	0.163240000

C	-5.554328000	-4.017030000	-0.812954000
C	-6.173963000	-2.797962000	-0.560763000
C	-5.424325000	-1.738918000	-0.061189000
H	-3.504851000	-1.063972000	0.586972000
H	-6.131061000	-4.852333000	-1.187412000
H	-7.234465000	-2.678741000	-0.737580000
H	-5.901987000	-0.794831000	0.164625000
C	-3.419050000	-3.102318000	-0.103175000
C	-4.196950000	-4.170462000	-0.571767000
H	-3.722739000	-5.126058000	-0.755580000
H	-1.749579000	-4.308559000	0.565890000
C	2.810607000	-2.812164000	0.134366000
C	4.530301000	-1.113354000	-0.687520000
C	4.169210000	-2.322340000	-0.064995000
C	5.864764000	-0.753286000	-0.813814000
C	5.208541000	-3.148170000	0.402783000
C	6.538546000	-2.783480000	0.279067000
C	6.873382000	-1.578947000	-0.330756000
H	6.114010000	0.181728000	-1.297383000
H	4.952740000	-4.088436000	0.873990000
H	7.313791000	-3.438485000	0.653806000
H	7.910771000	-1.289604000	-0.433681000
H	2.760198000	-3.831543000	0.500809000
H	3.787762000	-0.443603000	-1.088643000
O	0.538589000	-4.194367000	0.809448000

A3

C	0.970653000	-2.795429000	-0.010958000
C	-0.431897000	-2.352437000	-0.283634000
F	-1.161827000	3.893020000	-4.743879000
C	-1.619635000	2.065513000	-1.657061000
C	-1.907658000	2.674140000	-2.872012000
C	-0.886376000	3.299898000	-3.563849000
C	0.406665000	3.343095000	-3.076887000
C	0.675780000	2.729961000	-1.859550000
C	-0.323153000	2.077789000	-1.134073000
H	-2.418434000	1.597182000	-1.098645000
H	-2.908769000	2.674295000	-3.279175000
H	1.177427000	3.850247000	-3.639415000
H	1.683982000	2.768430000	-1.467540000
N	0.331235000	-0.059456000	0.102329000
C	-0.696709000	-0.887707000	-0.530049000
C	2.054377000	-1.767587000	-0.147115000
C	1.641116000	-0.354231000	-0.472732000
C	3.320799000	-2.200822000	-0.048603000
C	0.038790000	1.383888000	0.172931000
P	-1.162715000	1.677163000	1.543685000
O	-0.385902000	1.241978000	2.878782000
O	-1.115378000	3.274314000	1.609394000
C	-0.695076000	0.023005000	3.604864000

C	0.586498000	-0.722073000	3.904264000
C	-2.110814000	4.037742000	2.336231000
C	-1.527307000	4.594896000	3.618463000
H	-1.199370000	0.325900000	4.523239000
H	-1.389560000	-0.578517000	3.021483000
H	-2.414091000	4.837290000	1.661517000
H	-2.978916000	3.406054000	2.525391000
H	-2.271314000	5.214917000	4.122014000
H	-1.230344000	3.794650000	4.295169000
H	-0.652986000	5.210964000	3.408970000
H	1.274180000	-0.098263000	4.475699000
H	0.364466000	-1.614106000	4.492435000
H	1.076001000	-1.032022000	2.982423000
H	-0.740355000	-0.691506000	-1.615186000
H	2.363730000	0.355688000	-0.083364000
O	-2.504630000	1.060939000	1.393207000
H	-1.664567000	-0.618352000	-0.112630000
H	1.634623000	-0.223680000	-1.569613000
H	0.950683000	1.850475000	0.549481000
C	4.818546000	-0.256896000	0.551893000
C	6.832580000	-1.312591000	-1.041208000
C	7.046808000	-0.120990000	-0.357398000
C	6.039121000	0.396787000	0.448370000
H	4.059288000	0.137704000	1.212463000
H	7.615219000	-1.731946000	-1.659324000

H	7.995711000	0.391643000	-0.439641000
H	6.206807000	1.308169000	1.006962000
C	4.580006000	-1.447701000	-0.145302000
C	5.619058000	-1.974804000	-0.923803000
H	5.462969000	-2.908795000	-1.448189000
H	3.426146000	-3.273024000	0.083579000
C	-1.371351000	-3.319150000	-0.277809000
C	-3.581787000	-4.336058000	-0.026125000
C	-2.808033000	-3.272933000	-0.522706000
C	-4.955381000	-4.372458000	-0.201377000
C	-3.469816000	-2.264239000	-1.240890000
C	-4.843813000	-2.308464000	-1.426996000
C	-5.593911000	-3.354551000	-0.902092000
H	-5.527922000	-5.197327000	0.201263000
H	-2.910369000	-1.456242000	-1.684495000
H	-5.331223000	-1.522690000	-1.988461000
H	-6.665555000	-3.381809000	-1.046866000
H	-0.990196000	-4.301499000	-0.020485000
H	-3.087082000	-5.135044000	0.511008000
O	1.233394000	-3.948580000	0.295435000

A4

C	0.629713000	-2.969134000	0.336549000
C	1.762035000	-2.069846000	-0.043648000
F	-5.385620000	2.759516000	0.779843000

C	-2.034933000	1.421869000	1.023900000
C	-3.311402000	1.825363000	1.392528000
C	-4.140536000	2.372817000	0.429152000
C	-3.735283000	2.526276000	-0.883519000
C	-2.455098000	2.115349000	-1.233923000
C	-1.590600000	1.561307000	-0.291910000
H	-1.376986000	0.987282000	1.761227000
H	-3.665600000	1.720237000	2.408135000
H	-4.412651000	2.951723000	-1.609996000
H	-2.127342000	2.229595000	-2.258748000
N	0.107278000	-0.238052000	-0.229689000
C	1.469850000	-0.677289000	-0.539631000
C	-0.742212000	-2.521573000	-0.057638000
C	-0.836690000	-1.213283000	-0.794222000
C	-1.756508000	-3.363626000	0.201509000
C	-0.193671000	1.124030000	-0.697805000
P	1.063518000	2.389147000	-0.150561000
O	0.313891000	3.712780000	-0.645708000
O	0.970376000	2.451922000	1.450402000
C	0.994786000	4.991407000	-0.626344000
C	0.181843000	5.971801000	-1.441385000
C	2.099201000	2.193526000	2.325639000
C	1.760556000	1.072781000	3.284543000
H	1.079494000	5.318494000	0.411810000
H	1.996987000	4.862288000	-1.035518000

H	2.971772000	1.960405000	1.717738000
H	2.290422000	3.124355000	2.860201000
H	2.599572000	0.903975000	3.961696000
H	0.884580000	1.323347000	3.882875000
H	1.560219000	0.145274000	2.749181000
H	-0.824120000	6.073471000	-1.035349000
H	0.661662000	6.951462000	-1.425037000
H	0.106423000	5.643944000	-2.477884000
H	1.648935000	-0.623290000	-1.629892000
H	-0.610325000	-1.379373000	-1.864602000
O	2.445270000	2.228872000	-0.668421000
H	2.174582000	0.012907000	-0.088623000
H	-1.840743000	-0.813792000	-0.753205000
H	-0.122452000	1.187207000	-1.797453000
C	-3.889178000	-4.395397000	-0.463874000
C	-5.296542000	-2.033812000	-0.118594000
C	-5.961478000	-3.175123000	-0.552886000
C	-5.251561000	-4.358714000	-0.721812000
H	-3.344116000	-5.323149000	-0.582422000
H	-5.844022000	-1.114666000	0.042404000
H	-7.025924000	-3.146009000	-0.743216000
H	-5.761561000	-5.256814000	-1.044063000
C	-3.195458000	-3.244817000	-0.061541000
C	-3.929985000	-2.066042000	0.122407000
H	-3.441622000	-1.177895000	0.495102000

H	-1.449342000	-4.306310000	0.643668000
C	2.999714000	-2.595236000	0.072365000
C	4.618833000	-0.845440000	-0.843456000
C	4.329180000	-2.070562000	-0.215241000
C	5.932449000	-0.453878000	-1.060271000
C	5.417125000	-2.877447000	0.167392000
C	6.726628000	-2.482551000	-0.048226000
C	6.990723000	-1.264002000	-0.664568000
H	6.126532000	0.494198000	-1.543574000
H	5.216604000	-3.828083000	0.644448000
H	7.540675000	-3.124555000	0.260935000
H	8.011456000	-0.950351000	-0.838162000
H	3.003006000	-3.606488000	0.463983000
H	3.834405000	-0.184287000	-1.172126000
O	0.815131000	-4.025337000	0.922625000

A5

C	0.820522000	-2.285730000	0.292986000
C	-0.571673000	-2.060445000	-0.213142000
F	-4.999190000	3.534487000	-1.718773000
C	-1.546291000	2.548567000	-2.101045000
C	-2.789050000	3.089936000	-2.397953000
C	-3.787327000	3.009301000	-1.442759000
C	-3.578860000	2.408485000	-0.215995000
C	-2.326281000	1.873185000	0.063167000

C	-1.293556000	1.937048000	-0.871256000
H	-0.757945000	2.597306000	-2.841051000
H	-2.988242000	3.569392000	-3.345695000
H	-4.383797000	2.363269000	0.503397000
H	-2.157947000	1.405024000	1.021879000
N	0.244434000	0.047145000	-1.276898000
C	-0.790092000	-0.968041000	-1.237777000
C	1.935112000	-1.558800000	-0.392045000
C	1.572818000	-0.515929000	-1.437307000
C	3.184178000	-1.959765000	-0.105379000
C	0.088373000	1.348800000	-0.638755000
P	0.766184000	1.527776000	1.093028000
O	0.237084000	2.952936000	1.630343000
O	-0.051790000	0.454317000	1.956242000
C	1.129285000	4.035795000	1.992548000
C	1.041412000	5.157590000	0.978855000
C	0.378179000	0.130726000	3.309748000
C	-0.443652000	-1.033561000	3.812384000
H	0.801716000	4.371896000	2.976273000
H	2.142781000	3.646288000	2.072841000
H	1.439522000	-0.112240000	3.281224000
H	0.235257000	1.016748000	3.931679000
H	-0.154289000	-1.260677000	4.839937000
H	-1.507185000	-0.794447000	3.802077000
H	-0.269106000	-1.918288000	3.202781000

H	0.014233000	5.504398000	0.869229000
H	1.656542000	5.998340000	1.304776000
H	1.403611000	4.833168000	0.002864000
H	-0.835232000	-1.420504000	-2.239224000
H	2.302817000	0.286104000	-1.451608000
O	2.245732000	1.406004000	1.141335000
H	-1.754469000	-0.494139000	-1.075421000
H	1.612900000	-0.996433000	-2.425345000
H	0.794513000	2.010682000	-1.155357000
C	5.433232000	-2.341298000	-1.063419000
C	6.003513000	0.371143000	-1.065404000
C	6.945795000	-0.532365000	-1.544494000
C	6.657365000	-1.892732000	-1.538605000
H	5.217810000	-3.402271000	-1.047699000
H	6.233283000	1.428359000	-1.038809000
H	7.903421000	-0.181035000	-1.905217000
H	7.389847000	-2.605105000	-1.894708000
C	4.460913000	-1.438279000	-0.613928000
C	4.770156000	-0.072096000	-0.605426000
H	4.060931000	0.629217000	-0.187083000
H	3.256112000	-2.803753000	0.573394000
C	-1.527804000	-2.871513000	0.279289000
C	-3.760319000	-3.609843000	0.955884000
C	-2.957909000	-2.960351000	0.001521000
C	-5.129432000	-3.735544000	0.785994000

C	-3.590035000	-2.473760000	-1.154719000
C	-4.960049000	-2.610745000	-1.329516000
C	-5.737607000	-3.232544000	-0.359453000
H	-5.722070000	-4.232703000	1.542346000
H	-3.011680000	-2.014132000	-1.939933000
H	-5.421774000	-2.234056000	-2.232522000
H	-6.805291000	-3.334319000	-0.499975000
H	-1.176176000	-3.566453000	1.034340000
H	-3.290521000	-4.012749000	1.843864000
O	1.040707000	-3.047923000	1.225155000

A6

C	0.307458000	-3.211428000	-0.296710000
C	-1.014074000	-2.519489000	-0.186686000
F	4.056270000	3.978035000	-2.080359000
C	1.339849000	1.670280000	-1.472455000
C	2.306179000	2.402468000	-2.149946000
C	3.113139000	3.265998000	-1.429110000
C	2.986872000	3.416770000	-0.059861000
C	2.015530000	2.674780000	0.602018000
C	1.183132000	1.798149000	-0.091135000
H	0.702095000	0.985111000	-2.012022000
H	2.437520000	2.311818000	-3.218801000
H	3.641667000	4.094366000	0.469150000
H	1.906563000	2.782274000	1.673378000

N	0.119946000	-0.393675000	0.211824000
C	-1.058031000	-1.198078000	0.538404000
C	1.518194000	-2.428840000	0.118701000
C	1.316110000	-1.063408000	0.727011000
C	2.705855000	-3.042726000	-0.010540000
C	0.104962000	1.009886000	0.636547000
P	-1.526104000	1.864380000	0.379441000
O	-2.291368000	1.450620000	1.725675000
O	-1.228200000	3.419429000	0.648904000
C	-3.662578000	1.863144000	1.954164000
C	-4.142970000	1.215346000	3.233005000
C	-1.139236000	4.392035000	-0.425407000
C	-1.710512000	5.704503000	0.064018000
H	-3.686740000	2.951587000	2.028639000
H	-4.267165000	1.557158000	1.100047000
H	-0.086948000	4.494913000	-0.690377000
H	-1.683789000	4.011757000	-1.288148000
H	-1.611953000	6.460876000	-0.716272000
H	-2.767459000	5.603728000	0.310558000
H	-1.179534000	6.054479000	0.949134000
H	-3.523255000	1.516584000	4.077279000
H	-5.171202000	1.518802000	3.434940000
H	-4.115996000	0.129335000	3.151417000
H	-1.112396000	-1.388171000	1.626211000
H	1.237828000	-1.176291000	1.826038000

O	-2.226498000	1.594513000	-0.898776000
H	-1.955963000	-0.660438000	0.270938000
H	2.177112000	-0.431566000	0.546743000
H	0.277166000	1.110214000	1.720607000
C	4.947793000	-3.436506000	0.934872000
C	5.837041000	-0.912940000	0.214698000
C	6.692463000	-1.779629000	0.885623000
C	6.243043000	-3.046110000	1.242253000
H	4.607335000	-4.428837000	1.201678000
H	6.186483000	0.064039000	-0.091680000
H	7.705091000	-1.475974000	1.114728000
H	6.905120000	-3.734388000	1.750590000
C	4.060335000	-2.561429000	0.293116000
C	4.535064000	-1.296641000	-0.076749000
H	3.897381000	-0.622700000	-0.630649000
H	2.646183000	-4.070037000	-0.355959000
C	-2.076247000	-3.159776000	-0.704322000
C	-4.465049000	-3.739252000	-0.615083000
C	-3.483629000	-2.756006000	-0.809033000
C	-5.814585000	-3.436240000	-0.717124000
C	-3.900975000	-1.464353000	-1.159620000
C	-5.252722000	-1.169243000	-1.280265000
C	-6.214623000	-2.146589000	-1.049822000
H	-6.553232000	-4.209040000	-0.550149000
H	-3.179204000	-0.688899000	-1.372995000

H	-5.550645000	-0.169649000	-1.567874000
H	-7.266133000	-1.909963000	-1.144188000
H	-1.857333000	-4.153339000	-1.082992000
H	-4.157887000	-4.748840000	-0.373229000
O	0.405825000	-4.346968000	-0.734710000

A7

C	0.289455000	-3.161774000	-0.133881000
C	-1.033654000	-2.469269000	-0.080842000
F	4.066017000	3.697149000	-2.636767000
C	2.157213000	2.572075000	0.216018000
C	3.142227000	3.212944000	-0.528205000
C	3.115662000	3.081758000	-1.903567000
C	2.147899000	2.332324000	-2.551620000
C	1.174465000	1.699223000	-1.793555000
C	1.164060000	1.815960000	-0.401875000
H	2.177352000	2.652963000	1.295845000
H	3.922485000	3.793899000	-0.057801000
H	2.164563000	2.253062000	-3.629182000
H	0.411658000	1.107758000	-2.279268000
N	0.098717000	-0.325853000	0.140241000
C	-1.075065000	-1.099205000	0.546215000
C	1.486950000	-2.367849000	0.291545000
C	1.304634000	-0.930520000	0.705879000
C	2.672525000	-3.005561000	0.243560000

C	0.087081000	1.118066000	0.414138000
P	-1.549210000	1.933296000	0.072594000
O	-2.330285000	1.656545000	1.456728000
O	-1.276774000	3.512929000	0.061556000
C	-3.770657000	1.824339000	1.535238000
C	-4.242438000	1.253170000	2.853161000
C	-0.914294000	4.318070000	1.203016000
C	-1.278218000	5.756345000	0.903517000
H	-4.003287000	2.888604000	1.459426000
H	-4.228028000	1.313412000	0.689002000
H	0.159325000	4.219145000	1.369325000
H	-1.437397000	3.950102000	2.086475000
H	-0.992675000	6.392626000	1.742700000
H	-0.758130000	6.105876000	0.012565000
H	-2.350327000	5.860836000	0.739334000
H	-3.766106000	1.759348000	3.692847000
H	-5.322079000	1.381202000	2.942686000
H	-4.018191000	0.189051000	2.916594000
H	-1.975952000	-0.585261000	0.244887000
H	1.272519000	-0.858798000	1.811389000
O	-2.245875000	1.513973000	-1.160674000
H	-1.117856000	-1.204455000	1.646204000
H	2.168061000	-0.353443000	0.380193000
H	0.274292000	1.323829000	1.482314000
C	5.094119000	-3.244678000	-0.023225000

C	5.646709000	-1.162492000	1.716568000
C	6.691049000	-1.834569000	1.092165000
C	6.408557000	-2.882025000	0.221484000
H	4.879133000	-4.066725000	-0.693584000
H	5.859188000	-0.361515000	2.412229000
H	7.716204000	-1.552763000	1.291433000
H	7.213862000	-3.420255000	-0.260222000
C	4.022045000	-2.558698000	0.572586000
C	4.329138000	-1.515746000	1.460886000
H	3.540184000	-0.999624000	1.984897000
H	2.612017000	-4.019302000	-0.137550000
C	-2.092934000	-3.146887000	-0.553892000
C	-4.485759000	-3.710252000	-0.415071000
C	-3.500036000	-2.751942000	-0.692853000
C	-5.833726000	-3.412122000	-0.548782000
C	-3.910277000	-1.495197000	-1.158871000
C	-5.260629000	-1.206728000	-1.310633000
C	-6.227317000	-2.155743000	-0.996926000
H	-6.576210000	-4.164147000	-0.316638000
H	-3.183495000	-0.745506000	-1.437667000
H	-5.553264000	-0.236511000	-1.689965000
H	-7.277491000	-1.924781000	-1.116838000
H	-1.870536000	-4.163791000	-0.862107000
H	-4.183817000	-4.695788000	-0.083910000
O	0.395967000	-4.317193000	-0.516155000

A8

C	0.935870000	-2.744851000	-0.672814000
C	2.059922000	-1.776360000	-0.458977000
F	0.945435000	5.535843000	-2.843621000
C	-0.502630000	2.577847000	-1.351902000
C	-0.293532000	3.628162000	-2.235593000
C	0.742403000	4.511620000	-1.989772000
C	1.570890000	4.376689000	-0.892097000
C	1.352032000	3.313450000	-0.024890000
C	0.320982000	2.396421000	-0.236578000
H	-1.330544000	1.909607000	-1.527892000
H	-0.926194000	3.772135000	-3.099835000
H	2.364550000	5.090660000	-0.724616000
H	1.993074000	3.202875000	0.840188000
N	0.377229000	-0.125290000	0.219314000
C	1.738112000	-0.315458000	-0.269410000
C	-0.448319000	-2.180625000	-0.773090000
C	-0.586417000	-0.678831000	-0.727405000
C	-1.446721000	-3.057142000	-0.966390000
C	0.155045000	1.228071000	0.733939000
P	-1.348955000	1.417827000	1.803922000
O	-2.556950000	1.572668000	0.760092000
O	-1.673592000	-0.018473000	2.449799000
C	-3.847562000	2.059641000	1.213576000

C	-4.653845000	2.471774000	0.002798000
C	-1.240708000	-0.384598000	3.784938000
C	-0.020395000	-1.280390000	3.738976000
H	-3.682430000	2.896855000	1.891082000
H	-4.341162000	1.257084000	1.764663000
H	-1.058397000	0.525960000	4.354821000
H	-2.089523000	-0.904896000	4.227117000
H	0.230464000	-1.606373000	4.750086000
H	-0.207284000	-2.161156000	3.126326000
H	0.841560000	-0.757369000	3.325916000
H	-4.157231000	3.277557000	-0.536806000
H	-4.795857000	1.631650000	-0.676646000
H	-5.635579000	2.826693000	0.320101000
H	1.897426000	0.233344000	-1.214555000
H	-1.589936000	-0.388318000	-0.442948000
O	-1.187928000	2.511284000	2.787741000
H	2.429644000	0.113232000	0.457442000
H	-0.419157000	-0.282613000	-1.744606000
H	0.925266000	1.366445000	1.498646000
C	-3.601398000	-3.558742000	-2.055638000
C	-4.992602000	-1.861418000	-0.365970000
C	-5.674867000	-2.556287000	-1.358388000
C	-4.972592000	-3.409003000	-2.202806000
H	-3.061970000	-4.236269000	-2.705162000
H	-5.536665000	-1.220504000	0.315354000

H	-6.746035000	-2.446837000	-1.462323000
H	-5.494874000	-3.966624000	-2.968818000
C	-2.893950000	-2.836998000	-1.084086000
C	-3.617093000	-1.995296000	-0.229187000
H	-3.109421000	-1.475449000	0.570687000
H	-1.125392000	-4.087495000	-1.082570000
C	3.300132000	-2.300425000	-0.417893000
C	4.896341000	-0.328594000	-0.517880000
C	4.602894000	-1.671153000	-0.228665000
C	6.169472000	0.182966000	-0.308580000
C	5.647904000	-2.478519000	0.252190000
C	6.914326000	-1.962997000	0.474700000
C	7.180480000	-0.626025000	0.197355000
H	6.374562000	1.217491000	-0.549917000
H	5.448488000	-3.522096000	0.458724000
H	7.697162000	-2.604916000	0.856010000
H	8.170285000	-0.221851000	0.361518000
H	3.327318000	-3.381030000	-0.506882000
H	4.141436000	0.312895000	-0.944936000
O	1.141742000	-3.945632000	-0.761927000

A9

C	0.820457000	-2.285522000	0.293093000
C	1.935029000	-1.558624000	-0.391958000
F	-4.999163000	3.534819000	-1.718651000

C	-2.326284000	1.873425000	0.063235000
C	-3.578830000	2.408795000	-0.215891000
C	-3.787312000	3.009580000	-1.442674000
C	-2.789087000	3.090094000	-2.397918000
C	-1.546346000	2.548659000	-2.101037000
C	-1.293583000	1.937186000	-0.871238000
H	-2.157942000	1.405295000	1.021959000
H	-4.383741000	2.363673000	0.503536000
H	-2.988288000	3.569511000	-3.345676000
H	-0.758032000	2.597306000	-2.841085000
N	0.244359000	0.047254000	-1.276960000
C	1.572739000	-0.515843000	-1.437305000
C	-0.571715000	-2.060307000	-0.213089000
C	-0.790182000	-0.967912000	-1.237728000
C	-1.527816000	-2.871468000	0.279252000
C	0.088338000	1.348907000	-0.638775000
P	0.766224000	1.527819000	1.093006000
O	0.237277000	2.952984000	1.630435000
O	-0.051732000	0.454389000	1.956263000
C	1.129486000	4.035889000	1.992458000
C	1.041286000	5.157710000	0.978820000
C	0.378175000	0.130818000	3.309817000
C	-0.443633000	-1.033503000	3.812380000
H	0.802172000	4.371931000	2.976286000
H	2.143031000	3.646420000	2.072446000

H	1.439531000	-0.112109000	3.281357000
H	0.235202000	1.016862000	3.931700000
H	-0.154385000	-1.260615000	4.839963000
H	-1.507179000	-0.794452000	3.801954000
H	-0.268996000	-1.918216000	3.202778000
H	0.014175000	5.504954000	0.869919000
H	1.657001000	5.998213000	1.304278000
H	1.402605000	4.833099000	0.002575000
H	1.612825000	-0.996451000	-2.425294000
H	-0.835467000	-1.420390000	-2.239161000
O	2.245771000	1.405941000	1.141124000
H	2.302734000	0.286192000	-1.451686000
H	-1.754523000	-0.493975000	-1.075264000
H	0.794498000	2.010768000	-1.155369000
C	-3.590145000	-2.473971000	-1.154758000
C	-5.129284000	-3.736102000	0.785927000
C	-5.737571000	-3.233152000	-0.359479000
C	-4.960136000	-2.611182000	-1.329529000
H	-3.011903000	-2.014215000	-1.939972000
H	-5.721814000	-4.233400000	1.542272000
H	-6.805241000	-3.335096000	-0.499986000
H	-5.421941000	-2.234534000	-2.232512000
C	-2.957900000	-2.960508000	0.001445000
C	-3.760190000	-3.610181000	0.955788000
H	-3.290310000	-4.013061000	1.843737000

H	-1.176146000	-3.566397000	1.034292000
C	3.184099000	-1.959612000	-0.105320000
C	5.433169000	-2.341489000	-1.063085000
C	4.460863000	-1.438298000	-0.613932000
C	6.657357000	-1.893127000	-1.538336000
C	4.770182000	-0.072125000	-0.605797000
C	6.003582000	0.370912000	-1.065831000
C	6.945851000	-0.532782000	-1.544611000
H	7.389822000	-2.605646000	-1.894177000
H	4.060996000	0.629354000	-0.187671000
H	6.233411000	1.428123000	-1.039522000
H	7.903511000	-0.181598000	-1.905382000
H	3.255987000	-2.803598000	0.573464000
H	5.217701000	-3.402448000	-1.047061000
O	1.040645000	-3.047632000	1.225334000

A10

C	0.476550000	-3.135603000	-0.356340000
C	-0.880736000	-2.526402000	-0.213697000
F	3.814611000	4.180444000	-2.220753000
C	1.227297000	1.747435000	-1.544852000
C	2.144673000	2.519294000	-2.245329000
C	2.918636000	3.429817000	-1.546997000
C	2.806002000	3.590428000	-0.177765000
C	1.883470000	2.808688000	0.507487000

C	1.086274000	1.882911000	-0.162720000
H	0.618305000	1.023492000	-2.066805000
H	2.265564000	2.421363000	-3.314726000
H	3.434704000	4.305711000	0.332963000
H	1.785044000	2.923768000	1.579220000
N	0.147119000	-0.359456000	0.189026000
C	-0.979192000	-1.225192000	0.539823000
C	1.637529000	-2.331672000	0.146278000
C	1.389575000	-0.948164000	0.689503000
C	2.850188000	-2.910438000	0.049999000
C	0.063004000	1.048117000	0.592042000
P	-1.614434000	1.811915000	0.351397000
O	-2.334307000	1.381233000	1.717659000
O	-1.395336000	3.384157000	0.592833000
C	-3.719736000	1.729410000	1.965578000
C	-4.136648000	1.099069000	3.275058000
C	-1.361490000	4.341705000	-0.498309000
C	-2.097644000	5.592505000	-0.071472000
H	-3.799811000	2.816933000	2.006532000
H	-4.325803000	1.364927000	1.135861000
H	-0.314019000	4.553141000	-0.712495000
H	-1.816989000	3.888603000	-1.377409000
H	-2.038572000	6.340724000	-0.863565000
H	-3.149547000	5.380926000	0.120413000
H	-1.657907000	6.015139000	0.831909000

H	-3.515881000	1.460424000	4.094624000
H	-5.175171000	1.353457000	3.491600000
H	-4.052509000	0.013964000	3.227081000
H	-0.993687000	-1.433151000	1.625559000
H	1.367336000	-0.975781000	1.796715000
O	-2.321342000	1.485923000	-0.910005000
H	-1.910780000	-0.731091000	0.305639000
H	2.218741000	-0.300357000	0.409912000
H	0.246785000	1.173491000	1.671812000
C	4.438966000	-1.452363000	1.392546000
C	6.578642000	-2.652869000	0.085295000
C	6.814525000	-1.661193000	1.031860000
C	5.740409000	-1.072136000	1.688789000
H	3.627761000	-1.001278000	1.941762000
H	7.407563000	-3.126770000	-0.423215000
H	7.826813000	-1.358506000	1.263672000
H	5.916746000	-0.315364000	2.441647000
C	4.178287000	-2.439739000	0.428664000
C	5.280589000	-3.042688000	-0.201280000
H	5.101882000	-3.821697000	-0.931113000
H	2.834997000	-3.887475000	-0.420817000
C	-1.914209000	-3.218146000	-0.722373000
C	-4.260268000	-3.944099000	-0.583816000
C	-3.345726000	-2.899262000	-0.782445000
C	-5.627902000	-3.721725000	-0.643053000

C	-3.849319000	-1.628468000	-1.093718000
C	-5.219350000	-1.413789000	-1.171251000
C	-6.113870000	-2.452129000	-0.936231000
H	-6.313844000	-4.541119000	-0.473422000
H	-3.181163000	-0.807265000	-1.310974000
H	-5.585185000	-0.428819000	-1.429630000
H	-7.179961000	-2.277856000	-0.996988000
H	-1.644620000	-4.187466000	-1.130070000
H	-3.885940000	-4.937760000	-0.372461000
O	0.637631000	-4.235144000	-0.864016000

A11

C	-0.044111000	2.829495000	0.394565000
C	-1.291909000	2.132896000	-0.048888000
F	5.053912000	-3.744230000	0.690428000
C	2.023732000	-1.797832000	0.987697000
C	3.226396000	-2.401942000	1.325407000
C	3.884080000	-3.154309000	0.368411000
C	3.379820000	-3.316598000	-0.908330000
C	2.177355000	-2.700690000	-1.229594000
C	1.486753000	-1.934166000	-0.293662000
H	1.501021000	-1.206975000	1.725728000
H	3.652656000	-2.298968000	2.312957000
H	3.918978000	-3.915828000	-1.627784000
H	1.766776000	-2.831655000	-2.221766000

N	0.068672000	0.112241000	-0.259740000
C	-1.156133000	0.782949000	-0.703633000
C	1.247732000	2.248224000	-0.080208000
C	1.212764000	0.896402000	-0.742706000
C	2.356689000	2.978536000	0.145060000
C	0.153272000	-1.301591000	-0.663195000
P	-1.132832000	-2.459292000	0.048424000
O	-1.219738000	-2.205696000	1.627563000
O	-2.533416000	-1.939962000	-0.565388000
C	-1.826070000	-1.068282000	2.286819000
C	-2.047226000	-1.431488000	3.738688000
C	-3.650059000	-2.866258000	-0.693702000
C	-3.720228000	-3.433835000	-2.095152000
H	-1.158163000	-0.213603000	2.187025000
H	-2.768887000	-0.829983000	1.793179000
H	-3.546325000	-3.662277000	0.042747000
H	-4.534992000	-2.278741000	-0.452883000
H	-2.834047000	-4.029055000	-2.308315000
H	-4.598804000	-4.075069000	-2.188471000
H	-3.799893000	-2.635814000	-2.833839000
H	-1.103906000	-1.680783000	4.223582000
H	-2.492115000	-0.585689000	4.265094000
H	-2.716223000	-2.286905000	3.828887000
H	-1.152938000	0.881281000	-1.806196000
H	1.162661000	0.997440000	-1.843569000

O	-0.832805000	-3.871038000	-0.256567000
H	-2.018192000	0.174553000	-0.456933000
H	2.132102000	0.365482000	-0.519114000
H	0.018908000	-1.412344000	-1.751842000
C	4.186784000	1.914348000	-1.252468000
C	6.083413000	3.217994000	0.306899000
C	6.493096000	2.385059000	-0.729113000
C	5.539354000	1.743772000	-1.511217000
H	3.468332000	1.432553000	-1.897145000
H	6.817198000	3.731821000	0.913345000
H	7.546292000	2.246451000	-0.932900000
H	5.849680000	1.111232000	-2.332096000
C	3.753089000	2.736248000	-0.200279000
C	4.733087000	3.399110000	0.557805000
H	4.418254000	4.055660000	1.358648000
H	2.179155000	3.890315000	0.705341000
C	-2.453749000	2.769830000	0.190971000
C	-4.212743000	1.541449000	-1.163541000
C	-3.831725000	2.408916000	-0.127679000
C	-5.551427000	1.263308000	-1.402686000
C	-4.851578000	3.001091000	0.636518000
C	-6.186913000	2.711645000	0.406190000
C	-6.543306000	1.837543000	-0.615502000
H	-5.821695000	0.602438000	-2.215628000
H	-4.579603000	3.692033000	1.424060000

H	-6.950992000	3.174837000	1.016018000
H	-7.585099000	1.617069000	-0.805618000
H	-2.343491000	3.700396000	0.737497000
H	-3.466519000	1.107235000	-1.810042000
O	-0.083416000	3.843410000	1.075286000

A12

C	0.307434000	-3.211673000	-0.295986000
C	-1.014092000	-2.519652000	-0.186338000
F	4.055971000	3.977975000	-2.080863000
C	1.339513000	1.670327000	-1.472654000
C	2.305770000	2.402519000	-2.150255000
C	3.112924000	3.265942000	-1.429504000
C	2.986892000	3.416607000	-0.060219000
C	2.015616000	2.674627000	0.601772000
C	1.183049000	1.798089000	-0.091295000
H	0.701600000	0.985270000	-2.012180000
H	2.436902000	2.311963000	-3.219147000
H	3.641815000	4.094125000	0.468736000
H	1.906817000	2.782068000	1.673154000
N	0.119954000	-0.393791000	0.211980000
C	-1.058051000	-1.198131000	0.538567000
C	1.518212000	-2.428937000	0.119108000
C	1.316103000	-1.063457000	0.727290000
C	2.705895000	-3.042755000	-0.010297000

C	0.104936000	1.009842000	0.636507000
P	-1.526172000	1.864203000	0.379358000
O	-2.291411000	1.450437000	1.725620000
O	-1.228443000	3.419288000	0.648782000
C	-3.662935000	1.862175000	1.953670000
C	-4.141600000	1.218177000	3.235072000
C	-1.139401000	4.391894000	-0.425499000
C	-1.710536000	5.704400000	0.063961000
H	-3.688401000	2.950838000	2.024448000
H	-4.267708000	1.552539000	1.101010000
H	-0.087121000	4.494652000	-0.690540000
H	-1.684052000	4.011701000	-1.288233000
H	-1.611933000	6.460748000	-0.716353000
H	-2.767498000	5.603691000	0.310476000
H	-1.179543000	6.054371000	0.949064000
H	-3.521805000	1.523247000	4.077907000
H	-5.170152000	1.520855000	3.436558000
H	-4.113120000	0.131922000	3.157271000
H	-1.955962000	-0.660512000	0.270976000
H	1.237761000	-1.176249000	1.826321000
O	-2.226617000	1.594239000	-0.898837000
H	-1.112497000	-1.388104000	1.626395000
H	2.177128000	-0.431639000	0.547005000
H	0.277160000	1.110317000	1.720554000
C	4.947998000	-3.436202000	0.934918000

C	5.836895000	-0.912614000	0.214358000
C	6.692482000	-1.779135000	0.885290000
C	6.243241000	-3.045626000	1.242109000
H	4.607681000	-4.428539000	1.201881000
H	6.186190000	0.064376000	-0.092156000
H	7.705099000	-1.475341000	1.114264000
H	6.905446000	-3.733772000	1.750459000
C	4.060390000	-2.561298000	0.293147000
C	4.534929000	-1.296499000	-0.076913000
H	3.897093000	-0.622686000	-0.630801000
H	2.646287000	-4.070097000	-0.355644000
C	-2.076182000	-3.159967000	-0.704113000
C	-4.464979000	-3.739454000	-0.615387000
C	-3.483546000	-2.756181000	-0.809129000
C	-5.814506000	-3.436472000	-0.717660000
C	-3.900846000	-1.464528000	-1.159759000
C	-5.252585000	-1.169447000	-1.280618000
C	-6.214512000	-2.146818000	-1.050374000
H	-6.553169000	-4.209291000	-0.550846000
H	-3.179072000	-0.689041000	-1.373012000
H	-5.550477000	-0.169846000	-1.568241000
H	-7.266011000	-1.910211000	-1.144909000
H	-1.857263000	-4.153603000	-1.082586000
H	-4.157838000	-4.749042000	-0.373507000
O	0.405812000	-4.347403000	-0.733490000

A13

C	1.000390000	-2.717478000	-0.452995000
C	2.098358000	-1.698954000	-0.417750000
F	-0.165995000	5.246347000	-3.421173000
C	1.026997000	3.282419000	-0.634299000
C	0.988835000	4.255931000	-1.624456000
C	-0.115453000	4.307780000	-2.453830000
C	-1.173944000	3.428338000	-2.315530000
C	-1.119145000	2.465281000	-1.316449000
C	-0.016003000	2.369976000	-0.463215000
H	1.884413000	3.241068000	0.025156000
H	1.792952000	4.966297000	-1.752771000
H	-2.025954000	3.508896000	-2.975460000
H	-1.958013000	1.799096000	-1.187747000
N	0.379861000	-0.076575000	0.166685000
C	1.712470000	-0.242383000	-0.401323000
C	-0.388512000	-2.214098000	-0.694801000
C	-0.618713000	-0.723930000	-0.675462000
C	-1.339559000	-3.145160000	-0.895049000
C	0.109850000	1.293564000	0.612482000
P	-1.188248000	1.428065000	1.929448000
O	-2.557293000	1.105101000	1.157634000
O	-1.033254000	0.136508000	2.869751000
C	-3.831062000	1.168230000	1.855466000

C	-4.472347000	2.532217000	1.705071000
C	-0.393586000	0.189804000	4.167982000
C	0.968992000	-0.470674000	4.125939000
H	-4.438397000	0.385682000	1.403798000
H	-3.678152000	0.913405000	2.904615000
H	-1.061809000	-0.343865000	4.843511000
H	-0.333274000	1.229042000	4.489560000
H	1.657769000	0.079276000	3.484504000
H	1.393545000	-0.498225000	5.131204000
H	0.895007000	-1.491477000	3.753276000
H	-4.622081000	2.775936000	0.653252000
H	-5.446661000	2.533494000	2.197615000
H	-3.850656000	3.302596000	2.157756000
H	1.771245000	0.187227000	-1.417388000
H	-0.593152000	-0.320978000	-1.703487000
O	-1.118491000	2.716532000	2.654891000
H	2.424927000	0.310355000	0.211817000
H	-1.607887000	-0.517616000	-0.275859000
H	0.982262000	1.570568000	1.211270000
C	-3.356253000	-1.891239000	-1.785703000
C	-4.966129000	-4.042921000	-1.075935000
C	-5.535401000	-2.917888000	-1.663527000
C	-4.722817000	-1.849316000	-2.025335000
H	-2.743551000	-1.064637000	-2.110243000
H	-5.586769000	-4.886026000	-0.803273000

H	-6.600016000	-2.880469000	-1.851425000
H	-5.153898000	-0.981584000	-2.506995000
C	-2.765140000	-3.010089000	-1.178180000
C	-3.600006000	-4.091137000	-0.849301000
H	-3.160133000	-4.973225000	-0.402167000
H	-0.989696000	-4.167312000	-0.798403000
C	3.358546000	-2.169870000	-0.373213000
C	4.853584000	-0.173970000	-0.838663000
C	4.639120000	-1.470037000	-0.343216000
C	6.108489000	0.415270000	-0.771536000
C	5.742065000	-2.154200000	0.194902000
C	6.991108000	-1.559882000	0.274147000
C	7.179297000	-0.268288000	-0.206929000
H	6.252614000	1.410825000	-1.169970000
H	5.602554000	-3.163426000	0.560328000
H	7.820662000	-2.106063000	0.702883000
H	8.154767000	0.196222000	-0.154769000
H	3.427928000	-3.250789000	-0.315569000
H	4.048871000	0.364360000	-1.314939000
O	1.235918000	-3.909954000	-0.330248000

A14

C	1.102752000	-2.869580000	-0.195997000
C	-0.287236000	-2.415304000	0.139358000
F	-4.030270000	1.800323000	-4.241245000

C	-2.011190000	2.439151000	-1.306916000
C	-3.011972000	2.573252000	-2.263224000
C	-3.057620000	1.677661000	-3.313498000
C	-2.132035000	0.657689000	-3.437695000
C	-1.138172000	0.539654000	-2.475876000
C	-1.060775000	1.421335000	-1.393419000
H	-1.985592000	3.147675000	-0.494776000
H	-3.750412000	3.359369000	-2.195095000
H	-2.191533000	-0.025927000	-4.272656000
H	-0.410831000	-0.254633000	-2.556486000
N	0.467333000	-0.139962000	-0.235064000
C	-0.467051000	-0.967512000	0.522029000
C	2.202717000	-1.854569000	-0.108932000
C	1.848410000	-0.411819000	0.151406000
C	3.456277000	-2.315317000	-0.285617000
C	0.110099000	1.261130000	-0.416267000
P	0.029497000	2.215375000	1.187224000
O	-0.047690000	3.778002000	0.784890000
O	-1.439112000	1.899195000	1.745463000
C	1.001952000	4.725668000	1.109512000
C	1.687497000	5.208554000	-0.151548000
C	-1.816560000	2.301974000	3.087305000
C	-3.189485000	1.741301000	3.381050000
H	0.509637000	5.549409000	1.626219000
H	1.703183000	4.252636000	1.795049000

H	-1.068660000	1.926058000	3.785298000
H	-1.817580000	3.392889000	3.133084000
H	-3.499946000	2.035899000	4.384630000
H	-3.923916000	2.117581000	2.669398000
H	-3.183776000	0.653226000	3.328340000
H	0.967651000	5.637560000	-0.848193000
H	2.421211000	5.976299000	0.100320000
H	2.211495000	4.394280000	-0.652786000
H	-0.301481000	-0.858554000	1.607157000
H	2.020948000	-0.152533000	1.204847000
O	1.147772000	1.906334000	2.111513000
H	-1.478247000	-0.629417000	0.330714000
H	2.502544000	0.231942000	-0.439300000
H	0.983321000	1.729493000	-0.885501000
C	4.989424000	-0.415590000	0.413746000
C	7.079247000	-1.660606000	-0.934658000
C	7.298970000	-0.450949000	-0.283721000
C	6.252078000	0.160560000	0.396430000
H	4.207169000	0.065114000	0.979706000
H	7.889664000	-2.152194000	-1.456181000
H	8.280306000	0.004087000	-0.295412000
H	6.420074000	1.089163000	0.925672000
C	4.743206000	-1.628395000	-0.250986000
C	5.823127000	-2.244074000	-0.906915000
H	5.659733000	-3.190907000	-1.405275000

H	3.512727000	-3.372103000	-0.522830000
C	-1.246130000	-3.355464000	0.145223000
C	-3.286965000	-4.261823000	1.186262000
C	-2.682811000	-3.247005000	0.430595000
C	-4.642101000	-4.227470000	1.480592000
C	-3.498521000	-2.220064000	-0.062001000
C	-4.858200000	-2.195858000	0.217363000
C	-5.434243000	-3.191277000	0.998776000
H	-5.082315000	-5.015893000	2.076570000
H	-3.078036000	-1.457205000	-0.701170000
H	-5.470838000	-1.400436000	-0.185776000
H	-6.493472000	-3.167913000	1.217051000
H	-0.891485000	-4.360984000	-0.057986000
H	-2.678251000	-5.079198000	1.551182000
O	1.329931000	-4.027290000	-0.513338000

A15

C	1.061596000	-2.488447000	-0.786263000
C	2.170975000	-1.495614000	-0.629805000
F	-0.828334000	5.584972000	-2.843560000
C	-1.372114000	2.695564000	-0.740840000
C	-1.622150000	3.724240000	-1.639417000
C	-0.590517000	4.583278000	-1.972459000
C	0.674051000	4.449419000	-1.431110000
C	0.903583000	3.413294000	-0.534774000

C	-0.105735000	2.516869000	-0.178593000
H	-2.182134000	2.038726000	-0.460049000
H	-2.601737000	3.871918000	-2.071237000
H	1.452483000	5.148103000	-1.702318000
H	1.887074000	3.312471000	-0.094032000
N	0.478908000	0.055042000	0.186763000
C	1.800947000	-0.048877000	-0.426870000
C	-0.321244000	-1.940255000	-0.956194000
C	-0.538201000	-0.465982000	-0.722763000
C	-1.279852000	-2.823367000	-1.293945000
C	0.218153000	1.377482000	0.780132000
P	-0.883877000	1.320259000	2.267855000
O	-2.265191000	0.665190000	1.739842000
O	-0.237542000	0.240083000	3.258005000
C	-3.470902000	0.814149000	2.539070000
C	-4.668311000	0.521408000	1.663897000
C	-0.293350000	-1.200415000	3.135482000
C	-0.097196000	-1.798193000	4.511828000
H	-3.416345000	0.115648000	3.376412000
H	-3.497189000	1.827554000	2.937568000
H	0.488408000	-1.514792000	2.447046000
H	-1.255248000	-1.488347000	2.710459000
H	0.859541000	-1.490062000	4.932481000
H	-0.888150000	-1.484052000	5.192690000
H	-0.109049000	-2.887233000	4.444726000

H	-4.615117000	-0.482613000	1.244110000
H	-5.581101000	0.598637000	2.256608000
H	-4.734949000	1.235889000	0.843570000
H	1.839502000	0.504165000	-1.381881000
H	-1.518954000	-0.313839000	-0.280422000
O	-1.020321000	2.633467000	2.924699000
H	2.530281000	0.416536000	0.236022000
H	-0.525859000	0.081072000	-1.680945000
H	1.150347000	1.652155000	1.280187000
C	-3.274643000	-1.432137000	-2.011037000
C	-4.911159000	-3.649182000	-1.643863000
C	-5.462933000	-2.444810000	-2.068526000
C	-4.637114000	-1.342798000	-2.259979000
H	-2.651473000	-0.572057000	-2.200398000
H	-5.542290000	-4.516813000	-1.505360000
H	-6.524391000	-2.369911000	-2.262741000
H	-5.054792000	-0.409780000	-2.614370000
C	-2.701051000	-2.634094000	-1.567456000
C	-3.548562000	-3.743682000	-1.410422000
H	-3.121490000	-4.686498000	-1.093709000
H	-0.939825000	-3.852154000	-1.345112000
C	3.426053000	-1.982121000	-0.647855000
C	4.938431000	0.034542000	-0.909779000
C	4.713159000	-1.302062000	-0.544771000
C	6.198422000	0.603402000	-0.787327000

C	5.809782000	-2.046510000	-0.079139000
C	7.063884000	-1.473355000	0.057008000
C	7.263188000	-0.142301000	-0.294568000
H	6.351455000	1.631853000	-1.086306000
H	5.661741000	-3.085735000	0.184702000
H	7.888860000	-2.066208000	0.428684000
H	8.242857000	0.305915000	-0.198181000
H	3.484068000	-3.062887000	-0.719955000
H	4.138319000	0.623366000	-1.331227000
O	1.280906000	-3.690191000	-0.812310000

A16

C	0.899912000	-2.702263000	-0.483005000
C	2.034358000	-1.724876000	-0.459866000
F	-0.038690000	5.356971000	-3.272904000
C	1.148457000	3.292314000	-0.557347000
C	1.122854000	4.286452000	-1.527100000
C	0.000310000	4.397803000	-2.325453000
C	-1.087813000	3.557269000	-2.175615000
C	-1.044929000	2.572578000	-1.197177000
C	0.075672000	2.417017000	-0.375964000
H	2.020256000	3.205539000	0.078354000
H	1.950387000	4.967976000	-1.662669000
H	-1.952860000	3.683961000	-2.810892000
H	-1.905945000	1.937191000	-1.059561000

N	0.393167000	-0.055100000	0.197172000
C	1.698259000	-0.256854000	-0.421849000
C	-0.474204000	-2.145354000	-0.692523000
C	-0.657627000	-0.649831000	-0.619052000
C	-1.454194000	-3.039365000	-0.921071000
C	0.189316000	1.315314000	0.675287000
P	-1.058684000	1.475884000	2.037370000
O	-2.474621000	1.279303000	1.305677000
O	-0.961260000	0.137736000	2.918276000
C	-3.699061000	1.451154000	2.068034000
C	-4.860858000	1.502405000	1.102231000
C	-0.268273000	0.089565000	4.189170000
C	1.057933000	-0.630734000	4.057765000
H	-3.791711000	0.609248000	2.755645000
H	-3.619645000	2.371041000	2.647453000
H	-0.144826000	1.105921000	4.561886000
H	-0.935824000	-0.447859000	4.862173000
H	1.513966000	-0.739433000	5.043581000
H	0.920989000	-1.622211000	3.628144000
H	1.751628000	-0.076281000	3.425617000
H	-4.922233000	0.585910000	0.516029000
H	-5.793041000	1.617333000	1.657361000
H	-4.762646000	2.348520000	0.422238000
H	1.732835000	0.179069000	-1.436265000
H	-0.663586000	-0.217187000	-1.635093000

O	-0.885233000	2.728607000	2.805734000
H	2.449962000	0.268468000	0.167790000
H	-1.623316000	-0.427686000	-0.171511000
H	1.088943000	1.546849000	1.252558000
C	-3.735438000	-3.933544000	-0.928156000
C	-4.804053000	-1.609088000	-1.987813000
C	-5.641215000	-2.677673000	-1.686845000
C	-5.099100000	-3.843952000	-1.156894000
H	-3.316715000	-4.847690000	-0.527526000
H	-5.213996000	-0.707893000	-2.424331000
H	-6.703756000	-2.607401000	-1.876947000
H	-5.738932000	-4.686640000	-0.931449000
C	-2.875560000	-2.855256000	-1.196682000
C	-3.440005000	-1.692744000	-1.744890000
H	-2.809174000	-0.862155000	-2.020243000
H	-1.133867000	-4.074045000	-0.863646000
C	3.277741000	-2.239813000	-0.442652000
C	4.829384000	-0.294646000	-0.931990000
C	4.581206000	-1.582950000	-0.432297000
C	6.103355000	0.254230000	-0.884545000
C	5.669700000	-2.302423000	0.088668000
C	6.938253000	-1.748335000	0.148174000
C	7.160252000	-0.463357000	-0.336178000
H	6.273104000	1.244626000	-1.285714000
H	5.503598000	-3.306747000	0.456528000

H	7.756483000	-2.320862000	0.564109000
H	8.150826000	-0.030434000	-0.299319000
H	3.311247000	-3.322881000	-0.393894000
H	4.034842000	0.268667000	-1.396557000
O	1.093803000	-3.903953000	-0.379018000

A17

C	-0.044242000	2.829466000	0.394490000
C	-1.292046000	2.132872000	-0.049017000
F	5.054263000	-3.743782000	0.689693000
C	2.023884000	-1.797763000	0.987458000
C	3.226660000	-2.401759000	1.324989000
C	3.884310000	-3.153996000	0.367871000
C	3.379901000	-3.316287000	-0.908814000
C	2.177325000	-2.700504000	-1.229895000
C	1.486755000	-1.934102000	-0.293834000
H	1.501208000	-1.207001000	1.725590000
H	3.653026000	-2.298795000	2.312494000
H	3.919039000	-3.915426000	-1.628359000
H	1.766621000	-2.831468000	-2.222015000
N	0.068595000	0.112256000	-0.259850000
C	-1.156243000	0.782927000	-0.703770000
C	1.247630000	2.248227000	-0.080250000
C	1.212661000	0.896449000	-0.742840000
C	2.356573000	2.978506000	0.145136000

C	0.153180000	-1.301608000	-0.663180000
P	-1.132793000	-2.459255000	0.048762000
O	-1.219549000	-2.205445000	1.627872000
O	-2.533469000	-1.940016000	-0.564943000
C	-1.825794000	-1.067949000	2.287050000
C	-2.046979000	-1.431045000	3.738945000
C	-3.650112000	-2.866337000	-0.693047000
C	-3.720142000	-3.434382000	-2.094328000
H	-1.157821000	-0.213329000	2.187206000
H	-2.768590000	-0.829601000	1.793391000
H	-4.535041000	-2.278716000	-0.452499000
H	-3.546446000	-3.662123000	0.043661000
H	-4.598788000	-4.075520000	-2.187596000
H	-3.799541000	-2.636590000	-2.833296000
H	-2.833994000	-4.029784000	-2.307136000
H	-1.103682000	-1.680439000	4.223835000
H	-2.491741000	-0.585155000	4.265312000
H	-2.716086000	-2.286368000	3.829202000
H	-2.018294000	0.174510000	-0.457102000
H	1.162484000	0.997572000	-1.843688000
O	-0.832780000	-3.871046000	-0.256050000
H	-1.152994000	0.881255000	-1.806331000
H	2.132018000	0.365529000	-0.519350000
H	0.018643000	-1.412474000	-1.751792000
C	4.186691000	1.914326000	-1.252353000

C	6.083303000	3.217771000	0.307204000
C	6.493000000	2.384865000	-0.728827000
C	5.539265000	1.743678000	-1.511025000
H	3.468246000	1.432614000	-1.897102000
H	6.817082000	3.731523000	0.913721000
H	7.546200000	2.246200000	-0.932552000
H	5.849603000	1.111156000	-2.331914000
C	3.752981000	2.736190000	-0.200143000
C	4.732971000	3.398951000	0.558038000
H	4.418127000	4.055474000	1.358900000
H	2.179026000	3.890249000	0.705472000
C	-2.453890000	2.769788000	0.190853000
C	-4.212686000	1.541570000	-1.163999000
C	-3.831835000	2.408819000	-0.127894000
C	-5.551322000	1.263323000	-1.403292000
C	-4.851791000	3.000679000	0.636403000
C	-6.187076000	2.711120000	0.405929000
C	-6.543307000	1.837235000	-0.616007000
H	-5.821464000	0.602633000	-2.216421000
H	-4.579940000	3.691448000	1.424139000
H	-6.951251000	3.174061000	1.015828000
H	-7.585066000	1.616691000	-0.806230000
H	-2.343669000	3.700325000	0.737434000
H	-3.466359000	1.107639000	-1.810573000
O	-0.083571000	3.843355000	1.075240000

A18

C	0.640936000	-2.955173000	0.434099000
C	1.769727000	-2.088075000	-0.023118000
F	-5.408355000	2.816763000	0.404315000
C	-2.083622000	1.472361000	0.863905000
C	-3.371855000	1.900513000	1.154302000
C	-4.152946000	2.404297000	0.128702000
C	-3.688402000	2.488952000	-1.170351000
C	-2.397163000	2.053683000	-1.441864000
C	-1.578830000	1.543510000	-0.435928000
H	-1.462910000	1.076281000	1.653538000
H	-3.771279000	1.849679000	2.157220000
H	-4.328908000	2.881965000	-1.946790000
H	-2.023383000	2.115079000	-2.455406000
N	0.122050000	-0.258870000	-0.251179000
C	1.466461000	-0.730218000	-0.600974000
C	-0.737069000	-2.527582000	0.036896000
C	-0.850318000	-1.245531000	-0.743621000
C	-1.742240000	-3.369016000	0.332634000
C	-0.165632000	1.094088000	-0.759984000
P	1.063582000	2.353956000	-0.136020000
O	0.331745000	3.698545000	-0.588938000
O	0.925942000	2.398271000	1.464452000
C	1.016348000	4.973536000	-0.494985000

C	0.252119000	5.981100000	-1.323569000
C	1.848233000	1.688452000	2.332439000
C	1.838025000	2.359042000	3.687782000
H	1.045484000	5.266408000	0.555723000
H	2.038568000	4.850411000	-0.853156000
H	2.839387000	1.711247000	1.881392000
H	1.516584000	0.651622000	2.401052000
H	0.837463000	2.353141000	4.119676000
H	2.506944000	1.825975000	4.365027000
H	2.176639000	3.392300000	3.613329000
H	-0.774817000	6.073709000	-0.971382000
H	0.731903000	6.958116000	-1.249294000
H	0.234210000	5.686357000	-2.372520000
H	1.581168000	-0.755944000	-1.701526000
H	-0.667435000	-1.451897000	-1.815124000
O	2.457833000	2.197172000	-0.618641000
H	2.198911000	-0.015658000	-0.242121000
H	-1.848860000	-0.837431000	-0.678445000
H	-0.035742000	1.139911000	-1.854810000
C	-3.933607000	-2.101783000	0.203566000
C	-5.228930000	-4.450114000	-0.519265000
C	-5.953744000	-3.269116000	-0.404733000
C	-5.301612000	-2.099366000	-0.030871000
H	-3.456647000	-1.189293000	0.528837000
H	-5.728365000	-5.369678000	-0.793985000

H	-7.019383000	-3.263123000	-0.590369000
H	-5.860387000	-1.180480000	0.087124000
C	-3.183905000	-3.278358000	0.073949000
C	-3.864936000	-4.456064000	-0.267625000
H	-3.308316000	-5.381409000	-0.343454000
H	-1.422345000	-4.292082000	0.805923000
C	3.006743000	-2.612425000	0.102736000
C	4.615326000	-0.951760000	-0.979218000
C	4.333240000	-2.118213000	-0.245955000
C	5.925473000	-0.582526000	-1.248389000
C	5.425552000	-2.893832000	0.185282000
C	6.732177000	-2.521694000	-0.082696000
C	6.988536000	-1.359606000	-0.802566000
H	6.113603000	0.321421000	-1.811878000
H	5.231141000	-3.800932000	0.742851000
H	7.549985000	-3.137948000	0.266353000
H	8.006876000	-1.064333000	-1.017406000
H	3.012398000	-3.595310000	0.561004000
H	3.825227000	-0.321043000	-1.350568000
O	0.832593000	-3.973950000	1.081304000

A19

C	0.976713000	-2.863222000	-0.127212000
C	2.056496000	-1.817736000	-0.157285000
F	-0.003431000	4.490383000	-4.301312000

C	-1.147432000	2.118594000	-1.822317000
C	-1.122451000	2.905403000	-2.966581000
C	-0.030299000	3.723638000	-3.192039000
C	1.031598000	3.785286000	-2.308850000
C	0.990632000	2.989840000	-1.170651000
C	-0.088331000	2.142910000	-0.909410000
H	-2.013863000	1.504925000	-1.625167000
H	-1.939266000	2.895097000	-3.674085000
H	1.863848000	4.443956000	-2.512054000
H	1.814572000	3.036890000	-0.469672000
N	0.284925000	-0.158628000	0.140541000
C	1.663083000	-0.366893000	-0.285725000
C	-0.421471000	-2.414767000	-0.408901000
C	-0.622605000	-0.948928000	-0.694494000
C	-1.366336000	-3.371410000	-0.454711000
C	-0.054822000	1.257288000	0.331827000
P	-1.588291000	1.394902000	1.358610000
O	-1.378640000	0.474994000	2.651535000
O	-1.438412000	2.915942000	1.855717000
C	-0.201464000	0.498546000	3.487522000
C	-0.502918000	-0.282232000	4.747904000
C	-2.588634000	3.658579000	2.336995000
C	-2.263051000	5.133481000	2.269753000
H	0.619138000	0.041042000	2.933342000
H	0.056055000	1.534556000	3.718231000

H	-3.449407000	3.405858000	1.718962000
H	-2.795623000	3.344805000	3.361828000
H	-3.107957000	5.714076000	2.643096000
H	-1.389881000	5.369199000	2.878118000
H	-2.062418000	5.438273000	1.243188000
H	-0.771177000	-1.309741000	4.505673000
H	0.376943000	-0.297767000	5.392799000
H	-1.327424000	0.170354000	5.298092000
H	1.809736000	-0.022449000	-1.324524000
H	-0.435269000	-0.759367000	-1.765575000
O	-2.873514000	1.061309000	0.702483000
H	2.319536000	0.240348000	0.340572000
H	-1.642076000	-0.645323000	-0.500005000
H	0.724425000	1.661108000	0.983135000
C	-3.385570000	-4.417840000	-1.361209000
C	-5.010910000	-2.307288000	-0.612595000
C	-5.559688000	-3.394362000	-1.283778000
C	-4.739858000	-4.454191000	-1.655425000
H	-2.753544000	-5.251751000	-1.639212000
H	-5.641874000	-1.487347000	-0.296781000
H	-6.618814000	-3.421626000	-1.502974000
H	-5.156984000	-5.312888000	-2.164617000
C	-2.807235000	-3.311856000	-0.717728000
C	-3.652292000	-2.260264000	-0.332404000
H	-3.273953000	-1.412442000	0.218352000

H	-0.979624000	-4.375503000	-0.312652000
C	3.321885000	-2.261029000	-0.035109000
C	4.811864000	-0.278613000	-0.586329000
C	4.594685000	-1.546605000	-0.022939000
C	6.062073000	0.321678000	-0.529025000
C	5.691798000	-2.190514000	0.574468000
C	6.935719000	-1.584577000	0.643381000
C	7.126044000	-0.321189000	0.093225000
H	6.208113000	1.293866000	-0.980997000
H	5.551489000	-3.178579000	0.993409000
H	7.759878000	-2.100316000	1.117811000
H	8.097731000	0.152055000	0.136917000
H	3.402386000	-3.332424000	0.113383000
H	4.014789000	0.227903000	-1.107521000
O	1.242811000	-4.032712000	0.105970000

A20

C	0.976381000	-2.863457000	-0.127832000
C	2.056256000	-1.818059000	-0.158210000
F	-0.005803000	4.491416000	-4.300412000
C	-1.148177000	2.118739000	-1.821486000
C	-1.123891000	2.905854000	-2.965563000
C	-0.032012000	3.724373000	-3.191332000
C	1.030296000	3.785978000	-2.308615000
C	0.990042000	2.990216000	-1.170613000

C	-0.088648000	2.143031000	-0.909076000
H	-2.014424000	1.504907000	-1.624054000
H	-1.941052000	2.895563000	-3.672673000
H	1.862329000	4.444855000	-2.512052000
H	1.814340000	3.037212000	-0.470049000
N	0.285060000	-0.158790000	0.140248000
C	1.662874000	-0.367235000	-0.286974000
C	-0.421881000	-2.414873000	-0.409028000
C	-0.623226000	-0.948950000	-0.694033000
C	-1.366742000	-3.371520000	-0.455089000
C	-0.054405000	1.257111000	0.331993000
P	-1.587221000	1.394872000	1.359747000
O	-1.376960000	0.474767000	2.652432000
O	-1.436838000	2.915818000	1.856954000
C	-0.199349000	0.497949000	3.487853000
C	-0.500080000	-0.283701000	4.747858000
C	-2.586712000	3.659020000	2.338283000
C	-2.261887000	5.133923000	2.267528000
H	0.621070000	0.040934000	2.932993000
H	0.058099000	1.533862000	3.719078000
H	-3.448256000	3.404625000	1.722011000
H	-2.792014000	3.347248000	3.364063000
H	-3.106453000	5.714901000	2.641070000
H	-1.387848000	5.371252000	2.874017000
H	-2.063052000	5.436740000	1.240031000

H	-0.768293000	-1.311090000	4.505065000
H	0.380097000	-0.299541000	5.392327000
H	-1.324383000	0.168398000	5.298746000
H	1.808762000	-0.023213000	-1.326036000
H	-0.437013000	-0.759176000	-1.765290000
O	-2.872884000	1.061573000	0.704344000
H	2.319838000	0.240234000	0.338556000
H	-1.642498000	-0.645376000	-0.498460000
H	0.725361000	1.660763000	0.982791000
C	-3.652671000	-2.260401000	-0.331600000
C	-4.740634000	-4.453634000	-1.655444000
C	-5.560354000	-3.393997000	-1.283003000
C	-5.011373000	-2.307269000	-0.611415000
H	-3.274162000	-1.412851000	0.219463000
H	-5.157910000	-5.312061000	-2.164970000
H	-6.619545000	-3.421145000	-1.501901000
H	-5.642242000	-1.487485000	-0.294999000
C	-2.807719000	-3.311804000	-0.717706000
C	-3.386255000	-4.417445000	-1.361600000
H	-2.754320000	-5.251220000	-1.640223000
H	-0.980024000	-4.375689000	-0.313588000
C	3.321616000	-2.261387000	-0.035887000
C	4.811646000	-0.279436000	-0.588491000
C	4.594412000	-1.546930000	-0.024001000
C	6.061807000	0.320990000	-0.531435000

C	5.691426000	-2.190254000	0.574218000
C	6.935296000	-1.584174000	0.642876000
C	7.125673000	-0.321258000	0.091641000
H	6.207894000	1.292774000	-0.984263000
H	5.551083000	-3.177958000	0.994005000
H	7.759380000	-2.099441000	1.117953000
H	8.097324000	0.152082000	0.135133000
H	3.402101000	-3.332737000	0.112964000
H	4.014661000	0.226529000	-1.110370000
O	1.242435000	-4.032986000	0.105209000

A21

C	0.476084000	-3.135902000	-0.355871000
C	-0.881111000	-2.526465000	-0.213415000
F	3.815485000	4.179819000	-2.220254000
C	1.883774000	2.808355000	0.507737000
C	2.806504000	3.589970000	-0.177398000
C	2.919318000	3.429309000	-1.546605000
C	2.145352000	2.518865000	-2.245039000
C	1.227776000	1.747138000	-1.544681000
C	1.086568000	1.882663000	-0.162569000
H	1.785208000	2.923465000	1.579453000
H	3.435216000	4.305191000	0.333404000
H	2.266396000	2.420899000	-3.314414000
H	0.618764000	1.023257000	-2.066696000

N	0.147028000	-0.359575000	0.189033000
C	-0.979436000	-1.225106000	0.539869000
C	1.637170000	-2.332000000	0.146516000
C	1.389352000	-0.948443000	0.689676000
C	2.849791000	-2.910817000	0.050100000
C	0.063076000	1.048022000	0.592046000
P	-1.614246000	1.812049000	0.351127000
O	-2.334340000	1.381521000	1.717325000
O	-1.395020000	3.384271000	0.592536000
C	-3.719664000	1.730068000	1.965230000
C	-4.136733000	1.099912000	3.274753000
C	-1.360957000	4.341807000	-0.498616000
C	-2.096481000	5.592912000	-0.071595000
H	-3.799471000	2.817613000	2.006112000
H	-4.325844000	1.365684000	1.135548000
H	-0.313440000	4.552806000	-0.713007000
H	-1.816820000	3.888890000	-1.377619000
H	-1.656379000	6.015325000	0.831712000
H	-2.037237000	6.341128000	-0.863677000
H	-3.148438000	5.381794000	0.120494000
H	-3.515874000	1.461166000	4.094294000
H	-5.175193000	1.354568000	3.491282000
H	-4.052859000	0.014784000	3.226841000
H	-0.994046000	-1.432878000	1.625638000
H	1.366961000	-0.976041000	1.796887000

O	-2.321018000	1.486079000	-0.910357000
H	-1.910932000	-0.730913000	0.305517000
H	2.218648000	-0.300764000	0.410190000
H	0.246700000	1.173346000	1.671843000
C	5.280172000	-3.042781000	-0.201731000
C	5.740164000	-1.072646000	1.688741000
C	6.814209000	-1.661435000	1.031456000
C	6.578242000	-2.652899000	0.084689000
H	5.101399000	-3.821633000	-0.931717000
H	5.916574000	-0.316051000	2.441759000
H	7.826512000	-1.358709000	1.263148000
H	7.407113000	-3.126592000	-0.424096000
C	4.177940000	-2.440087000	0.428572000
C	4.438701000	-1.452926000	1.392649000
H	3.627544000	-1.002073000	1.942130000
H	2.834516000	-3.887891000	-0.420641000
C	-1.914668000	-3.218123000	-0.722042000
C	-4.260883000	-3.943594000	-0.583500000
C	-3.346121000	-2.898978000	-0.782292000
C	-5.628467000	-3.720969000	-0.642906000
C	-3.849439000	-1.628158000	-1.093894000
C	-5.219424000	-1.413231000	-1.171597000
C	-6.114164000	-2.451344000	-0.936420000
H	-6.314585000	-4.540189000	-0.473147000
H	-3.181103000	-0.807133000	-1.311270000

H	-5.585045000	-0.428250000	-1.430233000
H	-7.180216000	-2.276879000	-0.997310000
H	-1.645223000	-4.187565000	-1.129542000
H	-3.886762000	-4.937279000	-0.371884000
O	0.637023000	-4.235604000	-0.863255000

A22

C	0.580236000	-2.933452000	0.418012000
C	1.721254000	-2.106088000	-0.078148000
F	-5.336090000	2.993630000	0.392227000
C	-2.325365000	2.176752000	-1.430286000
C	-3.603518000	2.652505000	-1.164669000
C	-4.094785000	2.538530000	0.122206000
C	-3.352742000	1.966704000	1.140921000
C	-2.075970000	1.502957000	0.857962000
C	-1.544721000	1.601323000	-0.429671000
H	-1.929724000	2.262723000	-2.433775000
H	-4.213310000	3.100558000	-1.936062000
H	-3.774211000	1.890495000	2.133026000
H	-1.485910000	1.055197000	1.643647000
N	0.112316000	-0.259021000	-0.284623000
C	1.415777000	-0.777656000	-0.717371000
C	-0.787611000	-2.514377000	-0.018746000
C	-0.926548000	-1.195249000	-0.729582000
C	-1.801296000	-3.348883000	0.277977000
C	-0.142631000	1.116949000	-0.750325000

P	1.115203000	2.312070000	-0.059295000
O	0.440081000	3.698013000	-0.474946000
O	0.949851000	2.302371000	1.538448000
C	1.163153000	4.944162000	-0.311961000
C	0.448031000	6.011838000	-1.108584000
C	1.829609000	1.524237000	2.392754000
C	1.839536000	2.155916000	3.766712000
H	1.181253000	5.189764000	0.751053000
H	2.187759000	4.803529000	-0.656743000
H	1.447910000	0.503261000	2.427674000
H	2.824614000	1.511677000	1.949944000
H	2.475041000	1.571424000	4.433696000
H	2.229332000	3.172869000	3.724872000
H	0.835881000	2.187275000	4.190334000
H	-0.581667000	6.123707000	-0.770348000
H	0.958282000	6.968021000	-0.983444000
H	0.439845000	5.763363000	-2.169584000
H	1.435506000	-0.868726000	-1.820779000
H	-0.868484000	-1.330722000	-1.826544000
O	2.512277000	2.122980000	-0.522040000
H	2.187978000	-0.061078000	-0.460021000
H	-1.904681000	-0.776250000	-0.517462000
H	0.000134000	1.195370000	-1.841369000
C	-4.111890000	-3.958223000	0.824058000
C	-5.147962000	-2.530326000	-1.311315000

C	-6.007276000	-3.198788000	-0.447014000
C	-5.481964000	-3.917607000	0.621891000
H	-3.706919000	-4.527351000	1.650929000
H	-5.546905000	-1.987489000	-2.157833000
H	-7.075876000	-3.170526000	-0.612249000
H	-6.140850000	-4.452813000	1.292563000
C	-3.228112000	-3.262778000	-0.017907000
C	-3.776365000	-2.559336000	-1.101662000
H	-3.129382000	-2.061393000	-1.807226000
H	-1.507385000	-4.215062000	0.861294000
C	2.951838000	-2.635401000	0.077303000
C	5.373606000	-2.872451000	0.244003000
C	4.281785000	-2.168254000	-0.295624000
C	6.680662000	-2.508960000	-0.034660000
C	4.563617000	-1.091697000	-1.153906000
C	5.873508000	-0.731436000	-1.434855000
C	6.936724000	-1.431608000	-0.876253000
H	7.499229000	-3.066841000	0.400242000
H	3.769477000	-0.530261000	-1.616757000
H	6.062340000	0.103712000	-2.095797000
H	7.955430000	-1.144191000	-1.099777000
H	2.949459000	-3.589418000	0.593344000
H	5.178744000	-3.714379000	0.895726000
O	0.754561000	-3.924967000	1.111239000

B1

C	0.889064000	-3.085567000	-0.217238000
C	2.011932000	-2.095627000	-0.118271000
F	1.544504000	4.736303000	-3.581971000
C	-0.190838000	2.065672000	-1.869897000
C	0.180849000	2.952741000	-2.870680000
C	1.185184000	3.869240000	-2.613096000
C	1.828116000	3.921222000	-1.391298000
C	1.448926000	3.017137000	-0.406264000
C	0.439057000	2.078295000	-0.621531000
H	-0.993933000	1.372385000	-2.064065000
H	-0.302257000	2.946187000	-3.837384000
H	2.606973000	4.650991000	-1.222210000
H	1.951982000	3.047742000	0.551814000
N	0.323345000	-0.336912000	0.222176000
C	1.700104000	-0.620429000	-0.162216000
C	-0.493034000	-2.536756000	-0.379615000
C	-0.617952000	-1.055018000	-0.629984000
C	-1.507983000	-3.412791000	-0.338865000
C	0.101352000	1.084518000	0.488420000
P	-1.536824000	1.388853000	1.304973000
O	-1.340635000	2.833283000	1.990144000
O	-2.509017000	1.701445000	0.068219000
C	-1.424674000	3.027946000	3.424914000
C	-0.062514000	3.352855000	4.001570000

C	-3.888907000	2.078601000	0.299952000
C	-4.611877000	2.083057000	-1.027896000
H	-2.117059000	3.857058000	3.569729000
H	-1.848276000	2.132834000	3.877441000
H	-4.336041000	1.366435000	0.994206000
H	-3.898343000	3.068451000	0.759230000
H	-5.648760000	2.388685000	-0.880323000
H	-4.144316000	2.781693000	-1.721278000
H	-4.608481000	1.089469000	-1.474688000
H	0.373224000	4.221571000	3.508062000
H	-0.158062000	3.575569000	5.065725000
H	0.621349000	2.510194000	3.896498000
H	1.916460000	-0.225105000	-1.170603000
H	-0.419670000	-0.865614000	-1.700227000
O	-2.008002000	0.340636000	2.238577000
H	2.364539000	-0.092688000	0.524379000
H	-1.629069000	-0.716987000	-0.434704000
H	0.774749000	1.322208000	1.317769000
C	-3.740322000	-3.993072000	-1.227258000
C	-4.977693000	-2.053936000	0.316606000
C	-5.740184000	-2.850640000	-0.530286000
C	-5.115244000	-3.823757000	-1.302626000
H	-3.259970000	-4.763293000	-1.817269000
H	-5.460102000	-1.318185000	0.946865000
H	-6.814072000	-2.726857000	-0.575300000

H	-5.700360000	-4.459395000	-1.954041000
C	-2.956477000	-3.172799000	-0.404897000
C	-3.597918000	-2.206076000	0.380110000
H	-3.025982000	-1.599309000	1.069710000
H	-1.208378000	-4.453356000	-0.260572000
C	3.247331000	-2.610021000	0.034885000
C	4.859658000	-0.676472000	-0.297586000
C	4.548917000	-1.963965000	0.169376000
C	6.131088000	-0.145330000	-0.130202000
C	5.576025000	-2.698710000	0.786199000
C	6.840544000	-2.161928000	0.965733000
C	7.123605000	-0.878555000	0.509742000
H	6.349242000	0.844373000	-0.509100000
H	5.363961000	-3.701952000	1.132602000
H	7.608770000	-2.746831000	1.453659000
H	8.112041000	-0.459054000	0.640595000
H	3.270014000	-3.692240000	0.103387000
H	4.120985000	-0.097606000	-0.829277000
O	1.096459000	-4.287554000	-0.156881000

B2

C	0.899763000	-2.702239000	-0.483049000
C	2.034287000	-1.724944000	-0.459716000
F	-0.036904000	5.357227000	-3.272365000
C	-1.044233000	2.572822000	-1.197169000

C	-1.086631000	3.557595000	-2.175553000
C	0.001643000	4.398003000	-2.324957000
C	1.123893000	4.286451000	-1.526212000
C	1.149016000	3.292233000	-0.556533000
C	0.076048000	2.417057000	-0.375577000
H	-1.905377000	1.937541000	-1.059890000
H	-1.951441000	3.684444000	-2.811122000
H	1.951571000	4.967869000	-1.661423000
H	2.020595000	3.205296000	0.079449000
N	0.393164000	-0.055094000	0.197315000
C	1.698319000	-0.256888000	-0.421552000
C	-0.474294000	-2.145208000	-0.692616000
C	-0.657548000	-0.649666000	-0.619105000
C	-1.454341000	-3.039114000	-0.921299000
C	0.189324000	1.315219000	0.675621000
P	-1.059040000	1.475725000	2.037410000
O	-2.474819000	1.279788000	1.305225000
O	-0.962340000	0.137294000	2.917996000
C	-3.699401000	1.451136000	2.067483000
C	-4.861019000	1.503158000	1.101507000
C	-0.269397000	0.088561000	4.188897000
C	1.056748000	-0.631807000	4.057322000
H	-3.792214000	0.608727000	2.754453000
H	-3.620062000	2.370598000	2.647585000
H	-0.145865000	1.104762000	4.562007000

H	-0.937043000	-0.449039000	4.861664000
H	1.750517000	-0.077214000	3.425375000
H	1.512745000	-0.740811000	5.043126000
H	0.919778000	-1.623152000	3.627415000
H	-4.922403000	0.587051000	0.514703000
H	-5.793280000	1.617844000	1.656555000
H	-4.762586000	2.349704000	0.422085000
H	1.733027000	0.179147000	-1.435913000
H	-0.663250000	-0.217009000	-1.635154000
O	-0.885460000	2.728155000	2.806221000
H	2.450006000	0.268296000	0.168226000
H	-1.623303000	-0.427385000	-0.171781000
H	1.088842000	1.546643000	1.253096000
C	-3.439943000	-1.692320000	-1.745324000
C	-5.099315000	-3.843329000	-1.157390000
C	-5.641257000	-2.677023000	-1.687460000
C	-4.803960000	-1.608537000	-1.988394000
H	-2.808996000	-0.861808000	-2.020648000
H	-5.739259000	-4.685938000	-0.931967000
H	-6.703770000	-2.606661000	-1.877686000
H	-5.213762000	-0.707324000	-2.425007000
C	-2.875679000	-2.854862000	-1.197007000
C	-3.735686000	-3.933051000	-0.928512000
H	-3.317096000	-4.847221000	-0.527799000
H	-1.134112000	-4.073828000	-0.863933000

C	3.277628000	-2.239978000	-0.442498000
C	5.669571000	-2.302723000	0.088950000
C	4.581135000	-1.583194000	-0.432049000
C	6.938154000	-1.748703000	0.148483000
C	4.829391000	-0.294904000	-0.931734000
C	6.103392000	0.253903000	-0.884263000
C	7.160237000	-0.463742000	-0.335873000
H	7.756339000	-2.321278000	0.564439000
H	4.034890000	0.268457000	-1.396315000
H	6.273200000	1.244290000	-1.285430000
H	8.150836000	-0.030878000	-0.299002000
H	3.311062000	-3.323054000	-0.393858000
H	5.503410000	-3.307035000	0.456813000
O	1.093558000	-3.903953000	-0.379150000

B3

C	0.820930000	-2.845680000	-0.285746000
C	1.937258000	-1.843900000	-0.366069000
F	-1.000964000	4.362934000	-4.339861000
C	-1.580850000	2.227662000	-1.480212000
C	-1.822384000	2.966733000	-2.630563000
C	-0.770963000	3.642894000	-3.223202000
C	0.507783000	3.609312000	-2.699022000
C	0.730266000	2.864762000	-1.547020000
C	-0.300681000	2.158700000	-0.922405000

H	-2.404315000	1.715830000	-1.002283000
H	-2.810711000	3.027392000	-3.063296000
H	1.303018000	4.157252000	-3.183939000
H	1.727726000	2.839241000	-1.126216000
N	0.272830000	-0.111655000	0.100290000
C	1.594309000	-0.378069000	-0.456792000
C	-0.575689000	-2.330382000	-0.427743000
C	-0.746003000	-0.849396000	-0.650600000
C	-1.558892000	-3.249070000	-0.421661000
C	0.010452000	1.322143000	0.312332000
P	-1.227210000	1.493266000	1.679195000
O	-0.411844000	1.021824000	2.975170000
O	-1.413979000	3.067550000	1.897212000
C	-0.775937000	-0.144435000	3.762076000
C	0.419508000	-1.061143000	3.893427000
C	-0.396076000	3.959680000	2.400390000
C	-1.049492000	5.286210000	2.719815000
H	-1.101400000	0.228495000	4.733431000
H	-1.618606000	-0.640622000	3.284433000
H	0.058714000	3.516136000	3.286260000
H	0.375000000	4.083713000	1.636126000
H	-0.300819000	5.987522000	3.091359000
H	-1.511418000	5.712764000	1.830163000
H	-1.817545000	5.163025000	3.482581000
H	1.258888000	-0.543145000	4.357854000

H	0.157975000	-1.917069000	4.517956000
H	0.731633000	-1.429115000	2.917420000
H	1.653234000	-0.039206000	-1.506661000
H	-0.667090000	-0.636111000	-1.730719000
O	-2.522629000	0.814847000	1.447706000
H	2.333219000	0.196857000	0.103247000
H	-1.723775000	-0.517726000	-0.332512000
H	0.927152000	1.720101000	0.753573000
C	-3.692502000	-4.168076000	-1.197441000
C	-5.155408000	-2.051558000	-0.178935000
C	-5.807173000	-3.076377000	-0.856221000
C	-5.068582000	-4.139325000	-1.364058000
H	-3.123047000	-5.005325000	-1.580544000
H	-5.722700000	-1.232802000	0.243282000
H	-6.882391000	-3.054172000	-0.974240000
H	-5.565274000	-4.950777000	-1.879302000
C	-3.013906000	-3.123531000	-0.549795000
C	-3.775530000	-2.067791000	-0.026759000
H	-3.310169000	-1.270983000	0.534505000
H	-1.202613000	-4.271942000	-0.351916000
C	3.189166000	-2.336263000	-0.313745000
C	4.717633000	-0.421612000	-0.973884000
C	4.483882000	-1.663364000	-0.361976000
C	5.984777000	0.145030000	-0.975085000
C	5.580627000	-2.319914000	0.221255000

C	6.841944000	-1.746732000	0.232788000
C	7.049347000	-0.507257000	-0.363809000
H	6.143478000	1.097099000	-1.464124000
H	5.426720000	-3.289752000	0.676444000
H	7.666278000	-2.269773000	0.698845000
H	8.034508000	-0.060628000	-0.364851000
H	3.238481000	-3.411108000	-0.176687000
H	3.917184000	0.087126000	-1.488412000
O	1.054551000	-4.033174000	-0.116214000

B4

C	0.613400000	-2.933723000	-0.044013000
C	1.796197000	-2.020264000	-0.161205000
F	0.346647000	4.421134000	-4.319770000
C	-0.986647000	2.258331000	-1.745593000
C	-0.911832000	3.022480000	-2.903190000
C	0.270845000	3.676403000	-3.197597000
C	1.375456000	3.597013000	-2.370017000
C	1.283665000	2.825827000	-1.218132000
C	0.112504000	2.140129000	-0.888929000
H	-1.920506000	1.782884000	-1.485542000
H	-1.758926000	3.123235000	-3.566658000
H	2.279414000	4.131216000	-2.625543000
H	2.141868000	2.763491000	-0.560761000
N	0.236283000	-0.184992000	0.163809000

C	1.533477000	-0.555772000	-0.392544000
C	-0.727201000	-2.358757000	-0.373861000
C	-0.843726000	-0.865023000	-0.548580000
C	-1.741163000	-3.239673000	-0.484899000
C	0.090270000	1.264542000	0.360548000
P	-1.356041000	1.635176000	1.457324000
O	-1.289510000	0.615700000	2.688768000
O	-0.882068000	3.063122000	2.028204000
C	-0.116995000	0.402136000	3.506150000
C	-0.527145000	-0.413419000	4.712547000
C	-1.847388000	4.007469000	2.558882000
C	-1.240832000	5.391404000	2.500794000
H	0.623306000	-0.130903000	2.908533000
H	0.293373000	1.369013000	3.804560000
H	-2.760636000	3.940560000	1.968882000
H	-2.078188000	3.719676000	3.586279000
H	-1.941495000	6.117988000	2.915033000
H	-0.317656000	5.437940000	3.078505000
H	-1.020125000	5.673906000	1.471915000
H	-0.944350000	-1.370836000	4.403101000
H	0.342660000	-0.603493000	5.343141000
H	-1.274694000	0.114951000	5.303683000
H	1.591747000	-0.310680000	-1.467254000
H	-0.833513000	-0.605688000	-1.621607000
O	-2.697851000	1.610637000	0.835775000

H	2.306737000	0.032824000	0.102215000
H	-1.797985000	-0.524879000	-0.150131000
H	0.949258000	1.563197000	0.966311000
C	-3.703415000	-1.940533000	-1.445624000
C	-5.376977000	-4.049819000	-0.751231000
C	-5.906157000	-2.918851000	-1.363959000
C	-5.062328000	-1.871346000	-1.715153000
H	-3.072726000	-1.122711000	-1.753828000
H	-6.022722000	-4.875152000	-0.482547000
H	-6.965594000	-2.857743000	-1.573767000
H	-5.463961000	-0.993783000	-2.203639000
C	-3.150745000	-3.066173000	-0.813579000
C	-4.018763000	-4.123788000	-0.490149000
H	-3.609759000	-5.008368000	-0.019265000
H	-1.456916000	-4.262956000	-0.264871000
C	3.012140000	-2.578720000	-0.019844000
C	4.662856000	-0.836509000	-0.835706000
C	4.344436000	-1.985860000	-0.095362000
C	5.958647000	-0.339174000	-0.857315000
C	5.386265000	-2.627568000	0.594713000
C	6.676512000	-2.122435000	0.583970000
C	6.968295000	-0.971472000	-0.140929000
H	6.182755000	0.540886000	-1.445512000
H	5.166681000	-3.529787000	1.150854000
H	7.457777000	-2.630898000	1.133024000

H	7.976389000	-0.579780000	-0.159439000
H	2.992670000	-3.635778000	0.222752000
H	3.906509000	-0.350112000	-1.432472000
O	0.751117000	-4.107243000	0.267791000

B5

C	0.914786000	-2.680279000	-0.607972000
C	2.066676000	-1.732991000	-0.468783000
F	0.600101000	5.631207000	-2.796187000
C	1.364184000	3.334909000	-0.114609000
C	1.482885000	4.408451000	-0.988129000
C	0.496613000	4.596045000	-1.937824000
C	-0.598261000	3.755546000	-2.028730000
C	-0.703045000	2.692894000	-1.141021000
C	0.279963000	2.457102000	-0.175092000
H	2.129370000	3.184892000	0.636189000
H	2.318875000	5.091245000	-0.937118000
H	-1.355909000	3.943056000	-2.776240000
H	-1.575236000	2.059941000	-1.188393000
N	0.431170000	-0.070017000	0.223262000
C	1.762191000	-0.262719000	-0.341106000
C	-0.439477000	-2.080653000	-0.828227000
C	-0.589968000	-0.587046000	-0.679690000
C	-1.433518000	-2.937262000	-1.128312000
C	0.233490000	1.270868000	0.784233000

P	-1.156234000	1.371436000	2.010006000
O	-2.487637000	1.473374000	1.122505000
O	-1.332476000	-0.095755000	2.638124000
C	-3.755743000	1.774819000	1.764849000
C	-4.781427000	2.047350000	0.688421000
C	-0.447696000	-0.576662000	3.677888000
C	-1.125220000	-1.729885000	4.384130000
H	-4.041475000	0.917156000	2.375319000
H	-3.616091000	2.637682000	2.415573000
H	0.481882000	-0.900774000	3.206221000
H	-0.233223000	0.242155000	4.365373000
H	-0.466913000	-2.125487000	5.159055000
H	-2.053106000	-1.404708000	4.854258000
H	-1.352168000	-2.532608000	3.683263000
H	-4.905658000	1.180004000	0.040459000
H	-5.744580000	2.270902000	1.149594000
H	-4.487978000	2.902494000	0.079755000
H	1.860693000	0.245000000	-1.316846000
H	-0.530883000	-0.100039000	-1.668864000
O	-0.937944000	2.451712000	2.996232000
H	2.492839000	0.199033000	0.323715000
H	-1.572727000	-0.363546000	-0.271196000
H	1.073460000	1.412549000	1.469994000
C	-3.359949000	-1.503156000	-1.942679000
C	-5.093393000	-3.625457000	-1.477486000

C	-5.589179000	-2.423623000	-1.972133000
C	-4.714933000	-1.369459000	-2.212145000
H	-2.698452000	-0.682033000	-2.170717000
H	-5.762382000	-4.456808000	-1.299688000
H	-6.644623000	-2.314386000	-2.182273000
H	-5.088506000	-0.440254000	-2.621780000
C	-2.842365000	-2.702316000	-1.428732000
C	-3.738478000	-3.764605000	-1.222865000
H	-3.355664000	-4.705854000	-0.850011000
H	-1.139503000	-3.981276000	-1.116178000
C	3.297099000	-2.277892000	-0.434628000
C	4.911647000	-0.342606000	-0.717971000
C	4.613415000	-1.657187000	-0.325238000
C	6.195808000	0.166772000	-0.584220000
C	5.663569000	-2.441629000	0.180653000
C	6.941840000	-1.927511000	0.327078000
C	7.213495000	-0.617214000	-0.052878000
H	6.404218000	1.178906000	-0.904713000
H	5.459801000	-3.465347000	0.467055000
H	7.729379000	-2.550695000	0.729244000
H	8.212231000	-0.215257000	0.051731000
H	3.304649000	-3.362243000	-0.460194000
H	4.149834000	0.275451000	-1.167438000
O	1.080834000	-3.890461000	-0.582245000

B6

C	1.095907000	-3.071638000	-0.147910000
C	2.163030000	-2.021825000	-0.048301000
F	1.328041000	4.701484000	-3.680263000
C	-0.266237000	1.980095000	-1.912016000
C	0.058007000	2.865015000	-2.931025000
C	1.014911000	3.836215000	-2.694015000
C	1.656679000	3.944578000	-1.475264000
C	1.325579000	3.041870000	-0.471862000
C	0.364143000	2.049173000	-0.665888000
H	-1.033986000	1.243701000	-2.089895000
H	-0.426099000	2.815114000	-3.895947000
H	2.397694000	4.716235000	-1.322798000
H	1.829407000	3.116053000	0.483617000
N	0.379706000	-0.348157000	0.233783000
C	1.774329000	-0.566160000	-0.127501000
C	-0.310714000	-2.599547000	-0.338332000
C	-0.511351000	-1.131093000	-0.614849000
C	-1.279863000	-3.525638000	-0.296256000
C	0.076144000	1.063417000	0.464704000
P	-1.579295000	1.299280000	1.269391000
O	-1.485014000	2.774249000	1.912333000
O	-2.569448000	1.545582000	0.035753000
C	-1.160169000	2.962738000	3.311491000
C	-1.709177000	4.300901000	3.753994000

C	-3.972245000	1.834433000	0.266880000
C	-4.688755000	1.808326000	-1.063930000
H	-1.584364000	2.140270000	3.885832000
H	-0.073082000	2.934513000	3.418750000
H	-4.374538000	1.087830000	0.952028000
H	-4.043403000	2.815966000	0.737136000
H	-5.743199000	2.046815000	-0.916951000
H	-4.263670000	2.542951000	-1.747408000
H	-4.622192000	0.822265000	-1.522421000
H	-2.795150000	4.322577000	3.665333000
H	-1.446768000	4.479779000	4.797786000
H	-1.296736000	5.110032000	3.151652000
H	1.983948000	-0.180750000	-1.141256000
H	-0.312103000	-0.948552000	-1.686098000
O	-1.982449000	0.247784000	2.229446000
H	2.400278000	0.009881000	0.556422000
H	-1.541556000	-0.845257000	-0.435185000
H	0.732285000	1.357122000	1.291646000
C	-3.471756000	-4.208752000	-1.217447000
C	-4.817742000	-2.334446000	0.315949000
C	-5.531596000	-3.163237000	-0.542345000
C	-4.852097000	-4.103122000	-1.309761000
H	-2.948956000	-4.953770000	-1.803704000
H	-5.341593000	-1.623824000	0.941935000
H	-6.609443000	-3.089667000	-0.600538000

H	-5.398598000	-4.762791000	-1.970762000
C	-2.737639000	-3.355663000	-0.383017000
C	-3.433187000	-2.421845000	0.395668000
H	-2.898219000	-1.789023000	1.091644000
H	-0.930424000	-4.548678000	-0.196745000
C	3.420624000	-2.466254000	0.138607000
C	4.934623000	-0.456828000	-0.205205000
C	4.682966000	-1.748658000	0.284686000
C	6.172262000	0.144928000	-0.023971000
C	5.734031000	-2.414029000	0.938663000
C	6.964142000	-1.806722000	1.131850000
C	7.188263000	-0.520240000	0.652497000
H	6.346157000	1.136357000	-0.420879000
H	5.567797000	-3.419483000	1.303239000
H	7.751645000	-2.338969000	1.648454000
H	8.150030000	-0.045669000	0.793595000
H	3.499575000	-3.544220000	0.229369000
H	4.177691000	0.069589000	-0.765066000
O	1.365412000	-4.259842000	-0.065572000

B7

C	-1.226837000	-2.783546000	-0.128178000
C	-2.292666000	-1.753559000	0.082772000
F	1.811268000	3.742033000	4.466438000
C	-0.475116000	2.659435000	1.887357000

C	-0.008686000	3.245768000	3.057748000
C	1.343156000	3.173970000	3.336047000
C	2.231524000	2.543789000	2.483182000
C	1.745486000	1.963352000	1.319267000
C	0.385544000	2.005844000	1.002727000
H	-1.530884000	2.723743000	1.657066000
H	-0.672932000	3.755409000	3.740967000
H	3.283893000	2.521093000	2.728095000
H	2.440937000	1.489662000	0.641560000
N	-0.533329000	-0.084342000	-0.138668000
C	-1.863946000	-0.342937000	0.397987000
C	0.177793000	-2.387067000	0.206716000
C	0.457139000	-0.936834000	0.507856000
C	1.096629000	-3.370751000	0.204911000
C	-0.196340000	1.344788000	-0.239754000
P	0.665070000	1.652387000	-1.847286000
O	1.136509000	3.182984000	-1.678426000
O	2.009730000	0.770898000	-1.747491000
C	1.102670000	4.130389000	-2.773088000
C	0.696809000	5.484699000	-2.233841000
C	2.723536000	0.391392000	-2.954633000
C	3.671405000	-0.737443000	-2.619892000
H	2.102652000	4.169100000	-3.209632000
H	0.406857000	3.770504000	-3.529778000
H	1.994222000	0.097906000	-3.708172000

H	3.268225000	1.265346000	-3.318684000
H	4.228938000	-1.022384000	-3.513555000
H	4.385295000	-0.438427000	-1.852722000
H	3.126846000	-1.610779000	-2.263688000
H	1.382208000	5.815047000	-1.453609000
H	0.710128000	6.221446000	-3.038801000
H	-0.309795000	5.450737000	-1.817324000
H	-1.897082000	-0.161445000	1.488327000
H	0.470522000	-0.767180000	1.598825000
O	-0.146857000	1.407092000	-3.059178000
H	-2.566338000	0.351382000	-0.062588000
H	1.443015000	-0.677421000	0.132560000
H	-1.140374000	1.850964000	-0.452799000
C	3.146265000	-2.397022000	1.338751000
C	4.687591000	-4.419477000	0.215677000
C	5.288983000	-3.458683000	1.022187000
C	4.510258000	-2.456025000	1.588712000
H	2.561000000	-1.629031000	1.819864000
H	5.281161000	-5.212726000	-0.218864000
H	6.351864000	-3.499625000	1.218980000
H	4.965700000	-1.719877000	2.237864000
C	2.523103000	-3.347596000	0.514482000
C	3.323372000	-4.370147000	-0.022467000
H	2.857829000	-5.127527000	-0.639932000
H	0.713773000	-4.336954000	-0.105618000

C	-3.566327000	-2.163802000	-0.059283000
C	-4.999029000	-0.306412000	0.901066000
C	-4.823772000	-1.437949000	0.089347000
C	-6.233239000	0.322060000	0.990921000
C	-5.944753000	-1.924987000	-0.603097000
C	-7.172936000	-1.288426000	-0.524407000
C	-7.322091000	-0.157929000	0.272291000
H	-6.347371000	1.186215000	1.631963000
H	-5.835767000	-2.810225000	-1.216232000
H	-8.016947000	-1.677871000	-1.077819000
H	-8.281562000	0.336527000	0.342739000
H	-3.669324000	-3.198041000	-0.369532000
H	-4.180010000	0.062158000	1.499294000
O	-1.497378000	-3.911074000	-0.511770000

B8

C	0.899830000	-2.702286000	-0.483005000
C	2.034319000	-1.724947000	-0.459715000
F	-0.037682000	5.356715000	-3.273104000
C	-1.044615000	2.572644000	-1.197271000
C	-1.087218000	3.557273000	-2.175792000
C	0.001050000	4.397614000	-2.325579000
C	1.123488000	4.286139000	-1.527083000
C	1.148816000	3.292071000	-0.557259000
C	0.075856000	2.416964000	-0.375917000

H	-1.905737000	1.937399000	-1.059690000
H	-1.952176000	3.684044000	-2.811175000
H	1.951147000	4.967521000	-1.662603000
H	2.020543000	3.205198000	0.078528000
N	0.393150000	-0.055113000	0.197272000
C	1.698298000	-0.256905000	-0.421602000
C	-0.474250000	-2.145309000	-0.692521000
C	-0.657573000	-0.649772000	-0.619090000
C	-1.454283000	-3.039269000	-0.921075000
C	0.189295000	1.315288000	0.675404000
P	-1.058907000	1.475908000	2.037319000
O	-2.474767000	1.279470000	1.305411000
O	-0.961698000	0.137766000	2.918244000
C	-3.699241000	1.451634000	2.067688000
C	-4.860996000	1.502628000	1.101822000
C	-0.268857000	0.089613000	4.189229000
C	1.057475000	-0.630476000	4.057993000
H	-3.791965000	0.609954000	2.755566000
H	-3.619776000	2.371695000	2.646814000
H	-0.936430000	-0.447934000	4.862108000
H	-0.145594000	1.105966000	4.562010000
H	0.920777000	-1.621995000	3.628398000
H	1.751155000	-0.075914000	3.425921000
H	1.513422000	-0.739052000	5.043866000
H	-4.922401000	0.585945000	0.515923000

H	-5.793191000	1.617814000	1.656876000
H	-4.762704000	2.348510000	0.421552000
H	1.733005000	0.179093000	-1.435982000
H	-1.623314000	-0.427526000	-0.171717000
O	-0.885528000	2.728599000	2.805741000
H	2.449974000	0.268324000	0.168153000
H	-0.663340000	-0.217162000	-1.635155000
H	1.088867000	1.546796000	1.252766000
C	-3.735560000	-3.933357000	-0.928132000
C	-4.804067000	-1.608953000	-1.988007000
C	-5.641270000	-2.677490000	-1.686988000
C	-5.099210000	-3.843743000	-1.156925000
H	-3.316878000	-4.847485000	-0.527419000
H	-5.213965000	-0.707781000	-2.424616000
H	-6.703801000	-2.607204000	-1.877144000
H	-5.739075000	-4.686395000	-0.931438000
C	-2.875643000	-2.855114000	-1.196711000
C	-3.440029000	-1.692629000	-1.745026000
H	-2.809168000	-0.862075000	-2.020416000
H	-1.134003000	-4.073964000	-0.863660000
C	3.277679000	-2.239936000	-0.442491000
C	4.829389000	-0.294815000	-0.931731000
C	4.581170000	-1.583126000	-0.432080000
C	6.103380000	0.254017000	-0.884274000
C	5.669638000	-2.302653000	0.088861000

C	6.938209000	-1.748611000	0.148380000
C	7.160253000	-0.463627000	-0.335935000
H	6.273161000	1.244419000	-1.285412000
H	5.503501000	-3.306982000	0.456691000
H	7.756419000	-2.321180000	0.564296000
H	8.150846000	-0.030747000	-0.299075000
H	3.311140000	-3.323009000	-0.393819000
H	4.034864000	0.268545000	-1.396270000
O	1.093667000	-3.903993000	-0.379104000

B9

C	0.613399000	-2.933719000	-0.044010000
C	1.796199000	-2.020263000	-0.161183000
F	0.346816000	4.421185000	-4.319706000
C	1.283709000	2.825859000	-1.218040000
C	1.375543000	3.597059000	-2.369913000
C	0.270970000	3.676443000	-3.197544000
C	-0.911712000	3.022498000	-2.903200000
C	-0.986572000	2.258337000	-1.745614000
C	0.112542000	2.140141000	-0.888900000
H	2.141881000	2.763525000	-0.560630000
H	2.279505000	4.131278000	-2.625390000
H	-1.758776000	3.123248000	-3.566707000
H	-1.920436000	1.782877000	-1.485612000
N	0.236277000	-0.184994000	0.163814000

C	1.533488000	-0.555766000	-0.392501000
C	-0.727196000	-2.358753000	-0.373883000
C	-0.843709000	-0.865020000	-0.548617000
C	-1.741161000	-3.239665000	-0.484926000
C	0.090261000	1.264539000	0.360565000
P	-1.356087000	1.635171000	1.457296000
O	-1.289611000	0.615674000	2.688727000
O	-0.882118000	3.063103000	2.028217000
C	-0.117134000	0.402090000	3.506157000
C	-0.527335000	-0.413521000	4.712499000
C	-1.847446000	4.007461000	2.558860000
C	-1.240864000	5.391385000	2.500813000
H	0.623204000	-0.130916000	2.908555000
H	0.293208000	1.368959000	3.804628000
H	-2.760669000	3.940573000	1.968818000
H	-2.078300000	3.719663000	3.586244000
H	-1.941533000	6.117978000	2.915026000
H	-0.317712000	5.437899000	3.078566000
H	-1.020105000	5.673892000	1.471946000
H	-0.944512000	-1.370931000	4.402989000
H	0.342439000	-0.603612000	5.343130000
H	-1.274922000	0.114814000	5.303617000
H	1.591799000	-0.310650000	-1.467203000
H	-0.833452000	-0.605690000	-1.621644000
O	-2.697875000	1.610658000	0.835700000

H	2.306732000	0.032817000	0.102299000
H	-1.797982000	-0.524869000	-0.150208000
H	0.949230000	1.563182000	0.966360000
C	-3.703381000	-1.940533000	-1.445725000
C	-5.376984000	-4.049767000	-0.751273000
C	-5.906139000	-2.918814000	-1.364052000
C	-5.062289000	-1.871335000	-1.715274000
H	-3.072674000	-1.122735000	-1.753954000
H	-6.022745000	-4.875080000	-0.482568000
H	-6.965572000	-2.857698000	-1.573875000
H	-5.463903000	-0.993786000	-2.203800000
C	-3.150736000	-3.066157000	-0.813629000
C	-4.018774000	-4.123747000	-0.490172000
H	-3.609790000	-5.008316000	-0.019249000
H	-1.456924000	-4.262946000	-0.264880000
C	3.012139000	-2.578727000	-0.019828000
C	4.662864000	-0.836491000	-0.835622000
C	4.344438000	-1.985873000	-0.095329000
C	5.958658000	-0.339165000	-0.857214000
C	5.386265000	-2.627618000	0.594714000
C	6.676516000	-2.122495000	0.583990000
C	6.968305000	-0.971501000	-0.140859000
H	6.182770000	0.540921000	-1.445372000
H	5.166677000	-3.529861000	1.150817000
H	7.457779000	-2.630987000	1.133019000

H	7.976402000	-0.579815000	-0.159356000
H	2.992663000	-3.635790000	0.222747000
H	3.906519000	-0.350060000	-1.432364000
O	0.751108000	-4.107241000	0.267796000

B10

C	1.079729000	-2.195401000	-0.501055000
C	2.145238000	-1.163938000	-0.744435000
F	-2.910057000	5.999399000	-1.446017000
C	-0.967569000	3.750391000	0.618765000
C	-1.710553000	4.857311000	0.231905000
C	-2.184042000	4.925361000	-1.065989000
C	-1.928910000	3.926469000	-1.984259000
C	-1.179507000	2.825504000	-1.582888000
C	-0.695811000	2.714220000	-0.281118000
H	-0.603608000	3.687979000	1.634530000
H	-1.921681000	5.660528000	0.923529000
H	-2.305340000	4.014362000	-2.993597000
H	-0.953478000	2.052173000	-2.300838000
N	0.361259000	0.541208000	-0.923041000
C	1.690801000	0.152669000	-1.339230000
C	-0.331796000	-1.847490000	-0.874658000
C	-0.652517000	-0.424512000	-1.295652000
C	-1.226592000	-2.851662000	-0.793922000
C	0.170337000	1.512496000	0.130171000

P	-0.330870000	0.850704000	1.808588000
O	-1.834481000	0.367221000	1.547181000
O	0.462541000	-0.534668000	1.990367000
C	-2.649665000	-0.132824000	2.638441000
C	-4.080913000	-0.222917000	2.159787000
C	1.568850000	-0.692031000	2.917562000
C	1.472967000	-2.058408000	3.559952000
H	-2.266125000	-1.111921000	2.929819000
H	-2.552975000	0.548288000	3.484299000
H	2.490686000	-0.599618000	2.341992000
H	1.525465000	0.111196000	3.651197000
H	1.476785000	-2.840589000	2.801956000
H	2.327403000	-2.211483000	4.221635000
H	0.562588000	-2.146721000	4.153270000
H	-4.166696000	-0.900026000	1.310596000
H	-4.713193000	-0.599947000	2.965170000
H	-4.450982000	0.757907000	1.862644000
H	1.684547000	0.043196000	-2.434332000
H	-0.772147000	-0.395566000	-2.389850000
O	-0.128740000	1.798541000	2.932177000
H	2.388111000	0.956398000	-1.129415000
H	-1.606505000	-0.123868000	-0.869365000
H	1.162698000	1.908764000	0.365237000
C	-3.362091000	-1.977843000	-1.865881000
C	-4.741498000	-4.119803000	-0.752442000

C	-5.423406000	-3.183511000	-1.522770000
C	-4.725336000	-2.119771000	-2.083098000
H	-2.846239000	-1.157166000	-2.336889000
H	-5.271567000	-4.958536000	-0.321291000
H	-6.486352000	-3.287779000	-1.693944000
H	-5.244484000	-1.397088000	-2.698454000
C	-2.656160000	-2.903846000	-1.079618000
C	-3.378383000	-3.985246000	-0.544604000
H	-2.850318000	-4.722514000	0.046224000
H	-0.812833000	-3.782634000	-0.421873000
C	3.414529000	-1.531902000	-0.506010000
C	5.782690000	-1.441191000	-1.182988000
C	4.666201000	-0.776483000	-0.656245000
C	6.997351000	-0.790239000	-1.341608000
C	4.828745000	0.553509000	-0.247503000
C	6.048612000	1.200350000	-0.389828000
C	7.134935000	0.536123000	-0.947943000
H	7.840145000	-1.321660000	-1.763134000
H	4.008322000	1.076959000	0.221992000
H	6.153034000	2.223918000	-0.055273000
H	8.083578000	1.043214000	-1.061021000
H	3.536403000	-2.564456000	-0.193619000
H	5.685952000	-2.478147000	-1.478147000
O	1.361444000	-3.286231000	-0.027661000

B11

C	-0.008678000	2.817962000	0.486366000
C	-1.263982000	2.138957000	0.032740000
F	5.001532000	-3.793671000	0.760085000
C	2.189230000	-2.696433000	-1.225183000
C	3.378165000	-3.325463000	-0.879861000
C	3.843997000	-3.192192000	0.414834000
C	3.160998000	-2.455750000	1.366819000
C	1.972383000	-1.836933000	1.004128000
C	1.474358000	-1.944468000	-0.295534000
H	1.809647000	-2.803693000	-2.232289000
H	3.936562000	-3.911900000	-1.595135000
H	3.555137000	-2.378266000	2.370019000
H	1.429598000	-1.257582000	1.736673000
N	0.071581000	0.103368000	-0.232572000
C	-1.153462000	0.794719000	-0.640319000
C	1.283003000	2.217823000	0.031721000
C	1.213078000	0.902449000	-0.700684000
C	2.390706000	2.953223000	0.236666000
C	0.156624000	-1.292757000	-0.689973000
P	-1.156583000	-2.474749000	-0.077188000
O	-1.288815000	-2.301636000	1.509606000
O	-2.534796000	-1.917448000	-0.706790000
C	-1.915639000	-1.201712000	2.211233000
C	-2.161811000	-1.636712000	3.639103000

C	-3.643704000	-2.832322000	-0.940038000
C	-3.675768000	-3.278377000	-2.385998000
H	-1.252270000	-0.338578000	2.167308000
H	-2.850762000	-0.946312000	1.711529000
H	-3.553794000	-3.688068000	-0.272138000
H	-4.537489000	-2.270197000	-0.672541000
H	-4.547455000	-3.913280000	-2.555681000
H	-3.741921000	-2.421047000	-3.056394000
H	-2.781135000	-3.850027000	-2.626817000
H	-1.226021000	-1.902762000	4.129622000
H	-2.623499000	-0.820749000	4.197145000
H	-2.825589000	-2.500154000	3.674211000
H	-1.172230000	0.908901000	-1.741039000
H	1.123856000	1.089066000	-1.787586000
O	-0.850787000	-3.870318000	-0.444849000
H	-2.018376000	0.194098000	-0.384354000
H	2.122155000	0.336880000	-0.563350000
H	0.061331000	-1.360264000	-1.786425000
C	4.570418000	3.835294000	-0.456431000
C	5.775488000	1.343621000	-0.392796000
C	6.516482000	2.457665000	-0.771471000
C	5.908001000	3.707391000	-0.798668000
H	4.105327000	4.812759000	-0.464184000
H	6.246545000	0.371116000	-0.341766000
H	7.562100000	2.354274000	-1.028419000

H	6.478167000	4.584026000	-1.075797000
C	3.796066000	2.717510000	-0.108886000
C	4.432133000	1.468809000	-0.068228000
H	3.893645000	0.592586000	0.259442000
H	2.198595000	3.917140000	0.696850000
C	-2.417720000	2.787092000	0.283463000
C	-4.200130000	1.592572000	-1.072532000
C	-3.801577000	2.448770000	-0.033700000
C	-5.544058000	1.338104000	-1.308791000
C	-4.809485000	3.053409000	0.736850000
C	-6.150064000	2.787252000	0.509404000
C	-6.523971000	1.924601000	-0.515756000
H	-5.827693000	0.686037000	-2.124297000
H	-4.523743000	3.735615000	1.527104000
H	-6.904462000	3.259717000	1.124138000
H	-7.569824000	1.722344000	-0.703806000
H	-2.293751000	3.709672000	0.840390000
H	-3.463781000	1.148305000	-1.723368000
O	-0.040785000	3.828954000	1.171427000

B12

C	1.061034000	-2.488717000	-0.785514000
C	2.170613000	-1.496075000	-0.629262000
F	-0.826254000	5.584664000	-2.844596000
C	0.904634000	3.412943000	-0.535076000

C	0.675559000	4.448941000	-1.431677000
C	-0.588893000	4.583100000	-1.973220000
C	-1.620858000	3.724486000	-1.640112000
C	-1.371280000	2.695931000	-0.741265000
C	-0.105029000	2.516942000	-0.178818000
H	1.888036000	3.311894000	-0.094186000
H	1.454259000	5.147305000	-1.702942000
H	-2.600341000	3.872394000	-2.072086000
H	-2.181565000	2.039449000	-0.460407000
N	0.478852000	0.055022000	0.187064000
C	1.800864000	-0.049240000	-0.426562000
C	-0.321676000	-1.940265000	-0.955682000
C	-0.538388000	-0.465942000	-0.722353000
C	-1.280372000	-2.823218000	-1.293621000
C	0.218361000	1.377623000	0.780177000
P	-0.883910000	1.321100000	2.267738000
O	-2.265404000	0.666518000	1.739571000
O	-0.238304000	0.240850000	3.258300000
C	-3.470936000	0.815447000	2.539094000
C	-4.668348000	0.520056000	1.664826000
C	-0.294311000	-1.199641000	3.135725000
C	-0.098012000	-1.797489000	4.512020000
H	-3.415349000	0.118391000	3.377568000
H	-3.498123000	1.829469000	2.935964000
H	0.487282000	-1.514115000	2.447152000

H	-1.256327000	-1.487421000	2.710867000
H	-0.110070000	-2.886524000	4.444870000
H	0.858861000	-1.489550000	4.932503000
H	-0.888778000	-1.483246000	5.193055000
H	-4.614563000	-0.484766000	1.247041000
H	-5.581049000	0.597857000	2.257600000
H	-4.735671000	1.232818000	0.843062000
H	1.839527000	0.503648000	-1.381658000
H	-0.526049000	0.081027000	-1.680585000
O	-1.019869000	2.634501000	2.924279000
H	2.530285000	0.416143000	0.236259000
H	-1.519082000	-0.313594000	-0.279938000
H	1.150557000	1.652125000	1.280337000
C	-3.549022000	-3.743462000	-1.411152000
C	-4.637360000	-1.342261000	-2.260054000
C	-5.463219000	-2.444366000	-2.069301000
C	-4.911548000	-3.648899000	-1.644964000
H	-3.122027000	-4.686395000	-1.094685000
H	-5.054951000	-0.409104000	-2.614183000
H	-6.524620000	-2.369405000	-2.263804000
H	-5.542701000	-4.516601000	-1.507004000
C	-2.701469000	-2.633800000	-1.567490000
C	-3.274962000	-1.431672000	-2.010752000
H	-2.651775000	-0.571491000	-2.199580000
H	-0.940474000	-3.852053000	-1.344702000

C	3.425601000	-1.982814000	-0.647307000
C	4.938313000	0.033550000	-0.909632000
C	4.712836000	-1.302969000	-0.544432000
C	6.198417000	0.602200000	-0.787382000
C	5.809373000	-2.047550000	-0.078810000
C	7.063592000	-1.474601000	0.057136000
C	7.263102000	-0.143630000	-0.294637000
H	6.351601000	1.630586000	-1.086508000
H	5.661175000	-3.086714000	0.185185000
H	7.888499000	-2.067552000	0.428808000
H	8.242861000	0.304422000	-0.198409000
H	3.483420000	-3.063602000	-0.719233000
H	4.138262000	0.622469000	-1.331066000
O	1.280099000	-3.690513000	-0.811258000

B13

C	-0.222452000	2.951573000	-0.506882000
C	1.077359000	2.255380000	-0.241211000
F	-4.344267000	-4.122223000	-1.616555000
C	-2.101344000	-2.849116000	0.914471000
C	-3.110680000	-3.592748000	0.318389000
C	-3.361838000	-3.407037000	-1.029119000
C	-2.640912000	-2.505149000	-1.790999000
C	-1.633284000	-1.770382000	-1.178150000
C	-1.352948000	-1.931693000	0.178655000

H	-1.887201000	-2.996546000	1.964361000
H	-3.695176000	-4.306521000	0.880855000
H	-2.870789000	-2.384534000	-2.840124000
H	-1.067113000	-1.047978000	-1.748043000
N	-0.158005000	0.216844000	0.301235000
C	1.051487000	0.989844000	0.581339000
C	-1.467960000	2.282058000	-0.012283000
C	-1.324093000	0.986821000	0.745425000
C	-2.624014000	2.937667000	-0.213619000
C	-0.226457000	-1.145753000	0.839358000
P	1.295167000	-2.206570000	0.728468000
O	1.950386000	-1.898560000	-0.709328000
O	2.269310000	-1.500673000	1.793541000
C	2.321866000	-2.959673000	-1.632190000
C	1.987005000	-2.529983000	-3.042902000
C	3.472855000	-2.169606000	2.247849000
C	3.968758000	-1.462230000	3.488839000
H	3.393866000	-3.128143000	-1.517574000
H	1.798464000	-3.870014000	-1.346469000
H	3.236731000	-3.214708000	2.446749000
H	4.214418000	-2.122669000	1.447600000
H	4.881247000	-1.942022000	3.845917000
H	4.190210000	-0.416236000	3.279212000
H	3.223699000	-1.508067000	4.282652000
H	2.495879000	-1.602383000	-3.304179000

H	2.305333000	-3.303655000	-3.743705000
H	0.914262000	-2.382906000	-3.161334000
H	1.105053000	1.258837000	1.652395000
H	-1.234583000	1.211750000	1.825721000
O	1.045365000	-3.641778000	0.985829000
H	1.925242000	0.392511000	0.376321000
H	-2.210518000	0.376264000	0.634242000
H	-0.410130000	-1.138308000	1.927144000
C	-4.545412000	1.297715000	-0.016322000
C	-6.157934000	3.330756000	0.975337000
C	-6.674994000	2.048383000	0.829291000
C	-5.866746000	1.038023000	0.320244000
H	-3.948058000	0.506654000	-0.445693000
H	-6.782616000	4.128422000	1.354793000
H	-7.702932000	1.841586000	1.095060000
H	-6.267721000	0.043666000	0.176084000
C	-4.002029000	2.579201000	0.144282000
C	-4.843046000	3.594913000	0.621611000
H	-4.450118000	4.598441000	0.723279000
H	-2.510011000	3.912094000	-0.677976000
C	2.184022000	2.860968000	-0.705594000
C	4.547091000	3.492532000	-0.464532000
C	3.598407000	2.474529000	-0.641902000
C	5.904047000	3.210516000	-0.409274000
C	4.065937000	1.164205000	-0.812569000

C	5.425622000	0.886174000	-0.775900000
C	6.350077000	1.902809000	-0.562693000
H	6.613933000	4.012937000	-0.258866000
H	3.371273000	0.361851000	-1.012109000
H	5.765999000	-0.130106000	-0.925722000
H	7.408270000	1.680301000	-0.532816000
H	1.999044000	3.821045000	-1.177044000
H	4.206585000	4.514933000	-0.361720000
O	-0.268305000	4.009756000	-1.114962000

B14

C	0.285511000	-3.131876000	0.324722000
C	1.497522000	-2.319339000	0.000796000
F	-4.938813000	3.252402000	1.338408000
C	-2.358339000	2.286013000	-0.998898000
C	-3.530106000	2.845837000	-0.503555000
C	-3.801062000	2.718479000	0.845884000
C	-2.945711000	2.050585000	1.705488000
C	-1.779174000	1.500158000	1.192625000
C	-1.469237000	1.613786000	-0.163371000
H	-2.141134000	2.370921000	-2.056075000
H	-4.227403000	3.363978000	-1.146138000
H	-3.196138000	1.968322000	2.753589000
H	-1.100930000	0.968453000	1.843236000
N	0.010204000	-0.355994000	-0.244418000

C	1.340505000	-0.906971000	-0.500644000
C	-1.036087000	-2.557161000	-0.078520000
C	-1.005128000	-1.247116000	-0.817087000
C	-2.129177000	-3.292256000	0.181258000
C	-0.184295000	1.020542000	-0.715946000
P	1.255161000	2.160262000	-0.409609000
O	0.670115000	3.627440000	-0.708746000
O	1.378113000	2.136110000	1.181543000
C	0.994347000	4.358652000	-1.916732000
C	1.239031000	5.809202000	-1.562511000
C	2.432914000	2.866506000	1.858661000
C	2.428611000	2.460740000	3.314830000
H	1.868490000	3.904543000	-2.380767000
H	0.143908000	4.261957000	-2.594198000
H	2.237170000	3.933831000	1.744140000
H	3.383349000	2.630635000	1.379384000
H	2.617761000	1.393083000	3.420587000
H	3.209594000	3.003388000	3.849449000
H	1.470338000	2.691705000	3.779268000
H	2.104343000	5.908418000	-0.907314000
H	1.430283000	6.383106000	-2.470756000
H	0.372314000	6.238740000	-1.060660000
H	1.576401000	-0.866293000	-1.579003000
H	-0.784478000	-1.435134000	-1.885439000
O	2.489487000	1.864003000	-1.177174000

H	2.076564000	-0.281511000	-0.004556000
H	-1.971442000	-0.759746000	-0.785024000
H	-0.241656000	1.053370000	-1.819812000
C	-4.355613000	-4.067713000	-0.535277000
C	-5.508983000	-1.589926000	-0.077680000
C	-6.288479000	-2.633879000	-0.563087000
C	-5.705877000	-3.876184000	-0.788713000
H	-3.910628000	-5.040981000	-0.698272000
H	-5.958008000	-0.627219000	0.127314000
H	-7.343539000	-2.484741000	-0.749461000
H	-6.306215000	-4.699777000	-1.151849000
C	-3.547366000	-3.016151000	-0.080694000
C	-4.153741000	-1.776290000	0.159027000
H	-3.573809000	-0.963545000	0.571885000
H	-1.921768000	-4.261551000	0.623974000
C	2.687417000	-2.930443000	0.178161000
C	4.461470000	-1.313700000	-0.691072000
C	4.061558000	-2.491351000	-0.033220000
C	5.806846000	-1.001188000	-0.827680000
C	5.073528000	-3.336534000	0.459256000
C	6.414640000	-3.018981000	0.325490000
C	6.788153000	-1.844584000	-0.319709000
H	6.086169000	-0.089706000	-1.338924000
H	4.787322000	-4.253611000	0.957834000
H	7.168465000	-3.687386000	0.719752000

H	7.834378000	-1.592416000	-0.430738000
H	2.602782000	-3.938609000	0.568608000
H	3.740639000	-0.632893000	-1.112406000
O	0.366697000	-4.217058000	0.880746000

B15

C	-0.120242000	-3.142399000	-0.420340000
C	1.151624000	-2.533721000	0.078612000
F	4.899941000	3.234884000	-1.630285000
C	1.751471000	1.472345000	-1.393940000
C	2.908085000	2.014962000	-1.938541000
C	3.771131000	2.710464000	-1.108919000
C	3.515826000	2.878733000	0.239982000
C	2.353636000	2.328331000	0.768364000
C	1.461827000	1.622287000	-0.036251000
H	1.064659000	0.920894000	-2.019824000
H	3.143938000	1.905287000	-2.987447000
H	4.217926000	3.421285000	0.856741000
H	2.143101000	2.450423000	1.822905000
N	0.031134000	-0.363180000	0.128225000
C	1.042363000	-1.204429000	0.773837000
C	-1.376857000	-2.355046000	-0.211487000
C	-1.293663000	-0.946866000	0.327786000
C	-2.523513000	-2.979688000	-0.546209000
C	0.183455000	1.036016000	0.541861000

P	-1.229260000	2.142224000	0.051574000
O	-2.252369000	1.883782000	1.262840000
O	-0.719798000	3.628052000	0.396755000
C	-3.523024000	2.579766000	1.298089000
C	-4.220624000	2.225605000	2.592236000
C	-0.318127000	4.566777000	-0.634784000
C	-0.676980000	5.964117000	-0.179716000
H	-3.335128000	3.652861000	1.237480000
H	-4.108225000	2.280075000	0.428210000
H	0.757784000	4.463563000	-0.776587000
H	-0.821266000	4.302385000	-1.563480000
H	-0.193822000	6.201606000	0.767973000
H	-0.344865000	6.688835000	-0.924763000
H	-1.754645000	6.071494000	-0.055982000
H	-3.622339000	2.529260000	3.450869000
H	-5.182499000	2.737704000	2.644389000
H	-4.399628000	1.152754000	2.660028000
H	1.994192000	-0.688135000	0.770267000
H	-2.025863000	-0.336355000	-0.196241000
O	-1.758173000	1.981825000	-1.320785000
H	0.779753000	-1.367494000	1.836933000
H	-1.564539000	-0.938185000	1.399269000
H	0.220782000	1.134514000	1.638959000
C	-4.843453000	-3.272901000	-1.257930000
C	-5.761485000	-1.202348000	0.331900000

C	-6.655232000	-1.903919000	-0.469048000
C	-6.189284000	-2.945123000	-1.264776000
H	-4.486801000	-4.090316000	-1.871163000
H	-6.120525000	-0.404460000	0.968540000
H	-7.706333000	-1.648385000	-0.465378000
H	-6.876561000	-3.505272000	-1.884550000
C	-3.917867000	-2.559446000	-0.476712000
C	-4.410937000	-1.520622000	0.329348000
H	-3.746658000	-0.975807000	0.980496000
H	-2.375561000	-3.967849000	-0.967911000
C	2.275683000	-3.252950000	-0.067921000
C	4.261009000	-1.693340000	0.040788000
C	3.659821000	-2.930694000	0.304552000
C	5.585369000	-1.458501000	0.385008000
C	4.442676000	-3.931480000	0.896474000
C	5.760444000	-3.690341000	1.256692000
C	6.337242000	-2.450484000	1.004330000
H	6.032875000	-0.499847000	0.158893000
H	4.003481000	-4.903343000	1.081937000
H	6.341165000	-4.474149000	1.724668000
H	7.368068000	-2.264102000	1.274110000
H	2.128093000	-4.238607000	-0.498210000
H	3.703347000	-0.923185000	-0.472824000
O	-0.131492000	-4.236550000	-0.963388000

B16

C	0.049576000	2.903133000	-0.516007000
C	1.306177000	2.155670000	-0.190493000
F	-4.862693000	-3.600380000	-1.543015000
C	-1.823862000	-1.660580000	-1.270450000
C	-2.952544000	-2.250907000	-1.823719000
C	-3.761667000	-3.027063000	-1.012514000
C	-3.479894000	-3.228532000	0.326086000
C	-2.347574000	-2.629330000	0.862797000
C	-1.510874000	-1.839996000	0.077216000
H	-1.180633000	-1.043966000	-1.881263000
H	-3.207588000	-2.117293000	-2.865359000
H	-4.135269000	-3.841873000	0.927547000
H	-2.110975000	-2.788836000	1.906263000
N	-0.093053000	0.167855000	0.193892000
C	1.215354000	0.775891000	0.414314000
C	-1.233110000	2.318911000	-0.016453000
C	-1.133877000	1.033449000	0.758599000
C	-2.353144000	3.028678000	-0.232166000
C	-0.258045000	-1.208371000	0.669692000
P	1.136563000	-2.404200000	0.392467000
O	1.584226000	-2.076487000	-1.106690000
O	2.376869000	-1.890635000	1.293429000
C	2.494279000	-2.956534000	-1.816927000
C	2.467539000	-2.581820000	-3.281127000

C	2.911765000	-2.686596000	2.382906000
C	2.445860000	-2.156854000	3.723343000
H	3.492538000	-2.828862000	-1.394071000
H	2.175286000	-3.986655000	-1.658963000
H	2.611586000	-3.722908000	2.241273000
H	3.994823000	-2.614721000	2.287733000
H	2.918438000	-2.724898000	4.526638000
H	2.710669000	-1.106646000	3.846566000
H	1.365849000	-2.259747000	3.830937000
H	2.768179000	-1.544244000	-3.423178000
H	3.157261000	-3.219646000	-3.835886000
H	1.468648000	-2.714102000	-3.695423000
H	1.448134000	0.825953000	1.494099000
H	-0.905455000	1.259077000	1.817994000
O	0.765815000	-3.806026000	0.686503000
H	1.974434000	0.149427000	-0.043339000
H	-2.077746000	0.504252000	0.756077000
H	-0.351118000	-1.240355000	1.769883000
C	-4.353314000	1.493944000	0.024584000
C	-5.864559000	3.644330000	0.926784000
C	-6.444269000	2.384065000	0.831799000
C	-5.686357000	1.314729000	0.367753000
H	-3.796068000	0.657264000	-0.370831000
H	-6.449363000	4.487095000	1.270655000
H	-7.481713000	2.239406000	1.101779000

H	-6.136060000	0.336430000	0.262761000
C	-3.747484000	2.752718000	0.134453000
C	-4.537657000	3.827539000	0.567215000
H	-4.095125000	4.813550000	0.629048000
H	-2.188584000	3.983340000	-0.721885000
C	2.459987000	2.792766000	-0.474926000
C	4.327885000	1.358936000	0.497305000
C	3.858081000	2.417657000	-0.298876000
C	5.686353000	1.092641000	0.595607000
C	4.815136000	3.199461000	-0.970595000
C	6.169837000	2.926365000	-0.878093000
C	6.612906000	1.867169000	-0.093120000
H	6.023890000	0.275894000	1.219909000
H	4.476023000	4.029815000	-1.576362000
H	6.880882000	3.542283000	-1.412282000
H	7.670113000	1.652801000	-0.012152000
H	2.317386000	3.760460000	-0.943199000
H	3.641383000	0.747661000	1.059416000
O	0.072127000	3.949730000	-1.145921000

B17

C	0.820441000	-2.285951000	0.292536000
C	1.935025000	-1.558819000	-0.392246000
F	-4.998807000	3.535481000	-1.718460000
C	-2.326264000	1.873295000	0.063172000

C	-3.578736000	2.408819000	-0.215930000
C	-3.787009000	3.010091000	-1.442522000
C	-2.788651000	3.090941000	-2.397579000
C	-1.545968000	2.549328000	-2.100722000
C	-1.293410000	1.937374000	-0.871134000
H	-2.158052000	1.404821000	1.021753000
H	-4.383759000	2.363466000	0.503356000
H	-2.987684000	3.570740000	-3.345178000
H	-0.757552000	2.598241000	-2.840644000
N	0.244400000	0.047283000	-1.276840000
C	1.572762000	-0.515782000	-1.437346000
C	-0.571756000	-2.060452000	-0.213467000
C	-0.790188000	-0.967806000	-1.237852000
C	-1.527906000	-2.871574000	0.278848000
C	0.088424000	1.348912000	-0.638667000
P	0.766158000	1.527644000	1.093228000
O	0.237170000	2.952759000	1.630835000
O	-0.051929000	0.454146000	1.956274000
C	1.129384000	4.035675000	1.992781000
C	1.041513000	5.157294000	0.978891000
C	0.377630000	0.130621000	3.309937000
C	-0.443572000	-1.034366000	3.811963000
H	0.801886000	4.371949000	2.976470000
H	2.142888000	3.646162000	2.073067000
H	1.439171000	-0.111562000	3.281917000

H	0.233766000	1.016461000	3.931907000
H	-0.154541000	-1.261484000	4.839607000
H	-1.507274000	-0.796035000	3.801204000
H	-0.268119000	-1.918848000	3.202255000
H	0.014474000	5.504652000	0.869714000
H	1.657297000	5.997779000	1.304274000
H	1.402991000	4.832425000	0.002787000
H	1.612863000	-0.996163000	-2.425450000
H	-0.835554000	-1.420067000	-2.239387000
O	2.245699000	1.405737000	1.141504000
H	2.302771000	0.286242000	-1.451515000
H	-1.754493000	-0.493847000	-1.075242000
H	0.794687000	2.010739000	-1.155165000
C	-3.590434000	-2.473459000	-1.154736000
C	-5.129310000	-3.736353000	0.785663000
C	-5.737755000	-3.232938000	-0.359454000
C	-4.960450000	-2.610584000	-1.329363000
H	-3.012314000	-2.013398000	-1.939858000
H	-5.721736000	-4.233951000	1.541892000
H	-6.805445000	-3.334820000	-0.499854000
H	-5.422379000	-2.233567000	-2.232128000
C	-2.958022000	-2.960465000	0.001185000
C	-3.760193000	-3.610502000	0.955384000
H	-3.290196000	-4.013729000	1.843112000
H	-1.176221000	-3.566658000	1.033737000

C	3.184098000	-1.959800000	-0.105610000
C	5.433239000	-2.341296000	-1.063350000
C	4.460867000	-1.438277000	-0.613990000
C	6.657436000	-1.892745000	-1.538396000
C	4.770130000	-0.072093000	-0.605436000
C	6.003543000	0.371128000	-1.065262000
C	6.945877000	-0.532386000	-1.544250000
H	7.389952000	-2.605128000	-1.894404000
H	4.060892000	0.629243000	-0.187159000
H	6.233325000	1.428340000	-1.038626000
H	7.903546000	-0.181060000	-1.904858000
H	3.255978000	-2.803961000	0.572957000
H	5.217811000	-3.402268000	-1.047648000
O	1.040641000	-3.048475000	1.224440000
